# Time and change: a typology for presenting research findings in qualitative longitudinal research

**DOI:** 10.1186/s12874-023-02105-1

**Published:** 2023-12-06

**Authors:** Åsa Audulv, Thomas Westergren, Mette Spliid Ludvigsen, Mona Kyndi Pedersen, Liv Fegran, Elisabeth O. C. Hall, Hanne Aagaard, Nastasja Robstad, Åsa Kneck

**Affiliations:** 1 Department of Nursing, Umeå University, Umeå, Sweden; 2 Department of Health and Nursing Science, University of Agder, Kristiansand, Norway; 3 Department of Public Health, University of Stavanger, Stavanger, Norway; 4 Department of Clinical Medicine, Randers Regional Hospital, Aarhus University, Aarhus, Denmark; 5 Faculty of Nursing and Health Sciences, Nord University, Bodø, Norway; 6 Centre for Clinical Research, North Denmark Regional Hospital, Hjørring, Denmark; 7 Department of Clinical Medicine, Aalborg University, Aalborg, Denmark; 8 Faculty of Health, Aarhus University, Aarhus, Denmark; 9 Faculty of Health Sciences and Nursing, University of Faroe Islands, Torshavn, Faroe Islands; 10 Lovisenberg Diaconal University College, Oslo, Norway; 11 Department of Health Care Sciences, Marie Cederschöld University, Stockholm, Sweden

**Keywords:** Longitudinal studies, Method study, Qualitative research, Research design, Repeated data collection, Typology

## Abstract

**Background:**

Qualitative longitudinal research (QLR) is an emerging methodology used in health research. The method literature states that the change in a phenomenon through time should be the focus of any QLR study, but in empirical studies, the analysis of changes through time is often poorly described, and the emphasis on time/change in the findings varies greatly. This inconsistency might depend on limitations in the existing method literature in terms of describing how QLR studies can present findings. The aim of this study was to develop and describe a typology of alternative approaches for integrating time and/or change in QLR findings.

**Methods:**

In this method study, we used an adapted scoping review design. Articles were identified using EBSCOhost. In total, methods and results sections from 299 QLR articles in the field of health research were analyzed with inspiration from content analysis.

**Results:**

We constructed a typology of three types and seven subtypes. The types were based on the underlying structural principles of how time/change was presented: Type A) Findings have a low utilization of longitudinal data, Type B) Findings are structured according to chronological time, and Type C) Findings focus on changes through time. These types differed in 1) the way the main focus was on time, change or neither; 2) the level of interpretation in the findings; and 3) how theoretical understandings of time/change were articulated in the articles. Each type encompassed two or three subtypes that represented distinct approaches to the aim and results presentation of QLR findings.

**Conclusions:**

This method study is the first to describe a coherent and comprehensive typology of alternative approaches for integrating time/change into QLR findings in health research. By providing examples of various subtypes that can be used for results presentations, it can help researchers make informed decisions suitable to their research intent.

**Supplementary Information:**

The online version contains supplementary material available at 10.1186/s12874-023-02105-1.

## Introduction

Qualitative longitudinal research (QLR) is an emerging methodology that is currently being applied in many fields of health research [1–3]. QLR (also called longitudinal qualitative research) is used to investigate how time and/or change occur in specific phenomena or contexts by following the same sample or setting(s) across a period of time [4]; data are collected continuously or recurrently either at shorter time points or in longer time waves [5]. Recent large reviews of QLR studies have shown contradictions between how empirical QLR studies are conducted and recommendations from the QLR method literature [2, 3, 6]. Most importantly, in empirical QLR studies, change is often not the main phenomenon of the study, in contrast to QLR method literature recommendations [2, 6]. This inconsistency might be caused partly by limitations in the existing method literature in describing how QLR studies could (or should) present often comprehensive and complex findings and how elements of time/change could be integrated into the presentation of results.

## Background

QLR has its roots in sociohistorical studies of the 1960s and 1970s, for example, to investigate life transitions and societal development from a micro perspective. However, over the last two decades, the number of published QLR studies has increased substantially [4], which makes sense since most health research topics entail time/change. QLR can be used to investigate changes that occur over a few days at an intensive care unit or over decades with a focus on health precursors. It can be conducted with emphases on different entities and topics, such as individuals changing identities, families adjusting processes, or the implementation of guidelines in organizations. Previous studies have argued that the various possibilities for data collection procedures and data materials make QLR an intriguing approach, and this flexibility opens possibilities for innovation and creativity [2, 7].

The researcher´s relation to and reflection about time influence how longitudinal data are collected and analyzed. It is important that authors explicitly articulate their theoretical understanding of time so that readers may understand the premises of their findings [8]. Time can be understood either as chronological (e.g., fixed time, clock time) or as subjective and fluid (experiences of time) [5, 9]. Chronological time is continuous, measurable, and objective; an hour is always an hour long, and events happen in a certain order. In contrast, fluid time concerns experiences of time; some hours are felt to be longer, some are more important, and some are life changing. When fluid time is accounted for, participants’ stories of important events are in focus and narrated in light of previous experiences and future expectations. Furthermore, in QLR, there is a distinction between the terms over time (synchronic), through time (diachronic), and across time. When data are collected over time, the specific time points of the data collection are in focus, but the time in between is not [4, 5, 9]. For example, a research team might be interested in elderly people’s everyday activities; the respondents are interviewed on several occasions, and in each interview, they are asked to describe their daily activities. In this kind of study, one “snapshot” of the elderly people’s everyday life is created for each time point of the data collection. In contrast, a through-time (diachronic) perspective aims to also capture experiences in between the actual time points of data collection. Instead of several “snapshots”, the research team tries to capture whole sequences. For example, an investigation might focus on how new fathers take on the new-father role. Fathers are interviewed at multiple time points and are asked at each interview about what happened since the last time point of data collection, and topics from previous interviews are brought into the following interviews. Across time is not a perspective in its own right, but the term is used when there is unclearness regarding if an over time or through time perspective is used. In this article, we will use QLR over time and QLR through time accordingly to their different meanings, and we will use the expression across time when referring to both perspectives or when neither approach is explicit.

Change is another central concept in QLR. Saldaña [5] describes change to be contextual and multifactorial. Change cannot occur without some time passing, but time passing does not guarantee that change occurs [5]. When investigating change, Saldaña encourages researchers to look for what increases/decreases, emerges/ceases, is cumulative, or surges through time. However, the possibility of no change must also be considered. Therefore, what is constant or missing is also important. Lewis [10] describes four types of change that occur in QLR. First, narrative change is the participants' stories unfolding across time, for example how participants describe changes in hopes, goals, or behaviors. Second, participants reinterpretation of their experiences through time. Participants can explicitly reevaluate a situation in the past or be unaware of retelling a story differently. Third, researchers also reinterpret earlier data in light of the recent. Researchers understanding of individual participants might alter through time, maybe because the researchers come to know more about the participant or the phenomenon of interest. Fourth, Lewis also emphasizes the absence of change [10]. Taken together, change can be investigated on many levels ranging from large societal changes to changes within individuals, and layers of change are often interrelated. Within QLR, change is sometimes contradictory. For example, participants in a study can describe their narrative change while researchers detect participants’ implicit reinterpretations when comparing data from different time points; thus, the participants and researchers can describe different patterns of change and even contradict each other’s perspectives.

### Rationale

A typology is a classification system used to classify entities into groups based upon similarity [11]. A typology is often built on ideal types or cases to illustrate the classes [12] and is useful for describing the various dimensions and characters of a phenomenon. Thus, a typology can clarify the conceptualization of particular areas. From an objective perspective, a typology is not true but rather is a way of picturing various dimensions of a particular phenomenon [13]. Within the area of research methodology, datasets of published articles are scrutinized to develop typologies for different research methods or practices [14–17]. Within research methods, a typology is useful for making researchers aware of various approaches to apply a method. In the research community, there are not yet agreements or guidelines on how time/change should be treated in presenting results in empirical QLR studies. Furthermore, QLR is founded in sociohistorical studies drawing on ethnographic or case-study methods [4], and it can be difficult to fit extensive QLR studies into the article format in which much research is published. Exemplifying different approaches that researchers can use to integrate time/change into QLR results sections can inform future qualitative researchers’ study decisions. Today, QLR researchers often struggle with how to analyze and present the elements of time/change [8, 18]. The aim of this study was to develop and describe a typology of alternative approaches for integrating time and/or change in QLR findings.

## Method

Method studies are research studies that investigate the practices of certain research methods to suggest method developments. There are currently no set guidelines for how method studies should be conducted, although different types of review methods are often used [19]. We recently conducted a large method study of 299 articles to better understand how longitudinal perspectives have been integrated into the data collection practices of various QLR studies in health research [6]. In this article, we use the same articles to further investigate how time/change was presented in results sections. For the data collection, we used an adapted scoping review methodology [20–22] that has been reported in full elsewhere [6]. The adaptations of the scoping review method concerned the inclusion of articles; we used a large subsample of studies published during a three-year period, but not all QLR articles published, further, we did not include grey literature. Here, we provide only a brief description of the literature searches, data extraction and charting, while we describe the data analysis in more detail.

The notions leading to the development of this proposed typology have been discussed among the authors for a few years. The first insight about QLR results being presented differently across articles arose in discussions between ÅA and ÅK in 2016. We were then working on a publication to describe QLR analysis [18] and scrutinized approximately 50 empirical QLR articles as part of that work. Based on those 50 articles, we formulated some preliminary ideas for analyzing and presenting QLR studies, and that work was presented at a qualitative research method conference [23] and at research seminars. In 2019, further work on understanding the QLR method was undertaken within a larger research group (LF, MSL, TW, MKP, HA, EH). A method study with systematic searches was conducted to map how QLR articles within the existing health research literature were designed to capture aspects of time/change [6]. Database searches were conducted in Medline and Cinahl through the EBSCOhost interface and yielded 2895 records. Duplicates were removed (*n* = 715), and records were screened (*n* = 2180) and assessed for inclusion independently by two reviewers (*n* = 349). In total, 299 qualitative empirical articles were included (for a full list of the articles, see additional file 1). The included articles met the criteria of 1) being in the field of health research, 2) having longitudinal qualitative data collection, and 3) having been published between 2017 and 2019. To develop the typology presented here, we extracted the following parts of the included articles: aims, methods sections (e.g., primarily the analysis description) and results sections. The analysis was conducted in a three-phase approach inspired by qualitative content analysis [24] being part of the scoping review methodology, as suggested by Pollock and colleagues [25].

### Familiarization

In the first phase of the qualitative content analysis, we familiarized ourselves with the extracted data. The article results sections were read with a focus on how aspects of time/change were incorporated into the results sections. For example, we highlighted how results were presented (e.g., names of themes, use of descriptive cases or figures) and how elements of time/change were described in the results (e.g., use of words such as “before”, “after”, “over six months”; use of several quotations from the same participant from different time points; use of temporal aspects in contextual descriptions).

### Identifying types and subtypes

In the next stage, a preliminary typology was created by grouping articles with similarities in their results presentations. Each group of articles was developed into a subtype with a specific approach to incorporating time/change into the results. For example, in Subtype B1 (Recurrent cross-sectional approach), the data from each time point during data collection were presented in isolation, creating a sequential series of snapshots. Then, the overarching types were created, determining the underlying structural principles of how time/change was presented. Each type encompassed two or more subtypes. To further understand the properties of each type/subtype, data were compared and contrasted both within and across types/subtypes.

### Determining the typology

In the third phase, we determined the typology by discussing the types/subtypes and their internal relationships. All authors in the research group were assigned to review approximately ten articles representing different subtypes. These articles and the final typology were later discussed within the group to reach consensus regarding how to define and describe how elements of time/change differed between types and subtypes.

## Results

**Fig. 1:**
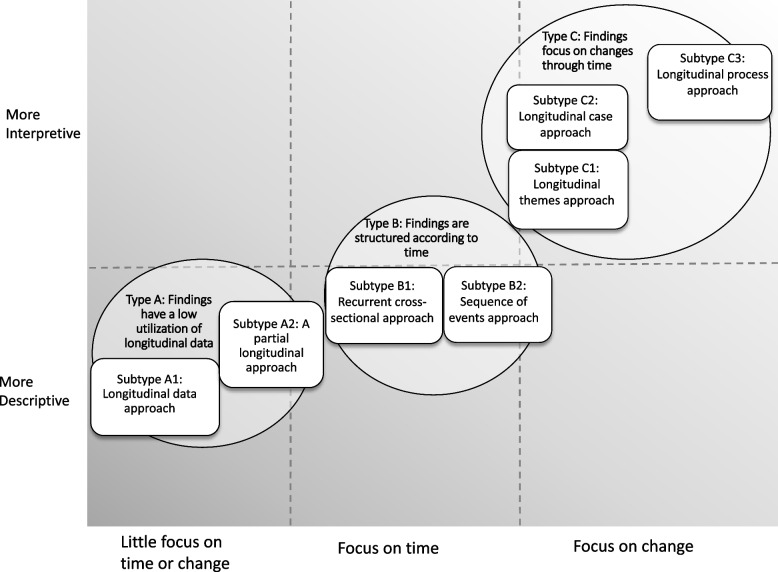
The relationships between types and subtypes in relation to interpretation level and primary focus on elements of time/change]

Our analysis of the 299 articles resulted in one typology encompassing three types of structural principles, which in turn included a total of seven subtypes (see Fig. 1). Most of the articles fitted within one type and one subtype. However, some articles, particularly those that divided their results according to two or more research questions, had elements of several subtypes.

Types differed regarding how time or change was used in the results presentation. In Type A articles, time/change played a minor role, mostly adding to the contextual description of the study. In Type B articles, time was used to organize the results presentation into a logical whole, whereas in Type C articles, the results presentation focused on change through time. Regarding the conceptualization of time, Type A and B articles often used an over-time perspective, while Type C articles attempted a through-time perspective. In general, Type C articles were interpretive, reaching for conceptual and sometimes theoretical understanding, whereas Type A and Type B articles tended to be more descriptive.

### Type A: Findings have a low utilization of longitudinal data

**Fig. 2:**
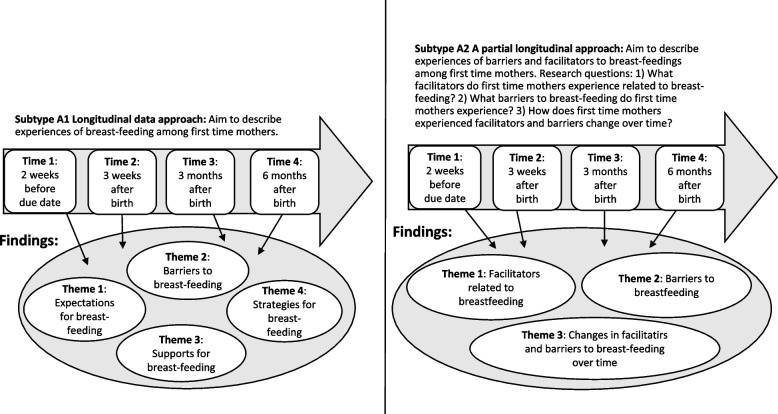
A schematic figure of what aim, longitudinal data collection and findings can look like in Subtypes A1 and A2.]

In Type A articles, researchers used serial data collection practices to collect rich data. Time/change was seldom explicitly conceptualized. In these articles, elements of time/change were of subordinate interest; instead, the emphasis was on the properties of the phenomena of interest. From a time/change perspective, these articles did not use the full potential of the collected longitudinal data. Results presentations were typically organized by themes capturing dimensions of a phenomenon. Two subtypes were identified (see Fig. 2).

#### Subtype A1: Longitudinal data approach

In Subtype A1 articles, longitudinal data collection was used to ensure that rich data material was collected. These articles often described, in their rationale, the choice of serial interviews or longitudinal data collection to capture participants’ experiences of different situations and thus create multifaceted data (see Table 1). The findings of these articles focused upon the studied phenomenon, and aspects of time/change were seldom mentioned in the results sections. However, words referring to time aspects (such as “later” or “after”) were used occasionally.

**Table 1:** Characteristics and example of Subtype A1

Characteristics of Subtype A1: Longitudinal data approach
- Data are collected over time and thus capture participants’ experiences in various situations - Aim does not emphasis time/change - Findings consist of themes capturing various aspects of a phenomenon - None, or very few, examples or descriptions relate to the passing of time or change
**Example**: Felice and colleagues [26] investigated mothers' perceptions of, attitudes toward, and practices in pumping and providing pumped human milk to their infants. Twenty mothers were interviewed between two and eight times starting in late pregnancy and during the child’s first year. Aspects such as motivation, practices, and attitudes toward pumping breast milk change during the first year of being a mother. The results were presented through the following themes: 1) types of pumps used, 2) mothers’ motivations for pumping, 3) mothers’ pumping practices, and 4) mothers’ attitudes toward and perceptions of pumping. Neither the aim nor the themes reflected elements of time/change; however, the longitudinal data collection contributed to data material with nuances and complexities

#### Subtype A2: Partial longitudinal approach

In Subtype A2 articles, the results were presented in several themes, and one of the themes focused on change across time. These articles typically had several research questions, with one focusing on change across time (see Table 2). In these articles, data from all time points of the data collection were first analyzed as one dataset, and in a final stage of the analysis, the themes/categories were organized in a time sequence to reveal changes across time.

**Table 2:** Characteristics and example of Subtype A2

Characteristics of Subtype A2: Partial longitudinal approach
- The aim is presented in several objectives/research questions, of which one (or some) focus on changes across time - The main focus of the results is to describe aspects of a phenomenon - Findings regarding changes across time are presented in one distinct part of the results section
**Example:** Richter Sundberg and colleagues aimed to investigate the bases for decisions and the decision-making process by a prioritization group of clinical experts during the development of clinical guidelines [27]. Data were collected over a period of three years in the form of observations at meetings, open-ended surveys and meeting documents. The study presented three specific research questions: 1) What decision-making criteria were used? 2) How did the composition of decision criteria change over time? 3) Did the participants encounter conflicts or dilemmas, and if so, on what subjects and how were these managed? The focus on time/change was related to research question 2. The findings of the study were then presented in three sections based on the research questions. The subheading for section three was “The decision-making process over time”. In this section, the findings were organized according to a time-line and findings described which topics were discussed early, in the middle, or in the later part of the decision-making process. The findings were also illustrated with a figure showing how the topics of discussion changed in the meetings over time

### Type B: Findings are structured according to chronological time

**Fig. 3:**
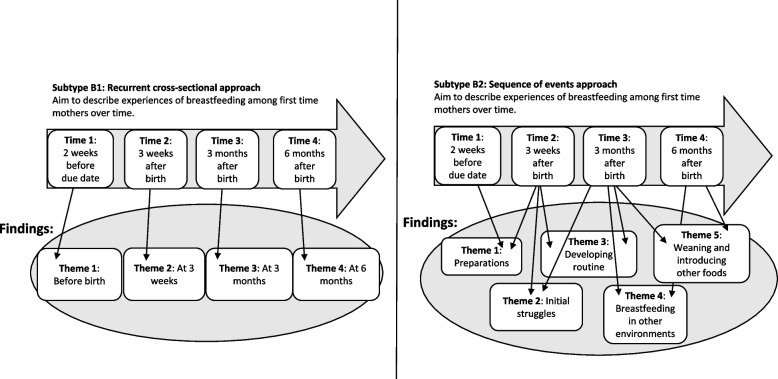
A schematic figure of what aim longitudinal data collection and findings can look like, in Subtype B1 and B2.]

In Type B articles, chronological time was used to help structuring the findings into a meaningful story (see Fig. 3). The results section typically started with an initial event (e.g., being diagnosed with a disease, retirement, starting school), and the findings then unfolded sequentially. The findings focused on the phenomenon of interest, and time provided a frame for presenting the findings in a logical way. Time was conceptualized mostly through an over-time perspective (especially for B1 Recurrent cross-sectional approach) and was often presented chronologically. Change played a minor role in these articles but describing experiences at different time points could allow the possibility of implicitly seeing change in the results. However, change was sometimes mentioned in relation to participants’ narratives or was described more explicitly, for example, in the discussion. Within Type B, two subtypes were identified.

#### Subtype B1: Recurrent cross-sectional approach

In Subtype B1 articles, data from each data collection period (e.g., time point/time wave) were analyzed separately; that is, the data were broken up into several datasets, each representing one time point of data collection (see Table 3). In the results sections, the findings were then presented chronologically according to the data collection periods. Therefore, Subtype B1 can be understood as showing a number of snapshots or as a series of cross-sectional studies. Within Subtype B1 articles, the results were mostly displayed descriptively and on a group level. The focus of the results sections was participants’ experiences at certain time points, and each time point was often described in a complete category system. In many of the Subtype B1 articles, the researchers conducted no or few analyses across the time points. Thus, change was vaguely (or not at all) described in the results sections. However, a few articles had a synthesized description of change across time either at the end of the results section or in the discussion.

**Table 3:** Characteristics and example of Subtype B1

Characteristics of B1: Recurrent cross-sectional approach
- The aim is to describe subjective experiences across time - Results are divided by time points according to the data collection and presented in chronological order - Change is vaguely (or not at all) described in the results but is sometimes addressed in the discussion section
**Example:** Lawton and colleagues [28] interviewed people with diabetes before and after testing an insulin pump with a closed-loop system. The results were presented in two isolated category systems. One category system focused on expectations before testing the pump, and the second described the experiences and consequences of using the insulin pumps. This study provided knowledge about both expectations and experienced consequences, but there were few connections between the two parts of the results

#### Subtype B2: Sequence of events approach

In Subtype B2 articles, the results sections were structured according to a chronological timeline building on a storytelling tradition where events are told sequentially. The results started at the beginning and often finished with expectations for the future. In these articles, the phenomenon of interest was the focus, and themes were arranged chronologically according to events that occurred during data collection (see Table 4). Therefore, change could be mentioned, but the focus was on describing dimensions of the phenomenon. Subtype B2 articles were often descriptive but could vary in interpretation level.

**Table 4:** Characteristics and example of Subtype B2

Characteristics of Subtype B2: Sequence of events approach
- The aim is to describe subjective experiences of a phenomenon across time - Data from all time points are treated as one dataset and inductively sorted in themes - The results are presented in a chronological order based on when events took place; thus, the results have a logical sequential structure
**Example:** Minton and colleagues investigated families’ experiences of having a relative with a prolonged critical illness in an intensive care unit [29]. Family members of six different patients were interviewed on a weekly basis during the time their relative spent in the intensive care unit (the length of stay varied between 17 and 66 days). The results were presented in themes and began with describing feelings regarding when the relatives first arrived at the hospital (Theme 1: Being overwhelmed). The results then focused upon balancing hope and fear during the initial days (Theme 2: Living in an uncertain world). New expectations and fears when the patient woke were described in Theme 3 (An altering uncertainty), and Theme 4 presented experiences related to the patient being transferred to a hospital ward (Uncertainty in a different location). Finally, thoughts around an altered future were presented in the last theme (Moving on). This example illuminates how a journey or path is created through the results, although the actual timelines differed among the families, since some went through these experiences over 17 days and others were interviewed repeatedly over 66 days

During analysis, all data material was treated as one dataset, and themes were constructed inductively to describe the experiences of a phenomenon across time. Some themes had a pronounced time element, and some did not. For example, experiences of what happened in the beginning (e.g., starting school) were often described in a theme concerning the beginning. This meant that when participants in serial interviews talked about the same experience in several interviews, all data regarding the same experience were allocated to one theme regardless of which interview these data were provided in. Thus, in Subtype B2 articles, the timeline of the results was constructed by the researchers based on the whole dataset. As a result, some participants might not have followed the chronological order of the results presentation. For example, a result presentation might have ended with a theme named “feeling at peace” even though some participants might have felt at peace at an earlier stage than at the end of data collection, whereas others might not have described feeling at peace at all.

### Type C: Findings focus on changes through time

**Fig. 4:**
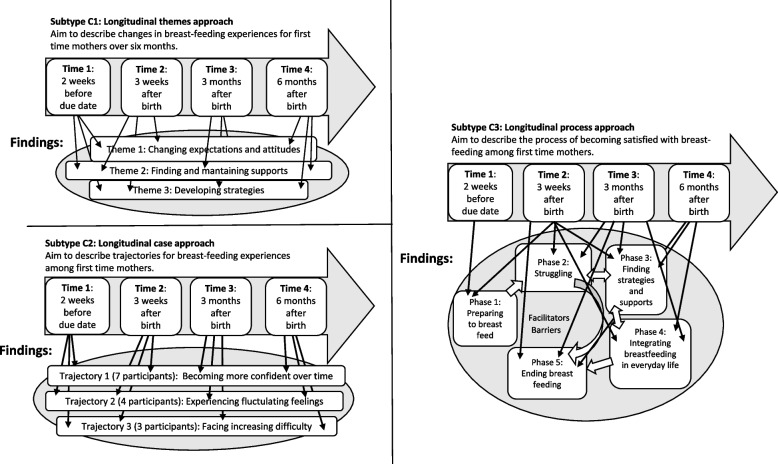
A schematic figure of what aim, longitudinal data collection and findings can look like in Subtypes C1, C2, and C3.]

These articles had a through-time perspective and often included both fluid and chronological understandings of time. In these articles, change was described from different perspectives and in several layers. The articles results were often interpretive, and in some articles conceptual models to explain change were presented in the result sections. In Type C articles, the analyses were often complex and performed in several stages, and analytic tools (such as matrices or timelines over events) were used to obtain a better understanding of change. In these articles, the QLR method literature was commonly used, and some articles also drew on other qualitative traditions, such as ethnography, phenomenology, case study or grounded theory. We identified three subtypes in Type C (see Fig. 4).

#### Subtype C1: Longitudinal themes approach

In Subtype C1 articles, the focus was on changes in a phenomenon and thus how elements/themes of the chosen phenomenon changed through time (see Table 5). One underlying assumption in Subtype C1 articles was that phenomena were constructed of several elements/themes and that these themes could change in different ways through time. In the results sections, each theme description started with describing the element at the first time point and continued with describing changes in that theme through the data collection period. During the analysis, the data material was first divided into themes, and the text representing themes was then arranged in chronological order. In some articles, the themes were constructed inductively during the first stages of the analysis; in others, predefined themes were used deductively. The results were presented at the group level.

**Table 5:** Characteristics and example of Subtype C1

Characteristics of Subtype C1: Longitudinal themes approach
- The aim is to describe changes in a phenomenon through time - Results are presented in themes and change through time is described for each theme - Typically, each theme description starts with the first time point and continues with a chronological description of change through time
**Example:** Fu and colleagues [30] investigated bereaved mothers’ longitudinal grief experiences after losing their child in an earthquake. The study used an interpretive phenomenological approach and was conducted in a Chinese sociocultural context. Six mothers were interviewed on four occasions over a period of two years. The results were presented in five themes: 1) anger toward the cause of their children’s deaths, 2) guilt and regret, 3) evolving yearning over time, 4) loss of family stability, and 5) social interactions bringing additional pressure. The themes had different change patterns. For example, the mothers’ anger (Theme 1) remained over the two years. In contrast, the mothers yearning (Theme 3), evolved from being constantly present to being triggered by certain situations to finally being controlled to situations when the mothers wanted to remember and yearn for their child. The themes also included diversity across participants; Theme 4 covered how the mothers lost their sense of being a family after their child’s death, and some mothers (but not all) consequently went through a divorce

#### Subtype C2: Longitudinal case approach

In Subtype C2, the focus was on variation in change across cases. The results sections described a number of cases or subgroups followed through time and their different change patterns (e.g., trajectories) (see Table 6). In the articles, cases consisted of different types of entities, most commonly individuals followed over time but also families or organizations. In the articles, the results sections either presented a few typical cases that represented the diversity of the whole dataset or described trajectories that each represented a subgroup of participants with similar change patterns. These articles often involved analyses based on strategies drawing on within- and across-case analysis and/or used matrices to show changes through time for each case/individual.

**Table 6:** Characteristics and example of Subtype C2

Characteristics of Subtype C2: Longitudinal case approach
- The aim is to describe trajectories, patterns, or profiles of change through time - Changes in individuals or cases are the focus - The analysis is conducted partly at an individual level, with data from each participant/case being viewed across time - The results are organized in descriptions of the changing trajectories for subgroups of participants or cases with different changing trajectories. Each subgroup/case is described from baseline and onward
**Example:** Foster and colleagues [31] explored parents’ psychosocial trajectories in the 12 months following their child’s critical injury. Twenty-seven parents were interviewed on three occasions, and data were analyzed with a longitudinal within- and across-case thematic analysis. Three different trajectories were presented in the results: Resilient trajectory (6 parents), Recovery trajectory (13 parents), and Distressed trajectory (8 parents). First, each trajectory was described generally, and thereafter, an example of a parent’s journey was provided in detail. The authors also used statistical tests to investigate associations between the parents’ trajectories and marital status, working status, and type/severity of the child’s injury but found no associations

#### Subtype C3: Longitudinal process approach

In Subtype C3 the focus was on the process of how and why phenomenon change (see Table 7). The results sections presented phases organized as a process of change, and/or incitements and barriers for change. The main focus of subtype C3 articles was to understand or give explanations to how change occur. The phases were often presented as the main findings, although the incitements or preconditions for change were sometimes in focus. Consequently, the results were presented as a synthesized model containing possible explanations for the changes. The process (and phases) as a whole was informed by data from all participants. However, all participants did not necessarily provide data to all parts of the model. The phase descriptions demonstrated change through time, but since change might not occur in the same way or according to the same timeline for all participants, data from some participants could provide more insight into particular phases. Negative cases (e.g., when a participant did not change as the researchers expected) rendered new insights into the process. It should be noted that phases could be chronological and multi-exclusive, but did not have to.

**Table 7:** Characteristics and example of Subtype C3

Characteristics of Subtype C3: Longitudinal process approach
- The aim is to investigate change processes or incitements to change - The results present stages or phases and often preconditions, incitements and consequences, thus explaining how change occurs - The results focus on the process of change and stages of change. Thus, time is of lesser interest. The change process is not necessarily chronological; multiple moves forward and backward between the stages of change are common
**Example:** Elliott and colleagues [32] investigated adolescent mothers’ housing instability across three years. They then developed a theory to describe the process of how mothers strove for independent housing, including descriptions of types of moves and factors affecting housing instability. Within this theory, horizontal moves were described as mothers moving to a similar housing situation (e.g., moving from living with one family member to another) and vertical moves as mothers moving from a dependent housing situation to an independent housing situation (e.g., an own contract). During the data collection, some mothers underwent several horizontal moves, and others experienced just one vertical move. The theory was developed by taking all the participants into account. This article provided interpretive results and suggested possible explanations and mechanisms for change

### Combination of subtypes

Most of the articles we scrutinized could be identified as presenting results according to one subtype. However, there were also several examples of articles combining subtypes (see Table 8). For example, Subtype C2 (Longitudinal case approach) was one of the least common subtypes identified in the data material but was sometimes seen in combination with other subtypes. When combined with other subtypes, C2 often played a minor role, presented as a table or a text box describing certain typical cases.

**Table 8:** Example of how subtypes could be combined

**Example of combination of subtypes**: McKay and colleagues [33] investigated staff fluctuations during an HIV prevention trial (RESPECT). The results were first presented in four themes, all including temporal aspects: 1) changes in the clients served and program maintenance, 2) changes in skill and knowledge, 3) changes in workload for remaining employees, and 4) innovative approaches to downsizing. After the themes were presented, the results continued with the presentation of two cases showing how staff changes and implementation were managed at two trial locations. In this example, the results were seen as starting according to Subtype C1 (Longitudinal themes approach) and continuing according to Subtype C2 (Longitudinal case approach). The combination of subtypes thus helped to demonstrate more angles of change	

## Discussion

QLR is a flexible methodology regarding topics, type of data and length of data collection [6]. However, its flexibility can also create difficulties when large and complex data materials are analyzed and presented, and unconscious implicit choices or interpretations by the researchers may shape how a study is reported. This study shows that qualitative longitudinal materials can be presented according to various types and subtypes depending upon the researchers’ aims, intentions, and perspectives on time/change. Type A and Type B are mainly descriptive, emphasizing subjective experiences over time, while Type C is more interpretive, focusing on how phenomena change or on precursors to change. Even within these overarching types, there are subtypes that can be used for different purposes, such as investigating individual patterns of change (Subtype C2: Longitudinal case approach) or incitements to change (Subtype C3: Longitudinal process approach). However, some qualitative articles with longitudinal data are not focused on describing elements of time or change, which is a criterion for QLR according to current method recommendations [4, 5]. Subtype A1 (Longitudinal data approach) was the most evident example in this study. We argue that future qualitative researchers should distinctly present their research either as QLR or as the use of qualitative longitudinal data (QLD). To perform QLR, a study should contain data collected across time with the same participants or settings and should focus on change.^1^ However, QLD can be used for reasons other than investigating change. Repeated data collection can, for example, generate a close relationship between researchers and participants, which in turn helps researchers collect rich and varied data [5, 34]. Furthermore, data collected over time can compensate for time-related fluctuations in a phenomenon (such as fluctuations in symptoms or seasonal changes) and might capture participants’ detailed “here and now” situation on several occasions instead of accounts given on one occasion but related to experiences over a time period [35].

One interesting finding of this study was the subtypes of describing diachronic (through time) results (e.g., Subtype C1 Longitudinal themes approach, Subtype C2 Longitudinal case approach, and Subtype C3 Longitudinal process approach). Elements of change are central in all three subtypes, but the results presentations show different angles of change. According to Neale [4], investigating change processes is the goal of QLR research, but these processes are understood by first exploring patterns of how cases and themes are enacted through time. We find Neale’s [4] analytical approach well suited to Subtype C3 studies. However, according to our study, diachronic descriptions of themes, cases and processes have their place, and rich data material could potentially be analyzed two or even three times for different research questions.

An important difference between Type B (Findings are structured according to chronological time) and Type C (Findings focus on changes through time) articles is the use of the descriptive versus the interpretive lens and that Type B relies more on a chronological time perspective. In Type B, change was only implicitly described since the focus was on describing subjective experiences at different points in time. In contrast, Type C articles used a more interpretive lens. The researchers emphasized fluid time and investigated the properties of change, variation in change patterns and/or why change occurred. Change was often analyzed both from the participants’ perspectives (e.g., explicitly asking about the experience of change) and the researchers’ interpretation of change (e.g., comparing data from different time points to understand differences).

Furthermore, there is variation in interpretation between the different subtypes within Type C. In Subtype C1 (Longitudinal themes approach) and Subtype C2 (Longitudinal case approach), the focus is on describing change through time, whereas Subtype C3 (Longitudinal process approach) focuses on why change occurs. In Subtype C3 articles, the researchers often developed theoretical understandings of causes and consequences of change, which constituted a higher level of understanding, in line with previous descriptions of longitudinal processes by Neale [4]. Although both descriptive and interpretive research are needed, overall, Type C articles were less common in our material. At the same time, Type C articles can push this research field forward since such studies can develop theoretical models to help us understand change. Possible reasons for the relative scarcity of Type C articles could be that conducting analyses that aim to understand change is both complex and time-consuming.

Our study showed that it was common that practices for analyzing the longitudinal aspects of the data were not explicitly described, which is in line with previous research [2, 3, 7]. However, as our analysis unfolded, we realized that this lack of description was closely connected to types and subtypes. We found that articles fulfilling the criteria for Type C (Findings focus on changes through time) were more explicit in their analysis descriptions and used more strategies for analyzing time/change than articles of Types A and B. For example, in Subtype B1, data were analyzed in parts, the different time points were analyzed in isolation, and chronological time was used as a deductive scheme. In Subtype B2, all data were analyzed together and a time lens was used; the time lens was often applied late in the analysis to help the researchers organize the results sequentially. In contrast, in Type C articles, the researchers analyzed data using strategies such as within- and across-case analysis, moving forward and backward between data from different time points, posing analytical questions regarding time/change, and/or using matrices to display change. In all, articles categorized as Type C often cited the QLR method literature and showed awareness of tools and practices for qualitative longitudinal analysis.

The existing QLR method literature has to a great extent been developed from the authors’ own projects and research experiences [8, 23, 36]. This might be an underlying reason for QLR analytical approaches often being described in isolation and similar approaches sometimes being described using different terminology. By drawing upon a large dataset, this study adds a comprehensive perspective and describes a range of types/subtypes for QLR results presentations. However, some of the subtypes described here are similar to previously described analytical approaches, and we have tried to align our terminology with the existing method literature. Grossoehme and Lipstein et al. [36] described two analytical approaches to QLR: recurrent cross-sectional analysis and trajectory analysis. We understand our recurrent cross-sectional approach to be the same as Grossoehme and Lipstein’s recurrent cross-sectional analysis [36] and very similar to the pool analysis^2^ described by Saldaña twenty years ago [5]. Grossoehme and Lipstein et al.’s [36] trajectory analysis is interpretive and diachronic and focuses on changes through time, but it contains no description of how to present results. Within the trajectory analysis approach, individuals’ trajectories, processes and themes are all mentioned, and a trajectory analysis might be more general and in line with Type C. Additionally, Neale [4] suggested that analyses of change should be built around a three-part logic consisting of cases, themes and processes. Longitudinal themes, cases and processes were mirrored in our results. Neale [4] suggested that change processes should be the goal of QLR research, but processes are understood by first exploring patterns of how cases and themes are enacted through time. Our results showed that the results could emphasize longitudinal themes, cases or processes and thus present change from different angles. Furthermore, Subtype C2 (Longitudinal case approach) is similar to the pattern-oriented analysis approach (POLA, previously described by our team) [18] since the focus of POLA is to describe various patterns of change. However, POLA uses an inductive analysis, and in Subtype C2 both inductively derived example cases and predefined subgroups are presented. Nevertheless, two subtypes, Subtype A2 (Partial longitudinal approach) and Subtype B2 (Sequence of events approach), have not been previously described, and no comprehensive typology has been arranged to show comparisons of types/subtypes of presenting QLR results.

### Recommendations and considerations when performing QLR

Having the option of using different subtypes when presenting QLR results opens new possibilities. All types and subtypes have benefits and can be used for different kinds of research questions; they can be chosen depending on what the researchers want to accomplish. However, data collection should also be considered since different data collection practices and data material fit better with various types/subtypes. Some recommendations can be deduced depending on the nature of the seven subtypes (see Table 9). For example, a study using Subtype B1 (Recurrent cross-sectional approach) would need to follow the same data collection plan for all participants, since results referring to Subtype B1 are presented according to the time points of the data collection. In articles categorized as Type C (Findings focus on changes through time), it might be advantageous to adapt the data collection to the participants’ experiences to capture data from the period in which the most change was happening. Further, in Type C articles using more than two data collection time points might be advisable in order to understand change in more depth. This matter raises the question of whether researchers should decide their study’s QLR approach beforehand. We suggest that in cases when the researchers have a clear picture of their intent, they can collect data material that is in line with the planned analysis and results presentation. However, researchers must be open to issues/elements that might change during a study that takes one or more years of data collection [5]. Planned courses of action might need to be altered. Nevertheless, preplanned studies and protocols may facilitate transparency concerning the justification of such alterations.

**Table 9:** Overview of recommendations and considerations for use of the various subtypes

Subtype	Recommendations	To be considered
**Subtype A1: Longitudinal data approach**	Suitable when the phenomenon of interest is expected to vary over time. Change is not the focus of the study	• Aspects of time/change are not analyzed
		• This subtype is not in line with existing definitions of QLR and should rather be identified as QLD
**Subtype A2: Partial longitudinal approach**	Suitable when the phenomenon of interest is the major focus and change over time is secondary	• A borderline case regarding QLR methodological recommendations
		• The studies present longitudinal data and results, but time/change plays a minor role and is often presented last
		• Risk of results becoming fragmentized when time/change is presented separately from the phenomenon
		• Risk that aspects of time/change are superficially described because of limited space when several research questions are presented in the results section
**Subtype B1: Recurrent cross-sectional approach**	Suitable when the focus is to describe experiences/a phenomenon across time and	• A borderline case in relation to QLR method recommendations
	1) Participants are expected to undergo similar experiences at the same pace, for example, a health-care pathway, an intervention, or an education program	• Longitudinal data collection is used, and findings consist of experiences over time. However, the diachronic (through time) perspective is often weak or lacking
	2) When different aspects of the phenomenon are in focus at different time points of data collection, for example, incitements and maintenance are addressed at two different time points during data collection	
		• Studies interviewing different groups of participants before and after could potentially yield similar results
		• The contrast between time points is important
	• Crucial to follow the same data collection plan for all participants	
		• There should not be too many data collection periods or data collection periods too close in time, which could lead to ambiguous result presentations
	• The time points should have a logical rationale connected to the phenomenon of interest	
	• It is recommended that comparisons be made between time points as a synthesized part of the results to incorporate some aspects of change	
	• Can be combined with mixed methods if quantitative data are collected at the same time points as qualitative data	
**Subtype B2: Sequence of events approach**	Suitable in projects in which the goal is to describe experiences/a phenomenon across time, especially if the phenomenon has a defined beginning and unfolds at a similar pace across participants	• The element of time is evident; change is less in focus
		• The focus on time over change might depend upon these studies’ predominantly descriptive character
		• The timeline in this subtype is logically constructed by the researcher; therefore, some participants might not add to all parts of the results
	• Not dependent on distinct time points for data collection; instead, participant-adapted data collection (where the amount of data and the tempo of data collection can vary across participants) might be advantageous	
		• During longitudinal data collection, participants can provide different information regarding the same event since memories are reconstructed over time. In Subtype B2, the results unfold chronologically, and thus, researchers must consider how to manage reconstruction of memories in the analysis and results
**Subtype C1: Longitudinal themes approach**	Suitable when the intention is to describe change through time in complex and multidimensional phenomena	• A possible challenge is displaying how the different themes coexist and interact across time. In some studies, this is shown in a conceptual model or by a few illustrative cases
	• Themes can be inductively constructed or predefined, depending upon how much is known about the phenomenon	
		• A risk might be that some themes are less thoroughly followed across the data collection, resulting in difficulties analyzing changes in themes across time
	• Predefined themes can provide the advantage of themes well integrated into interview guides and data collection strategies; inductive themes can be more accommodative of the data	
	• Can be combined with mixed methods if quantitative variables can mirror the qualitative themes	
**Subtype C2: Longitudinal case approach**	Suitable for projects in which cases are expected to have various trajectories or when contextual factors are likely to render large variations within the sample	• It is important to collect data to obtain a deep understanding of each participant/case; otherwise, there is a risk that some trajectories will be superficially described. Therefore, this subtype is sensitive to dropouts and probably needs more data from each participant/case than subtypes describing data on a group level
	• Can also be relevant if the aim is to unpack change on a more detailed level, for example, how an intervention worked for some participants but not for others	
	• Using a participant-adapted data collection strategy and/or several data collection points is probably advantageous. The data collection should be crafted around the periods of most change in participants/cases	
	• Could be combined with mixed methods if quantitative data can be divided by subgroups/trajectories	
**Subtype C3: Longitudinal process approach**	Suitable when researchers want to create qualitative explanations for change, for example, developing models and understanding what makes change happen	• Time is of lesser interest, since change processes are not necessarily chronological
		• Probably the most time-consuming and complex subtype to conduct
	• Using a participant-adapted data collection strategy and/or several data collection points is probably advantageous in order to collect nuanced data on change when most changes are happening	
	• It is probably advantageous to collect several types of data and/or add elements of theoretical sampling; such strategies may support a fuller description of the process	
	• Requires an interpretive and complex analysis	

Combining subtypes is another possibility. Various subtypes can be applied and presented in one or more papers when one large project is analyzed from different angles. Our recommendations to authors conducting QLR are to be transparent regarding the conceptualization of time/change, the analysis description, and the intentions/rationale for using longitudinal qualitative data. Future method literature might continue to explore suitable design elements, and recommendations or method combinations that would suite the use of types/subtypes in relation to various research questions.

### Strengths and limitations

A strength of our study was the large and varied data material, including 299 articles used to develop the typology. However, these articles were published in the field of health research and within a specific time period of three years. A limitation is that the data base searches were conducted in 2019, thus omitting more recent articles. Research methods, analysis strategies and ways of presenting results differ both between disciplines and over time; thus, it is possible that the inclusion of other research fields or time periods would yield additional types/subtypes. However, the typology is based upon a large data material and all types/subtypes are well represented by several studies. We also took care to explore “negative cases”, the articles that represented two subtypes or were difficult to allocate to a particular subtype. Further, the development of this typology has been on-going for quite some time (actually started in 2016) and even if this analysis included articles published between 2017–2019 we have been reading QLR articles from both before and after that time period. Another aspect supporting the taxonomy is the similarities between this typology and other QLR method literature.

Additionally, as already mentioned, typologies are theoretical constructs that build on data. Thus, some of the 299 articles fit better with our description of types/subtypes, some were on the interphase between subtypes, and some fulfilled criteria for several subtypes. This disparity was the reason why we did not calculate percentages regarding which types/subtypes were the most or least common. Instead, we described various ways of presenting results within a typology.

It should be acknowledged that this typology does not mirror the quality of the research articles; all the types/subtypes have both strengths and limitations. The categorization into types and subtypes was based upon the methods and results presentations of the articles and not the actual quality of the articles. In the analysis, we used the published text, and the article authors, having more insight into the actual research process, might have different views of how they conducted their study.

## Conclusions

The method study presented here is the first to describe a coherent yet comprehensive typology of alternative approaches for integrating time/change into QLR findings. Previous research has revealed that results presentations of QLR studies vary and must be allowed some variation depending on the research question [37]. Our typology ranges from the use of longitudinal qualitative data with no elements of time/change to advanced theoretical explanations of change, and it includes synchronic descriptions on the one hand and diachronic interpretations on the other. We argue that articles using QLD but that do not present findings related to time/change should not be called QLR. Instead, the authors of such articles should describe that they have collected qualitative longitudinal data. When researchers consider longitudinal data collection, they have several options for how to present their results. Being aware of different options can help researchers make informed decisions regarding how to plan both data collection and results presentation. Our hope is that this innovative typology for how to present research findings in qualitative longitudinal research will be advantageous for future researchers and that the typology can facilitate the transparency and repeatability of QLR studies.

### Supplementary Information

**Additional file 1. **

## Data Availability

The dataset analyzed during the current study are not publicly available due to publication rights. The dataset analyzed in this study consists of methods and results sections from published research articles. The data are available from the corresponding author on reasonable request. A table of included articles is also provided in Additional file 1.

## Footnotes

1 When change is investigated across time, non-change is also a possible result.

2 Saldaña describes the first steps of a QLR analysis as a kind of pool analysis in which data from each time pool/time point are analyzed separately; in later stages, changes between time pools are compared. A pool analysis would then be equal to the first steps of a QLR analysis.
