# Supplementary material for: Time and change: a typology for presenting research findings in qualitative longitudinal research
Source: BMC Med Res Methodol. 2023 Dec 6;23:284. doi: 10.1186/s12874-023-02105-1 (PMC10698947; doi:10.1186/s12874-023-02105-1)
Supplement: Supplementary file 1 — Additional file 1. [file 12874_2023_2105_MOESM1_ESM.docx]

## **Additional file 1: Table of included articles**

**Reference & country**: Full reference to the articles and the country/countries where data were collected.

**Aim and research questions**: Article aim and research questions as presented in the articles background. In cases were no succinct aim could be found in the background aim is extracted from the abstract. Abbreviations have been clarified.

**Qualitative methodology**: Methodology/tradition used in the individual articles. Methodology/tradition was most often extracted from the introduction of the method section or the abstract. Methodology/tradition were only extracted if it was explicitly described in the article (e.g., for example articles with the text “in this qualitative longitudinal study” were categorized as belonging to a qualitative longitudinal tradition, or the text “this study used a multiple case-study design” were categorized as case-study). If an article described several methodological traditions all are included. Specific methodological branches have been categorized in larger groupings, such as Constructive Grounded Theory as Grounded Theory.

**Method:** This column includes the article population, type(s) of data, and length of data collection. Population generally describes the included participants. Data describe the type of qualitative data material used in the article, for example individual interviews or observations. In some cases, it was unclear to what extent different kind of data were used in the analysis, and if questionnaires included qualitative data and/or were analyzed as part of a qualitative analysis. Length of data collection describes the period between the first data collection and last data collection with the same participants, cases or setting. Where possible we have extracted the wording from the articles, for example 30 days, 4 weeks or one month. In articles providing information in dates (e.g., from November 2017 until July 2019) we have made an estimate.

**Results:** In this column the findings as presented in the article abstracts are presented. Abbreviations have been clarified. Subheadings are the extracted subheadings (often, but not always, themes/subthemes) from the result sections, this will give an idea of how results were outlined and organized.

Additional information regarding this data material can be found in: Audulv, Å., Elisabeth O. C. Hall, E.O.C., Kneck, Å., Westergren, T., Fegran, L., Pedersen, M.K., Aagaard, H., Lund Dam, K. & Spliid Ludvigsen M.S.., Qualitative longitudinal research in health research: a method study. BMC Medical Research Methodology, 2022. 22: p. 255. <https://doi.org/10.1186/s12874-022-01732-4> and additional files for that publication.

| Reference & country | Aim and research questions | Qualitative methodology | Method | Results |
| --- | --- | --- | --- | --- |
| Abel, G., & Thompson, L. (2018). 'I don't want to look like an AIDS victim': A New Zealand case study of facial lipoatrophy. Health & Social Care in the Community, 26(1), 41-47.  Country: New Zealand | "This paper takes a longitudinal qualitative approach to explore one man’s struggle with the visible signs of HIV and the emotional toll this had on his life. It argues for the need for HIV treatment to look more broadly than just the reduction of viral load, and consider the physical, mental and social aspects essential for health and well- being. This paper presents a year of Tom’s emotional struggle with lipoatrophy." | Case study & qualitative longitudinal research | Population: Person with HIV and facial lipoatrophy (n=1)  Data: Interviews and pictures  Length of data collection: 12 months | “This paper looks at one man’s struggle to conceal or veil his facial lipoatrophy. His story is presented in the form of ‘selfies’ and extracts from in- depth interviews. It tells of an emotional (ongoing) journey of frustration, anger, excitement, depression and resignation which had a profound effect on his sense of social and psychological well- being.”  Subheadings: 1) August 2013, 2) December 2013, 3) April 2014, 4) August 2014 |
| Ahlstrom, L., Dellve, L., Hagberg, M., & Ahlberg, K. (2017). Women with neck pain on long-term sick leave-approaches used in the return to work process: A qualitative study. Journal of Occupational Rehabilitation, 27(1), 92-105  Country: Sweden | "The aim of this study was to identify approaches used in the RTW [Return to Work] process among women with neck pain on long-term sick leave from human service organizations." | Grounded theory | Population: Women with neck pain on long-term sick leave (n=117)  Data: Individual interviews and open ended questions in questionnairs  Length of data collection: 6 years | “Individuals expressed their coping approaches in terms of fluctuating in work status over time: either as a strategy or as a consequence. Periods of sick leave were interwoven with periods of work. The women were either controlling the interaction or struggling in the interaction with stakeholders”  Subheadings: 1) Fluctuating in work status over time (core category), 2) Fluctuating in work status over time as a strategy—controlling the interaction with stakeholders, 3) Fluctuating in work status over time as a consequence—struggling in the interaction with stakeholders |
| Albrecht, T. A., Keim-Malpass, J., Boyiadzis, M., & Rosenzweig, M. (2019). Psychosocial experiences of young adults diagnosed with acute leukemia during hospitalization for induction chemotherapy treatment. Journal of Hospice & Palliative Nursing, 21(2), 167-173.  Country: US | "...to examine the experiences of younger adults diagnosed with Acute leucemia who are actively receiving induction chemotherapy, to better direct the care delivered by oncology and palliative care clinicians." | mixed methods & qualitative longitudinal research | Population: Younger adults diagnosed with Acute leukemia (n=7)  Data: Individual interviews and diaries  Length of data collection: 4 to 6 weeks | “Three thematic classifications emerged: getting through, supported yet isolated, and information exchange preferences, which detail how these YAs [younger adults] processed and coped during treatment. The findings from this study provide important insights for nurses regarding coping mechanisms that YAs apply, which included relying on technology and social media platforms. Additionally, the YAs in this study discussed their need for information.”  Subheadings: Sample Description 1) Getting through, 2) Supported yet isolated, and 3) Information exchange preferences |
| Allan, E. (2017). Community nursing middle management: 'dealing with different people in different time zones on both sides'. British Journal of Community Nursing, 22(9), 448-457.  Country: UK | "What are CNMMs’ [Community nurse middle managers] perceptions of their role within CHPs [Community Health Partnerships]? What are CNMMs’ experiences and views of negotiating and managing change within CHPs? How do CNMMs understand the impact on themselves and others? What sense do they make of this? What does this mean in the context of wider understandings from the literature in Scotland and the UK? What implications are there for community nursing policy, practice, education and research?" | Phenomenology | Population: Community nurse middle managers (n=26)  Data: individual interviews  Length of data collection: 2 years | “CNMMs [Community nurse middle managers] perceived that their responsibilities had increased, become more complex and wider ranging. Maintaining an implicit connection with service users was a primary motivation for CNMMs. They were proud to be members of the nursing profession aligning their identity with their career history. A small but significant proportion resigned during the study and some were considering leaving the NHS [National Health Service].”  Subheadings: 1) Participant profile information, 2) Role content: shifting ground, 3) Role set: The view from the middle position, 4) Role form: How CNMMs navigated change through professional identity, 5) Overview of exemplars |
| Alonso, W. W., Kitko, L. A., & Hupcey, J. E. (2018). Intergenerational caregivers of parents with end-stage heart failure. Research & Theory for Nursing Practice, 32(4), 413-435.  Country: US | "This study was undertaken to longitudinally examine the experiences of intergenerational young adult, adult, and older adult caregivers as they cared for a parent with end-stage HF [heart failure] in the context of the caregiver empowerment model (Jones, Winslow, Lee, Burns, & Zhang, 2011)." | Grounded theory | Population: Intergenerational caregivers (n=23)  Data: Individual interviews  Length of data collection: Up to 24 months | “Five major themes were identified: caregiver resources, role management, caregiver–parent relationships, filial responsibility, and personal benefits and challenges.”  Subheadings: Sample demographics, 1) Caregiver resources, 2) Role management, 3) Caregiver–parent relationships, 4) Filial responsibility, 5) Personal benefits and challenges |
| Alvarez, S., & Schultz, J.-H. (2019). Professional and personal competency development in near-peer tutors of gross anatomy: A longitudinal mixed-methods study. Anatomical Sciences Education, 12(2), 129-137.  Country: Germany | "The purpose of this study was to explore the professional and personal competencies peer tutors of gross anatomy developed as a result of their tutoring activities. It was hypothesized that because of the unique and challenging environment of the gross anatomy course, tutors acquire competencies that match, and even go beyond those described in some of the competency catalogues used for curriculum development." | mixed methods | Population: Medical students who are peer tutors (n=24)  Data: Individual interviews and questionnairs  Length of data collection: 8 to 12 months | “It was found that most of the skills tutors developed or strengthened over one semester matched most of the core competencies described in various official competency frameworks used for physician education. In particular, tutors thought that tutoring gross anatomy had improved their knowledge of professional behavior as well as their communication skills. They also felt that they had learned to take on more responsibility and to use available resources more effectively. Overall, tutoring gross anatomy was perceived as very challenging, but also very rewarding, mainly because it provided the opportunity to develop and strengthen important skills such as self-confidence, selfawareness, positive thinking, self-insight, and stress management.”  Subheadings: 1) Quantitative Evaluation: Questionnaire Results, 2) Qualitative Evaluation: Personal Interviews, 2.1) doctors as medical experts, 2.2) doctors as scholars, 2.3) doctors as communicators, 2.4) doctors as managers, 2.5) doctors as team members, 2.6) doctors as professionals, 2.7) personal competencies |
| Alves-Costa, F., Hamilton-Giachritsis, C., Christie, H., & Halligan, S. L. (2018). Self-perception of adaptation among homicidally bereaved individuals following a psychoeducational intervention: a UK longitudinal qualitative study. BMJ Open, 8(8), e020443-e020443.  Country: UK | "Thus, this study aimed to contribute to the literature by gathering the individuals’ perceptions about how they adjust posthomicide longitudinally. Furthermore, it sought to understand their perspectives on benefits 1. What changes occurred over time? 2. What was their perception of the benefits of a residential intervention?" | mixed methods & qualitative longitudinal research | Population: participants of a psychoeducational intervention following traumatic experiences particularly homicidal bereavement for those affected by serious crime (n=14)  Data: Individual interviews  Length of data collection: Up to 5 years | “Three main themes and nine subthemes emerged, and applied to both groups, as follows: (1) actual changes perceived by the participants (increased understanding, improved coping strategies and positive self-change), (2) barriers to recovery (severe psychological difficulties over time, need for further support, reminders and close relationships with both victim and perpetrator), and finally perceived future progression (living day by day, hope and hopelessness). The only significant differences between the two groups related to the reported self-growth among LTG [long-term trajectory group] individuals and the perceived increased informal support among STG [short-term trajectory group] individuals by keeping in touch in other EV [Escaping Victimhood] participants.”  Subheadings: 1) Actual change (Increased understanding, Improved coping strategies, Positive self-change, Positive self-growth, Desire to help others, Role of the Escaping Victimhood programme), 2) Barriers to recovery (Ongoing emotional fluctuation and need for support, Reminders, Close relationhip to the victim and perpetrator), 3) Perceived future progression (Living day by day, Hope, Hopelessness) |
| Andersen, I. C., Thomsen, T. G., Bruun, P., Bødtger, U., & Hounsgaard, L. (2017a). The experience of being a participant in one's own care at discharge and at home, following a severe acute exacerbation in chronic obstructive pulmonary disease: a longitudinal study. International Journal of Qualitative Studies on Health and Well-Being, 12(1), 1371994-1371994.  Country: Denmark | "To explore COPD [chronic obstructive pulmonary disease] patients’ experiences of participating in their care in the transitional period around discharge from hospital and in their own subsequent day-to-day care at home following a severe AECOPD [acute exacerbation in chronic obstructive pulmonary disease]." | Phenomenology & qualitative longitudinal research | Population: Chronic obstructive pulmonary disease patients’ (n=15)  Data: Observations, individual interviews and joint interviews  Length of data collection: Up to 18 months (range 4 days to 18 months) | “Before discharge, the patients struggled to regain a sense of control in their efforts to build up strength, and acquire sufficient clarity and confidence to face self-management at home. At home, the patients strived to comply with advice and encouragement in a struggle to stay motivated and confident, and to ask for help.”  Subheadings: 1) Hospital phase: struggling to regain a sense of control (Building up strength, Seeking clarity and confidence), 2) Home phase: striving to comply with advice and encouragement to maintain health and well-being (Struggling to stay motivated and confident, Asking for help) |
| Andersen, I. C., Thomsen, T. G., Bruun, P., Bødtger, U., & Hounsgaard, L. (2017b). Patients' and their family members' experiences of participation in care following an acute exacerbation in chronic obstructive pulmonary disease: A phenomenological-hermeneutic study. Journal Of Clinical Nursing, 26(23), 4877-4889.  Country: Denmark | "The aim of this study was to explore COPD [chronic obstructive pulmonary disease] patients’ and their family members’ experiences of both participation in care during hospitalisation for an AECOPD[acute exacerbation in chronic obstructive pulmonary disease], and of the subsequent day-to-day care at home." | Phenomenology | Population: COPD patients’ and their family members’ (n=27)  Data: Observations, informal interviews, and individual interviews  Length of data collection: 11 to 18 months (range 4 days to 18 months) | “Participation in care was perceived as valuable, but could be associated with tensions and increased uncertainty. While patients mostly demonstrated a reactive approach to care, family members strived to be more proactive. In hospital, preparing for discharge included an effort to find a balance between powerlessness and influence during interactions with healthcare professionals. At home, managing further recovery and self-management were characterised by navigating between mutual pressure and consideration within the family.  Subheadings: 1) Hospital phase: Balancing between powerlessness and influence, 2) Home phase: Navigating between mutual pressure and consideration |
| Andersen, I. C., Thomsen, T. G., Bruun, P., Bødtger, U., & Hounsgaard, L. (2018). Between hope and hopelessness: COPD patients' and their family members' experiences of interacting with healthcare providers – a qualitative longitudinal study. Scandinavian Journal of Caring Sciences, 32(3), 1197-1206.  Country: Denmark | "…explore the meaning of COPD patients’ and their family members’ experiences of interacting with healthcare providers to their daily self-management over time." | Phenomenology & qualitative longitudinal research | Population: COPD patients and family members (n=17)  Data: Participant observations, individual and joint interviews  Length of data collection: Up to 18 months | ‘Between hope and hopelessness’ involved frustrations, concerns and doubts, all of which could relate to the interaction with healthcare providers. ‘Seeking support from healthcare services’, ‘navigating between healthcare providers’ and ‘collaborating with healthcare providers at home’ could entail opportunities to strengthen self-management and hope; however, it could also entail reduced faith in getting the right help and hopelessness.”  Subheadings: 1) Between hope and hopelessness (Seeking support from follow-up healthcare services, Navigating between healthcare providers, Collaborating with healthcare providers at home) |
| Armuand, G., Wettergren, L., Nilsson, J., Rodriguez-Wallberg, K., & Lampic, C. (2018). Threatened  fertility: A longitudinal study exploring experiences of fertility and having children after cancer treatment. European Journal of Cancer Care, 27(2), e12798-e12798.  Country: Sweden | "The aim of this study was, therefore, to explore how men and women experience the threat of infertility and their thoughts about having children after cancer during the first 2 years following diagnosis." | qualitative longitudinal research | Population: Couples with risk of infertility after cancer diagnosis (n=21 participants)  Data: Individual interviews  Length of data collection: 2 years | “The analysis resulted in the identification of four themes: Continue calmly on chosen path, Abandoning plans for children, Avoiding the subject of fertility and Struggling towards life goals.”  Subheadings: Descriptions of the participants, 1) Continue calmly on chosen path, 2) Abandoning plans for children, 3) Avoiding the subject of fertility, and 4) Struggling towards life goals |
| Arnolds, M., Xu, L., Hughes, P., McCoy, J., & Meadow, W. (2018). Worth a try? Describing the experiences of families during the course of care in the neonatal intensive care unit when the prognosis is poor. The Journal of Pediatrics, 196, 116-122.e113.  Country: US | "This study illuminates the experience of families during an infant’s admission as well as after discharge or death." | qualitative longitudinal research | Population: Parents to infants (n=52)  Data: Joint interviews  Length of data collection: Average of 435 days (range, 259-750 days) | “The most common themes identified included realism about death (24 families), appreciation for the infant’s care team (23 families), and optimism and hope (22 families). Overall themes were very similar across both centers, and among parents of infants who died and those who survived. Themes of regret, futility, distrust of care team, and infant pain were brought up infrequently or not at all.”  Subheadings: No headlines in resultsection. |
| Asada, Y., Gilmet, K., Welter, C., Massuda-Barnett, G., Kapadia, D. A., & Fagen, M. (2019). Applying theory of change to a structural change initiative: Evaluation of model communities in a diverse county. Health Education & Behavior, 46(3), 377-387.  Country: US | "...this article examines the application of a ToC framework [the theory of change] that guided planning, implementation, and evaluation of the Model Communities grant program (2011-2012) that was funded to suburban Cook County as one component of the CPPW [Communities Putting Prevention to Work] initiative." | Case study | Population: Community-based organization receiving funds to work for health behaviours (six data collection sites)  Data: Individual interviews  Length of data collection: Unclear | “Adaptations to the ToC included the addition of a construct, ‘change readiness,’ as well as refinements to constructs: organizational capacity (human capital, technical assistance, informal and formal leadership), local partnerships, and the importance of sustainability.”  Subheadings: 1) Readiness and change readiness: Before and after model communities, 2) Enhanced organizational capacity: Capacity building (Human capital, Technical assistance, Formal and informal leadership), 3) Alliance strengthening: Local partnerships, 4) Sustainability of structural change |
| Asamane, E. A., Greig, C. A., Aunger, J. A., & Thompson, J. L. (2019). Perceptions and factors influencing eating behaviours and physical function in community-dwelling ethnically diverse older adults: A longitudinal qualitative study. Nutrients, 11(6).  Country: UK | "The present study uses a longitudinal qualitative design to: (1) identify and compare factors influencing eating behaviours and physical function among ethnic older minorities living in Birmingham, United Kingdom; and (2) understand how these factors and their association with healthy eating and physical function changed over 8 months" | Phenomenology & qualitative longitudinal research | Population: Older people from ethnic minorities (n=92)  Data: Individual interviews  Length of data collection: 8 months | “Healthy eating was viewed as more important than, and unrelated to, physical function. Personal, social and cultural/environmental factors were identified as the main factors influencing eating behaviours and physical function, which differed by ethnicity, age, and sex. At 8-month interviews, more men than women reported adverse changes.”  Subheadings: 1) The differing perceptions of healthy eating and physical function, 2) The personal, social and cultural/environmental factors influencing eating behaviours and physical function and how these factors differ among the sample, 3) Perceived changes to eating behaviours and physical function over the 8-month follow-up period |
| Bagot, K. L., Moloczij, N., Barclay-Moss, K., Vu, M., Bladin, C. F., & Cadilhac, D. A. (first published 2018). Sustainable implementation of innovative, technology-based health care practices: A qualitative case study from stroke telemedicine. Journal of Telemedicine and Telecare, 2020, 26(1–2) 79–91.  Country: Australia | "The aim of this study was to identify factors to support the sustainability of an innovative technology-based programme beyond the initial implementation phases." | Case study | Population: Health-care professionals (n=25)  Data: Individual interviews  Length of data collection: 6 months | “New facilitators were identified including hospital system changes, benefits to clinicians and telemedicine becoming standard practice. New and ongoing barriers included infrequent use, competing demands and the continued resistance to a specific treatment.”  Subheadings: 1) Identification of barriers and facilitators for sustainability, 2) Telemedicine and thrombolysis (overarching theme), 3) Perceptions of telemedicine systems and technology, 4) Organisational and cultural environment, 5) Processes of clinical care, and 6) Benefits of utilisation |
| Balmer, D. F., Devlin, M. J., & Richards, B. F. (2017). Understanding the relation between medical students' collective and individual trajectories: An application of habitus. Perspectives on Medical Education, 6(1), 36-43.  Country: US | "Thus, we posed another research question in this secondary analysis, ‘How might the concept of habitus speak to the relation between collective trajectories and individual trajectories of medical students?’" | Case study | Population: Medical students (n=19)  Data: Individual interviews  Length of data collection: About 30 months | “The social space of undergraduate medical education harmonized students’ experience and helped explain the collective trajectory, as evidenced by students’ consistent reports of taking initiative and staying openminded. But individuals were not totally harmonized. They had unique dispositions that influenced their ability to access valued resources and shaped their behaviour. For example, Emily consistently spoke of being driven by her own goals; Zach focused on meeting expectations of au thorities; Hilary routinely oriented toward abstract medical knowledge.”  Subheadings: 1) Emily: achieving personal goals, 2) Zach: ‘Stepping up’ to expectations of authorities, 3) Hilary: gaining abstract medical knowledge |
| Baloh, J., Zhu, X., & Ward, M. M. (2018). Types of internal facilitation activities in hospitals implementing evidence-based interventions. Health Care Management Review, 43(3), 229-237.  Country: US | "To examine internal facilitation activities at ten critical access hospitals in rural Iowa during their implementation of TeamSTEPPS, a patient safety intervention, and to identify characteristics that distinguish different types of facilitation activities" (from abstract) | qualitative longitudinal research | Population: Health-care providers and managers at 10 hospitals (n=77 key informants)  Data: Individual interviews  Length of data collection: 2 years | “We identified four types of facilitation activities—Leadership, Buy-in, Customization, and Accountability. Individuals and teams engaged in different types of facilitation activities, both in a planned and an ad-hoc manner. These activities targeted at both people and practices, and exhibited varying temporal patterns (start and peak time).”  Subheadings: 1) Leadership, 2) Buy-in, 3) Customization, and 4) Accountability. In end of results interactions between the themes and temporality is described. |
| Baretta, D., Perski, O., & Steca, P. (2019). Exploring users' experiences of the uptake and adoption of physical activity apps: Longitudinal qualitative study. JMIR Mhealth And Uhealth, 7(2), e11636-e11636.  Country: Italy | "The aim of this study was to guide the selection of design features to implement in Physical Activity [PA] apps for nonclinical, adult populations. Through a combination of think-aloud methodology and in-depth interview techniques, this study examined (1) what features potential users expect to be important for engagement with PA apps during first exposure to never-used, randomly allocated, and commercially available PA app and (2) what features are judged to be important for supporting engagement and satisfactory experiences after 2 weeks’ usage of the same PA app." | Qualitative longitudinal research | Population: Adults using physical activity apps (n=20)  Data: Individual interviews and think aloud interviews  Length of data collection: 2 weeks | “Features that promote a fair and simple user experience, support users’ self-regulation skills, and address users’ exercise motives were considered important for engagement both during a first exposure and after a 2-week use of PA [Physical Activity] apps. Features that support users’ need for relatedness as well as those that facilitate users to implement their intentions were expected to be important for engagement mainly during a first exposure to PA apps. Proactive and tailored features that integrate behavioral, psychological, and contextual information to provide adaptive exercise plans and just-in-time support were considered relevant to sustain engagement over time.”  Subheadings: Descriptive statistics, Thematic analysis, 1) A fair and simple user experience (baseline, follow-up), 2) Features that promote a sense of autonomy and self-regulation of behavior (baseline, follow-up), 3) Efficient and reliable monitoring and feedback of physical activity (baseline, follow-up), 4) Features that address users’ exercise motives (baseline, follow-up), 5) Need for relatedness (baseline, follow-up), 6) Peer support (baseline, follow-up), 7) Coaching support (baseline, follow-up), 8) Social comparison (baseline, follow-up), 9) Tailored action planning (baseline, follow-up), 10) Proactive motivational features (baseline, follow-up) |
| Barros Ferreira, E., Oliveira de Almeida Marques da Cruz, F., Alves Costa de Jesus, C., Moura Pinho, D. L., Kamada, I., & Diniz dos Reis, P. E. (2017). Telephone contact as a strategy for the promotion of comfort to the patient submitted to chemotherapy. Journal of Nursing UFPE, 11(5), 1936-1942.  Country: Brazil | "This study aimed to monitor the adverse effects of antineoplastic chemotherapy in patients undergoing outpatient treatment and to describe the telephone follow-up as a strategy to provide comfort, according to the assumptions of Katherine Kolcaba." | not described | Population: Patients undergoind chemotherapy (n=21)  Data: Observations (recorded phone consultations)  Length of data collection: 7 weeks | “(…) data were collected on the main signs and symptoms reported by them: nausea, weakness, vomiting, inappetence, alopecia and decreased food intake.”  Subheadings: 1) Relief in the physical context, 2) Transcendence in the psycho-spiritual and physical contexts, 3) Tranquility in the physical, psychospiritual and sociocultural contexts |
| Barthel, S., Belton, S., Raymond, C. M., & Giusti, M. (2018). Fostering children's connection to nature through authentic situations: The case of saving salamanders at school. Frontiers in Psychology, 9, 928-928.  Country: Sweden | "The aim of this paper is to increase our understanding about if, how and by which means children’s affective relationships with nature change by taking part in a nature conservation project during school hours, and if such a shift persists 2 years post-participation. Does participating in the Salamander Project at school strengthen children’s connection to nature? If so, how do children learn to create affective relations with nature? Which specific situations might encourage or enable stronger affective relationships with nature? Do affective relations persist 2 years after the project?" | qualitative longitudinal research | Population: 10 year old children (n=57)  Data: Individual interviews, open-ended questions in a questionnaire, and field observations  Length of data collection: 2 years | “We found indications that children developed sympathy for salamanders and increased concern and care for nature, and that such relationships persisted 2 years after participation. Our rich qualitative data suggest that whole situations of sufficient unpredictability triggering free exploration of the area, direct sensory contact and significant experiences of interacting with a species were important for children’s development of affective relationships with the salamander species and with nature in an open-ended sense. Saving the lives of trapped animals enabled direct sensory interaction, feedback, increased understanding, and development of new skills for dynamically exploring further ways of saving species in an interactive process experienced as deeply meaningful, enjoyable and connecting. The behavioral setting instilled a sense of pride and commitment, and the high degree of responsibility given to the children while exploring the habitat during authentic situations enriched children’s enjoyment.”  Subheadings: 1) Participating in something of significance with a sense of responsibility, 2) The project provided authentic situations, 3) Fun and excitement, 4) self-reflection on methodological approach and results |
| Bélanger, M., Wolfe Phillips, E., O'Rielly, C., Mallet, B., Aubé, S., Doucet, M., Couturier, J., Mallet, M., Martin, J., Gaudet, C., Murphy, N. & Brunet, J. (2017). Longitudinal qualitative study describing family physicians' experiences with attempting to integrate physical activity prescriptions in their practice: 'It's not easy to change habits'. BMJ Open, 7(7), e017265-e017265.  Country: Canada | "We aimed to explore how their [*family physicians who were new recipients of physical activity prescription pads]* prescription habits changed (if at all) over time and to identify barriers and enablers to writing physical activity prescriptions for their patients." | Qualitative longitudinal research | Population: Family physicians (n=11)  Data: Individual interviews  Length of data collection: 12 months | “Initially, participants exhibited confidence in their ability to write PA [Physical Activity] prescriptions in the future and intended to write prescriptions. However, data from the follow-up interviews indicated that the rate of implementation was lower than anticipated by participants and prescriptions were not part of their regular practice. Two themes emerged as factors explaining the gap between their intentions and behaviours: (1) uncertainty about the effectiveness of written PA prescription, and (2) practical concerns (eg, changing well-established habits, time constraints, systemic institutional barriers).”  Subheadings: 1) Lack of conviction about effectiveness of physical activity prescription, and 2) Practical concerns |
| Bengtsson, M., Sjöblom, Y., & Öberg, P. (2018). Young care leavers' expectations of their future: A question of time horizon. Child & Family Social Work, 23(2), 188-195.  Country: Sweden | "The aim of this paper is to study young care leavers' own expectations of their future during their transition from care to independent adulthood. How, while still in care, do they express their expectations of this transition, and do these expectations change while they are taking their first step towards an independent life?" | qualitative longitudinal research | Population: Young people (n=15)  Data: Individual interviews  Length of data collection: 6 to 9 months | “The analysis using a general inductive approach showed that their expectations were dependent on the time horizon and that there was an obvious difference between the young informants' short‐ and long‐term expectations. Their short‐term expectations consisted of worries connected to their approaching discharge (at T1) and how to cope with challenges of everyday life after discharge from care (at T2).”  Subheading: 1) Short‐term expectations at Time 1, 2) Long‐term expectations at Time 1, 3) Short‐term expectations at Time 2, 4) Long‐term expectations at Time 2 |
| Bernays, S., Paparini, S., Seeley, J., & Rhodes, T. (2017). "Not taking it will just be like a sin": Young people living with HIV and the stigmatization of less-than-perfect adherence to antiretroviral therapy. Medical Anthropology, 36(5), 485-499.  Country: Uganda, US, UK, and Ireland | "To overcome the challenge of evaluating meaningfulness of treatment effect from a patient-reported perspective in the context of this rare cancer, a mixed methods approach that followed a convergent design was used [28]." | Qualitative longitudinal research | Population: Young people living with HIV (n=43)  Data: Individual interviews, field notes, observations  Length of data collection: Unclear, up to three interviews during a trial | “Drawing from Goffman’s notion of stigma, we analyze relational dynamics in HIV clinics, as rare spaces where HIV is ‘known,’ and how young people’s relationships may be threatened by non-adherence to treatment.”  Subheadings: 1) “A conversation from nowhere”: Silences and medicalization, 2) Moralized “ART talk” in the clinic, 3) The truth economy, 4) Viral monitoring, legitimate slippages, and “getting caught”, 5) Reflections on researching stories of non-adherence |
| Bernet, M., Sommerhalder, K., Mischke, C., Hahn, S., & Wyss, A. (2019). "Theory does not get you from bed to wheelchair": A qualitative study on patients' views of an education program in spinal cord injury rehabilitation. Rehabilitation Nursing, 44(5), 247-253.  Country: Switzerland | "The purpose of the current study was, therefore, to evaluate the new nurse-guided Patient Education [PE] program in Spinal Cord Injury rehabilitation, with a particular focus on the patients' perspectives and experiences. The study focused on two areas: (1) to analyze the program’s impact on patients’ preparation for their everyday life at home after rehabilitation and (2) to reveal which aspects of the PE program were most valuable from the participants’ perspective" | Qualitative longitudinal research | Population: Persons with spinal cord injury (n= 10)  Data: Individual interviews  Length of data collection: 5 to 6 months | “Patients emphasized the importance of the practical training of the education program. This impacted their well-being as well as their autonomy. They rated discussions with primary nurses and peers about physical or psychological concerns. However, after discharge, the learning process was ongoing, and patients experienced the transition to living at home as a major challenge.”  Subheadings: Participant description, 1) Acquisition of self-management competences, 2) Internal and external factors for developing daily life competences, 3) From competences to performance: transition to home |
| Beryl, L. L., Rendle, K. A. S., Halley, M. C., Gillespie, K. A., May, S. G., Glover, J., Yu, P., Chattopadhyay, R. & Frosch, D. L. (2017). Mapping the decision-making process for adjuvant endocrine therapy for breast cancer. Medical Decision Making, 37(1), 79-90.  Country: US | "To help fill this gap in understanding, we present data from a longitudinal, qualitative study of women undergoing breast cancer treatment. We pay particular attention to patterns of uncertainty and change in women’s decisions over time. We use detailed, qualitative data to characterize the multiple dimensions of the decision-making process and to consider how the chronic care decision-making process around hormone therapy differs from other acute decision making processes in breast cancer treatment. Our analysis suggests the importance of tracking ‘‘decisional resolve,’’ that is, a patient’s firm determination to maintain her decision to take or not take hormone therapy over time." | qualitative longitudinal research | Population: Women undergoing breast cancer treatment (n=41)  Data: Individual interviews  Length of data collection: Avarage 260 days | “Our data reveal that most patients do not make a single, discrete decision to take or not take hormone therapy but rather traverse multiple decisional states, characterized by 1) phase, 2) direction, and 3) strength of resolve. Our analysis tracks these decisional states longitudinally using a gray scale coded matrix. Our data show that decisional resolve wavers not just when considering therapy, as the existing concept of decisional conflict suggests, but even after initiating it, which may signal future decisions to for go therapy.”  Subheadings: Patient characteristics, 1) Multiple dimensions and states of hormone therapy decision making, 2) Variation and patterns across the decision-making process |
| Bharmal, M., Guillemin, I., Marrel, A., Arnould, B., Lambert, J., Hennessy, M., & Fofana, F. (2018). How to address the challenges of evaluating treatment benefits-risks in rare diseases? A convergent mixed methods approach applied within a Merkel cell carcinoma phase 2 clinical trial. Orphanet Journal of Rare Diseases, 13(1), 95-95.  Country: Germany | "To overcome the challenge of evaluating meaningfulness of treatment effect from a patient-reported perspective in the context of this rare cancer, a mixed methods approach that followed a convergent design was used [28]. In this study, qualitative interviews were performed with the patients participating in the JAVELIN Merkel 200 trial; in parallel, patients’ overall response by Independent Endpoint Review Committee (IERC) per Response Evaluation Criteria In Solid Tumors version 1.1 (RECIST) was determined clinically to report patients’ tumour response status [29]. Data from both the patient interviews and the clinical evaluations were then merged to look for correspondence between the qualitative outcomes data and the clinical and patient-reported quantitative outcomes data." | mixed methods | Population: Adults who had chemotherapy-refractory, with histologically confirmed Merkel cell carcinoma, rare skinn cancer (n=19)  Data: Individual interviews  Length of data collection: 25 weeks | “A high concordance between patient-reported qualitative data and assessed tumour response was observed. All eight patients who clinically improved had perceived a subjective improvement in their disease since the beginning of the study; the single patient whose disease worsened had a perceived deterioration. Patient perceived benefit in physical functioning, fatigue/energy and pain was subsequent to the measured change in clinical status as assessed by tumour response. This suggests that patient-reported assessment should be examined over the long term in order to optimally capture meaningful treatment effect.”  Subheadings: 1) Patient population, 2) Mixed methods research analysis: Qualitative findings in relation with quantitative findings, 2.1) Perceived change in cancer since starting study treatment, 2.2) Physical functioning, 2.3) Fatigue, 2.4) Pain |
| Bilodeau, K., Tremblay, D., & Durland, M.-J. (2019). Return to work after breast cancer treatments: Rebuilding everything despite feeling "in-between". European Journal of Oncology Nursing, 41, 165-172.  Country: Canada | "We propose targeting a turning point in the survival experience, i.e. the end of active cancer treatments. The goal of this study is therefore to describe the Return To Work journey of Breast cancer survivors from the end of active treatments through their return to work and job retention." | Interpretative Description method & qualitative longitudinal research | Population: Women after breast cancer treatment (n=9)  Data: Individual interviews  Length of data collection: Total data collection for the study were within 9 months. Unclear regarding the individual cases. | “The first six months after the end of treatment was identified as an ‘in-between’ period, during which participants questioned their ability to return to normal life due to the impact of side effects and a sense of withdrawal from health-care services. A three-stage journey similar to a rite of passage process (Van Gennep, 1969) was observed. 1) BC [breast cancer] survivors became aware of feeling on the fringes of the workplace as they awaited RTW [return to work]. 2) During that waiting period, BC survivors were rebuilding a “normal routine” and taking actions on their own in order to re-enter their workplace. 3) After RTW, they needed to make adjustments to maintain a work routine.”  Subheadings: 1) Stage 1: awareness of being “in between” health-care services and the workplace, 2) Stage 2: Waiting to go back to work, 3) Stage 3: Resuming work and adjusting |
| Birt, L., Poland, F., Charlesworth, G., Leung, P., & Higgs, P. (2019). Relational experiences of people seeking help and assessment for subjective cognitive concern and memory loss. Aging & Mental Health, 1-9.  Country: UK | "...understand the experiences of those who seek help from a primary care doctor for subjective cognitive concerns, but who do not receive a cognitive assessment. We also report the experiences of patients who have experienced cognitive assessments from diverse health providers, reporting processes and procedures which disrupt their trust in the process of cognitive assessment." | interpretive constructivist approach | Population: People experience cognitive concerns (n=41)  Data: Individual interviews  Length of data collection: 12 to 15 months | “Sample of 41 people (mean 75 years, 25 dementia diagnoses). Interpretative thematic analyses focused on the presence or absence of trust in relational experiences. There were three transition points where trust could be specifically developed or undermined: (1) deciding to seek help; (2) healthcare practitioners’ response to help-seeking; (3) process and outcome of assessment. Triggers for help-seeking for subjective cognitive concern were being prompted by family and knowing a relative with dementia. When participants perceived healthcare practitioners’ behaviour as dismissive, they had less trust in the outcome of the healthcare encounter. Misunderstandings and absence of trust in assessment processes led to participants stating they did not fully agree with the outcomes of the assessment.”  Subheadings: 1) Making a judgement about memory changes, 2) Professionals’ responses to participant help-seeking (Persistence – achieving an assessment, Dismissed with no assessment), 3) The assessment process (Trust and mistrust in assessment processes, Understanding the outcome of the assessment) |
| Blagden, N., & Wilson, K. (2019). "We're all the same here"- Investigating the rehabilitative climate of a re-rolled sexual offender prison: A qualitative longitudinal study. Sexual Abuse, 1-24.  Country: UK | "This study aims to explore the qualitative changes in the rehabilitative climate of a re-rolled prison (in this case, a general prison turned into a prison only for individuals who have sexually offended) from just after the re-roll to a year later. 1. To understand how individuals with sexual convictions experience a prison for only that client group, the challenges they face, and the opportunities to change and whether such experiences change over time. 2. To investigate the perspectives of prisoners on the purpose of the prison, its regime, and climate and whether this changes over time." | Qualitative longitudinal research | Population: Idividuals in prison for a sexual crime (n=10)  Data: Individual interviews  Length of data collection: 12 months | “Twenty interviews were conducted across the time points and revealed two main superordinate themes: ‘ ‘Being’ in a prison for individuals with sexual convictions’ and ‘obstructions to change.’ “  Subtitles: 1) Superordinate theme 1: “Being” in a prison for individuals with sexual convictions, 2) Subordinate theme 1: Facilitates “space” to change, 3) Subordinate theme 2: Constructive, meaningful, and reciprocal relationships, 4) Superordinate theme 2: Obstructions to change, 5) Subordinate theme 3: Relational ambivalence, 6) Subordinate theme 4: Deviant undercurrent, 8) Subordinate theme 5: Regime impediments |
| Bolier, M., Doulougeri, K., de Vries, J., & Helmich, E. (2018). 'You put up a certain attitude': a 6-year qualitative study of emotional socialisation. Medical Education, 52(10), 1041-1051.  Country: the Netherlands | "The aim of this longitudinal qualitative study was to gain a better understanding of the socialisation of emotion in the process of becoming a doctor." | qualitative longitudinal research | Population: Medical students (n=17)  Data: Individual interviews  Length of data collection: 6 years | “The socialisation of emotion in the process of becoming a doctor happens in a complex interplay between student and context. We identified two modes of emotional socialisation (e.g. explicit and implicit teaching about emotions), the latter including how the people observed by students express their emotions and how they respond to the emotions expressed by students. Although the main message conveyed to students still seemed one about hiding or suppressing emotion, we found that students were able to identify and build upon the emotional expression and responses they observed in positive role models and managed to create their own opportunities to express their emotions. We found large differences between students in how they perceived, presented and developed themselves.”  Subheadings: 1) Explicit and implicit teaching about emotion, 2) Emotional socialisation: The interplay between student and context |
| Bomsta, H., & Sullivan, C. M. (2018). IPV survivors’ perceptions of how a flexible funding housing intervention impacted their children. Journal of Family Violence, 33(6), 371-380.  Country: US | "The current study, then, presents IPV survivors’ perceptions of how a brief intervention designed to enhance their safety and housing stability also impacted their children’s well-being." | qualitative, longitudinal evaluation of a brief intervention | Population: Mothers experienced intimate partner violence (n=42)  Data: Individual interviews  Length of data collection: 6 months | “Mothers described improvements in children’s stability and safety, decreases in children’s stress levels, and improvements to their mood and behavior. They also discussed the symbiotic relationship between their own stress and well-being, and their children’s. The provision of flexible funding to assist domestic violence survivors with their housing also collaterally impacted their children’s safety, stress, mood and behavior.”  Subheadings: 1) Family housing outcomes at six-months after receiving flexible funding, 2) How flexible funding impacted children (The impact of housing stability on children, The impact of safety on children, Stress relief for mothers and children) |
| Boström, M., Ernsth Bravell, M., Björklund, A., & Sandberg, J. (2017). How older people perceive and experience sense of security when moving into and living in a nursing home: A case study. European Journal of Social Work, 20(5), 697-710.  Country: Sweden | "Therefore, a longitudinal design with in-depth interviews and observations was used in order to highlight sense of security by the older person when moving into and living in nursing home." | Case study | Population: Older persons (n=3)  Data: Individual interviews and observations  Length of data collection: 3 to 4 months | “The main theme, ‘Adaptation and sense of security’, indicates older persons’ need to adapt to the new context of the nursing home, and how this relates to their sense of security. The categories – ‘Control’, ‘Struggling for understanding’, ‘Lack of influence’, and ‘Grasping’ – suggest that older persons’ sense of security is reduced when they must adjust to routines without sufficient management and understanding. When able to maintain control over daily routines, and felt as a part of the new context, they perceived a sense of security.”  Subheadings: 1) The story of Frances (Control as sense of security, Struggling for understanding and sense of security, Influence and sense of security, Grasping for sense of security), 2) The story of Roland (Control as sense of security, Struggling for understanding and sense of security, Influence and sense of security, Grasping for sense of security), 3) The story of Hilma (Control as sense of security, Struggling for understanding and sense of security, Influence and sense of security, Grasping for sense of security) |
| Braaf, S., Ameratunga, S., Ponsford, J., Cameron, P., Collie, A., Harrison, J., Ekegren, C., Christie, N., Nunn, A., & Gabbe, B. (2019). Traumatic injury survivors' perceptions of their future: A longitudinal qualitative study. Disability and Rehabilitation, 1-11.  Country: Australia | "...this study aims to explore seriously injured adults’ perceptions of their future including their concerns, anxieties, coping mechanisms, and sources of resilience over time." | Qualitative longitudinal research | Population: Seriously injured adults (n=66)  Data: Individual interviews  Length of data collection: About 2 years | “Many traumatically injured people had persistent physical and mental impacts. Participants reported being anxious about pain, mobility, work, housing and accommodation, social activities, and finances in their future. Others were hopeful and optimistic regarding their future and developed coping strategies and adopted new viewpoints.”  Subheadings: 1) Future outlook (An uncertain future, Hopeful, Future viewpoint over time), 2) Concerns about and expectations of the future (Impact of ageing, Persistent pain and mental health issues, Feeling powerless to change or plan the future, Future losses and opportunities), 3) Ways of coping with concerns about the future (Living in the present, Preventive action, Looking for financial security, Redefining normal and the future, Seeking information) |
| Bradley, E. H., Brewster, A., L., McNatt, Z., Linnander, E. L., Cherlin, E., Fosburgh, H., Ting, H. H., Curry, L. A. (2018). How guiding coalitions promote positive culture change in hospitals: a longitudinal mixed methods interventional study. BMJ Quality & Safety, 27(3), 218-225.  Country: US | "Accordingly, we sought to understand what distinguished hospitals that succeeded to shift culture substantially and to reduce 30-day RSMR [risk-standardised mortality rate] after AMI [acute myocardial infarction] through participation in the LSL [Leadership Saves Lives] collaborative." | mixed methods | Population: Staff from multiple departments and professions (n=197)  Data: Questionnairs, individual interviews, and observations  Length of data collection: 18 months | “The six hospitals that experienced substantial culture change and greater reductions in RSMR demonstrated distinctions in: (1) effective inclusion of staff from different disciplines and levels in the organisational hierarchy in the team guiding improvement efforts (referred to as the ‘guiding coalition’ in each hospital); (2) authentic participation in the work of the guiding coalition; and (3) distinct patterns of managing conflict. Guiding coalition size and turnover were not associated with success (p values>0.05). In the six hospitals that experienced substantial positive culture change, staff indicated that the LSL learnings were already being applied to other improvement efforts.”  Subheadings: Overview, 1) Membership in the guiding coalition, 2) Participation by members, 3) Managing conflict, fatigue and engagement, 4) Views on future use of LSL learnings. |
| Brietzke, M., & Perreira, K. (2017). Stress and coping: Latino youth coming of age in a new latino destination. Journal of Adolescent Research, 32(4), 407-432.  Country: US | "Our study aimed to contextualize the processes of stress and coping among Latino adolescents growing up in an emerging Latino destination in the US—North Carolina (NC)." (from abstract) | Qualitative longitudinal research | Population: Latino adolescents (n=12)  Data: Individual interviews  Length of data collection: 3 years | “We identified four stress-coping trajectories that varied on the following dimensions: primary sources of stress, buffers countering these stressors, coping approaches and the effects of these processes on adolescents’ striving for socioeconomic mobility. Our findings underscore the interplay between family, school, and community environments within an emerging Latino destination.”  Subheadings: 1) The Protected Trajectory: “We help each other out…it’s basically the whole team”, 2) The Americanized Trajectory: “Her thoughts are about becoming American”, 3) The Resilient Trajectory: “We have to work now, to have the rewards in the future”, 4) The Consumed Trajectory: “If he [my dad] was involved with me, I think I would have kept going, but he never told me nothing” |
| Bright, F. A. S., Kayes, N. M., McPherson, K. M., & Worrall, L. E. (2018). Engaging people experiencing communication disability in stroke rehabilitation: A qualitative study. International Journal of Language & Communication Disorders, 53(5), 981-994.  Country: New Zealand | "The aim of this research was to develop rich understandings of the process of engagement for people experiencing communication disability after stroke and, in particular, to examine how rehabilitation practitioners worked to engage patients throughout rehabilitation." | underpinned by the Voice Centred Relational Approach | Population: People experiencing communication disability after stroke and their providers (n=31)  Data: Observations, short debrie interviews,and individual interviews  Length of data collection: 2 weeks | “Engagement was a relational practice on the part of the rehabilitation practitioner. It was underpinned by a relational philosophy and characterized by three core processes: embedding relational work throughout rehabilitation; getting to know the patient and working in ways valued by the patient; and communicating using relational dialogue and supported conversation. Practitioners wove these together with their technical, disciplinary-based work and rehabilitation tasks.”  Subheadings: 1) Valuing relationships and embedding relational work throughout interactions, 2) Getting to know the person: What matters and how to work with them? 3) Communicating to engage through supportive relational dialogue, 4) Enacting engagement as a relational practice: A summary |
| Brooks, H., Lovell, K., Bee, P., Fraser, C., Molloy, C., & Rogers, A. (2019). Implementing an intervention designed to enhance service user involvement in mental health care planning: A qualitative process evaluation. Social Psychiatry & Psychiatric Epidemiology, 54(2), 221-233.  Country: UK | "This manuscript reports on the nested qualitative process evaluation informed by implementation theory which aimed to explore the impact of the EQUIP training package to enhance user involvement in care planning." | qualitative process evaluation | Population: Service users, carers and mental health professionals (n=54)  Data: Individual interviews  Length of data collection: 12 months | “The process evaluation demonstrated that despite buy-in from those delivering care planning in mental health services, there was a failure of training to become embedded and normalised in local provision. This was due to a lack of organisational readiness to accept change combined with an underestimation and lack of investment in the amount and range of relational work required to successfully enact the intervention.”  Subheadings: 1) The sense and sense making of care planning training, 2) The absence of the required relational work to enact the principles of SDM, 3) The failure of organisational readiness to support the workability of the intervention, 4) Workability in context |
| Brown, D. (2019). Changes in communication tensions for men facing prostate cancer: A longitudinal study. Journal of Communication in Healthcare, 12(1), 44-53.  Country: New Zealand | "In an effort to better understand tensions over time, the principle aims of the present study are to establish if and how they change after men have undergone a prostate biopsy. Three research questions guided this study: RQ1: How were the communication tensions of a group of men waiting for the result of a prostate biopsy initially (T1) resolved three years later (T2)? RQ2: What new communication tensions emerged three years after the biopsy (T2)? RQ3: What competing discourses underpin the communication tensions in this cohort of men at T1 as compared to T2?" | not described | Population: Men undergone prostate biopsy (n=25)  Data: Individual interviews  Length of data collection: About 3 years | “The study found that the form of communication tensions changed from T1 to T2. Furthermore, it found that the tensions were both time- and context-specific. The communication tension at T2 were embedded in two competing discourses of health and wellbeing and of individualism.”  Subheadings: 1) RQ1: resolution of T1 tensions at T2, 2) RQ2: evolution of T1 tensions at T2, 3) RQ3: competing discourses at T2 |
| Busza, J., Dauya, E., Bandason, T., Simms, V., Chikwari, C. D., Makamba, M., Mchugh, G., Munyati, S., Chonzi, P., & Ferrand, R. A. (2018). The role of community health workers in improving HIV treatment outcomes in children: Lessons learned from the ZENITH trial in Zimbabwe. Health Policy and Planning, 33(3), 328-334.  Country: Zimbawe | "The aim was to give a voice to the central actors of a CHW [community health workers] programme that successfully met its goals, reflect on how use of a criteria-based framework during intervention design affected CHWs’ [community health workers] delivery, job satisfaction and motivation, and consider implications for the programme’s future scale-up and adoption in other settings." | not described | Population: Community health workers (n=19)  Data: Individual interviews  Length of data collection: 2 years | “CHWs [community health workers] expressed strong motivation, commitment and job satisfaction. They considered the intervention acceptable and feasible to deliver, and levels of satisfaction rose over interview rounds. Intensive supervision and mentoring emerged as critical to ensuring CHWs’ long-term satisfaction. Provision of job aids, standardized manuals and refresher training were also important, as were formalized links between clinics and CHWs. Concerns raised by CHWs included poor remuneration, their reluctance to stop providing support to individual families following the requisite number of home visits, and disappointment at the lack of programme sustainability following completion of the trial. Furthermore, intensive supervision and integration with clinical services may be difficult to replicate outside a trial setting.”  Subheadings: Selection and motivation, 1) Initial training, simple guidelines and standardized protocols, 2) Remuneration, career structure and supervision, 3) Issues around sustainability: political will, health systems and flexibility, 4) The role of people living with HIV, 5) Emphasis on retention and adherence, |
| Busza, J., Simms, V., Dziva Chikwari, C., Dauya, E., Bandason, T., Makamba, M., McHughc, G., & Ferrand, R. A. (2018). “It is not possible to go inside and have a discussion”: How fear of stigma affects delivery of community-based support for children’s HIV care. AIDS Care, 30(7), 903-909.  Country: Zimbawe | "We investigated how stigma affected a community-based intervention to support caregivers of children newly diagnosed with HIV in Harare, Zimbabwe. Specifically, we assessed children’s, caregivers’, and CHWs’ [community health workers] perceptions of how HIV-related stigma affected implementation." | qualitative longitudinal research | Population: Children with HIV, caregivers, andcommunity health workers (n=71)  Data: Individual interviews  Length of data collection: 18 months | “Children and caregivers described experiencing or witnessing stigma and discrimination, causing some to resist home visits by CHWs [community health workers]. Anxiety around stigma made it difficult for CHWs to promote key messages. In response, CHWs adapted the intervention by meeting caregivers outside the home, pretending to be friends or relatives, and proactively counteracting stigmatising beliefs. As members of local communities, some CHWs shared concerns about discrimination. HIV stigma can hinder “getting a foot over the threshold” in communitybased programmes, particularly for households most affected by discrimination and thus least likely to engage with services.”  Subheadings: 1) Anxiety around HIV discrimination, 2) Effects of stigma on participation in the intervention, 3) Responses by community health workers, |
| Cain, C. L., Frazer, M., & Kilaberia, T. R. (2019). Identity work within attempts to transform healthcare: Invisible team processes. Human Relations, 72(2), 370-396.  Country: US | "In this article, we ask: How do workers in a new healthcare team negotiate multiple identification targets, including their previous professional identities as well as their new team identities? What contextual events produce shifts in how workers identify? What do these shifts tell us about work identities, teams and organizational change?" | not described | Population: Workers in health-care teams    Data: Diaries  Length of data collection: 30 weeks | “We analyze 176 recordings over 30 weeks and find that: team members experience multiple identification targets more or less conflicting, depending on the organizational context; team members from different professional backgrounds experience identity processes differently; and conflicts with others affect how team members see themselves and one another.”  Subheadings: 1) Critical moment 1: Critique and stabilization, 2) Critical moment 2: Understanding the work and drawing boundaries |
| Cameron, A., Johnson, E. K., Lloyd, L., Evans, S., Smith, R., Porteus, J., Darton, R., & Atkinson, T. (2019). Using longitudinal qualitative research to explore extra care housing. International Journal of Qualitative Studies on Health and Well-Being, 14(1), 1593038-1593038.  Country: UK | "Using a longitudinal qualitative research (LQR) approach, the aim of The Provision of Social Care in Extra Care Housing (ECHO) project was to investigate how care is negotiated and delivered in ECH. Focusing on the “extra care” element of extra care housing services, the ECHO project explored the perspectives of residents on their changing care needs and their experiences of being cared for." | Qualitative longitudinal research | Population: Care staff and local commissioners of housing and care (n=51)  Data: Unstructured observations, individual interviews, and documents (e.g., annual reports)  Length of data collection: 20 months | “The study highlighted the complex ways in which some participants proactively managed the care and support they received, which we argue would have been difficult to discern through other methods.”  Subheadings: 1) Changing care needs, 2) The changing mix of residents, 3) Organisational changes |
| Campbell, V., & Nolan, M. (2019). 'It definitely made a difference': A grounded theory study of yoga for pregnancy and women's self-efficacy for labour. Midwifery, 68, 74-83.  Country: UK | "The aim was to generate a theory, grounded in women’s voices, about which aspects of Yoga for Pregnancy (YfP) are effective in enhancing women’s ability to manage labour." | Grounded theory | Population: Pregnant woman (n=22)  Data: Individual interviews  Length of data collection: Differed between partricipants, up to 7.5 months | “Analysis of interviews with women at three time points led to a propositional theory that yoga for pregnancy enhances women’s self-efficacy for labour by building their confidence and competence through a combination of techniques. These include repeated practice of a variety of pain management strategies, use of affirming language and the telling of positive labour stories, underpinned by yoga practice to lower somatic response to stress.”  Subheadings: 1) First interviews (Looking after myself and the baby, Hoping for a natural or easier labour, Preparing for something I can’t prepare for, Being calm and in control, Making friends), 2) Second interviews with the pregnant women - after attending YfP classes (Gaining confidence in managing labour, Practising techniques for labour, Learning from each other, Being in control, Preparing for something I can’t prepare for), 3) Postnatal interviews (Having a positive labour experience, Using techniques to manage labour, Being calm, confident and in control, Being positive and telling stories) |
| Carduff, E., Kendall, M., & Murray, S. A. (2018). Living and dying with metastatic bowel cancer: Serial in-depth interviews with patients. European Journal of Cancer Care, 27(1).  Country: UK | "This paper reports the longitudinal experiences, perceptions and service use of patients with metastatic colorectal cancer." | Narrative research | Population: Patients with metastatic colorectal cancer (n=16)  Data: Individual interviews  Length of data collection: One year | “Patients experience metastatic colorectal cancer in three phases; (1) Diagnosis and initial treatment; (2) Deterioration and social isolation and (3) Death and dying. Many patients initially said they hoped to survive, but, as ‘private’ and in- depth accounts of the experience emerged in further interviews, so did the understanding that this hope co- existed with the knowledge that death was near.”  Subheadings: Sample characteristics, 1) Theme 1: Diagnosis and initial treatment, 2) Theme 2: Deterioration, 3) Theme 3: Last months of life |
| Carusone, S. C., O'Leary, B., McWatt, S., Stewart, A., Craig, S., & Brennan, D. J. (2017). The lived experience of the hospital discharge "Plan": A longitudinal qualitative study of complex patients. Journal of Hospital Medicine, 12(1), 5-10.  Country: Canada | "A longitudinal case study approach was used, with multiple sources of data, to understand the clinical context and discharge plans in relation to the lived experience of patients over time, exploring potential misalignment and areas for improvement." | Case study & qualitative longitudinal research | Population: Complex patients (n=9)  Data: Individual interviews, medical chart abstraction, and review of discharge summaries.  Length of data collection: About 30 days | “Data were analyzed and reported in 4 key themes: 1) social support; 2) discharge process and transition experience; 3) post-discharge follow-up; and 4) patient priorities. After hospital discharge, the complexity of participants’ lives resulted in a change in priorities and subsequent divergence from the discharge plan. Despite the comprehensive discharge plans, with referrals designed to support their health and activities of daily living, participants experienced challenges with social support and referral uptake, resulting in a loss of stability achieved while in hospital.”  Subheadings: 1) Social support, 2) Discharge process and transition experience, 3) Follow-up and referrals, 4) Patient priorities |
| Castro, A., & Andrews, G. (2018). Nursing lives in the blogosphere: A thematic analysis of anonymous online nursing narratives. Journal of Advanced Nursing, 74(2), 329-338.  Country: Canada | "The purpose of this study was to explore the work-life narratives of nurses through a thematic analysis of the nursing accounts they post in their public blogs. The overall research question was, 'when nurses who work in traditional healthcare settings have the protection of online anonymity, how do they describe their nursing insights and experiences on blog platforms?' " | qualitative description | Population: Nurses (n=4)  Data: Blog texts  Length of data collection: 12 months | “Three major themes arose in these nurses’ online discussions of their work lives: they truly care about and value their nursing work, but they are feeling stressed and burnt out and they are using their anonymous blogs to share factors that frustrate them in their nursing work. Three main areas of frustration were revealed: teamwork problems, challenging patients and families, and management issues.”  Subheadings: 1) Nurses care about nursing work (Describing “real life” nursing tasks, Expressing pride in nursing, Describing caring about and advocating for patients, Describing sympathizing with patients), 2) Feeling stressed and burnt out, 3) Areas of frustration (Teamwork issues, Challenging patients and families, “Manglement” issues) |
| Cherry, M. G., Salmon, P., Byrne, A., Ullmer, H., Abbey, G., & Fisher, P. L. (2019). Qualitative evaluation of cancer survivors' experiences of metacognitive therapy: A new perspective on psychotherapy in cancer care. Frontiers in Psychology, 10, 949-949.  Country: UK | "In this study, we report the findings of qualitative research nested in an open trial of MCT [Metacognitive Therapy] for anxiety and depression in adult cancer survivors. We also interviewed consenting patients at each time point, to explore qualitatively: (i) how they understood and experienced the intervention; (ii) once treatment ended, how, and to what extent over the follow-up period, did patients transfer what they had learned across the range of emotional challenges arising during survivorship; and (iii) what characterized any patients who did not benefit?" | Qualitative longitudinal research | Population: Adult cancer survivors with depression or anxiety (n=19)  Data: Individual interviews  Length of data collection: 6 months | “Participants felt ‘overwhelmed’ by worry before starting MCT [Metacognitive Therapy] and doubted that such brief therapy could help. Their accounts focused on feeling ‘challenged’ to think differently by the psychologist. Those completing therapy were enthusiastic about it. They described having learned that thoughts are ‘only thoughts,’ that feelings of worry or sadness are a normal part of life, and that they were in control of whether and how they engaged with thoughts. Consequently, most described a sense of freedom to live free from worry. A minority described being unable to apply MCT to certain thoughts. Two patients who withdrew before completing MCT did not describe having learned what MCT was intended to achieve.”  Subheadings: Sample characteristics, 1) Participants Who Completed Therapy and Consistently Improved (The starting point: ‘caught in a spiral ofworry’, Doubt and challenge in learningMCT, A new relationship with thoughts: ‘they’re only thoughts’, The benefit oftherapy: ‘I can live my life now’), 2) Participants Who Did Not Complete Therapy or Did Not Consistently Improve |
| Choi, J., Lingler, J. H., Donahoe, M. P., Happ, M. B., Hoffman, L. A., & Tate, J. A. (2018). Home discharge following critical illness: A qualitative analysis of family caregiver experience. Heart & Lung, 47(4), 401-407.  Country: US | "The purpose of this study was to longitudinally describe the varying challenges and needs of family caregivers of ICU [intensive care units] survivors related to patients’ home discharge." | descriptive qualitative study | Population: Family caregivers of ICU survivors (n=20)  Data: Individual interviews  Length of data collection: 4 months | “Family caregivers (n = 20, all white, 80% woman) viewed home discharge as positive progress, but reported having insufficient time to transition from family visitor to the active caregiver role. Caregivers expressed feelings of relief during the steady recovery of family members’ physical and cognitive function. However, the slow pace of improvement conflicted with their expectations. Even after patients achieved independent physical function, emotional needs persisted and these issues contributed to caregivers’ anxiety, worry, and view that recovery was incomplete.”  Subheadings: Sample characteristics, 1) Patients’ care needs perceived by family caregivers, 2) Feeling overwhelmed and unprepared, 3) Feeling relieved and hopeful in the beginning but uncertain over time. 4) Staying vigilant, 5) Pacing expectation and negotiation, 5) Negative impact on caregivers’ health |
| Chu, H., Westbrook, R. A., Njue-Marendes, S., Giordano, T. P., & Dang, B. N. (2019). The psychology of the wait time experience - what clinics can do to manage the waiting experience for patients: a longitudinal, qualitative study. BMC Health Services Research, 19(1), 459-459.  Country: US | "Here in, we examine contextual factors and potential intervening variables that can shape the manner in which patients may respond to different waits. In addition, this study aims to identify actions providers and clinics can take to promote positive wait time experiences and mitigate negative ones." | Qualitative longitudinal research | Population: People with HIV (n=56)  Data: Individual interviews  Length of data collection: 6 to 12 months | “Our study showed that patients’ ‘willingness to wait’ is the product of the actual wait time, individual factors, such as the perceived value of the visit and cost of a long wait, and clinic and provider factors. Analyses revealed key steps providers and clinics can take to improve the wait time experience. These include: 1) proactively informing patients of delays, 2) explicitly apologizing for delays, and 3) providing opportunities for diversion. Patients noted the importance of these steps in curtailing frustrations that may result from a long wait.”  Subheadings: 1) Factors affecting the perception of wait time (Most patients expect to wait, Patients rationalize that “things happen” and tend to be forgiving, Patients weigh the cost of waiting in their willingness to wait, Patients who perceive greater value in a visit are willing to wait), 2) Key opportunities for making wait times less stressful and more tolerable (Informing patients of wait delays reduces uncertainty and increases tolerance, Apologizing for delays can mitigate negative emotions arising from a long wait, Create opportunities for patients to use wait time constructively) |
| Ciclitira, K., Starr, F., Payne, N., Clarke, L., & Marzano, L. (2017). A sanctuary of tranquillity in a ruptured world: Evaluating long-term counselling at a women’s community health centre. Feminism & Psychology, 27(4), 530-552.  Country: UK | "The main aim of this study was to explore how service users make sense of long term counselling in a women-only service in the context of their gendered experiences and complex needs." | not described | Population: Female service users (n=59)  Data: Individual interviews  Length of data collection: Unclear, before and after counseling | “Four main themes emerged: ‘violence and loss in the context of female oppression’, ‘a sanctuary for women’, ‘non-medicalised longterm counselling in a safe setting’, and ‘benefits of the long view’. Participants attributed various benefits to receiving long-term counselling in a women-only environment. These included gaining employment; reduced suicidal ideation, anxiety and depression; improved physical health, improved confidence and being able to make positive changes in their relationships. The women interviewed post-counselling valued long-term counselling in this context, in contrast to short-term therapy in a medicalised environment.”  Subheadings: 1) Violence and loss in the context of female oppression (The permeating losses of migration, Life blown apart: The impact of childhood abuse), 2) A sanctuary for women (An oasis of tranquillity, The gendered dimensions of mental distress, Non-medicalised long-term counselling in a safe setting), 3) Benefits of the long view (It takes time to do the work, Healing the ruptures: Psychological and physical wellbeing) |
| Clarke, G., Fistein, E., Holland, A., Tobin, J., Barclay, S., & Barclay, S. (2018). Planning for an uncertain future in progressive neurological disease: a qualitative study of patient and family decision-making with a focus on eating and drinking. BMC Neurology, 18(1), 115-115.  Country: UK | "We therefore undertook a study of patients and their families with a range of progressive neurological diseases. We investigated their experiences and views on decision-making concerning their care as their disease progressed, with a focus on problems with eating and drinking. The key research question was: How do patients and their family members make decisions about their future care, with a particular focus on mealtimes, eating and drinking?" | Qualitative longitudinal research | Population: Patients and their families with a range of progressive neurological diseases (n=29)  Data: Individual interviews, observation and informsal conversation  Length of data collection: 3 to 12 months depending upon disease | “Twenty-nine participants were interviewed between 2015 and 2017. Two key themes emerged from the analysis: 1) Health Literacy: the extent to which patients and relatives appeared to know about the condition and its treatment. Patients and their family members varied in their ability to speak and communicate about their condition and prognosis. 2) Planning style: the extent to which participants appeared to value involvement in advance care-planning. Patients and their family members varied in the way in which they made decisions: some preferred to ‘take each day as it comes’, while others wished to plan extensively for the future.”  Subheadings: 1) Theme one: Health literacy (Experts by experience, Well informed from the outset, Mixed picture, Still unaware), 2) Theme two: Planning style (‘Advance planning’, ‘Take each day as it comes’, Mixed strategies, No decision to be made) |
| Clermont, A., Kodish, S. R., Matar Seck, A., Salifou, A., Rosen, J., Grais, R. F., & Isanaka, S. (2018). Acceptability and utilization of three nutritional supplements during pregnancy: Findings from a longitudinal, mixed-methods study in Niger. Nutrients, 10(8).  Country: Niger | "The objective of this paper is to examine the factors influencing acceptability and utilization of these three supplements among a rural population in southern Niger using a longitudinal, mixed-methods design. In this paper, we discuss the typical consumption, perceived benefits, facilitating factors, and barriers to appropriate utilization reported by participants for each of the three supplement types over the course of pregnancy. We also examine household and community member perceptions of supplement utilization and triangulate qualitative findings with quantitative household utilization data from unannounced spot checks." | mixed methods | Population: Pregnant woman, household members, and community members (n>100)  Data: Individual interview and focus group interviews  Length of data collection: 6 months | “Participants accepted all three supplement types, and perceived a wide range of health benefits attributed to supplement consumption. However, several important barriers to appropriate consumption were reported, and rumors about the supplements leading to childbirth complications also decreased utilization. The household spot checks suggested that IFA [iron and folic acid supplements] had the highest level of correct consumption. Overall, despite a stated high level of acceptance and enthusiasm for the supplements among participants and their household members, certain fears, side effects, and organoleptic factors led to decreased utilization.”  Subheadings: 1) Typical Utilization, 2) Perceived Benefits, 3) Facilitating Factors, 4) Acceptability-Related Barriers, 5) Utilization-Related Barriers, 6) Family, Community, and Health StaffPerceptions, 7) Change over the Course ofPregnancy (Longitudinal Analysis), 8) Household Spot Checks |
| Coombe, J., Harris, M. L., & Loxton, D. (2019). Motivators of contraceptive method change and implications for long-acting reversible contraception (non-)use: A qualitative free-text analysis. Sexual & Reproductive Healthcare, 19, 71-77.  Country: Australia | "...this analysis sought to develop a greater understanding of the motivators of method change over time for young Australian women using three waves of survey data from the Contraceptive Use, Pregnancy Intention and Decisions (CUPID) Study. Using responses to an open-ended question regarding reasons for contraceptive change, we were particularly interested in exploring what these comments could tell us (if anything) about long-acting reversible contraception (LARC; IUDs, contraceptive implants, contraceptive injections), (non-)use among young women." | not described | Population: Young women (n=512)  Data: Free-text comments from questionnairs  Length of data collection: About 12 months | “512 women reported making at least one contraceptive method change, with 740 comments explaining these changes between them. Participants reported a multitude of reasons motivating their contraceptive change. Five key themes were developed to explain these motivators: the natural, sexual and fertile body, specific contraceptive characteristics and other important people. Findings suggest that women’s decisions to switch or discontinue a contraceptive depended largely on her ability (and desire) to juggle its impact on her sexual, fertile and natural body. Importantly, the transient and fluid nature of contraceptive practices were demonstrated, as the women adjusted their method to suit their needs at the time.”  Subheadings: 1) Initial analysis: motivators of method change, 2) The natural body, 3) The sexual body, 4) The fertile body, 5) Specific contraceptive characteristics, 6) Other important people, 7) Secondary analysis: motivators of contraceptive change over time, 8) Juggling the natural, sexual and fertile bodies, 9) Fertility intention and pregnancy, |
| Coombs, M. A., Parker, R., & de Vries, K. (2017). Managing risk during care transitions when approaching end of life: A qualitative study of patients’ and health care professionals’ decision making. Palliative Medicine, 31(7), 617-624.  Country: New Zealand | "To describe decision-making processes that influence transitions in care when approaching the end of life." | Qualitative longitudinal research | Population: Patients in palliative care who approache end of life, and their family members (n=29)  Data: Field observation and individual interviews.  Length of data collection: 3 to 4 months | “Managing risk was an important factor that influenced transitions in care. Patients and health care staff held different perspectives on how such risks were managed. At home, patients tolerated increasing risk and used specific support measures to manage often escalating health and social problems. In contrast, decisions about discharge in hospital were driven by hospital staff who were risk-adverse. Availability of community and carer services supported risk management while a perceived need for early discharge decision making in hospital and making ‘safe’ discharge options informed hospital discharge decisions.”  Subheadings: 1) How patients managed risk in decision making about transitions in care, 2) How HCPs managed risk in decision making about transitions in care |
| Corepal, R., Best, P., O'Neill, R., Tully, M. A., Edwards, M., Jago, R., Miller, S.J., Kee, F., Hunter, R. F. (2018). Exploring the use of a gamified intervention for encouraging physical activity in adolescents: a qualitative longitudinal study in Northern Ireland. BMJ Open, 8(4), e019663-e019663.  Country: UK (northern Ireland) | "The aim of this study was to explore the views and experiences of adolescents who participated in a gamified PA [physical activity] intervention based on Self-determination Theory (SDT), and the temporal changes of these views and experiences over the 1-year study period. Study objectives included: 1. To explore key aspects of a gamified PA intervention over a 1-year period using a qualitative longitudinal research (QLR) method. 2. To discuss key issues relating to the intervention, such as PA opportunities/barriers, the value of competition and types of rewards and so on. 3. To explore the key influences of PA and to determine who benefited from the intervention, how and why it worked for them. 4. To qualitatively chart changes in behaviours, opinions or views as a result of participating in the intervention." | qualitative longitudinal research | Population: Adolescents who participated in a gamified PA intervention (n=19)  Data: Focus group interviews  Length of data collection: 12 months | “Three core themes were identified: (1) competition; (2) incentives and (3) influence of friends. Participants indicated that a pedometer competition may help initiate physical activity but suggested that there were a number of barriers such as participants finding it ‘boring’, and feeling as though they had a remote chance of ‘winning’. ‘Incentives’ were viewed favourably, although there were participants who found not winning a prize ‘annoying’. Friends were a motivator to be more physically active, particularly for girls who felt encouraged to walk more when with a friend.”  Subheadings: 1) Theme 1: Competition (Subtheme A: Usefulness of competition for PA behaviour change, Subtheme B: Perceptions of the usefulness of self-competition for PA behaviour change, Subtheme C: Experiences of the team and individual competition) 2) Theme 2: Incentives (Subtheme A: Type of Incentive, Subtheme B: Perceptions of usefulness of incentives) 3) Theme 3: Influence of friends (Subtheme A: Role of friends in general for encouraging PA behaviour, Subtheme B: Role of friends in team competition) |
| Côté-Arsenault, D., & Denney-Koelsch, E. (2018). “Love is a choice”: Couple responses to continuing pregnancy with a lethal fetal diagnosis. Illness, Crisis & Loss, 26(1), 5-22.  Country: US | "Hence, the purpose of this study is to describe pregnant couples’ responses and relationships during pregnancy and after birth when they choose to continue a pregnancy with an LFD [Lethal Fetal Diagnosis]. A secondary goal is to examine similarities and differences in these responses within the couple." | longitudinal naturalistic study | Population: Pregnant couples (n=30 participants)  Data: Individual interviews and joint interviews  Length of data collection: 90 interviews were conducted between 2012 and 2014; each parent participated in one to five interviews | “Three categories emerged (Pregnant vs. Not Pregnant; Individual Responses to Adversity; Strength of the Couple Relationship) with 12 themes. Findings indicate that individual responses to these stressful pregnancies were inherent in who was physically pregnant, choosing whether to love and embrace the unborn baby, personal characteristics, and the strength of the couple relationship.”  Subheadings: 1) Overall impression, 2) Pregnant versus not pregnant (Physical experience, Choice, Grief, Prenatal attachment, Roles), 2) Individual responses to adversity (Maintaining normalcy, Recognizing reality, Relying on faith and hope), 3) Strength of couple relationship (Commitment to relationship, Shared decision-making, Mutual support), 4) Father’s experience of the study |
| Cresswell, K. M., Mozaffar, H., Lee, L., Williams, R., & Sheikh, A. (2017). Safety risks associated with the lack of integration and interfacing of hospital health information technologies: A qualitative study of hospital electronic prescribing systems in England. BMJ Quality & Safety, 26(7), 530-541.  Country: UK | "As part of an English programme of research, we explored the social and technical challenges relating to integration and interfacing experienced by early adopter hospitals of standalone and hospital-wide multimodular integrated electronic prescribing (ePrescribing) systems." (from abstract) | longitudinal, qualitative, multisite case study | Population: Health-care providers, managers, policy-makers for example users, implementers and software suppliers  Data: Individual interviews, documentary data, observations and expert roundtable discussions  Length of data collection: Up to 3 years (differed between sites) | “We observed that integration and interfacing problems obstructed effective information transfer in both standalone and multimodular systems, resulting in threats to patient safety emerging from the lack of availability of timely information and duplicate data entry. Interfacing problems were immediately evident in some standalone systems where users had to cope with multiple log-ins, and this did not attenuate over time. Multimodular systems appeared at first sight to obviate such problems. However, with these systems, there was a perceived lack of data coherence across modules resulting in challenges in presenting a comprehensive overview of the patient record, this possibly resulting from the piecemeal implementation of modules with different functionalities. Although it was possible to access data from some primary care systems, we found poor two-way transfer of data between hospitals and primary care necessitating workarounds, which in turn led to the opportunity for new errors associated with duplicate and manual information transfer. Extending ePrescribing to include modules with other clinically important information needed to support care was still an aspiration in most sites, although some advanced multimodular systems had begun implementing this functionality. Multimodular systems were, however, seen as being difficult to interface with external systems.”  Subheadings: 1) Lack of effective information transfer and associated safety risks (Lack of effective information transfer, Patient safety risks resulting from the lack of effective information transfer), 2) Approaches to improve quality and safety by promoting integration of information (Promoting presentation integration through effective interfacing, Incorporation of other clinically important information with ePrescribing), 3) Trade-offs between integration and interfacing strategies. |
| Curry, L. A., Brault, M. A., Linnander, E. L., McNatt, Z., Brewster, A. L., Cherlin, E., Peterson Flieger, S., Ting, H. H. & Bradley, E. H. (2018). Influencing organisational culture to improve hospital performance in care of patients with acute myocardial infarction: A mixed-methods intervention study. BMJ Quality & Safety, 27(3), 207-217.  Country: US | "We designed a 2-year intervention, Leadership Saves Lives (LSL),26 directed at fostering changes in hospital organisational culture that might contribute to reductions in RSMRs [risk-standardised mortality rates] for patients with AMI [acute myocardial infarction]. Our study addresses limitations of prior research through a longitudinal design in a diverse sample of hospitals, the use of robust quantitative and qualitative measures of culture, and the inclusion of an important clinical outcome." | mixed methods | Population: Participants from different departments and professions (n=197)  Data: Questionnairs, individual interviews, and ethnographic observations  Length of data collection: 18 months | “We observed significant changes (p<0.05) in culture between baseline and 24 months in the full sample, particularly in learning environment (p<0.001) and senior management support (p<0.001). Qualitative data indicated substantial shifts in these domains as well as psychological safety. Six of the 10 hospitals achieved substantial improvements in culture, and four made less progress. The use of evidence-based strategies also increased significantly (per hospital average of 2.4 strategies at baseline to 3.9 strategies at 24 months; p<0.05). The six hospitals that demonstrated substantial shifts in culture also experienced significantly greater reductions in RSMR than the four hospitals that did not shift culture (reduced RSMR by 1.07 percentage points vs 0.23 percentage points; p=0.03) between 2011–2014 and 2012–2015.”  Subheadings: Study hospitals and respondents, Quantitative results, Qualitative results, 1) Changes in learning environment, 2) Changes in senior management support, 3) Changes in psychological safety. |
| Daker-White, G., Hays, R., Blakeman, T., Croke, S., Brown, B., Esmail, A., & Bower, P. (2018). Safety work and risk management as burdens of treatment in primary care: Insights from a focused ethnographic study of patients with multimorbidity. BMC Family Practice, 19, 155.  Country: UK | "The aims of the study were to describe the safety issues identified in a cohort of primary care patients with multimorbidity; and, to explore the clinical and social context in which patient safety concerns arise and play out. These objectives were addressed via a longitudinal, multi-method qualitative study in the form of a focused ethnography [44]. The principle aim of the analysis was to construct a line of argument concerning the circumstances under which patient agency in safety monitoring might be bolstered." | longitudinal multimethod qualitative study, ethnography & case study | Population: Primary care patients with multimorbidity, spouses, health-care providers (n=6)  Data: Individual interviews, observations, and health care diaries  Length of data collection: 24 months | “Twenty-six patients were recruited. Events which could lead to harm were found in all areas of a framework based on published literature. ‘Under’ and ‘over’ consultation as a precursor of safety failures emerged through thematic analysis of observation and interview material. Other findings concerned workload (for doctors and patients) and the limitations of short consultation times. There were differences in health data collected directly from the patients versus that found in EHRs. Examples included reference to a stroke history and diagnoses for CKD and hypertension. Case study analysis revealed specific issues which appeared contextual to safety concerns, mostly around the management of polypharmacy and patient medication adherence. Clinical imperatives appear around risk management, but the study findings point to a potential conflict with patient expectations around investigation, diagnosis and treatment.”  Subheadings: 1) Results I – Health records analysis, 2) Results II - Case studies (Kathleen, Pamela, Deborah, Alan, Helen, Victoria) |
| Dambha-Miller, H., Silarova, B., Irving, G., Kinmonth, A. L., & Griffin, S. J. (2018). Patients' views on interactions with practitioners for type 2 diabetes: A longitudinal qualitative study in primary care over 10 years. The British Journal Of General Practice, 68(666), e36-e43.  Country: UK | "This study aims to explore patient views on factors within patient–practitioner interactions that are of significance to them after diagnosis, and over a 10-year experience of living with the disease." | qualitative longitudinal research | Population: Patients with diabetes type 2 (n=311)  Data: Open ended question in questionnairs  Length of data collection: 10 years | “At the 1-year follow-up, 311 out of 1106 (28%) participants had commented; 101 out of 380 (27%) participants commented at 10-year follow-up; and 46 participants commented at both times. Comments on preferences for faceto-face contact, more time with practitioners, and relational continuity of care were more common over time.”  Subheadings: 1) Face-to-face contact with practitioner, 2) Length of patient–practitioner interaction, 3) Continuity of care |
| Dang, B. N., Westbrook, R. A., Njue, S. M., & Giordano, T. P. (2017). Building trust and rapport early in the new doctor-patient relationship: A longitudinal qualitative study. BMC Medical Education, 17(1), 32-32.  Country: US | "...this study aims to identify what patients see as the most critical elements for building trust and rapport from the outset." | Qualitative longitudinal research | Population: People with HIV (n=21)  Data: Individual interviews  Length of data collection: 6 to 12 months | “Patients described significant anxiety and vulnerability not just from HIV itself, but also in starting a relationship as a new patient to a new provider. Our analysis of these experiences revealed five actions providers can take to reduce their patients’ anxiety and build trust early in the first visit: 1) provide reassurance to patients, 2) tell patients it’s okay to ask questions, 3) show patients their lab results and explain what they mean, 4) avoid language and behaviors that are judgmental of patients, and 5) ask patients what they want [i.e., treatment goals and preferences].”  Subheadings: 1) Patients assume the provider is knowledgeable; what they hope for is a provider who genuinely cares, 2) Patients experience a lot of anxiety and vulnerability as a new patient to a new provider, 3) First impressions matter and continuity of care is important to patients, 4) Actionable things providers can do to build trust and rapport (Patients want their providers to provide reassurance, Patients feel anxious asking their providers questions; they want their providers to tell them it’s okay to ask questions, Patients want to see their lab results and for the doctor to explain what they mean, Patients do not want to feel judged by their providers, Patients want to be participants in medical decisionmaking; they want providers to ask them what they want [i.e., treatment goals and preferences]) |
| Danneris, S. (2018). Ready to work (yet)? Unemployment trajectories among vulnerable welfare recipients. Qualitative Social Work, 17(3), 355-372.  Country: Denmark | "A central aim of the article is to introduce the client perspective as a way of increasing and supplementing our knowledge about policy delivery in terms of understanding not only what services and benefits clients receive but also how they are received and the consequences of receiving them (Borghi and Van Berkel, 2007; Larsen, 2013). Investigating the fabric and textures of the perceived policy implications and their longitudinal interrelations from the client perspective will illuminate corners of the concept of employability where the current map ceases and reveal new, perhaps conflicting, understandings and dilemmas (Verd and Lo´ pez, 2011: 3)." | qualitative longitudinal research | Population: Vulnerable welfare recipients (n=36)  Data: Individual interviews, observations, and informal conversations  Length of data collection: 24 months | “The article depicts four main stages – clients step into and out of through time, dependent on their perception of individual agency, institutional role and orientation towards the future. The results show that stages in vulnerable clients’ unemployment trajectories overlap in complicated ways, in which different explanations, effects, unintended consequences and interlocking problems are closely intertwined.”  Subheadings: 1) Analytical frame: Identifying transitions along the trajectory, 2) Analysis: Stages in unemployment trajectories, 3) Deterioration – Moving backward, 4) Progression – Moving forward, 5) Stagnation – Standing still, 6) Derailment – Off track |
| Dattalo, M., Wise, M., Ford Li, J. H., Abramson, B., & Mahoney, J. (2017). Essential resources for implementation and sustainability of evidence-based health promotion programs: A mixed methods multi-site case study. Journal of Community Health, 42(2), 358-368.  Country: US | "This study examines the varying abilities of intervention sites from the randomized trial to implement and sustain SO [Stepping On is a falls prevention program] and CDSMP [Chronic Disease Self-Management Program] workshop delivery over 3 years and compares different approaches to preparing for workshop implementation by level of sustainability. | mixed methods and case study | Population: Coaches and country change leaders (n=9)  Data: Individual interviews, documents (e.g., annual county reports), and open-ended questions in questionnairs  Length of data collection: 3 years | “Readiness to implement evidence-based programs as low at baseline as all site leaders described needing to secure additional resources for program implementation. Sites that successfully utilized six essential resources implemented and sustained greater numbers of workshops: (1) External Partnerships, (2) Agency Leadership Commitment, (3) Ongoing Source of Workshop Leaders, (4) Health Promotion Coordination Tasks Assigned to Specific Staff, (5) Organizational Stability, and (6) Change Team Engagement.”  Subheadings: 1) External partnerships, 2) Agency leadership commitment, 3) Ongoing source of workshop leaders, 4) Health promotion coordination tasks assigned to specific staff, 5) Organizational stability, 6) Change team engagement |
| Davies, K. E., Marshall, J., Brown, L. J. E., & Goldbart, J. (2017). Co-working: Parents’ conception of roles in supporting their children’s speech and language development. Child Language Teaching & Therapy, 33(2), 171-185.  Country: UK | "The current study aimed to identify parents’ conception of roles and to track changes in conception as they participated in typical speech and language therapy intervention provided by services in the UK. The study posed the following research questions: 1. What are parents’ conceptions of their own and SLTs’ [speech and language therapists'] roles in speech and language therapy intervention for their pre-school children? 2. How do parents’ conceptions of these roles change during speech and language therapy intervention?" | Qualitative longitudinal research | Population: Parents (n=14)  Data: Individual interviews  Length of data collection: 30 weeks | “Parents had a firm conception of their role as advocates but did not express a clear notion of their role as intervener before involvement with the SLT. During intervention, some described changing their conception of role including adopting roles as ‘implementer’ and ‘adaptor’ of intervention. In some cases, parents described changes in their approach to parenting which they associated with adopting more active roles as interveners. Policy statements about parental choice and co-working typically present parents’ conception of roles as fixed”  Subheadings: 1) Parents’ conception of their role during involvement in speech and language therapy (Advocate role, Intervener role, Taking responsibility), 2) Parents’ conception of SLTs’ role during intervention (SLT as assessor, SLT as intervener, SLT as intervention planner, SLT as teacher, 3) Changes in parents’ conception of their own role during intervention (Conception of role remains unchanged, Gaining a conception of role as an ‘implementer’, Gaining a conception of role as an ‘intervener’). |
| Davies, S., Salmon, P., & Young, B. (2017). When trust is threatened: Qualitative study of parents' perspectives on problematic clinical relationships in child cancer care. Psycho-Oncology, 26(9), 1301-1306.  Country: UK | "So we took an inductive approach, analysing qualitative interviews with parents who described pervasive difficulties in their relationships with clinicians to understand their needs and identify ways of helping them." | Qualitative longitudinal research | Population: Parents who described pervasive difficulties in their relationships with clinicians in childhood cancer (n=20)  Data: Individual interviews  Length of data collection: 12 months | “All 20 parents described problems with clinical care such as inadequate information or mistakes by staff but varied in how much the problems threatened their sense of relationship with clinicians. Some parents saw the problems as having no relevance to the parent‐clinician relationship. Others saw the problems as threats to the clinical relationship but worked to ‘contain’ the threat in ways that preserved a trusting relationship with at least one senior clinician. Parents' containment work protected the security they needed from the parent‐clinician relationship, but containment was a tenuous process for some. A few parents were unable to contain the problems at all; lacking trust in clinicians, these parents suffered considerably”  Subheadings: Participant characteristics, 1) Parents in the threatened relationship group (All described problems related to clinical care, clinical interactions, or care “systems”, Parents described problems as threats to their relationships with clinicians), 2) Parents in the comparison group also experienced problems with clinical care, but these did not threaten clinical relationships, |
| Davis, E. B., Kimball, C. N., Aten, J. D., Andrews, B., Van Tongeren, D. R., Hook, J. N., Davis, D. E., Granqvist, P., & Park, C. L. (2019). Religious meaning making and attachment in a disaster context: A longitudinal qualitative study of flood survivors. Journal of Positive Psychology, 14(5), 659-671.  Country: US | "In the current study, we develop a grounded theory of how people draw on their religion and their religious attachment (perceived relationship with God) to make meaning of their disaster experiences. More specifically, we employed a longitudinal qualitative design to examine how post-disaster religious meaning making and attachment unfold over time and to compare these processes in the acute and intermediate wake of a disaster (e.g. at 4 weeks [Time 1, T1] and 6 months post-disaster [Time 2, T2]) The specific research questions we sought to explore were: (1) How do disaster survivors engage in religious meaning making after a disaster? (2) What role does religious attachment play in this process of religious meaning making? (3) Does this religious meaning making process differ across disaster phases?" | Grounded theory | Population: Disaster survivors (n=36)  Data: Individual interviews  Length of data collection: 6 months | “At both timepoints, results revealed that survivors who were theistic believers engaged in postdisaster religious meaning making in which they drew on benevolent God representations and theodicies to appraise the disaster’s cause, purpose, and religious-attachment effects, thereby contributing to positive religious-attachment outcomes (religious meanings made; e.g. renewed beliefs and experiences of God’s benevolence and providence).”  Subheadings: 1) Grounded theory of religious meaning making in a disaster context, 2) Predisaster religious attachment and global religious meaning, 3) Postdisaster religious meaning making (Benevolent God representations and theodicies), 3) Benevolent God representations and theodicies, (Cause, Purpose, Religious-attachment effects), 4) Religious meanings made |
| Davis, E. B., Kimball, C. N., Aten, J. D., Hamilton, C., Andrews, B., Lemke, A., Hook, J. R., Captari, L., Granqvist, P., Hook, J. N., Davis, D. E., Van Tongeren, D. R., Cattrell, E. L., Cuthbert, A. D., Chung, J. (2019). Faith in the wake of disaster: A longitudinal qualitative study of religious attachment following a catastrophic flood. Psychological Trauma: Theory, Research, Practice & Policy, 11(6), 578-587.  Country: US | "...we anticipated this natural disaster would have some type of effect on survivors’ religious attachment (i.e., perceived relationship with God). We chose a longitudinal design in order to permit an examination of these effects over time and a comparison of such effects in the acute versus intermediate wake of the disaster. Research Question 1: In the wake of a disaster, what types of religious-attachment language do theistic survivors use to describe their pre-disaster and post-disaster religious attachment? Research Question 2: After a disaster, what types of differences are there in the religious-attachment language that theistic disaster survivors use to describe their religious attachment, based on the type of disaster exposure they have experienced (e.g., direct vs. indirect exposure) or based on their previous exposure to a catastrophic natural disaster? Research Question 3: How do theistic survivors describe the quality and characteristics of their post-disaster religious attachment (perceived relationship with God)? What are the themes that emerge as survivors discuss various aspects of their post-disaster religious attachment?" | Qualitative longitudinal research | Population: Disaster survivors (n=36)  Data: Individual interviews  Length of data collection: 6 months | “At T1 and T2, survivors emphasized God being a safe haven (source of protection, comfort, or nurturance). This emphasis was especially pronounced for survivors who were directly affected (their home or business flooded) or had previous disaster exposure to Hurricane Katrina. Overall, survivors consistently emphasized God serving as a stronger and wiser attachment figure, and it was rare for them to report experiencing perceived separation or loss of intimacy from God. At T1 and T2, around 85% of survivors described their current religious attachment as either having a positive affective quality (e.g., closer, stronger) or as no different from before the disaster; around 15% said it had a negative affective quality (e.g., disappointed, strained). In describing their postdisaster religion/spirituality, survivors highlighted (a) God being a source of love, comfort, strength, and hope; (b) actively putting trust/faith in God; and (c) experiencing God through family/community.”  Subheadings: 1) Research Question 1, 2) Research Question 2, 3) Research Question 3 |
| Day, M. R., Thompson, A. R., Poulter, D. R., Stride, C. B., & Rowe, R. (2018). Why do drivers become safer over the first three months of driving? A longitudinal qualitative study. Accident; Analysis And Prevention, 117, 225-231.  Country: UK | "We adopted an innovative longitudinal qualitative design, with thirteen newly qualified drivers completing a total of 36 semi-structured interviews, one, two and three months after acquiring a full UK driving license. The interviews probed high-risk factors for new drivers, as well as allowing space for generating novel road safety issues." (from abstract) | qualitative longitudinal research | Population: New drivers (n=13)  Data: Individual interviews  Length of data collection: 3 months | “Analysis adopted a dual deductive and inductive interpretative thematic approach, identifying three super-ordinate themes: (1) Improvements in car control skills and situation awareness; (2) A reduction in the thrill of taking risks when driving against a background of generally increasing driving speed; (3) Early concerns about their social status in the eyes of other road users during the early stages of driving, which may put pressure on them to drive faster than they felt comfortable with.”  Subheadings: 1) Driving skills (Control skills, Situation awareness), 2) violations and thrill-seeking, and 3) social status and pressure. |
| De Clercq, M., Roland, N., Brunelle, M., Galand, B., & Frenay, M. (2018). The delicate balance to adjustment: A qualitative approach of student's transition to the first year at university. Psychologica Belgica, 58(1), 67-90.  Country: Belgia | "More precisely, two main objectives underlie this approach: (1) to identify the key determinants and events of adjustment regarding the student’s experience; (2) to understand how these determinants and events interact in the adjustment process across the first year." | qualitative longitudinal research | Population: Students in biology (n=17)  Data: Individual interviews  Length of data collection: 12 months | “Four themes (readiness, reaching personal drives, fighting an overwhelming program and becoming a self-regulated learner) and four different events (starting up, click, exhaustion and deficiencies accumulation) were identified in the material disclosing the dynamic nature of adjustment process.”  Subheadings: 1) Theme 1: Readiness (Event 1: Starting out), 2) Theme 2: Reaching personal goals (Event 2: The click), 3) Theme 3: Fighting against an overwhelming system, 4) Theme 4: Becoming a self-regulated learner (Event 3 & 4: The balance between deficiency accumulation and exhaustion) |
| DeGuzman, P. B., Colliton, K., Nail, C. J., & Keim-Malpass, J. (2017). Survivorship care plans: Rural, low-income breast cancer survivor perspectives. Clinical Journal of Oncology Nursing, 21(6), 692-698.  Country: US | "This pilot study explores post-treatment survivorship care planning execution, perception, and needs among rural, low-income cancer survivors." (from abstract) | Qualitative longitudinal research | Population: Rural, low-income cancer survivors (n=7)  Data: Individual interviews  Length of data collection: 6 months | “Rural survivors’ responses reflected lack of knowledge about post-treatment care, including how to assess for cancer recurrence. Delivery of the SCP [survivorship care plans] during the final treatment appointment was inadequate for knowledge retention. Individualized assessment of survivorship needs and education post-treatment may improve long-term health outcomes for this population.”  Subheadings: 1) Themes or Perspectives on Supportive Care and Survivorship Care Plans |
| den Herder-van der Eerden, M., Hasselaar, J., Payne, S., Varey, S., Schwabe, S., Radbruch, L., Van Beek, K., Menten, J., Busa, C., Csikos, A., Vissers, K., & Groot, M. (2017). How continuity of care is experienced within the context of integrated palliative care: A qualitative study with patients and family caregivers in five European countries. Palliative Medicine, 31(10), 946-955.  Country: Belgium, Germany, Hungary, the Netherlands and UK | "Therefore, this article examines how relational, informational and management continuity of care are experienced by patients with advanced cancer, chronic obstructive pulmonary disease (COPD) or heart failure and family caregivers receiving care from integrated palliative care initiatives in five European countries." | Qualitative longitudinal research | Population: Patients with advanced cancer, chronic obstructive pulmonary disease or heart failure and their family caregivers (n=244)  Data: Individual interviews  Length of data collection: 3 months | “Trusted relationships with a small number of key health care professionals to receive tailored care and easily access help were essential. Relational continuity was often deficient, especially with general practitioners. Although informational and management continuity was often lacking in care provision, collaborative integrated palliative care initiatives were related to consistent and coherent care.”  Subheadings: 1) Relational continuity, 2) Informational continuity, 3) Management continuity, |
| Denney-Koelsch, E. M., Côté-Arsenault, D., & Jenkins Hall, W. (2018). Feeling cared for versus experiencing added burden: Parents' interactions with health-care providers in pregnancy with a lethal fetal diagnosis. Illness, Crisis & Loss, 26(4), 293-315.  Country: US | "The focus of this report is parents’ perspectives on health-care provider interactions from the time of prenatal diagnosis through postpartum." | phenomenology | Population: Parents pregnant with a fetus with a lethal fetal diagnosis (n=30)  Data: Individual interviews and joint interviews  Length of data collection: Unclear, "The goal was to interview participants individually and jointly twice during pregnancy and twice after the baby’s birth and subsequent death." | “During individual and joint couple interviews conducted during pregnancy and postpartum, parents described numerous health-care interactions throughout pregnancy. From the participants’ words, Categories of the Content, Process, and Outcome of these interactions emerged. They sought health care for the Content (information and expert guidance), but they desired providers who maintain hope, were caring and nonjudgmental, used a straightforward manner, and showed sensitivity to their developmental journey (Process). They also desired health-care systems that provided continuity of care and minimized waiting times (Process).”  Subheadings: Sample, 1) Interactions With Health-Care Providers (Categories and themes), 2) Content (Information, Expert guidance), 3) Process at the Individual Provider Level (Caring manner, Straightforward and nonjudgmental. Maintains hope and sensitive to developmental process), 4) Process at the System Level (Continuity of care, Minimal waiting times) |
| Donnellan, W. J., Bennett, K. M., & Soulsby, L. K. (2018). How does carer resilience change over time and care status? A qualitative longitudinal study. Aging & Mental Health, 1-7.  Country: UK | "Based on our original sample of 20 spousal dementia carers, the current study uses qualitative longitudinal methods to follow the care status transitions of 13 current and former spousal dementia carers, including: continuing home carers; former carers (institutionalised); and former carers (widowed). We address the following research objectives: i. To examine trajectories of resilience in spousal dementia carers over time and across care status, and ii. To identify which assets and resources from the resilience framework (Windle & Bennett, 2011) are associated with resilience and care status transitions." | Qualitative longitudinal research | Population: Care givers to spouses with dementia (n=23)  Data: Individual interviews  Length of data collection: 18 to 36 months | “Five participants remained resilient (stable resilient), three remained non-resilient (stable non-resilient) and four participants became resilient (non-resilient to resilient). Only one participant became non-resilient (resilient to non-resilient). Stable resilience was characterised by continuing individual assets and community resources. Carers who became resilient returned to previous resources, or gained new resources.”  Subheadings: 1) Trajectories of carer resilience, 2) Assets and resources associated with resilience and care status transitions, 3) Stable resilient, 4) Stable non-resilient, 5) Resilient to non-resilient, 6) Non-resilient to resilient |
| Dowell, A., Stubbe, M., Macdonald, L., Tester, R., Gray, L., Vernall, S., Kenealy, T., Sheridan, N., Docherty, B., Hall, D.-H., Raphael, D., & Dew, K. (2018). A longitudinal study of interactions between health professionals and people with newly diagnosed diabetes. Annals of Family Medicine, 16(1), 37-44.  Country: New Zealand | "We undertook video observation of a cohort of patients with newly diagnosed diabetes to gain an in-depth picture of communication and miscommunication processes within a primary health care team over time." | not described | Population: Patients with newly diagnosed diabetes (n=32)  Data: Observations (video recordning)  Length of data collection: 6 months | “Challenges to effective communication in diabetes care were identified. Although clinicians showed high levels of technical knowledge and general communication skill, initial consultations were often driven by biomedical explanations out of context from patient experience. There was a perception of time pressure, but considerable time was spent with patients by health professionals repeating information that may not be relevant to patient need. Health professionals had little knowledge of what disciplines other than their own do and how their contributions to patient care may differ.”  Subheadings: 1) System of diabetes care, 2) Initial consultations, 3) Preexisting knowledge of diabetes, 4) Duplication of information, 5) Coordination of care, 6) Social context, 7) Structure of the consultation, 8) Evolution of self-management |
| Draaisma, A., Meijers, F., & Kuijpers, M. (2017). Towards a strong career learning environment: Results from a Dutch longitudinal study. British Journal of Guidance & Counselling, 45(2), 165-177.  Country: the Netherlands | "This longitudinal study is designed to gain theoretical and practical insight into the influence of the project ‘COG/SVE’ on the creation of a strong career learning environment. Moreover, this article describes how teachers and project managers of these schools perceive the developments in their own learning environment and the learning environment of their students, since the start of the project. Three research questions will therefore be investigated: (1) How and to what extent does the project ‘COG/SVE’ accomplish strong career learning environments that are dialogical, practice- and inquiry-based? (2) To what extent does the project stimulate collective learning of the teachers? (3) To what extent is transformational leadership present, needed to promote the development of a strong career learning environment?" | Qualitative longitudinal research | Population: Teachers and project managers (n=83)  Data: Individual interviews  Length of data collection: 5 to 6 months | “Results show that, although the school’s vision on career orientation and guidance is clear to the teachers, it is not supported by all of them. It appears that the renewed vision is imposed on the teachers, and this absence of a shared and widely supported vision appears to withhold the teachers and the project managers to engage in collective action.”  Subheadings: 1) Career learning environment, 2) Collective learning of teachers, 3) Transformational leadership, |
| Drake, E. K., & Urquhart, R. (2019). "Figure out what it is you love to do and live the life you love": The experiences of young adults returning to work after primary cancer treatment. Journal of Adolescent & Young Adult Oncology, 8(3), 368-372.  Country: Canada | "The aim of this study was to explore younger adults (YA) cancer survivors’ perspectives on and experiences with return to work (RTW) following primary cancer treatment." | Phenomenology and qualitative longitudinal research | Population: Younger adults cancer survivors (n=5)  Data: Individual interviews  Length of data collection: 9 months | “(…) four themes were identified: YAs [younger adults] face uncertainty about RTW [return to work]; cancer may be a catalyst for career change; employment benefits are important; and YA-specific resources are needed to support RTW.”  Subheadings: 1) Uncertainty about RTW, 2) Cancer as a catalyst for career change, 3) The importance of employment benefits, 4) The benefit of YA-specific resources, |
| Dubé, T., Schinke, R., & Strasser, R. (2019). It takes a community to train a future physician: Social support experienced by medical students during a community-engaged longitudinal integrated clerkship. Canadian Medical Education Journal, 10(3), e5-e16.  Country: Canada | "The purpose of our study was to learn which sources of social support students sought out during a rural-based LIC [longitudinal integrated clerkship]. Therefore, our purpose was to answer the following research question: Which, if any, sources of social support do students experience during a LIC in the context of rural family practice?" | social constructivistic | Population: Medical students year 3 (n=12)  Data: Individual interviews  Length of data collection: 9 months | “The participants described the relationships they developed with various sources of social support such as (a) preceptors, (b) peers, (c) family, (d) health professionals, and (e) community members.”  Subheadings: 1) Preceptors (Physician-student dynamics, Physicians as teachers, Physicians as role models), 2) Peers (Peers within the same community, Peers at other communities), 3) Family (Listening and emotional support, Onsite support), 4) Health professionals (Health professionals as teachers, Interprofessional collaboration), 5) Community members (Welcoming, Integration, Farewell) |
| Due‐Christensen, M., Willaing, I., Ismail, K., & Forbes, A. (2019). Learning about type 1 diabetes and learning to live with it when diagnosed in adulthood: Two distinct but inter‐related psychological processes of adaptation a qualitative longitudinal study. Diabetic Medicine, 36(6), 742-752.  Country: UK and Denmark | "… explored the adaptation processes that adults experience after their diagnosis of Type 1 diabetes in order to elicit areas for supportive intervention to enhance the psychosocial well-being of adults during this phase of life with Type 1 diabetes." | Narrative research & qualitative longitudinal research | Population: Adults diagnosed with type 1 diabetes within the last 3 years (n=30)  Data: Individual interviews  Length of data collection: 6 months | “The narratives could be grouped into three thematic areas: the diagnosis; learning about diabetes; and learning to live with diabetes. Diabetes was characterized as a major disruptor to the established and future life plans of participants, causing significant emotional distress. The narratives showed how early experiences triggered the development of ongoing psychological problems (fear of complications or hypoglycaemia) and diabetes distress, and that navigating different social scenarios (relationships and employment) could be challenging, leading to suboptimal selfmanagement behaviours. The narratives also showed that health professionals often did not attend effectively to participants’ emotional needs after diagnosis, and that the language used frequently triggered negative feelings, such as fear or a sense of failure.”  Subheadings: 1) Overview of narrative structure, 2) Experience of diagnosis (Time of diagnosis, Introduction to diabetes), 3) Learning about diabetes (Learning self-management, Encountering hypoglycaemia, Hyperglycaemia) 3) Learning to live with diabetes (Disruption, Emotional response, Acceptance, Social adaptation) |
| Dunivan, G. C., McGuire, B. L., Rishel Brakey, H. A., Komesu, Y. M., Rogers, R. G., & Sussman, A. L. (2019). A longitudinal qualitative evaluation of patient perspectives of adverse events after pelvic reconstructive surgery. International Urogynecology Journal. 30, 2023–2028.  Country: US | "… to longitudinally examine how patients view surgical AEs [adverse events] overtime and explore how such perspectives may change from the preoperative period to 6 months after surgery." | mixed methods | Population: Women planning pelvic floor disorder surgery (n=20)  Data: Individual interviews  Length of data collection: Approximatley 11 months | “Women’s perceptions of AEs [adverse events] changed as more time passed from surgery. Women identified potential problems related to surgery such as anesthesia complications, pain, injury, catheter issues, and an unsuccessful surgery as the most concerning AEs preoperatively. Postoperatively (6–8 weeks), women expressed concern about functional outcomes (e.g., performing daily activities, symptom reduction). Late postoperatively (6 months), the majority identified unsuccessful surgery, incontinence, and sexual dysfunction as severe AEs. These findings are consistent with prior work that suggests women perceive functional outcomes as fundamental to their recovery.”  Subheadings: no subheadings in result section |
| Dury, S. (2018). Dynamics in motivations and reasons to quit in a Care Bank: A qualitative study in Belgium. European Journal of Ageing, 15(4), 407-416.  Country: Belgium | "Given the previously established importance of examining (changes in) motives for and reasons to quit time bank volunteering, we investigated what motivates people to start and what encourages or prevents them from continuing, and whether these motivations change throughout the project. In addition, we examined the moderating effects of individual and contextual factors that may play a role in the changes in motives and reasons to quit volunteering with a time bank. To achieve our aim, we posed the following research questions: 1a. What motivates volunteers to start participating in the Neighborhood Pension project? 1b. How do these motives change over time? 1c. Which moderating factors change participants’ motives over time? 2a. What reasons to quit do volunteers express at the start of their participation in the project? 2b. How do these reasons change over time? 2c. Which moderating factors change participants’ reasons over time?" | qualitative longitudinal research | Population: Volunteers (n=13)  Data: Focus group interviews, and questionnaire  Length of data collection: 11 months | “There were two main themes, the first of which pertains to older adults’ motives for volunteering with the time bank. These motives are largely attributable to the volunteer organization’s contextual factors. The second theme focuses on reasons for quitting volunteering. Factors for retaining volunteers relate strongly to the purpose of the volunteer organization. Co-production (i.e., engaging the volunteers in the design of the project) and having an attention officer (i.e., a confidant who listens to the volunteers’ worries) are examples of retention strategies. Moreover, earning time credits did not appear to be a motive for continued volunteering.”  Subheadings: 1) 1a. Motives, 2) 1b. Change in motives, 3) 1c. Moderating factors of changes in motives, 4) 2a. Reasons to quit (when starting to volunteer), 5) 2b. Changes in reasons to quit, 6) 2c. Moderating factors of changes in reasons to quit volunteering, |
| Eaton Russell, C., Widger, K., Beaune, L., Neville, A., Cadell, S., Steele, R., Rapoport, A., Rugg, M., & Barrera, M. (2018). Siblings’ voices: A prospective investigation of experiences with a dying child. Death Studies, 42(3), 184-194.  Country: Canada | "study aimed to fill some of the research gaps by examining healthy siblings’ perspectives of their experiences when a brother or sister was dying. Specifically, we investigated: (a) siblings’ involvement with the dying child and (b) the impact of involvement on siblings’ grief, growth, and coping and their interpersonal world within and outside the family." | interpretative description & qualitative longitudinal research | Population: Siblings to dying children (n=10)  Data: Individual interviews  Length of data collection: 12 weeks | “The insights from the 10 siblings revealed complex experiences, both personal and with the ill child, their families, and peers. These experiences were paradoxically sources of strain and of support, revealing the importance of validation and normalization in assisting siblings to successfully navigate the experience.”  Subheadings: Description of sample, 1) Experience with the ill child (Playmates, Companions, Helpers, Strain and support), 2) Experience with family (Separation and strain, Strengthening, Communication), 3) Experience with peers, 4) Personal experiences (Grief and uncertainty, Coping strategies, Development) |
| Eg, M., Frederiksen, K., Vamosi, M., & Lorentzen, V. (2017). How family interactions about lifestyle changes affect adolescents' possibilities for maintaining weight loss after a weight-loss intervention: A longitudinal qualitative interview study. Journal of Advanced Nursing (John Wiley & Sons, Inc.), 73(8), 1924-1936.  Country: Denmark | "Thus, the purpose of this study is to examine how the family interactions related to lifestyle changes influence the adolescent’s potential for maintaining weight loss after participating in a weight-loss treatment programme." | hermeneutic tradition & qualitative longitudinal research | Population: Adolescents and their parents (n=38)  Data: Individual interviews  Length of data collection: 5 years | “Five years after the intervention ended, we found that a family’s interactions were a key factor in how the family handled challenges involved in changing its diet and increasing physical activity and that daily activities in modern families influenced their interaction, as activities demanded so much of the family that it was difficult to sustain the lifestyle changes necessary for the adolescent to maintain achieved weight loss. Supporting the adolescent was far more difficult than families expected; more time-consuming and also a cause of family conflicts. Siblings who did not need to lose weight played a major, but overlooked, role.”  Subheadings: Participant characteristics, 1) Overview of the themes, 2) Challenges in everyday life overrule the lifestyle change in the family, 3) Conflicts and non-aligned expectations in the family challenge lifestyle changes (When family members perpetuate old habits, When parents had split up, When siblings prevent lifestyle change), 4) Interactions in the family can make lifestyle changes a joint project (When the family’s support makes it possible for the adolescent to take responsibility) |
| Elberg Dengsø, K., Tjørnhøj-Thomsen, T., Oksbjerg Dalton, S., Christensen, B. M., Hillingsø, J., & Thomsen, T. (2019). It's all about the CA-19-9. A longitudinal qualitative study of patients' experiences and perspectives on follow-up after curative surgery for cancer in the pancreas, duodenum or bile-duct. Acta Oncologica, 58(5), 642-649.  Country: Denmark | "In this study, we aimed to explore patients’ experiences of follow-up to get a sense of their perspectives on the rehabilitative scope of the current follow-up within the first year after surgery and adjuvant chemotherapy with curative intent." | longitudinal qualitative research | Population: Patients attending current follow-up after treatment for cancer in the pancreas, duodenum or bileduct (n=12)  Data: Individual interviews  Length of data collection: 9 months | “The patients experienced the cancer antigen (CA-19-9) as the center piece of follow-up, with consultations revolving largely around the CA-19-9 results. Parallel to and independent of follow-up, the patients described an array of creative strategies for adapting to their altered bodies and new life situation. The strategies included homemade endeavors to minimize gut symptoms, for example mint tablets or dairy products without lactose: realizing life-long dreams and resolving financial matters; confiding with likeminded outside the family or professionals outside the hospital. First encounters with HCPs [health care providers] were critically important with bad first encounters haunting patients throughout follow-up and good first encounters facilitating trust and reciprocity between patients and HCPs.”  Subheadings: 1) Ambiguous reassurance, 2) Adaptive agency, 3) Learning by doing, 4) Turning point – being true to oneself, 5) Confiding away from home, 6) Critical first encounters, |
| Elliott, K., & McVicar, A. (2018). The impact of prolonged disorders of consciousness on the occupational life of family members. Neuropsychological Rehabilitation, 28(8), 1375-1391.  Country: UK | "To explore the process of occupational adaptation within a group of primary caregivers." | mixed methods | Population: Partners/ primary caregivers (n=6)  Data: Individual interviews, time diaries, and questionnaires  Length of data collection: 6 months | “At 6 months post-injury the greatest amount of carers’ time was allocated to occupations involving the person in a Disorder of Consciousness and less time to social and leisure activities. Participants had difficulty viewing the future, lacked the desire or capacity to engage in previously enjoyed activities. At 12 months those impacts were still evident although changing. The transition to balanced occupational activity is slow, requiring a number of catalysts to change. A conceptual framework for a return to balance is provided, and guidance on advice from family members to families in a similar situation is given.”  Subheadings: 1) Occupational activities, 2) Theme 1. Impact on emotional/health, 3) Theme 2. Change – occupation, role and environment, 4) Catalyst — recognition of a change in role with their relative, 5) Catalyst—a sense that their relative was happy and the environment is safe/ stimulating, 6) Theme 3. Use of time, 7) Catalyst to change — desire to have time for self, 8) Catalyst — change of routine, 9) Catalyst — trust and confidence in leaving their relative, 10) Theme 5. The future, 11) Catalyst — medical stability and realisation of the slowness of potential recovery and long term nature of the condition, 12) Catalyst — support to undertake change, 13) Support and guidance |
| Elliott, M. C., Shuey, E. A., Zaika, N., Mims, L., & Leventhal, T. (2017). Finding home: A qualitative approach to understanding adolescent mothers' housing instability. American Journal of Community Psychology, 60(1), 55-65.  Country: US | "The aim of this study was to use a qualitative approach to explore the unique experiences of low-income Latina adolescent mothers who depended on their families of origin for housing and parenting support and had unstable living conditions (into another doubling up situation—a horizontal move—or into independent housing—a vertical move)." | Ethnography | Population: Low-income Latina adolescent mothers (n=15)  Data: Individual interviews  Length of data collection: 3 years | “Results of analysis employing grounded theory and narrative approaches suggested two types of  instability: ‘Horizontal moves’ between family homes and ‘vertical moves’ between family homes and independent living. Although family support often was fundamental in allowing for participants’ pursuit of independent housing (i.e., vertical moves), it also was associated with greater residential mobility (i.e., horizontal moves), most often in the context of intrafamilial conflict and family instability.”  Subheadnings: 1) Horizontal Moves: Mobility between Family Homes (Relationships and Conflict, Family Instability), 2) Vertical Moves: Mobility between Family Homes and Independent Housing (Housing Assistance, Child Care, Individual Goals) |
| Eriksson, C., Erikson, A., Tham, K., & Guidetti, S. (2017). Occupational therapists experiences of implementing a new complex intervention in collaboration with researchers: A qualitative longitudinal study. Scandinavian Journal of Occupational Therapy, 24(2), 116-125.  Country: Sweden | "The aim of this study was to identify and describe the process of how OTs [occupational therapists] in collaboration with researchers implemented a client-centred ADL [activities of daily living] intervention for persons with stroke." | Qualitative longitudinal research | Population: Occupational therapists (n=33)  Data: Focus group interviews  Length of data collection: 10 months | “Three categories were identified: (1) Including in the scientific world, (2) Involving as an implementer of science and (3) Integrating in a partnership. One core category emerged: The implementation of client-centred intervention enabled the fusion of practice and science. An increased experience of using CADL [client-centred activity of daily living intervention] and support from the researchers changed the OTs’ [occupational therapists] attitudes towards engaging in research from being an outsider to the scientific world to being included and then becoming a part of the research as an implementer of science.”  Subheadings: 1) Including in the scientific world (Positive expectations inspire the integration of the new, A context that allows criticism, Integrating existing and new knowledge, Applying research to practice is needed to feel engagement), 2) Being involved as an actor of science inspires professional pride (Feeling more professional in clinical work, Reaching out and applying knowledge in clinical settings, Shared exchange of experience adds value), 3) Being integrated in a partnership |
| Essery, R., Kirby, S., Geraghty, A. W. A., & Yardley, L. (2017). Older adults' experiences of internet-based vestibular rehabilitation for dizziness: A longitudinal study. Psychology & Health, 32(11), 1327-1347.  Country: UK | "...the study aimed to determine the acceptability and accessibility of an internet-based intervention for delivering VR [viritual reality] to older adults with dizziness, and to provide insight into how they engage with, and utilise, such an intervention. To achieve this, the main objectives of the study were to gain a greater understanding of: how older adults experience internet-based VR, including their perceptions of its impact upon their symptoms; their perceptions of what may help or hinder their engagement with a self-directed VR programme; and how their experiences change over the intervention period." | Phenomenology and qualitative longitudinal study | Population: Older adults with dizziness (n=18)  Data: Individual interviews  Length of data collection: 6 weeks | “The internet intervention was reported to facilitate engagement with rehabilitation exercises, providing motivation to continue through symptom reduction and simple but helpful strategies. It was perceived as informative, reassuring, visually pleasing and easy to use. Barriers to engagement included practicalities, symptoms and doubts about exercise efficacy. Participants’ perceptions did not always remain consistent over time.”  Subheadings: Overview, 1) Perceptions of ‘Balance Retraining’, 2) Facilitators ofengagement with Balance Retraining, 3) Difficulties with engagement in ‘Balance Retraining’ |
| Evans, B. C., Coon, D. W., Belyea, M. J., & Ume, E. (2017). Collective care: Multiple caregivers and multiple care recipients in mexican american families. Journal Of Transcultural Nursing: Official Journal Of The Transcultural Nursing Society, 28(4), 398-407.  Country: US | "We will describe the understudied phenomenon of multiple caregiving in MA [Mexican American] families, asking, “How do MA families adapt to the informal care needs of more than one older family member?”" | Case study | Population: Mexican American families (family members n=47)  Data: Interviews (unclear if individual or joint), and standardized instruments  Length of data collection: 15 months | “We identified three types of collective caregivers: those providing care for multiple family members simultaneously, those providing care successively to several family members, and/or those needing care themselves during their caregiving of others.”  Subheadings: Sample attributes, 1) Cultural adaptation to caregiving: Collective care (Collective caregivers: Singleton and multiple, Collective caregivers: Repetitive caregiving, Collective caregivers: Chain reactions, Adaptation to transitions and turning points) |
| Fadyl, J. K., Theadom, A., Channon, A., & McPherson, K. M. (2019). Recovery and adaptation after traumatic brain injury in New Zealand: Longitudinal qualitative findings over the first two years. Neuropsychological Rehabilitation, 29(7), 1095-1112.  Country: New Zealand | "To investigate the subjective experiences of recovery and adaptation over the first two years after having a TBI [traumatic brain injury] from the perspective of people with the injury and the family and/or friends most closely involved in their recovery (who we termed “significant others”). The specific research questions focused on what helped or hindered recovery and adaptation over time following TBI. " | longitudinal qualitative research | Population: People with traumatic brain injury, their family and /or friends closely involved in the recovery (n=62)  Data: Individual interviews and joint interviews  Length of data collection: About 2 years | “Two overarching themes were captured in the analysis: making room for recovery and cultivating important resources. Themes comprise circumstances and processes that changed and developed over time in different ways for different participants. Key complexities within the overarching themes included the notion of ‘acceptance’ and the role it played in allowing for recovery and adaptation; and the concept of ‘self’ as a resource aiding recovery, but one that is perpetually at risk due to the intersection between the functional and social effects of the injury.”  Subheadings: 1) Central themes, 2) Room for recovery: The complexities of“accepting”and learning how to allow for recovery and adaptation for the individual and their family network, 3) Complexity of acceptance, 4) Allowing me to change what I normally do in order to manage, adapt and recover, 5) Complications: The “tangle” of brain injury symptoms and competing demands, 6) Cultivating important resources (and the intricacies that affect their utility), 7) Developing a concept of TBI recovery, 8) Developing a concept of living with TBI, 9) Connectedness to others: Its utility and its complications, 10) Self as an at-risk resource, |
| Farr, M. (2018). Power dynamics and collaborative mechanisms in co-production and co-design processes. Critical Social Policy, 38(4), 623-644.  Country: UK | "describe empirical examples of co-production and co-design processes within public services" | Case study | Population: Breast cancer patients and staff, e.g., policy managers, senior managers, service managers, project co-ordinators and front-line staff (n=71)  Data: Observations, feedback sheets from co-design events, organisational documents and reports, individual interviews and focus group interviews  Length of data collection: 19 to 22 month (differs between cases) | ”The two cases illustrate how co-production and codesign techniques involve facilitating, managing and co-ordinating a complex set of psychological, social, cultural and institutional interactions. Whilst existing power relations can be challenged in different ways, constant critical reflective practice and dialogue is essential to facilitate more equal relational processes within these techniques, and to institute changes at individual, local community and organisational levels.”  Subheadings: 1) EBCD in breast cancer services, 2)Co-production in local government |
| Felice, J. P., Geraghty, S. R., Quaglieri, C. W., Yamada, R., Wong, A. J., & Rasmussen, K. M. (2017). 'Breastfeeding' without baby: A longitudinal, qualitative investigation of how mothers perceive, feel about, and practice human milk expression. Maternal & Child Nutrition, 13(3), e12426.  Country: US | "Here, we describe results related to the mothers' perspective: their attitudes and perceptions of, strategies for, and experiences with pumping, including their motivations to pump, how they incorporated pumping into infant feeding practices and other obligations, and how their attitudes, perceptions, practices, and experiences changed over time." | Qualitative longitudinal research | Population: Mothers pumping breast milk (n=20)  Data: Individual interviews and observations  Length of data collection: Up to one year | “Mothers' reasons for pumping changed over time and reflected their needs and desires (e.g., latch difficulty, return to work, and increasing their milk supply). Mothers reported that pump type and quality were important to pumping success and that pumping was time‐consuming, costly, and unpleasant compared to feeding at the breast. Regardless of how often mothers pumped, most felt pumping was necessary to meet their infant HM‐feeding [human milk feeding] goals and was a welcome means of sharing with other caregivers the bonding opportunity and tasks they associated with feeding infants. Mothers interpreted output from pumping sessions to understand their ability to provide enough milk to meet their infants' needs. Mothers' reasons for pumping may signal constraints to infant HM feeding that may be addressed with policy changes. Mothers' attitudes and perceptions toward pumping indicate that, although pumping fills important and welcome roles for many mothers, the reality of its practice may make it an unacceptable or infeasible substitute for some.”  Subheadings: 1) Types of pumps used, 2) Mothers' motivations for pumping (Motivations for pumping), 3) Mother's practices for pumping, 4) Mothers' attitudes toward and perceptions of pumping |
| Fletcher, A. C., & Blair, B. L. (2018). Youth disclosure about friendships across the transition to middle school. Journal of Early Adolescence, 38(5), 606-628.  Country: US | "To considered youth disclosure to parents about friends as well as parents’ responses to such disclosure across the transition to middle school. Research Question 1: How does disclosure to parents about friends change or remain stable across the transition to adolescence? Given that adolescence is a time of increased desire for independence from parents, we hypothesize that the most typical pattern of disclosure over time will involve adolescents disclosing less to parents as they transition into adolescence. Research Question 2: What are the emotional components of adolescent disclosure to parents? Based on research indicating that levels of disclosure vary based on adolescent involvement in problem behaviors and perceptions regarding how parents will react to disclosure, we hypothesize that youth will perceive both positive and negative components of disclosure, depending in part on the nature of the information being disclosed and in part on the manner in which parents react to disclosure. Research Question 3: How do youth perceptions regarding their parents and their parents’ reactions to disclosure shape stability and change in disclosure? Consistent with quantitative research findings, we hypothesize that good parenting, encouragement by parents to share information, feelings of trust and security in relationships with parents, and perceptions that parents respect adolescent desires for autonomy will all shape adolescent decisions regarding disclosure." | mixed methods & qualitative longitudinal research | Population: Children grade 5, 6, 7 (n=20)  Data: Individual interviews  Length of data collection: About 3 years | “(…) to categorize participants into four groups: stable complete disclosers, stable partial disclosures, increasing disclosers, and decreasing disclosers. We then identified themes regarding disclosure about friends as they distinguished these disclosure groups. Findings indicated that adolescent disclosure to parents about friends is embedded within the nature of adolescents’ relationships with parents and the manner in which parents respond to adolescent disclosure episodes across time.”  Subheadings: 1) Global Disclosure Classifications Over Time, 2) Universal Disclosure Experiences (Enjoyment of positive disclosure experiences, Parents as a source of advice/support/assistance, Feelings of discomfort involving sharing of negative information, Links between disclosure and parental permission), 3) Stable Complete Disclosers (Trust of parents, History of positive parental response to negative disclosure information), 4) Stable Partial Disclosers (Reasons for withholding positive/neutral information from parents, Reasons for withholding negative information from parents), 5) Changes in Disclosure Over Time (Changes in friends, Intrusive parenting) |
| Forslund, A.-S., Jansson, J.-H., Lundblad, D., & Söderberg, S. (2017). A second chance at life: People's lived experiences of surviving out-of-hospital cardiac arrest. Scandinavian Journal of Caring Sciences, 31(4), 878-886.  Country: Sweden | "The aim of this study was to elucidate meanings of people’s lived experiences and changes in daily life during their first year after surviving OHCA [out-of-hospital cardiac arrest]." | phenomenological hermeneutic interpretation & qualitative longitudinal research | Population: People surviving out-of-hospital cardiac arrest (n=11)  Data: Individual interviews  Length of data collection: 6 months | “The structural analysis resulted in two themes: (i) striving to regain one’s usual self and (ii) a second chance at life, and subthemes (ia) testing the body, (ib) pursuing the ordinary life, (ic) gratitude for help to survival, (iia) regaining a sense of security with one’s body, (iib) getting to know a new self, and (iic) seeking meaning and establishing a future.”  Subheadings: 1) Naive understanding – 6 months, 2) Structural analysis – 6 months (Testing the body, Pursuing the ordinary life, Gratitude for help to survival), 3) Naive understanding – 12 months, 4) Structural analysis – 12 months (Regaining a sense of security with one’s body, Knowing a new self, Establishing meaning and a future), 5) Comprehensive understanding and reflections |
| Foster, K., Mitchell, R., Van, C., Young, A., McCloughen, A., & Curtis, K. (2019). Resilient, recovering, distressed: A longitudinal qualitative study of parent psychosocial trajectories following child critical injury. Injury, 50, 1605-1611.  Country: Australia | "This study aimed to explore parent experiences and psychosocial support needs and identify parent psychosocial trajectories in the 12 months following child critical injury. Research questions were: What are the psychosocial trajectories for parents of critically injured children in the 12 months following injury? What factors facilitate or hinder the psychosocial trajectories of parents of critically injured children in the 12 months following injury?" | longitudinal qualitative research | Population: Parents to a critically injured child (n=27)  Data: Individual interviews  Length of data collection: 12 months | “Three parent trajectory patterns were identified: resilient trajectory where parents were temporarily disrupted by the child’s injury and hospitalisation, but recovered their mental and emotional wellbeing quickly, which was maintained over time; recovering trajectory where parents were initially disrupted at the time of injury but their mental and emotional wellbeing fluctuated over time and had not been fully restored by 12 months; and distressed trajectory where parents experienced significant psychosocial disruption due to their child’s injury and struggled to adapt and regain their wellbeing over time, remaining emotionally distressed about the circumstances and impacts of the injury on their child and family. Illustrative narratives that represent each trajectory are presented.”  Subheadings: 1) Resilient trajectory, 2) Jonathon’s story, 3) Recovering trajectory, 4) Victoria’s story, 5) Distressed trajectory, 6) Maggie’s story |
| Freytag, J., Jiang, Z. J., Giordano, T. P., Westbrook, R. A., McCurdy, S. A., Njue-Marendes, S., & Dang, B. N. (2019). What patient involvement means to new patients at two HIV clinics: A longitudinal, qualitative study. Patient Education & Counseling, 102(8), 1535-1540.  Country: US | "...we aim to: 1) present definitions of patient involvement from the perspectives of patients seeing a new provider, 2) examine physician behaviors that patients identify as cultivating their involvement, and 3) identify some of the ways new patient expectations of involvement change after their first visit with a new provider." | Qualitative longitudinal research | Population: Adults with HIV (n=56)  Data: Individual interviews  Length of data collection: 6 to 12 months | “The mean age was 45 years; 54% were men. Patient definitions of involvement ranged from adherence- to decision-oriented. Analysis revealed three provider communication behaviors that patients perceive as promoting involvement: 1) soliciting patient feedback, 2) discussing treatment options and trade-offs, 3) narrating the decision-making process. Definitions of involvement can change over time as providers reframe the patient's illness as manageable and through perceived partnerships with the provider.”  Subheadings: 1) How patients define involvement (Adherence-oriented involvement, Information-oriented involvement, Decision-oriented involvement), 2) How patients describe physician communication that promotes involvement (Soliciting patient feedback, Discussing treatment trade-offs, Narrating decision-making processes), 3) Changes in patients’ expectations of involvement (Reframed role in care, Involvement in decision making, Formed partnership) |
| Froh, E. B., Deatrick, J. A., Curley, M. A. Q., & Spatz, D. L. (2017). Mothers of infants with congenital diaphragmatic hernia describe "breastfeeding" in the neonatal intensive care unit: "As long as it's my milk, i'm happy". Journal Of Human Lactation, 33(3), 524-532.  Country: US | "This article focuses explicitly on the mothers’ descriptions of what breastfeeding truly means in the context of a diagnosis of CDH [Congenital Diaphragmatic Hernia] for their infants and the course of care in the NICU setting." | Qualitative longitudinal research | Population: Mothers with an infant with a diagnosis of Congenital Diaphragmatic Hernia (n=11)  Data: Individual interviews  Length of data collection: Unclear, during time at hospital | “Six themes emerged from the data: (a) hopeful for breastfeeding, (b) latching on . . . to the pump, (c) we’ve already worked so hard, (d) getting the hang of it—it’s getting easier, (e) a good safety net, and (f) finding a way that works for us.”  Subheadings: 1) Hopeful for breastfeeding, 2) Latching on . . . to the pump, 3) We’ve already worked so hard, 4) Getting the hang of it—it’s getting easier, 5) A good safety net, 6) Finding a way that works for us, |
| Frost, J., Wingham, J., Britten, N., Greaves, C., Abraham, C., Warren, F. C., Jolly, K., Doherty, P. J., Miles, J., Singh, S. J., Paul, K., Rod Taylor, Dalal, H. (2019). Home-based rehabilitation for heart failure with reduced ejection fraction: Mixed methods process evaluation of the REACH-HF multicentre randomised controlled trial. BMJ Open, 9(8), e026039-e026039.  Country: UK | "The work reported here constituted part of the REACH-HF process evaluation that assessed intervention fidelity, patients’ and caregivers’ experiences of trial participation and sought to identify change processes that may be responsible for change in HRQoL (the trial primary outcome)." | Case study and mixed methods | Population: People with heart failure taking part in an patient education program, caregivers and facilitators in the program (n=19)  Data: Individual interviews, fieldnotes, and recorded consultations  Length of data collection: 12 months | “Intervention session attendance with facilitators was high. Fidelity scores were indicative of adequate quality of REACH-HF intervention delivery, although indicating scope for improvement in several areas. Intervention effectiveness was contingent on matching the intervention implementation to the concerns, beliefs and goals of participants. Behaviour change was sustained when shared meaning was established. Respondents’ comorbidities, socio-economic circumstances and existing networks of support also affected changes in healthrelated quality of life.”  Subheadings: study participants, 1) level of intervention received, 2) Fidelity of intervention delivery, 3) Key processes, 4) Adaptation, 5) Competence, 6) Comorbidities, 7) Social context |
| Fu, F., Chen, L., Sha, W., Chan, C. L. W., Chow, A. Y. M., & Lou, V. W. Q. (2018 published online). Mothers' grief experiences of losing their only child in the 2008 sichuan earthquake: A qualitative longitudinal study. Omega – Journal of Death and Dying, 2020, 81(1) 3–17.  Country: China | "Thus, this study not only explores bereaved mothers’ longitudinal grief experiences since the earthquake but also examines their experiences within the Chinese sociocultural contexts." | phenomenological & qualitative longitudinal research | Population: Bereaved mothers’ (n=6)  Data: Individual interviews  Length of data collection: 2 years | “The findings suggest that these mothers’ personal grief experiences evolved: initially, anger toward the cause of their children’s deaths, following despair of meaningless life, guilt and regret, and finally yearning. Although their yearning and missing ebbed after 2 years, these mothers still had unresolved grief. These mothers also faced strained marital relationships and additional pressure from social interactions.”  Subheadings: 1) Anger toward the cause of their children’s deaths, 2) Guilt and regret, 3) Evolving yearning over time (Involuntary yearning, Triggered yearning, Voluntary yearning), 4) Losing family stability, 5) Social interactions bringing additional pressure |
| Gallagher, K., Partridge, C., Tran, H. T., Lubran, S., & Macrae, D. (2017). Nursing & parental perceptions of neonatal care in Central Vietnam: A longitudinal qualitative study. BMC Pediatrics, 17(1), 161-161.  Country: Vietnam | "The aim of this study was to explore changes in the perceptions and attitudes of nurses and parents towards their experiences in the neonatal unit following a neonatal nursing education intervention in a single neonatal unit in central Vietnam. | Qualitative longitudinal research | Population: Parents of infants admitted to the neonatal unit and neonatal nurses who had undertaken the education intervention (n=83)  Data: Individual interviews  Length of data collection: 18 months | “Analysis of nursing transcripts identified 14 basic categories which could be grouped (23) into 3 themes: (1) perceptions of the role of the neonatal nurse, (2) perception of the parental role and (3) professional recollections. Analysis of parent transcripts identified 14 basic categories which could be grouped into 3 themes: (1) information sharing, (2) participation in care, and (3) personal experience.”  Subheadings: 1) Nursing participants, 2) Parent participants, |
| Gammons, R. W., Carroll, A. J., & Carpenter, L. I. (2018). "I never knew I could be a teacher": A student-centered MLIS fellowship for future teacher-librarians. Portal: Libraries & the Academy, 18(2), 331-362.  Country: US | "...results from a mixed methods and longitudinal study identifying the successful components of RTF [Research and Teaching Fellowship] and charting the development of teacher efficacy and identity among participants" (from abstract). "Our research had two directives: (1) to identify successful components of RTF and better understand how these elements contributed to the growth and development of participants; and (2) to chart the development of teacher efficacy and teacher identity among participants." (from background] | grounded theory and mixed methods | Population: Students seeking a master’s of library and information science, their teachers, mentors and administratve staff (n=17)  Data: Indivudual interviews, focus group interviews, written reflections, and questionnaires  Length of data collection: 18 months | “Findings indicate that a strong sense of community, sustained engagement with teaching, and the integration of evidenced-based practice prepare MLIS [master’s of library and information science] students to succeed in a competitive job market.”  Subheadings: 1) Fellows: Written Reflections and Focus Groups, 2) Fellows: Teacher Efficacy and Identity, 3) Fellows: Job Placement, 4) Librarians: Focus Groups, |
| Garbett, K., Harcourt, D., & Buchanan, H. (2017). Using online blogs to explore positive outcomes after burn injuries. Journal of Health Psychology, 22(13), 1755-1766.  Country: UK, US and Australia | "This study aims to build on current research by qualitatively exploring the positive outcomes that may be present following a burn injury since previous literature offers limited knowledge into the specific positive aspects that may arise in this growing population. Acknowledging that they can exist, and exploring specifically what they are, is an important step in guiding burn care practice and may help to challenge assumptions about the ubiquity of negative impacts following burn injuries." | not described | Population: Burn survivors (n=10)  Data: Blog texts  Length of data collection: Unclear | “(…) three themes emerged: shift in self-perception, enhanced relationships and a change in life outlook. Many of these themes contained stories and experiences unique to a traumatic burn injury, suggesting that standardised trauma scales are not effectively measuring the impact of a burn in this population.”  Subheadings: 1) Shift in self-perception, 2) Enhanced relationships (Interpersonal relationships, Interpersonal skills), 3) Change in life outlook |
| Garner, C. D., McKenzie, S. A., Devine, C. M., Thornburg, L. L., & Rasmussen, K. M. (2017). Obese women experience multiple challenges with breastfeeding that are either unique or exacerbated by their obesity: Discoveries from a longitudinal, qualitative study. Maternal & Child Nutrition, 13(3), e12344.  Country: US | "Our aim was to understand obese women’s experiences and perceptions longitudinally, with a normal-weight comparison group, beginning in late pregnancy and continuing through 3months post-partum, to identify key experiences and barriers that are unique to or more common among obese women." | Qualitative longitudinal research | Population: Obese women’s with an infant (n=22)  Data: Individual interviews  Length of data collection: 6 months | “Themes that emerged in analysis were compared between obese and normal-weight women. Differences were identified and described. Prenatally, obese women expressed less confidence about breastfeeding than normal-weight women. Post-partum, obese women and their infants had more health issues that affected breastfeeding, such as low infant blood glucose. Compared with normal-weight women, they also experienced more challenges with latching and positioning their infants. Breastfeeding required more time, props and pillows, which limited where obese women could breastfeed. Obese women also experienced more difficulty finding nursing bras and required more tangible social support than normal-weight women.”  Subheadings: Participants, 1) Presentation of themes, 2) Allison: from ‘they kept taking her away fromme’ to ‘we made it through’, 3) Jana: from ‘small steps’ to ‘getting it over with’, 4) Natalie: from ‘excruciatingly painful’ to ‘the best thing in the world’, 5) Comparison of cases |
| Garrett, S. B., Abramson, C. M., Rendle, K. A., & Dohan, D. (2019). Approaches to decision-making among late-stage melanoma patients: A multifactorial investigation. Supportive Care in Cancer, 27(3), 1059-1070.  Country: US | "...to characterize late-stage melanoma patients holistically as treatment decision-makers." | Ethnography | Population: Late-stage melanoma patients (n=13)  Data: Observations, and individual interviews  Length of data collection: Up to 2 years | “Exploratory analysis revealed eight themes. Heatmap analysis indicated two broad types of patient decision-makers. BReliant outsiders^ relied on providers for medical information, demonstrated low involvement in decision-making, showed a low or later-in-care interest in clinical trials, and expressed altruistic motives. BActive insiders^ accessed substantial medical information and expertise in their networks, consulted with other doctors, showed early and substantial interest in trials, demonstrated high involvement in decision-making, and employed multiple decision-making strategies.”  Subheadings: Sample characteristics, 1) Inductive analysis: exploring themes in decision-making (Involvement in treatment discussions and decisions, Intensive information seeking, Access to medical knowledge, Interest in clinical trials, Optimism, Relationship with oncologist, Decision strategies, Decision-making motives), 2) Ethnoarray analysis: identifying patterns among themes (Characterizing decision-making types) |
| Gaskin, K. L., Wray, J., & Barron, D. J. (2018). Acceptability of a parental early warning tool for parents of infants with complex congenital heart disease: A qualitative feasibility study. Archives of Disease In Childhood, 103(9), 880-886.  Country: UK | "To explore the acceptability and feasibility of a parental early warning tool, called the Congenital Heart Assessment Tool (CHAT), for parents going home with their infant between first and second stage of surgery for complex congenital heart disease." | mixed methods | Population: Parents of infants with complex congenital heart disease needing sugery (n=12)  Data: Individual interviews and joint interviews  Length of data collection: Unclear, a few months, (time point 3 was 8 weeks after time point 1, time point 4 unclear) | “Four main themes emerged: (1) parental preparation and vigilance, (2) usability, (3) mastery, and (4) reassurance and support.”  Subheadings: 1) CHAT recordings, 2) Interviews (Parental preparation and vigilance, Usability, Mastery, Reassurance and support) |
| Gelpí-Acosta, C., Guarino, H., Benoit, E., Deren, S., Pouget, E. R., & Rodríguez, A. (2019). Injection risk norms and practices among migrant Puerto Rican people who inject drugs in New York City: The limits of acculturation theory. International Journal of Drug Policy, 69, 60-69.  Country: US | "This study identifies the P.R. [Puerto Rico] native norms supporting the continued injection risk behavior of migrant Puerto Rican PWID [people who inject drugs] in NYC [New York City] to inform a culturally appropriate risk-reduction intervention." (from abstract) | Grounded theory & qualitative longitudinal research | Population: Migrant Puerto Rican adults who inject drugs (n=40)  Data: Individual interviews  Length of data collection: 12 months | “Most participants (90%) reported having had chronic HCV [hepatitis C], and 22.5% reported being HIV-positive. Syringe- and cooker-/cotton-sharing were widespread in both P.R. [Puerto Rico] and NYC [New York City]. The ubiquitous practice of cleaning used syringes by ‘water-rinsing and air-blowing’ was guided by a normative belief, learned in P.R., that ‘water and air kill HIV.’ Sterile syringe use was not a priority. HCV was not a concern. P.R.-native abstinence-only narratives discouraged Opioid Agonist Treatment (OAT) enrollment among recent migrants (3 years). Experiences with drug dealers, prison-power groups, and injection doctors (‘Gancheros’) in P.R. influenced migrants’ injection risk behavior in NYC. Those who were Gancheros in P.R. continued working as Gancheros in NYC.”  Subheadings: Sociodemographics, 1) Puerto Rico, 2) Drug treatment, 3) Prisons & power groups, 4) Bichotes: street rulers, 5) SEP and Opioid Agonist Treatment access, 6) Water-rinsing & air-blowing 7) Laziness and desperation, 8) “Caballo,” cleaning the cooker, and HCV |
| Gilliland, S. (2017). Physical therapist students' development of diagnostic reasoning: A longitudinal study. Journal of Physical Therapy Education, 31(1), 31-48.  Country: US | "...this qualitative study examined how PT [physical therapy] students’ diagnostic reasoning processes develop through their coursework and clinical experiences." | not described | Population: Physio therapy students (n=6)  Data: Individual interviews and field notes  Length of data collection: About 18 months | “Students’ hypotheses focused on anatomical structures during their first year and shifted to medical diagnoses and biomechanical contributing factors during the second year and following clinical affiliation. Students consistently focused on the anatomical and biomechanical (impairment level) aspects of the patient’s condition and gave minimal attention the patient’s life context (participation level).”  Subheadings: 1) Information collected, 2) Hypotheses and evaluations, 3) Reasoning strategies for patient evaluation, 4) Reasoning errors and omissions |
| Godino, L., Jackson, L., Turchetti, D., Hennessy, C., & Skirton, H. (2018). Decision making and experiences of young adults undergoing presymptomatic genetic testing for familial cancer: A longitudinal grounded theory study. European Journal Of Human Genetics, 26(1), 44-53.  Country: Italy | "...explore the psychosocial implications of pre-symptomatic testing for hereditary cancer in Italian young adults aged 18–30 years" | Grounded theory | Population: New young consult and making an appointment for the cancergenetics clinic (n=15)  Data: Individual interviews  Length of data collection: About 7 months | “Four themes emerged: knowledge, genetic counselling process, decision making and dealing with test results. Although participants grew up with little or no information about their genetic risk, none expressed regret at having the test at a young age. Pre-test counselling was appreciated as a source of information, rather than support for decision making. Decisions were often made autonomously and sometimes conflicted with parents’ wishes. Participants reported no changes in health behaviours after testing.”  Subheadings: 1) Knowledge, 2) Genetic counselling process, 3) Decision making for testing or not, 4) Dealing with the result |
| Goedken, C. C., Moeckli, J., Cram, P. M., & Reisinger, H. S. (2017). Introduction of Tele-ICU in rural hospitals: Changing organisational culture to harness benefits. Intensive & Critical Care Nursing, 40, 51-56.  Country: US | "Our study expands this research through a qualitative investigation of rural ICU [intensive care unit] staff perceptions of Tele-ICU. We conducted a longitudinal qualitative study of three rural ICUs located in the upper Midwest of the United States to illuminate ways in which Tele-ICU can best serve rural facilities. For our study, clinicians and ICU administrators in rural ICUs were asked to discuss perceptions of Tele-ICU on care processes, practices and perceived need before and after implementation." | Qualitative longitudinal research | Population: Rural ICU staff (n=24)  Data: Individual interviews and focus group interviews  Length of data collection: 12 months | “Overall, rural ICU [intensive care unit] staff viewed Tele-ICU as a welcome benefit for their facility. Major themes included: (1) beneficial where recruitment and retention of staff can be challenging; (2) extra support for day shifts and evening, night and weekend shifts; (3) reduction in the number of transfers larger tertiary hospitals in the community; (4) improvement in standardisation of care; and (5) organisational culture of rural ICUs may lead to under-utilisation.”  Subheadings: 1) Theme one: Beneficial where recruitment and retention of staff can be challenging, 2) Theme two: Extra support for day shifts and evening, night and weekend shifts, 3) Theme three: Reduction in the number of transfers to larger tertiary hospitals in the community, 4) Theme four: Improvement in standardisation of care, 5) Theme five: Organisational culture of rural ICUs may lead to under-utilisation |
| González, M. G., Kelly, K. N., Dozier, A. M., Fleming, F., Monson, J. R. T., Becerra, A. Z., Aquina, C. T., Probst, C, P., Hensley B. J., Sevdalis, N., & Noyes, K. (2017). Patient perspectives on transitions of surgical care: Examining the complexities and interdependencies of care. Qualitative Health Research, 27(12), 1856-1869.  Country: US | "The goal of this study is to examine the surgical care pathway from the patient’s point of view to identify experiences and events that influence patient outcomes (e.g., a patient’s satisfaction, anxiety, and discharge readiness) in an effort to improve care transitions and reduce patient burden." | Grounded theory | Population: Adult patients undergoing colon or rectal resection (n=20)  Data: Individual interviews  Length of data collection: One month | “Thematic interdependencies illustrate how most outcomes of care are significantly influenced by two cascades identified as patients’ medical histories and home circumstances. Patients who reported previous medical or surgical histories also experienced less distress during the discharge process, whereas patients with no prior experiences reported more concerns and greater anxiety. Patient dissatisfactions and challenges were due in large part to the contrasts between hospital and home experiences.”  Subheadings: 1) Profile 1: Inpatient/hospital settings, 2) Profile 2: Discharge from hospital, 3) Profile 3: Home environments, 4) Cascades and thematic interconnections |
| Gordon, L., Jindal-Snape, D., Morrison, J., Muldoon, J., Needham, G., Siebert, S., & Rees, C. (2017). Multiple and multidimensional transitions from trainee to trained doctor: A qualitative longitudinal study in the UK. BMJ Open, 7(11), e018583-e018583.  Country: UK | "...to address the research gaps identified above by including trainees working in a range of specialties and contexts, and through its longitudinal study design, follows participants as they move from trainee to trained doctor roles. Our research questions are: (1) What MMTs [multiple and multidimensional transitions] are experienced as participants move from trainee to trained doctor? (2) What facilitates and hinders doctors’ successful transition experiences? (3) What is the impact of MMTs on trained doctors and their significant others?" | Narrative research & qualitative longitudinal research | Population: Trainee doctors (n=20)  Data: Individual interviews, and audio-diaries  Length of data collection: 12 months | “Participants experienced a multiplicity of expected and unexpected, positive and negative workrelated transitions (eg, new roles) and home-related transitions (eg, moving home) during their trainee–trained doctor transition. Factors facilitating or inhibiting successful transitions were identified at various levels: individual (eg, living arrangements), interpersonal (eg, presence of supportive relationships), systemic (eg, mentoring opportunities) and macro (eg, the curriculum provided by Medical Royal Colleges). Various impacts of transitions were also identified at each of these four levels: individual (eg, stress), interpersonal (eg, trainees’ children spending more time in childcare), systemic (eg, spending less time with patients) and macro (eg, delayed start in trainees’ new roles).”  Subheadings: 1) Multiple transitions, (Workplace transitions, Home-life transitions), 2) Supporting successful transitions: Facilitators and inhibitors (Individual-level facilitators/inhibitors, Interpersonal-level facilitators/inhibitors, Systemic-level facilitators/inhibitors, Macro-level facilitators/inhibitors), 3) MMts interacting and impacting (Individual-level impacts, Interpersonal-level impacts, Systemic-level impacts, Macro-level impacts) |
| Graham-Wisener, L., Hanna, J., Collins, L., & Dempster, M. (2019). Psychological adjustment in patients post-curative treatment for oesophageal cancer: A longitudinal interview study. Psychology & Health, 34(8), 901-921.  Country: Ireland | "In contrast to earlier research, this study will involve a sample of survivors for which less time has elapsed since treatment-end and will utilise a longitudinal qualitative design, recommended to suggest mechanisms involved in change during key transition periods by exploring how and why experiences change over time." | Phenomenology & qualitative longitudinal research | Population: Oesophageal cancer patients (n=6)  Data: Individual interviews  Length of data collection: 6 months | “The findings demonstrate an effortful process of adjustment, including recognising and accepting a changed self, fostering control beliefs over the course of the illness and physical sequelae, searching for meaning, developing illness coherence and moving away from self-blame.”  Subheadings: 1) Understanding of illness (Knowledge as empowering or threatening, Moving away from self-blame), 2) Acceptance of a new reality (Realisation of a changed self, Searching for meaning), 3) Relinquishing and harbouring control (Clinicians as uniquely gifted, Personal mastery over a new body) |
| Granbom, M., Taei, A., & Ekstam, L. (2017). Cohabitants' perspective on housing adaptations: A piece of the puzzle. Scandinavian Journal of Caring Sciences, 31(4), 805-813.  Country: Sweden | "The aim of this study was therefore to describe the cohabitants´ expectations and experiences of how a housing adaptation, intended for the partner, impacted on everyday life." | Grounded theory & qualitative longitudinal research | Population: Cohabitants of persons applying for housing adaption (n=9)  Data: Individual interviews  Length of data collection: Avarage of 7 months | “The findings revealed the expectations and experiences in four categories: partners’ activities and independence; cohabitants’ everyday activities and caregiving; couples’ shared recreational/leisure activities; and housing decisions. A core category putting the intervention into perspective was called ‘Housing adaptations – A piece of the puzzle’.”  Subheadings: 1) Housing adaptations – a piece of the puzzle, 2) Partners’ activities and independence, 3) Cohabitants’ everyday activities and caregiving, 4) Couples’ shared recreational/leisure activities, 5) Housing decisions |
| Graney, B. A., Wamboldt, F. S., Baird, S., Churney, T., Fier, K., Korn, M., McCormick, M., Vierzba, T., & Swigris, J. J. (2017). Looking ahead and behind at supplemental oxygen: A qualitative study of patients with pulmonary fibrosis. Heart & Lung, 46(5), 387-393.  Country: US | "In this study, we sought to better understand how patients with PF [pulmonary fibrosis] view and experience O2 [oxygene]- its benefits and challenges - at various stages of their illness." "Interviews were designed to gain appreciation for patients’ understanding of the process of when, why and how O2 was prescribed, and to examine their changing views of the benefits and challenges of O2 over time." | mixed methods | Population: Patients with pulmonary fibrosis (n=5)  Data: Individual interviews , observations, questionnaires  Length of data collection: 10 to 13 months | “Prior to starting supplemental oxygen, participants uniformly expected it would improve their physical function and quality of life. They also expected practical and psychological limitations, which after starting oxygen, they found to be more pronounced than anticipated. Despite the challenges, participants attributed benefits in symptoms, confidence and mobility to oxygen and came to a reluctant acceptance of it. Their expectations for guidance and support were inadequately met.”  Subheadings: 1) Looking ahead (At enrollment, Just prior to starting O2), 2) Looking behind (One month on O2, 9-12 months on O2) |
| Grossman, J. M., Jenkins, L. J., & Richer, A. M., (2018). Parents' perspectives on family sexuality communication from middle school to high school. International Journal Of Environmental Research And Public Health, 15(1).  Country: US | "The current study provides a unique longitudinal examination of parents’ perceptions of continuity and change in teen-parent communication from middle school to high school. The knowledge gained from this study will guide our understanding of how parents do or do not adapt their approaches to sexuality communication to teens’ changing development and sexuality. It also explores the role of teen gender in shaping parents’ approaches to talk with teens about sex and relationships and the content of these conversations." | qualitative longitudinal research | Population: Parents of adolescents from three schools that participated in an evaluation of Get Real (n=29)  Data: Individual interviews  Length of data collection: About 3 years | “Findings showed that many parents adapted their conversations with their teens about sex and relationships as teens developed. Once teens had entered high school, more parents described feeling comfortable with their conversations. However, parents also more often reported that their teens responded negatively to the communication in high school than they had in middle school.”  Subheadings: 1) Reasons for Sexuality Communication, 2) Comfort Talking about Sex, 3) Perceptions ofTeens’ Experiences ofSexuality Communication, 4) Talk about Dating and Relationships, 5) Talk about Readiness for Sex, 6) Talk about Sexual Risk and Protection |
| Grylka-Baeschlin, S., Meyer, T., Lengler, L., van Teijlingen, E., Pehlke-Milde, J., & Gross, M. M. (2019). Postnatal quality of life — A content analysis of qualitative results to the Mother-Generated Index. Women & Birth, 32(2), e229-e237.  Country: Germany and Switzerland | "The aims of this paper were therefore to investigate: (a) details and particularities of the are as of life affected after child birth and thus to identify specific domains and subdomains defining postnatal QoL; (b) changes in the importance of domains specifying QoL within the first weeks postpartum; and (c) the potential role of cultural differences with regard to the content of QoL definitions." | not described | Population: Women in post natal care (n=124)  Data: Open ended questions in questionnairs  Length of data collection: 6 weeks | “Women participated at three days (n = 124) and six and a half weeks (n = 82) postpartum. Eleven domains were identified, each with several subdomains: ‘physical well-being’ (e.g. fatigue), ‘psychological well-being’ (e.g. happiness, emotional confusion), ‘general well-being’, ‘motherhood’ (e.g. bonding with the  baby), ‘family and partnership’ (e.g. time for partner and children), ‘social life’ (e.g. friends, being isolated), ‘everyday life’ (e.g. organisation daily routine), ‘leisure’ (e.g. less time), ‘work life’ (e.g. worries about job), ‘financial issues’ (e.g. less money), and further aspects. The most frequently indicated domains were ‘motherhood’ and ‘family and partnership’. Differences between the stages of assessment and countries were identified.”  Subheadings: 1) Physical well-being, 2) Mental well-being, 3) General well-being, 4) Motherhood, 5) Family and partnership, 6) Social life, 7) Everyday life, 8) Leisure, 9) Work life, 10) Financial issues, 11) Further aspects, 12) Differences between Swiss and German women |
| Gulbas, L. E., Guz, S., Hausmann-Stabile, C., Szlyk, H. S., & Zayas, L. H. (2019). Trajectories of well-being among latina adolescents who attempt suicide: A longitudinal qualitative analysis. Qualitative Health Research, 29(12), 1766-1780.  Country: US | "Research Question 1: How do trajectories of well-being vary among Latina teens after a suicide attempt? Research Question 2: What risk and protective factors might contribute to different trajectories?" | longitudinal qualitative research | Population: Latina teens after a suicide attempt (n=17)  Data: Individual interviews  Length of data collection: About one year | “Analysis revealed three distinct trajectories after a suicide attempt: resilience, tenuous growth, and chronic stress. Our findings elucidate potential factors that contribute to resilience following a suicide attempt and underscore the importance of prevention and intervention programs that foster adolescents’ connectivity across ecodevelopmental contexts.”  Subheadings: 1) Trajectories of well-being, 2) Resilience trajectory, 3) Tenuous growth trajectory, 4) Chronic stress trajectory |
| Guldager, R., Willis, K., Larsen, K., & Poulsen, I. (2019). Relatives' strategies in subacute brain injury rehabilitation: The warrior, the observer and the hesitant. Journal Of Clinical Nursing, 28(1), 289-299.  Country: Denmark | "The aim of this study was to undertake theoretical‐empirical analysis of relatives’ strategies and practices in the rehabilitation process as evident in meetings with providers. We explored the experience of the rehabilitation process from the perspectives of relatives of patients with a traumatic brain injury [TBI]. Our research question was 'what kind of strategies do relatives of patients with a TBI apply and use in the rehabilitation process?' " | qualitative longitudinal research | Population: Relatives of patients with a TBI (n=11)  Data: Observations and individual interviews  Length of data collection: Unclear, over a few weeks | “Drawing on Bourdieu's concept of strategy, three relative positions were identified, the warrior, the observer and the hesitant. These positions illustrate how different relative positions and their related dispositions influence the strategies used by relatives of patients with a severe traumatic brain injury evidenced in how they act, participate and relate to both the patient and the providers during the course of rehabilitation.”  Subheadings: 1) Bodily and verbal interaction, 2) Relationship with the providers, 3) Perception of own involvement, 4) Perception of the patients’ illness |
| Hamblin, K. (2017). Telecare, obtrusiveness, acceptance and use: An empirical exploration. British Journal of Occupational Therapy, 80(2), 132-138.  Country: UK | "...to examine the daily experiences of telecare. The research questions addressed in this paper are therefore: (1) whether Hensel et al.’s (2006) obtrusiveness framework is applicable empirically to the English context; and (2) what is the impact of the dimensions of obtrusiveness on the acceptance and use of telecare?" | ethnography & qualitative longitudinal research | Population: People over 65 years, had memory problems and/or susceptibility to falls, were living in the communit, and were either ‘new’ or established telecare users (n=25)  Data: Individual interviews  Length of data collection: 11 to 24 months (differed between the two data collection sites) | “The obtrusiveness model is broadly applicable to the UK context, but there are also two further issues which affected the acceptance and use of telecare: the degree of control a service user feels they have and the information and support they receive in using their devices.”  Subheadings: 1) The physical dimension, 2) The usability dimension, 3) The privacy dimension, 4) The functional dimension, 5) The human interaction dimension, 6) The self-concept dimension, 7) The routine dimension, 8) The sustainability dimension, 9) The control dimension, 10) The information and ongoing support dimension |
| Hanrahan, F., & Banerjee, R. (2017). ‘It makes me feel alive’: The socio-motivational impact of drama and theatre on marginalised young people. Emotional & Behavioural Difficulties, 22(1), 35-49.  Country: UK | "We aimed to explore the participants’ experiences of long-term involvement in drama and theatre work from an idiographic, phenomenological perspective, and to consider the young people’s narratives in relation to the psychological mechanisms identified by our model of disaffection/engagement." | Phenomenology & qualitative longitudinal research | Population: Young people conducting a theatre project (n=4)  Data: Individual interviews  Length of data collection: 22 months | “In particular, the analysis highlighted the pivotal role of interpersonal relationships and a nurturing environment in re-engaging young people. Some participants’ accounts also suggested that drama provides a uniquely engaging and therapeutic way to reflect on, express and explore experiences.”  Subheadings: 1) A nurturing space (A nurturing space: supportive boundaries, A nurturing space: growth of trust and being valued, A nurturing space: it feels like we’re all a family), 2) Something for myself (Something for myself: a positive activity to fill time), 3) Changing the story (Changing the story: my life’ssodifferent) |
| Hansen, C. A., Abrahamsen, B., Konradsen, H., & Pedersen, B. D. (2017). Women's lived experiences of learning to live with osteoporosis: A longitudinal qualitative study. BMC Women's Health, 17(1), 17-17.  Country: Denmark | "The objective was to explore what characterizes women’s experiences of living with osteoporosis in the first year after diagnosis, when patients are prescribed anti-osteoporotic treatment, without experiencing an osteoporotic fracture." | Phenomenology & qualitative longitudinal research | Population: Women 65 years or older who attended DXA scan and diagnosed with osteoporosis (n=15)  Data: Individual interviews  Length of data collection: One year | “The participants’ experiences could be described in two key themes developed through the analysis: 1) ‘To become influenced by the medical treatment’ which consisted of two sub-themes ‘taking the medication’,  and ‘discontinuing the medication’.2) ‘Daily life with osteoporosis’, which was characterized by three sub-themes: ‘interpretation of symptoms’, ‘interpretation of the scan results’ and ‘lifestyle reflections’. The results highlighted that learning to live with osteoporosis is a multifaceted process that is highly influenced by the medical treatment. In some cases, this is a prolonged process that can take around one year.”  Subheadings: 1) To become influenced by the medical treatment (Taking the medication, Discontinuing the medication), 2) Discontinuing the medication (Interpretation of symptoms, Interpretation of the DXA result, Lifestyle reflections) |
| Hansen, F., Berntsen, G. K. R., & Salamonsen, A. (2018). "What matters to you?" A longitudinal qualitative study of Norwegian patients' perspectives on their pathways with colorectal cancer. International Journal of Qualitative Studies on Health And Well-Being, 13(1), 1548240-1548240.  Country: Norway | "We aim to offer rich descriptions of the participants’ lives lived with colorectal cancer and what they themselves emphasize as important. The research questions analysed in this article are: What is important for persons diagnosed with colorectal cancer during their patient pathways? And, based on these findings, which significant features do the patient pathways share?" | qualitative longitudinal research | Population: Adults between 18-70 with rectal cancer in treatment (n=10)  Data: Individual interviews and diaries  Length of data collection: One year | “We found that: (1) patients have an initial focus on ‘biological goals’ and conventional treatment; (2) pathways are unique and dynamic; (3) family and friends affected patient pathways positively with respect to meaningfulness and quality of life, but for some participants also negatively because there were heavy burdens of caretaking; (4) receiving help in the health care system depended on the patients’ navigation skills; (5) pluralism in health-seeking behaviour was important in all patient pathways.”  Subheadings: 1) Initial focus on biological goals and conventional treatment, 2) Initial focus on biological goals and conventional treatment, 3) Impact of family and friends in a pathway, 4) Navigation in the national health care system, 5) Pluralism in health-seeking behaviour |
| Hansen, L., Rosenkranz, S. J., Wherity, K., & Sasaki, A. (2017). Living with hepatocellular carcinoma near the end of life: Family caregivers' perspectives. Oncology Nursing Forum, 44(5), 562-570.  Country: US | "To explore family caregivers perspectives about caring for patients with terminal HCC [Hepatocellular carcinoma] as the patients approached the end of life" | Qualitative longitudinal research | Population: Family caregivers of adult patients with a diagnosis of HCC (n=13)  Data: Individual interviews  Length of data collection: 6 months | “Five core categories and nine subcategories were identified. From the time of the terminal diagnosis to the end of life, family caregivers felt unprepared, uncertain, and in need of information. They struggled with whether symptoms were HCC- [Hepatocellular carcinoma] or cirrhosis-related.”  Subheadings: 1) Quality of Relationship, 2) Response to Terminal Hepatocellular Carcinoma Diagnosis (Desire for information, Managing the stigma, Change in lifestyle), 2) Hepatocellular Carcinoma Progression: Symptom Interpretation (“I really do not see it”, Uncertainty, Fluctuating symptoms, Visibly getting worse), 3) Treatment Challenges (Desire for treatment information, Getting through treatment), 4) Getting through treatment |
| Hanson, C. L., Oliver, E. J., Dodd-Reynolds, C. J., & Allin, L. J. (2019). How do participant experiences and characteristics influence engagement in exercise referral? A qualitative longitudinal study of a scheme in Northumberland, UK. BMJ Open, 9(2), e024370-e024370.  Country: UK | "This longitudinal qualitative study aimed to gain an insight into differential engagement through understanding participant experiences of an ERS [exercise referral scheme]" | qualitative longitudinal research | Population: Participants in ERS program (n=15)  Data: Individual interviews  Length of data collection: 12 to 20 weeks | “Three overarching themes emerged. First, ‘success’, with engaged participants focused on health outcomes and reported increases in physical activity. Second, ‘struggle’, with short-term success but concerns regarding continued engagement. Participants reported scheme dependency and cyclical needs. Finally, ‘defeat’, where ill health, social anxiety and/or poor participation experience made engagement difficult.”  Subheadings: Participant characteristics, 1) Overarching themes, 2) Success: increased PA and improved health, 3) Struggle: cyclical needs and scheme dependency, 4) Defeat: inappropriate referral or poor participation experience |
| Harris, M. (2017). Managing expense and expectation in a treatment revolution: Problematizing prioritisation through an exploration of hepatitis C treatment 'benefit'. The International Journal of Drug Policy, 47, 161-168.  Country: UK | "To explore participants’ narratives of HCV [hepatitis C virus] treatment expectation and map their anticipatory accounts to those of post treatment ‘benefit." "This aim of this paper is to present analyses pertaining to participant treatment decision making, expectations and outcomes in a period of HCV biomedical transition and, through doing so, to explore the relevance and fit of contemporary public health discourses regarding ‘patient important benefits’ and HCV treatment prioritisation for this population." | Qualitative longitudinal research | Population: People living with hepatitis C and providers (n=28)  Data: Individual interviews, and observations  Length of data collection: 18 months | “Twenty-two participants commenced treatment. The majority who were unable to access DAAs [Direct-acting antivirals] chose to commence interferon-based treatment immediately rather than wait. Participants accounted for treatment urgency in relation to three interrelated narratives of hope and expectation. HCV [hepatitis C virus] treatment promised: social reconnection; social redemption and a return to ‘normality’. For many with successful treatment outcomes, these benefits appeared to be realised.”  Subheadings: 1) Treatment decision-making, 2) Treatment urgency, 3) Treatment hopes and expectation (Social reconnection, Redemption and return), 4) Treatment outcomes (Reconnection: exceeding the social, Return and redemption: a reappraisal, Expectations: exceeded and constrained) |
| Harris, M., & Rhodes, T. (2018). Caring and curing: Considering the effects of hepatitis C pharmaceuticalisation in relation to non-clinical treatment outcomes. The International Journal On Drug Policy, 60, 24-32.  Country: UK | "We consider the implications of simplified treatment provision in the era of direct acting antivirals (DAAs) for the realisation of non-clinical benefits., how engagement in HCV care is giving meaning for patient and provider" | Ethnography & qualitative longitudinal research | Population: Patient with hepatitis C, and providers (n=38)  Data: Individual interviews, observations and field notes  Length of data collection: Over 3 years | “Patient accounts of care accentuate the transformative value of interferon-based HCV [hepatitis C virus] treatment derived through non-clinical benefits linked to identity and lifestyle change. Such care is constituted as extending beyond the virus and its biomedical effects, with nurse specialists positioned as vital to this care being realised. Provider accounts emphasise the increased pharmaceuticalisation of HCV treatment; whereby care shifts from the facilitation of therapeutic relationships to pharmaceutical access.”  Subheadings: 1) Experiences of receiving HCV treatment: relational care (Care beyond hepatitis C, Care beyond expectation, Utilitarian care), Providing HCV treatment: anticipating disruption to care relations (Care beyond hepatitis C, Becoming superfluous, Diversifying to survive, Managing marginality) |
| Harvey, D., Foster, M., Quigley, R., & Strivens, E. (2018). Care transition types across acute, sub-acute and primary care. Journal of Integrated Care, 26(3), 189-198.  Country: Australia | "The purpose of this analysis is to distil the types and commonalities of care transitions of older people by examining the individual experiences of older people, and their carers, who transitioned from community through acute and sub-acute care to home as a case study of transition." | Case study | Population: Older people and their carers (n=19)  Data: Individual interviews, and service use data from medical records  Length of data collection: About one month | “Three types of care transitions were derived from the analysis: manageable, unstable and disrupted. Each type had distinguishing characteristics and older people could experience elements of all types across the system. Transition types varied according to personal and systemic factors.”  Subheadings: 1) Manageable care transitions, 2) Unstable care transitions, 3) Disruptive care transitions, |
| Harvey, D., Foster, M., Strivens, E., & Quigley, R. (2017). Improving care coordination for community-dwelling older Australians: A longitudinal qualitative study. Australian Health Review, 41(2), 144-150.  Country: Australia | "To (1) describe care transition experiences from multiple perspectives and identify personal, systemic and local factors affecting these experiences; and (2) identify applied solutions that could be used to enhance implementation and capacity of the GEM [Geriatric Evaluation and Management] model." | Case study | Population: Patients, carers and service providers (n=39)  Data: Individual interviews and focus groups interviews  Length of data collection: About one month | “Patients and carers experienced care transitions as dislocating and unpredictable within a complex and  turbulent service context. The experience was characterised by precarious self-management in the community, floundering with unmet needs and holistic care within the GEM [Geriatric Evaluation and Management] service. Patient and carer attitudes to seeking help, quality and timeliness of communication and information exchange, and system pressure affected care transition experiences.”  Subheadings: 1) Patient, carer and service provider perspectives on care transitions, 2) Factors affecting care transitions |
| Harvey-Lloyd, J. M., Morris, J., & Stew, G. (2019). Being a newly qualified diagnostic radiographer: Learning to fly in the face of reality. Radiography, 25(3), e63-e67.  Country: UK | "This study explored the experience of transition from student to practitioner in diagnostic radiography and in particular focused on being and becoming a radiographer." | Phenomenology | Population: Students in radiography (n=9)  Data: Individual interviews  Length of data collection: 12 months | “Six main themes were identified. This paper explores the subtheme ‘reality hits’ which strongly featured in the three and six month interviews.”  Subheadings: No subheadings in result section |
| Hatcher, A. M., Hofstedler, E. L., Doria, K., Dworkin, S. L., Weke, E., Conroy, A., Bukusi, E. A., Cohen, C. R., & Weiser, S. D. (first published 2019). Mechanisms and perceived mental health changes after a livelihood intervention for HIV-positive Kenyans: Longitudinal, qualitative findings. Transcultural Psychiatry, 2000, 57(1) 124–139.  Country: Kenya | "We examined how a multi-sectoral livelihood intervention affected mental health for HIV positive farmers in Kenya. Using qualitative methodology, we explored participant definitions of mental health, perceived changes in mental health due to the intervention, and mechanisms through which the intervention may have influenced mental health outcomes." | qualitative longitudinal research | Population: HIV-positive farmers in Kenya (n=54)  Data: Individual interviews  Length of data collection: 12 months | “Participants reported numerous mental health improvements post-intervention including reduced stress, fewer symptoms of anxiety, improved mood, lower depressive symptoms, fewer repetitive and ruminating thoughts, and more hopefulness for the future. Improvements in mental health appear to occur via several mechanisms including: 1) better food security and income; 2) increased physical activity and ability to create fruitful routines around farm work; and, 3) improved sense of self as an active member of the community.”  Subheadings: Participant characteristics, 1) Perceived mental health improvements, 2) Mechanisms towards improved mental health, 3) Productive farming routines reduced depressive and worrisome thoughts, 4) Reframed social identities: Future outlook and selfesteem, 5) Repayment of loans was a new source of stress |
| Henderson, G. E., Waltz, M., Meagher, K., Cadigan, R. J., Jupimai, T., Isaacson, S., Ormsby, N. Q., Colby, D. J., Kroon, E., Phanuphak, N., Ananworanich, J., Peay, H. L. (2019). Going off antiretroviral treatment in a closely monitored HIV "cure" trial: Longitudinal assessments of acutely diagnosed trial participants and decliners. Journal of the International AIDS Society, 22(3), e25260-e25260.  Country: Thailand | "We aim to understand motivations to join or decline and whether, how, and why decision satisfaction may change over time" | not described | Population: People with HIV in ART trails (n=14)  Data: Individual interviews and questionnairs  Length of data collection: 3 to 4 months | “The eight participants and six decliners had low overall decisional conflict, which remained low over time. Decision making was more difficult for decliners than participants, at least initially. While all interviewees described being satisfied with their decisions, our study identified important negative consequences for a few individuals, including seroconversion, negative experiences with optional procedures and disappointment due to rapid viral rebound.”  Subheadings: 1) Why join? Perceptions of participants, 2) Why not join? Perceptions of decliners, 3) How did participants experience the RV411 trial? 4) How were decisions about RV411 assessed at the final interviews? 5) What do quantitative decisional conflict scores reveal? 6) Why do some participants’ decisional conflict scores rise? 7) Why do some decliners have higher initial decisional conflict? |
| Hill, C., Knafl, K. A., Docherty, S., & Santacroce, S. J. (2019). Parent perceptions of the impact of the Paediatric Intensive Care environment on delivery of family-centred care. Intensive & Critical Care Nursing, 50, 88-94.  Country: US | "We conducted a secondary analysis of data from a longitudinal study of parent involvement in decision-making in an intensive care environment to further elaborate the role of the physical and cultural environment in parent perception of the delivery of FCC [family-centered care]." | Case study | Population: Parents to an infant in ICU (n=6)  Data: Interviews (unclear if individual interviews or joint interviews)  Length of data collection: About 12 months (varied between participants) | “The family-centred care core concepts of information sharing, participation, respect and dignity were present in parent interviews. Parents indicated that the physical and cultural environment of the pediatric intensive care unit impacted their perceptions of how each of the core concepts was implemented by clinicians. The unit environment both positively and negatively impacted how parents experienced their infant’s hospitalisation.”  Subheadings: 1) Information sharing, 2) Participation, 3) Respect and dignity |
| Hoag, J., Igler, E., Karst, J., Bingen, K., & Kupst, M. J. (2019). Decision-making, knowledge, and psychosocial outcomes in pediatric siblings identified to donate hematopoietic stem cells. Journal of Psychosocial Oncology, 37(3), 367-382.  Country: US | "The current study was conducted to (1) describe the decision-making experience and psychosocial outcome of sibling donors utilizing a mixed-methods approach, and (2) determine the feasibility of completing a prospective and longitudinal assessment of sibling donors at a single institution." | mixed methods | Population: Sibling donors of Hematopoietic stem cell (n=12)  Data: Interviews and questionnairs  Length of data collection: Unclear, 6 months between time point 2 and 3 | “Qualitative analysis indicated donors want to make their own decision about donation but may not be given the option or may feel that there is no choice given their limited awareness of alternative options. Donors felt well prepared for the donation procedure but demonstrated a poor understanding of possible recipient outcomes. A minority of donors endorsed emotional distress prior to and after donation; however, this was not linked to recipient health. Forty percent of donors felt that they had inadequate support following their donation. Small sample size restricted quantitative data analysis.”  Subheadings: 1) Feasibility, 2) Time 1: Pre-donation, Semi-structured interview (Decision-making, Knowledge, Family relationships, Emotional and behavioral functioning, Coping, Family relationships), 3) Time 2: Postharvest, Semi-structured interview (Donation experience, Pain), 4) Time 3: Six months post-donation, Semi-structured interview (Looking back on the experience, Psychosocial support, Advice to other potential donors, Family relationships, Emotional and behavioral functioning, Coping, Family relationships) |
| Hodson, T., Gustafsson, L., & Cornwell, P. (2019). "Just got to live life as it comes": A case study of the spousal-dyad longitudinal mild stroke transitional experience. Brain Injury, 33(9), 1200-1207.  Country: Australia | "Consequently, the aim of this study was to provide an in-depth exploration of the mild stroke experience of one couple who received a novel health service within the Australian context. Specifically, this research examined the experience of transitioning from acute hospital care to the community following a mild stroke, for a spousal dyad in the first 9-months post-hospital discharge." | Phenomenology & case study | Population: Patient and spouse following a mild stroke (n=2)  Data: Individual interviews  Length of data collection: 9 months | “Two themes were identified: (1) The Unexpected, Undesirable and Short-Lived, and (2) The New  ‘Normal’. The first theme reflects the confusion, adjustment and adaptation that occurred for the couple, especially during the first month at home. The second represents the couple’s journey back to their everyday lives following hospital discharge, but also the questions and changes that remained present at 9-months post-discharge.”  Subheadings: Participants & context, Qualitative findings, 1) The unexpected, undesirable and short lived, 2) The new ‘normal’ |
| Hopia, H., Miettinen, S., Miettinen, M., & Heino-Tolonen, T. (2019). The voice of paediatric oncology nurses: A longitudinal diary study of professional development. European Journal of Oncology Nursing, 42, 28-35.  Country: Finland | "The purpose of this paper is to describe how nurses perceive their professional development in paediatric oncology nursing by answering the question regarding the factors associated with professional development during the two-year training period. The rationale was that a deeper insight into the various aspects of professional development in paediatric oncology nursing from the nurses’ perspective would be gained in this. Furthermore, the objective was to conceptualise the phenomenon under the study." | Qualitative longitudinal research | Population: Nurses (n=17)  Data: Diaries  Length of data collection: 20 months | “Professional development is linked with a strong knowledge base in nursing, which involves the use of nursing methods and up-to-date nursing practices. Professional development is also linked with the use of medical knowledge, which manifests as a deep understanding of cancers and their treatment.”  Subheadings: 1) Strong knowledge base in nursing (Use of nursing methods, Up-to-date nursing work), 2) Use of medical knowledge (Deep understanding of cancers and their treatment) |
| Horter, S., Bernays, S., Thabede, Z., Dlamini, V., Kerschberger, B., Pasipamire, M., Rusch, B., & Wringe, A. (2019). "I don't want them to know": How stigma creates dilemmas for engagement with Treat-all HIV care for people living with HIV in Eswatini. African Journal of AIDS Research, 18(1), 27-37.  Country: Eswatini | "...we aim to examine how stigma shapes people living with HIV experiences with HIV, and engaging with HIV treatment and care services under Treat-all in Shiselweni, Eswatini (formerly Swaziland)." | Qualitative longitudinal research | Population: People living with HIV (n=30)  Data: Individual interviews  Length of data collection: Up to 12 months | “Stigma was pervasive within the narratives of PLHIV [people living with HIV], framing their engagement with treatment and care. Many asymptomatic PLHIV were motivated to initiate ART [antiretroviral therapy] in order to maintain a “discreditable” status, by preventing the development of visible and exposing symptoms. However, engagement with treatment and care services could itself be exposing. PLHIV described the ways in which these “invisibilising” benefits and exposing risks of ART were continually assessed and navigated over time. Where the risk of exposure was deemed too great, this could lead to intermittent treatment-taking, and disengagement from care.”  Subheadings: 1) HIV status concealment to avoid anticipated stigma, 2) ART offering the potential to maintain good health and a hidden HIV status, 3) The risk of HIV status exposure undermining engagement with HIV treatment and care, 4) HIV status acceptance countering anticipated stigma |
| Horter, S., Wringe, A., Thabede, Z., Dlamini, V., Kerschberger, B., Pasipamire, M., Lukhele, N., Rusch, B., & Seeley, J. (2019). "Is it making any difference?" A qualitative study examining the treatment-taking experiences of asymptomatic people living with HIV in the context of Treat-all in Eswatini. Journal of The International AIDS Society, 22(1), e25220-e25220.  Country: Eswatini | "We examine asymptomatic PLHIV’s [people living with HIV] experiences engaging with Treat-all care in the Kingdom of Eswatini (formerly named Swaziland), including how treatment-taking is navigated and motivated over the longer term (at least 12 months after initiation)." | Qualitative longitudinal research | Population: People with HIV and health care providers (n=37)  Data: Individual interviews and fieldnotes  Length of data collection: About 8 months | “It was important for PLHIV [people living with HIV] to perceive the need for treatment, and to have evidence of its effectiveness to motivate their treatment-taking, thereby supporting engagement with care. For some, coming to terms with a HIV diagnosis or re-interpreting past illnesses as signs of HIV could point to the need for ART [antiretroviral therapy] to prevent health deterioration and prolong life. However, others doubted the accuracy of an HIV diagnosis and the need for treatment in the absence of symptoms or signs of ill health, with some experimenting with treatment-taking as a means of seeking evidence of their need for treatment and its effect. Viral load monitoring appeared important in offering a view of the effect of treatment on the level of the virus, thereby motivating continued treatment-taking.”  Subheadings: Study participants, 1) The perceived need for treatment influencing engagement with treatment and care, 2) Doubts about treatment need and effect undermining treatment-taking, 3) Seeking evidence of the treatment’s effectiveness |
| Horwood, C., Jama, N. A., Haskins, L., Coutsoudis, A., & Spies, L. (2019). A qualitative study exploring infant feeding decision-making between birth and 6 months among HIV-positive mothers. Maternal & Child Nutrition, 15(2), e12726-e12726.  Country: South Africa | "This paper presents results from a study to explore infant feeding practices among HIV positive women and identify key role players influencing their infant feeding decisions. […] This study adopted a longitudinal qualitative design to prospectively capture critical moments and processes involved in infant feeding choices made by HIV‐infected women over the period from birth to 6 months. This methodology was chosen as the most appropriate method to explore the lived experience of change and capturing transitions" | Qualitative longitudinal research | Population: HIV-positive mothers to infants (n=11)  Data: Individual interviews  Length of data collection: About 6 months | “Mothers reported receiving strong advice from health workers [HWs] to exclusive breastfeedig [EBF] and made decisions based primarily on HWs advice, resisting contrary pressure from family or friends. The main motivation for EBF was to protect the child from HIV acquisition, but sometimes fear of mixed feeding led to mothers stopping breastfeeding entirely. Infant feeding messages from HWs advice were frequently inadequate and out of date, and failed to address mothers' challenges. Minimal support was provided for exclusively formula feed [EFF].”  Subheadings: 1) Role of health workers in infant feeding decision‐making, 2) High level of trust in health workers advice, 3) Lack of HW support for breastfeeding challenges, 4) Fear of HIV transmission influenced feeding choices, 5) Importance of mothers' self‐efficacy in sustaining feeding choice |
| Jama, N. A., Wilford, A., Haskins, L., Coutsoudis, A., Spies, L., & Horwood, C. (2018). Autonomy and infant feeding decision-making among teenage mothers in a rural and urban setting in KwaZulu-Natal, South Africa. BMC Pregnancy and Childbirth, 18(1), 52-52.  Country: South Africa | "...explore teenage mothers' narratives about infant feeding choices and practices from birth to 6 months" | Qualitative longitudinal research | Population: Women aged 15 years or older who were more than 36 weeks pregnant (n=10)  Data: Individual interviews  Length of data collection: 6 months | “Findings from this study showed that teenage mothers had knowledge about recommended feeding practices. However, our findings suggest that these mothers were not involved in infant feeding decisions once they were at home, because infant feeding decision-making was a role largely assumed by older mothers in the family. Further, the age of the mother and financial dependency diminished her autonomy and ability to influence feeding practices or challenge incorrect advice given at home. Most feeding advice shared by family members was inappropriate, leading to poor infant feeding practices among teenage mothers. Returning to school and fear of breastfeeding in public were also barriers to exclusive breastfeeding.”  Subheadings: 1) Infant feeding intentions and rationale, 2) Process of infant feeding decision making in the household, 3) Autonomy and teenage dependency, 3) Managing conflict between clinic advice vs. home advice, 4) Poor feeding advice from key family members, 5) Returning to school |
| Jama, N. A., Wilford, A., Masango, Z., Haskins, L., Coutsoudis, A., Spies, L., & Horwood, C. (2017). Enablers and barriers to success among mothers planning to exclusively breastfeed for six months: A qualitative prospective cohort study in KwaZulu-Natal, South Africa. International Breastfeeding Journal, 12, 1-13.  Country: South Africa | "We report the findings of a longitudinal cohort study, conducted among mothers who planned to exclusively breastfeed their infants for six months, and describe the factors that facilitated or acted as barriers to achieving their goal." | Qualitative longitudinal research | Population: Women aged 15 years or older who were more than 36 weeks pregnant (n=22)  Data: Individual interviews  Length of data collection: 6 months | “A total of 125 interviews were conducted between November 2015 and October 2016. Among 22 mothers who planned to exclusively breastfeed for six months, 17 reported adding other food or fluids before six months, and five reported exclusively breastfeeding successfully for the first six months. Key themes showed that all mothers relied strongly on health workers’ infant feeding advice and support. All mothers experienced challenges regardless of whether they succeeded in EBF, including inappropriate advice from health workers, maternal-baby issues, pressure from family members and returning to school and work. However, those who were successful at EBF for six months reported that high breastfeeding self-efficacy, HIV status and cultural meaning attached to breastfeeding were underlying factors for success.”  Subheadings: 1) Barriers to exclusive breastfeeding for six months, 2) The health system, 3) Maternal-baby factors, 4) Pressure from family to add other foods or liquids, 5) Returning to work or school, 6) Enablers among mothers who successfully exclusively breastfeed for six months |
| Janeiro, L. d. B., Ribeiro, E. M., & Lopez Miguel, M. J. (2018). What is inside the “black box”? Therapeutic community residents’ perspectives on each treatment phase. Addiction Research & Theory, 26(4), 294-305.  Country: Portugal | "This study aims to describe residents’ perspectives on change through each of the three treatment phases The TC [therapeutic communication] residents’ perspectives on their change can contribute towards an understanding of this process of change. More specifically, we aimed to show how the residents perceived the contextual conditions of treatment during each phase, how they related to the perceived treatment and which outcomes they felt that they had attained." | Grounded theory | Population: Residents of therapeutic community (n=25)  Data: Individual interviews and questionnairs  Length of data collection: 18 months | “According to residents’ views, the study results suggest that their agency was the core factor in change, while treatment tasks and healing relationships established the context for change. During each phase, contextual factors acquired particular features, with the common factor being conduciveness to action by the residents themselves. Such agency enabled the residents to appropriate treatment resources across three phases. By the end of the treatment residents had made major changes to the way in which they relate to themselves and others and could envisage a new life in which drugs had no place.”  Subheadings: 1) First treatment phase, (Contextual factors, Residents’ perspectives on treatment tasks, Healing relationships, Residents’ action: Self-object, Outcomes), 2) Second treatment phase (Contextual factors, Residents’ perspectives on treatment tasks, Healing relationships, esidents’ acts: Self-action, Outcomes), 3) Third treatment phase (Contextual factors, Residents’ perspectives on treatment tasks, Healing relationships, Residents’ actions: Therapeutic-self, Outcomes) |
| Jarvie, R. (2017). Lived experiences of women with co-existing BMI≥30 and Gestational Diabetes Mellitus. Midwifery, 49, 79-86.  Country: UK | "The aim of the study was to explore the lived experiences of women with co-existing maternal obesity (BMI ≥ 30) and GDM [Gestational Diabetes Mellitus] during pregnancy and the post-birth period (< 3 months post-birth)." | A qualitative, sociological design | Population: Pregnant women having co-existing maternal obesity and Gestational Diabetes Mellitus (n=27)  Data: Individual interviews and reflective field notes  Length of data collection: 6 to 9 months | “Women were experiencing a number of social and economic stressors that compromised their ability to manage pregnancies complicated by maternal obesity and GDM [Gestational Diabetes Mellitus], and make lifestyle changes.Women perceived themselves to be stigmatised by healthcare professionals and the general public due to their obese and gestational diabetic status.”  Subheadings: 1) Social and economic stressors, 2) Stigma, |
| Jee, S. D., Schafheutle, E. I., & Noyce, P. R. (2017). Using longitudinal mixed methods to study the development of professional behaviours during pharmacy work-based training. Health & Social Care in the Community, 25(3), 975-986.  Country: UK | "the aim of this study was to explore factors associated with changes in professional behaviours of trainees longitudinally during pre-registration training in pharmacy" | mixed methods | Population: Trainees and their main supervisors in pharmacies (n=41)  Data: Individual interviews  Length of data collection: 4 months | “Self-assessed (trainee) and tutor ratings of all elements of professional behaviours measured in questionnaires (appearance, interpersonal/social skills, responsibility, communication skills) increased significantly from the start of pre-registration training to post-registration. Some elements, for example, communication skills, showed more change over time compared with others, such as appearance, and continued to improve post-registration. Qualitative findings highlighted the changing roles of trainees and learning experiences that appeared to facilitate the development of professional behaviours. Trainees’ colleagues, and particularly tutors, played an essential part in trainees’ development through offering support and role modelling. Trainees noted that they would have benefited from more responsibilities during training to ease the transition into practising as a responsible pharmacist.”  Subheadings: 1) Research participants, 2) Quantitative findings: professional behaviour questionnaires (Trainees and tutors: round 1 and round 3, Trainees only: rounds 1–4),  2) Qualitative findings (Appearance, Interpersonal/social skills, Responsibility, Communication skills) |
| Jensen, J. F., Overgaard, D., Bestle, M. H., Christensen, D. F., & Egerod, I. (2017). Towards a new orientation: A qualitative longitudinal study of an intensive care recovery programme. Journal of Clinical Nursing, 26(1), 77-90.  Country: Denmark | "The aim of the study was to describe the patient experience of ICU [intensive care unit] recovery from a longitudinal perspective by analysing follow-up consultations at three time-points." | Qualitative longitudinal research | Population: Patient at ICU (n=12)  Data: Audio-recordings of consultations, patient photographs, and reflection sheets  Length of data collection: 9 to 11 months (depending upon case) | “The basic narrative of recovery was ‘toward a trajectory of new orientation’. This narrative contained the chronological narratives of being ‘at death’s door’, ‘still not out of the woods’ and ‘on the road to recovery’. The road to recovery was described as downhill, steady-state or progressive. New orientation was obtained in steady-state or progressive recovery.”  Subheadings: Participant characteristics, 1) Patient photographs, 2) Main theme: Towards a trajectory of new orientation, 3) First consultation narrative: At death’s door, 4) Second consultation narrative: still not out of the woods, 5) Third consultation narrative: On the road to recovery |
| Jensen, A. M., Pedersen, B. D., Olsen, R. B., & Hounsgaard, L. (2019). Medication and care in Alzheimer's patients in the acute care setting: A qualitative analysis. Dementia, 18(6), 2173-2188.  Country: Denmark | "The aim of the study was to investigate how oral medicine was administered to hip fracture patients with Alzheimer’s disease during acute hospital admission on an orthopaedic ward." | Phenomenological and ethnogaphy | Population: Patients with an Alzheimer’s diagnosis (n=3)  Data: Observations and informal interviews  Length of data collection: 4 days to 2 weeks (varied between cases) | “The data showed that, despite having little specific knowledge about dementia care, staff were able to gather information about the patient’s needs and wishes relevant to administrating medicine in a person-centred way. However, much of this valuable information was lost between shifts or became hidden among the overwhelming amount of information in electronic patient records. As a consequence, much of the knowledge about individual patients’ needs and wishes had to be collected all over again at every shift.”  Subheadings: 1) Naıve reading, 2) Structural analysis (Concealed medication, Dialogue and engagement on medicine intake) |
| Jensen, A. M., Pedersen, B. D., Olsen, R. B., Wilson, R. L., & Hounsgaard, L. (first published 2018). "If only they could understand me!" Acute hospital care experiences of patients with Alzheimer's disease. Dementia, 2020, 19(7) 2332-2353.  Country: Denmark | "The aim of this study was to investigate the observed experiences of patients with Alzheimer’s of receiving care in an acute hospital setting. This paper investigates the care of patients with Alzheimer’s disease in an acute setting, as co-morbidity to a hip fracture, and focuses on patients’ experiences of care during admission." | Ethnography | Population: Patients with dementia receiving acute care (n=3)  Data: Observations and field notes  Length of data collection: 4 to 14 days | “The data were interpreted from a phenomenological-hermeneutic perspective, inspired by Ricoeur’s interpretation theory. The study revealed a communication style among nurses who failed to take into account the comprehensive needs of patients with dementia, in terms of timely information exchange and clinical relevance. Patients expressed the desire to be more fully engaged in the care decisionmaking, together with indicating their appreciation of the work of the health professionals who cared for them. The data revealed that the process of getting to know the patient at the beginning of every shift left little room to alleviate patients’ experienced distress, caused by being in hospital. This resulted in patients who were less involved in the caring situation, or, if a patient took the initiative to act, intentions ended up being misinterpreted as disruptive behaviour.”  Subheadings: 1) Naıve understanding, 2) Structural analysis (Involving me at my pace – A communication disruption, “But what can I do”?, Appreciation of the staff – but not necessarily the situation, Self-blame for being in this situation) |
| Jindal-Snape, D., Johnston, B., Pringle, J., Kelly, T. B., Scott, R., Gold, L., & Dempsey, R. (2019). Multiple and multidimensional life transitions in the context of life-limiting health conditions: Longitudinal study focussing on perspectives of young adults, families and professionals. BMC Palliative Care, 18(1), 30-30.  Country: UK | "(1) What multiple and multi-dimensional transitions are Young Adults [YA] experiencing due to their life-limiting health conditions and developmental stage? (2) What multiple and multi-dimensional transitions are significant others experiencing due to the life-limiting health conditions and developmental stage of the YAs?" | mixed methods & qualitative longitudinal research | Population: Young adults with life limiting disease, their significant others, and health-care providers (n=31)  Data: Individual interviews  Length of data collection: 6 months | “Life transitions of YA [young adults] and significant others are complex; they experience multiple and multi-dimensional transitions across several domains. The findings challenge the notion that all life transitions are triggered by health transitions of YAs, and has highlighted environmental factors (attitudinal and systemic) that can be changed to facilitate smoother transitions in various aspects of their lives.”  Subheadings: 1) Multiple transitions of YAs with life-limiting conditions: educational, social and developmental (RQ1) (Health and educational transitions, Health and social transitions), 2) Health and developmental transitions (Independence, Moving out, Child to adult services, Death and loss), 3) Multiple and multi-dimensional transitions of significant others: family (RQ2) (Diagnosis, Change in role and family relationships, Change in aspirations, social and educational transitions), 4) Multiple and multi-dimensional transitions of significant others: professionals (RQ3) |
| Johannessen, A., Engedal, K., Haugen, P. K., Dourado, M. C. N., & Thorsen, K. (2018). "To be, or not to be": Experiencing deterioration among people with young-onset dementia living alone. International Journal of Qualitative Studies on Health and Well-Being, 13(1), 1490620-1490620.  Country: Norway | "aiming to explore the existential experiences and coping of people with YOD (age at onset of dementia before 65 years of age) , as they narrate the deterioration due to dementia that they go through over time." | Grounded theory | Population: People with young onset dementia living alone (n=10)  Data: Individual interviews  Length of data collection: About 2 years | “The main theme is the person’s experiences of changes of identity over time. The most significant aspects of their experiences of the dementia affecting them and their reactions are these: the initial signs, coping efforts, concealing the diagnosis, social retraction, existential anxiety, revival of the self, worse and worse, and health personnel as background.”  Subheadings: 1) Changes of identity over time, 2) The initial signs, 3) Coping efforts, 4) Concealing the diagnosis, 5) Social retraction, 6) Existential anxiety, 7) Revival of the self |
| Johannessen, A., Engedal, K., Haugen, P. K., Dourado, M. C. N., & Thorsen, K. (2019). Coping with transitions in life: A four-year longitudinal narrative study of single younger people with dementia. Journal of Multidisciplinary Healthcare, 12, 479-492.  Country: Norway | "Therefore, we have performed a longitudinal study aiming to explore how people living alone with YOD [young people with dementia] experiences of coping with transitions during the progression of dementia." | Qualitative longitudinal research | Population: People living alone with younger onset dementia (n=10)  Data: Individual interviews  Length of data collection: Up to 48 months | “Two significant main transitions and themes were registered under the perspective; experiencing and coping with (1) receiving the diagnosis of dementia and (2) moving to a residential care facility, which covers two subthemes: moving to a supported living accommodation and moving to a nursing home. To get the diagnosis was initially experienced as a dramatic disaster, while moving to residential care were mainly experienced as positive. With efficient cognitive and emotion-focused coping strategies, the participants adapted and experienced a mostly good life for a long time.”  Subheadings: 1) Receiving the diagnosis of dementia, 2) Moving to a residential care facility (Moving to a supported living accommodation), 3) Moving to a nursing home |
| Johansen, F., Loorbach, D., & Stoopendaal, A. (2018). Exploring a transition in Dutch healthcare. Journal Of Health Organization And Management, 32(7), 875-890.  Country: the Netherlands | "The purpose of this paper is to explore the contours of this transition in the Netherlands." | other | Population: Health care organisations/ project managers (n=22)  Data: Individual interviews  Length of data collection: 3 years | “The process validated the initial understanding of persistent sustainability challenges. An integral approach to sustainable healthcare is translated as a transformation of culture, structures and practices and the development of capacity for crossing borders and domains, inside and outside of the organisation. To facilitate and stimulate such a process the authors found that problem structuring and collective identification of persistent problems and the unsustainability in the healthcare system is a crucial step towards a shared view and discourse that supports change.”  Subheadings: 1) Rewards, 2) Positive health, 3) Changes in structure, culture and practices, 4) Challenges, 5) Frontrunners in the making? |
| Jones, K. F., Dorsett, P., Simpson, G., & Briggs, L. (2018). Moving forward on the journey: Spirituality and family resilience after spinal cord injury. Rehabilitation Psychology, 63(4), 521-531.  Country: Australia | "The aim of this exploratory study was to consider how spirituality (encompassing meaning, hope and purpose), may facilitate family resilience after SCI [spinal cord injury] over time." | Phenomenology | Population: Individuals with SCI, and family members (n=20)  Data: Joint interviews  Length of data collection: 6 months | “Participants reported drawing upon a range of different sources of spirituality, including religious faith, the natural world, inner strength, and meaningful connectedness with others. These sources of spirituality were often tested in some way after the SCI [spinal cord injury]. Meaning-making responses to these tests were linked with 3 key outcomes: gratitude, hope, and deeper connectedness with others, assisting families to move forward in their journey after SCI. Over time sources of spirituality did not change significantly; however, the intensity of spiritual experience lessened for some families.”  Subheadings: Sample description, 1) Theme 1: Sources of spirituality (Religious faith, The natural world, An inner strength, Meaningful connectedness with others) 2) Theme 2: Responses to testing of sources of spirituality (Religious faith tested, Inner strength tested, Connectedness with others tested), 3) Theme 3: Key outcomes of testing process (Gratitude, A “deepening of all of our relationships”), 4) Theme 4: Moving forward on the journey, 5) Changes in Spiritual Intensity Over Time |
| Kerrissey, M., Satterstrom, P., Leydon, N., Schiff, G., & Singer, S. (2017). Integrating: A managerial practice that enables implementation in fragmented health care environments. Health Care Management Review, 42(3), 213-225.  Country: US | "Our uniquely granular longitudinal data from 16 primary care clinics enable us to deeply explore the mechanisms that frontline workers use to overcome implementation barriers." | case study | Population: Frontline workers at primary care clinics (16 clinics)  Data: Individual interviews, field notes from observations, and documents (e.g., meeting minutes)  Length of data collection: 15 months | “Nine clinics implementedmore successfully over the study period, whereas seven implemented less. Successfully implementing clinics exhibited the managerial practice of integrating, which we define as achieving unity of effort among stakeholder groups in the pursuit of a shared and mutually developed goal. We theorize that integrating is critical in improvement implementation because of the fragmentation observed in health care settings, and weextend theory about clinic managers role in implementation.We identify four integrating mechanisms that clinic managers enacted: engaging groups, bridging communication, sense making, and negotiating. The mean patient survey results for integrating clinics improved by 0.07 units over time, whereas the other clinics - survey scores declined by 0.08 units on a scale of 5 (p =.02).”  Subheadings: 1) Variation in implementation, 2) Many factors affecting implementation, 3) Integrating factors as key for successfully implementing clinics, 4) Clinic managers as integrators, 5) Importance of reciprocal relationships, 6) Other factors did not differentiate clinics that implemented more, 7) Integrating physicians, 8) Integrating clinic staff, 8) Integrating patients, 10) Integrating external partners, 12) Relationship between integrating and performance |
| Kirshbaum, M. N., Ennis, G., Waheed, N., & Carter, F. (2017). Art in cancer care: Exploring the role of visual art-making programs within an Energy Restoration Framework. European Journal Of Oncology Nursing, 29, 71-78.  Country: Australia | "The aim of the study was to explore the experience of participation in a visual art-making program for people during or after cancer treatment in the Northern Territory of Australia, using a framework for energy restoration. The objectives of the study were: 1. To set up and facilitate an eight-week group experiential visual art-making program for people who have been diagnosed with cancer. 2. To document and analyse the participants' experiences of the arts program. 3. To interpret the findings using the Energy Restoration Framework." | Qualitative longitudinal research | Population: People who have been diagnosed with cancer (n=8)  Data: individual interviews and group discussions  Length of data collection: 8 weeks | “The four a priori themes were retained and an additional attribute of an energy restoration activity called Stimulating was added, along with sub-themes, which broadened and deepened understanding of the art-making experience within cancer care.”  Subheadings: 1) Participants and workshops, 2) Themes, 3) Expansive, 4) Belonging, 5) Nurturing, 6) Purposeful, 7) Stimulating |
| Klinga, C., Hasson, H., Andreen Sachs, M., & Hansson, J. (2018). Understanding the dynamics of sustainable change: A 20-year case study of integrated health and social care. BMC Health Services Research, 18(1), 400-400.  Country: Sweden | "The specific objective of this study is to gain insight into the dynamics of sustainable changes in integrated health and social care through an analysis of local actions that were trigged by a national policy." | Case study | Population: Minutes of the model organization's steering-committee  Data: Documents ( meeting minutes and notes)  Length of data collection: 20 years | “The development of inter-sectoral cooperation was characterized by a participatory approach in which a shared structure was created to support cooperation and on-going quality improvement and learning based on the needs of the service user. A key management principle was cooperation, not only on all organisational levels, but also with service users, stakeholder associations and other partner organisations. It was shown that all these parts were interrelated and collectively contributed to the creation of a structure and a culture which supported the development of a dynamic sustainable health and social care.”  Subheadings: 1) Case findings, 2) Empirical themes (Shared structure and ongoing refinement, Continuous learning, Cooperation as a guiding principle for management, Service user centeredness) |
| Kowalski, C. P., McQuillan, D. B., Chawla, N., Lyles, C., Altschuler, A., Uratsu, C. S., Bayliss, E. A., Heisler, M., & Grant, R. W. (2018). 'The hand on the doorknob': Visit agenda setting by complex patients and their primary care physicians. Journal of the American Board of Family Medicine, 31(1), 29-37.  Country: US | "to investigate how patients and physicians prepare for visits, how visit agendas are determined, and how discussion priorities are established during time-limited visits. 1) examine how patients and physicians prepared for upcoming visits; 2) gain further insight into how agendas are set during visits; and 3) identify factors that facilitate or impede alignment of visit agendas." | Qualitative longitudinal research | Population: Patients and their primary care physicians (n=57)  Data: Individual interviews  Length of data collection: Unclear, a few weeks | “Visit flow and alignment were enhanced when both patients and physicians were effectively prepared before the visit, when the patient brought up highest-priority items first, the physician and patient worked together at the beginning of the visit to establish the visit agenda, and other team members contributed to agenda setting. A range of factors were identified that undermined the ability of patient and physicians to establish an efficient working agenda: the most prominent were time pressure and short visit lengths, but also included differing visit expectations, patient hesitancy to bring up embarrassing concerns, electronic medical record/documentation requirements, differences balancing current symptoms versus future medical risk, nonactionable items, differing philosophies about medications and lifestyle interventions, and difficulty by patients in prioritizing their top concerns.”  Subheadings: 1) Patient and physician preparation for upcoming visits, 2) Insights into agenda setting, 3) Factors that facilitate or impede alignment of visit agendas |
| Križaj, T., Warren, A., & Slade, A. (2018). "Holding on to what I do": Experiences of older slovenians moving into a care home. Gerontologist, 58(3), 512-520.  Country: Slovenia | "...to explore Slovenian older people’s experiences of transition into a care home and how it influenced their everyday engagement in meaningful occupations" | Phenomenology | Population: Older people (n=6)  Data: Individual interviews  Length of data collection: 6 months | “One overarching theme from the research findings Holding on to what I do is presented in depth. Although the participants’ everyday occupations were challenged throughout the transition, they tried to maintain their most meaningful occupations that were an important part of their identity. Three superordinate themes underpinned this overarching theme: This is who I am, Adjusting my daily occupations, and The value of health.”  Subheadings: 1) Holding on to what I do, 2) This is who I am, 3) Ajusting my daily occupations, 4) The value of health |
| Laerkner, E., Egerod, I., Olesen, F., & Hansen, H. P. (2017). A sense of agency: An ethnographic exploration of being awake during mechanical ventilation in the intensive care unit. International Journal of Nursing Studies, 75, 1-9.  Country: Denmark | "The study aimed to explore patients’ experiences of being awake during critical illness and mechanical ventilation in the ICU {intensive care unit]." | other | Population: Patients’ being awake during critical illness and mechanical ventilation in the ICU (n=28)  Data: Individual interviews and observations  Length of data collection: 2 to 4 months (differ between participants) | “Three themes were identified: ‘A sense of agency’, ‘The familiar in the unfamiliar situation’ and ‘Awareness of surrounding activities’. Patients had the ability to interact from the first days of critical illness and a sense of agency was expressed through initiating, directing and participating in communication and other activities. Patients appreciated competent and compassionate nurses who were attentive and involved them as individual persons. Initiatives to enhance familiar aspects such as relatives, personal items and care, continuity and closeness of nurses contributed to the patients' experience of feeling safe and secure in the unfamiliar setting. Patients were aware of the surrounding activities and felt powerless when ignored by the staff and were affected when witnessing fellow patients’ suffering.”  Subheadings: 1) A sense of agency, 2) The familiar in the unfamiliar situation, 3) Awareness of surrounding activities |
| Lagsten, J., & Andersson, A. (2018). Use of information systems in social work - challenges and an agenda for future research. European Journal of Social Work, 21(6), 850-862.  Country: Sweden | "The aim of this paper is therefore to contribute to the emerging social work information systems research agenda. Research questions: What are the challenges in the use of a social work information system in a Swedish social work agency according to the stakeholders? How do the empirically grounded challenges relate to the literature on the use of social work information systems? What are the important areas for further study in the evolving social work information systems research agenda?" | Case study | Population: Social workers (n=70)  Data: Individual interviews, dialogue seminars, document, and observations  Length of data collection: 10 years | “Comparing the evaluation findings with the literature helped us identify six areas in need of intensified research: (i) Usability and interface design; (ii) Mismatch between social conceptualisation and system conceptualisation of the case; (iii) Skills and training for information systems use; (iv) Statistical production for accountability and quality assurance; (v) Terminology for interpersonal understanding; (vi) IT Governance. As can be seen from the areas above, this kind of research depends on professional knowledge from the social work field – but also from the field of information systems.”  Subheadings: 1) Evaluation results on system use and challenges (Social workers, Managers, IT support, IT managers, Follow-up on evaluation results) |
| Lahav, O., Daniely, N., & Yalon-Chamovitz, S. (2018). Interpersonal social responsibility model of service learning: A longitudinal study. Scandinavian Journal of Occupational Therapy, 25(1), 61-69.  Country: Israel | "The objective of this longitudinal study was to explore a structured model of Service-Learning (ISRSL), aimed towards the development of professional identity among OT [occupational therapy] students. In order to move beyond description a grounded theory approach was utilized to explore the perceptions and experiences of OT students who participated and experienced firsthand ISR-SL during their first academic year." | Grounded theory | Population: Occupational therapy students (n=105)  Data: Open-ended questions in questionnairs  Length of data collection: 5 years | “Our findings suggest that the structured, long-term relationship with a person with a disability in the natural environment, which is the core of the ISR-SL [structured model of Service-Learning], allowed students to develop a professional identity based on seeing the person as a whole and recognizing his/her centrality in the therapeutic relationship.”  Subheadings: 1) Primary experience (Primary experience with unknown populations, Primary experience communicating with a person with a disability), 2) The mentoring process and its contribution to the student (Group mentoring, Professional mentoring in the field, Mentoring a person with a disability in his/her natural environment), 3) Professional identity (Developing client centered approach, Developing therapeutic personality, Developing awareness of oneself as a future occupational therapist), 4) The interaction between the course and the overall curriculum (Timing of the course, Nature of the course) |
| Lang, H., France, E. F., Williams, B., Humphris, G., & Wells, M. (2018). The existence and importance of patients' mental images of their head and neck cancer: A qualitative study. Plos One, 13(12), e0209215-e0209215.  Country: UK Scotland | "The aim of our study was to explore in detail the existence and importance of mental images of cancer among people with HNC in terms of the perceived origins and meaning of mental images, their development over time, and the relationship of their mental images to illness beliefs." | Phenomenology & qualitative longitudinal research | Population: People with consecutive, newly-diagnosed head and neck cancer (n=25)  Data: individual interviews, and images/ drawings  Length of data collection: 3 to 9.5 months (avarage 5 months) | “Many participants had mental images of their cancer which appeared to both embody and influence their beliefs about their illness, and affect their emotional response. For those who held them, mental images appeared to constitute an important part of their cognitive representation (understanding) of their illness. For some, their images also had a powerful emotional impact, being either reassuring or frightening. Images often appeared to originate from early clinical encounters, and remained fairly stable throughout treatment. Images could be conceptualised as ‘concrete’ (the perceived reality) and/or ‘similic’ (figurative). Patients’ images reflected the perceived meaning, properties or ‘intent’ of the cancer–that is beliefs concerning the disease’s identity, consequences and prognosis (likelihood of cure or control).”  Subheadings: 1) Existence and nature of mental images of HNC, 2) Concrete versus similic images, 3) Mental images of the appearance and characteristics of cancer, 4) Mental images of how cancer behaves, 5) Origins and impact of visual communications |
| Larsen, S. M., Hounsgaard, L., Brandt, Å., & Kristensen, H. K. (2019). "Becoming acquainted": The process of incorporating assistive technology into occupations. Journal of Occupational Science, 26(1), 77-86.  Country: Denmark | "The purpose of this study was, therefore, to investigate older adults’ experiences of the process of incorporating Ats [Assistive Technology] into occupations." | Qualitative longitudinal research and phenomenology | Population: Older adults living in their usual home and who had applied to the municipality for an AT (n=8)  Data: Individual interviews and observations  Length of data collection: Up to 10 months, most participants were followed 1,5 months | “Through the analysis, one main theme, ‘Becoming acquainted’ and six related subthemes, ‘Acquaintance through use’, ‘Drawing on previous experiences’, ‘Engaging in valued occupations’, ‘Encounters with others’, ‘Regaining control over everyday life’ and ‘Challenges in using the AT’ were identified.”  Subheadings: 1) Becoming acquainted, 2) Acquaintance through use, 3) Drawing on previous experiences, 4) Engaging in personally valued occupations, 5) Encounters with others, 6) Regaining control over everyday life, 7) Challenges in using the AT |
| Laur, C., Bell, J., Valaitis, R., Ray, S., & Keller, H. (2018). The Sustain and Spread Framework: Strategies for sustaining and spreading nutrition care improvements in acute care based on thematic analysis from the More-2-Eat study. BMC Health Services Research, 18(1), 930-930.  Country: Canada | "The aim of this manuscript is to develop a potential framework of strategies to sustain and spread the successful implementation of INPAC [nutrition care improvements in acute care]." | not described | Population: Staff and management at five hospital sites (n=138 participants)  Data: Individual interviews, Focus group interviews, and informal group discussions  Length of data collection: 14 months | “After implementation, sites described a culture change with respect to nutrition care, where new activities were viewed as the expected norm and best practice. Strategies to sustain changes included: maintaining the new routine; building intrinsic motivation; continuing to collect and report data; and engaging new staff and management. Strategies to spread included: being responsive to opportunities; considering local context and readiness; and making it easy to spread. Strategies that supported both sustaining and spreading included: being and staying visible; and maintaining roles and supporting new champions.”  Subheadings: 1) Sustain (Maintaining the new routine, Engaging new staff and management, Building intrinsic motivation, Continue to measure and report), 2) Spread, (Being responsive to opportunities, Considering local context and readiness, Making it easy to spread), 3) Connecting spread to implementation, 4) Sustain and spread (Being and staying visible, Maintaining roles and supporting new champions), 5) Creating culture change |
| Lawton, J., Blackburn, M., Rankin, D., Allen, J. M., Campbell, F. M., Leelarathna, L., Tauschmann, M., Thabit, H., Wilinska, M.E., Elleri, D., & Hovorka, R. on behalf of the APCam11 Consortium. (2019). Participants' experiences of, and views about, daytime use of a day-and-night hybrid closed-loop system in real life settings: Longitudinal qualitative study. Diabetes Technology & Therapeutics, 21(3), 119-127.  Country: UK | "To address these objectives, we report findings from interviews undertaken with individuals who used a hybrid day-and-night closed-loop system combined with pump suspend feature over 3 months. Given that studies have overwhelmingly focused upon nighttime use, we focus our reporting on people’s experiences of using the closed-loop system during the day." | Qualitative longitudinal research | Population: Individuals who used a hybrid day-and-night closed-loop system for diabetes treatment including the adolentce participants parents (n=24)  Data: Individual interviews  Length of data collection: 3 months | “Participants praied the closed loop’s ability to respond to high and low blood glucose in ways which extended beyond their own capabilities and to act as a safety net and mop up errors, such as when a mealtime bolus was forgotten or unplanned activity was undertaken. Participants also described feeling less burdened by diabetes as a consequence and more able to lead flexible, spontaneous lives. Contrary to their initial expectations, and after trust in the system had been established, most individuals wanted opportunities to collaborate with the closed loop to optimize its effectiveness. Such individuals expressed a need to communicate information, such as when routines changed or to indicate different intensities of physical activity. While individuals valued frequent contact with staff in the initial month of use, most felt that their long-term support needs would be no greater than when using an insulin pump.”  Subheadings: 1) Initial expectations (baseline interviews) (Probationary period), 2) Using the closed-loop system (follow-up interviews) (Developing trust and confidence in the system), 3) Clinical and quality-of-life benefits (Lessening the burden of self-management, Better blood glucose control, Reassurance and less worry, A more flexible and active life), 3) Working in partnership with the closed loop (Collaborating with the closed loop to optimize blood glucose control, Wanting opportunities to communicate information to the closed loop), 4) Views about education and training and need for staff support (Training and support needs during the 3-month trial, Longer-term support needs) |
| Lawton, J., Blackburn, M., Breckenridge, J. P., Hallowell, N., Farrington, C., & Rankin, D. (2019). Ambassadors of hope, research pioneers and agents of change-individuals' expectations and experiences of taking part in a randomised trial of an innovative health technology: Longitudinal qualitative study. Trials, 20(1), 289-289.  Country: UK | "As part of this investigation, we explored people’s reasons for taking part in the trial as well as their actual experiences of using the closed-loop system during the trial. As we describe in this paper, the findings from this aspect of our study not only prompted us to problematise use of dichotomous categories such as ‘self-interest’ and ‘altruism’, but also to contribute literature on clinical trials by considering how participants’ understandings of the trial and their complex and interweaving agendas for taking part could have profound implications for their conduct during the trial." | Qualitative longitudinal research | Population: Patients and family members (participants in a diabetes trial and parents to some participants that were 13-17 yrs old (n=24)  Data: Individual interviews  Length of data collection: 3 months | “Participants provided complex agendas for taking part in which altruistic and self-interested considerations were often inseparable. Many described belonging to a wider diabetes community and being beneficiaries of others’ participation in research and how this had given rise to attendant citizenship obligations. Participants also shared the excitement and pride they experienced from contributing to research which situated them at the forefront of technological innovation and enabled them to present themselves to others, by virtue of their trial participation, as ambassadors of hope and research pioneers. Given their desire to support the progression of a potentially life-changing technology, and be part of that innovation, participants, at follow-up, described having made extra effort during the trial. Specifically, participants described having been more focused on their diabetes management to help create conditions in which the closed-loop could work most effectively to optimize their blood glucose control.”  Subheadings: 1) Self-interested agendas: therapeutic appropriation and incidental benefits, 2) Altruistic agendas: helping others, 3) Interweaving and inseparable agendas: research pioneers and ambassadors of hope, 4) Agents of change, 5) Impact of motivations and agendas on the participants’ conduct during the trial, |
| Lea, J., & Cruickshank, M. (2017). The role of rural nurse managers in supporting new graduate nurses in rural practice. Journal of Nursing Management, 25(3), 176-183.  Country: Australia | "This paper reports on findings specifically related to the unique role rural nurse managers (NMs) and nurse unit managers (NUMs) play in supporting new graduate nurses transitioning to rural nursing practice." | case study | Population: Nurses and nurse units managers (n=30)  Data: Individual interviews  Length of data collection: 9 months | “Graduates in rural health services rely on nurse unit managers and nurse managers for feedback, support and debriefing, provision of emotional support, advocacy, openness, encouragement and protection from organisational requests and demands during the transition to rural nursing practice.”  Subheadings: 1) Stage one: 3-month milestone – NUM visibility and disconnect, 2) Stage two: 6-month milestone – need for acknowledgement and interaction from the NM/ NUM, 3) Stage three: 9-month milestone – the need for feedback from the NM or NUM |
| Lees, S., Marchant, M., & Desmond, N. (first published 2019). Addressing intimate partner violence using gender-transformative approaches at a community level in rural Tanzania: The UZIKWASA program. Journal of Interpersonal Violence, 2021, 36(13-14), NP7791-NP7812.  Country: Tanzania | "Drawing on both innovative and traditional qualitative research methods, this article aims to explore the ways in which UZIKWASA’s [UZIKWASA is a civil society organization] interventions affect attitudes and norms surrounding violence against women and girls." | Ethnography | Population: People in the community (n=10)  Data: Hearsay ethnographies (e.g., dairies), individual interviews, and focus group interviews  Length of data collection: 5 years | “The findings reveal personal and community narratives about gender-based and IPV as forms of retributive justice and assertion of authority by men. Drawing on gender performance as an explanation for violence, the research revealed changes in norms and practice in relation to violence against women and girls.”  Subheadings: 1) Community Understanding of Violence Against Women (Hearsay: Gender-based violence as retributive justice, IPV as assertion of authority, Gendered explanations of violence against women), 2) UZIKWASA and Pangani FM: Evidence of Gender-Transformative Change (Perceived reduction of violence against women and girls in Pangani, Improved support for victims of violence) |
| Leibring, I., & Anderzén-Carlsson, A. (2019). Fear and coping in children 5–9 years old treated for acute lymphoblastic leukemia - A longitudinal interview study. Journal of Pediatric Nursing, 46, e29-e36.  Country: Sweden | "The aim of this study was to use a longitudinal perspective in a group of 5- to 9-year-old children with ALL [Acute lymphoblastic leukemia] to describe their ALL-related fears, the strategies they use to cope with those fears, and changes in their fears and strategies over time. 1. What fears do children with ALL experience at various time points during their treatment? 2. How do the children cope with their fears?" | Qualitative longitudinal research | Population: 5- to 9-year-old children with Acute lymphoblastic leukemia (n=13)  Data: Individual interviews  Length of data collection: About 2.5 years | “Initially, most children reported a fear of needles, but during the treatment period, fewer children reported this fear. Children's coping strategies also changed over time, as they wantedmore involvement and control during needle-related procedures. Other fears were having adhesive tapes removed, having a nasogastric tube, and taking tablets. During the treatment period, existential fears related to the seriousness of ALL [Acute lymphoblastic leukemia] and its consequences, such as having impairedphysical fitness andbeing different frombefore and different fromothers, became more prominent and caused feelings of loneliness and alienation.”  Subheadings: 1) Fear of needles, 2) Fear of having a feeding tube, 3) Fear of removing adhesive tape, 4) Fear of taking tablets, 5) Fear of the physical changes caused by ALL |
| Lennon, M. R., Bouamrane, M.-M., Devlin, A. M., O'Connor, S., O'Donnell, C., Chetty, U., Agbakoba, R., Bikker, A., Grieve, E., Finch, T., Watson, N., Wyke, S., & Mair, F. S., (2017). Readiness for delivering digital health at scale: Lessons from a longitudinal qualitative evaluation of a national digital health innovation program in the United Kingdom. Journal Of Medical Internet Research, 19(2), e42-e42.  Country: UK | "The aim of our study was to examine barriers and facilitators to implementation of digital health at scale through the evaluation of a £37m national digital health program: Delivering Assisted Living Lifestyles at Scale” (dallas) from 2012-2015. "The aim of this study was to capture barriers and facilitators to implementation of digital health across a wide range of stakeholders and across time, thus allowing us to answer the question of how ready” different people, processes, and systems are for mainstreaming digital health and to identify what measures might be taken to reduce some of the existing and persistent barriers in this area. Here we present our findings and conclude with a set of 10 recommendations to address some of the key readiness barriers identified." | Qualitative longitudinal research | Population: Health-care providers, patients and managers (key implementors, managers, patients, health-care providers of different professions  Data: Individual interviews, focus group interviews, questionnairs, documents, observations and field notes  Length of data collection: 3 years | “We identified three main levels of issues influencing readiness for digital health: macro (market, infrastructure, policy), meso (organizational), and micro (professional or public). Factors hindering implementation included: lack of information technology (IT) infrastructure, uncertainty around information governance, lack of incentives to prioritize interoperability, lack of precedence on accountability within the commercial sector, and a market perceived as difficult to navigate. Factors enabling implementation were: clinical endorsement, champions who promoted digital health, and public and professional willingness.”  Subheadings: 1) Analysis and coding scheme, 2) Macro-level readiness (Market readiness, Interoperability, Risk and liability, Clinical endorsement, Complexity of the market, Political readiness and national policy, Infrastructure), 2) Meso-level readiness (Industry readiness, Lack of market coherence, Collaboration, ompetition, and codesign, Health service readiness or information technology (it) infrastructure, Discontinuity and organizational culture, Resource constraints), 3) Micro-level readiness (Health professional readiness: workload and professional confidence,Training and alignment with professional roles and identities, Access to digital resources, Public readiness: digital literacy and access, Agency of individuals and their perceptions of consumer” Digital health tools, Trust in consumer-facing, Digital technologies) |
| Lerret, S. M., Johnson, N. L., & Haglund, K. A. (2017). Parents' perspectives on caring for children after solid organ transplant. Journal for Specialists in Pediatric Nursing, 22(3), e12178.  Country: US | "The purpose of this study was to explore parents’ perspectives on the discharge transition from acute hospitalization following SOT [solid organ transplant] to long-term management of a complex chronic condition. The time frame for transition to chronic condition care was defined as the first 6 months at home following SOT. The parents’ perspective may be used to inform discharge care and teaching in order to help providers anticipate issues that children and families may experience during the first 6 months after hospital discharge." | mixed methods | Population: Parents’ to children with solid organ transplant (n=48)  Data: Individual interviews  Length of data collection: 6 months | “Analysis of parent interviews (N = 48) resulted in three themes that characterized the phases of transition to home and complex chronic illness care. Three themes, corresponding to the three time periods of data collection, included ‘getting back to normal’ at 3 weeks, ‘becoming routine’ at 3 months, and ‘facing a future’ at 6 months. Challenges families experienced over the course of their transition are also described.”  Subheadings: 1) Theme 1: “Getting back to normal”, 2) Theme 2: “Becoming routine”, 3) Theme 3: “Facing a future” |
| Lewis, M., Jones, A., & Hunter, B. (2017). Women's experience of trust within the midwife-Mother relationship. International Journal of Childbirth, 7(1), 40-52.  Country: UK | "The broad aim of the research study was therefore to explore the concept of trust from the individual woman’s perspective with a view to developing a better understanding of trust within the midwife–mother relationship." | phenomenology | Population: Women (n=9)  Data: Individual interviews  Length of data collection: 7 to 8 months | “The experience of trust was described as an evolving concept that developed over time as a series of building blocks. The participants described an initial trust associated with an expectation of assumed competence in the midwife, but this was then influenced by the developing relationship between midwife and mother. The concept of trust was interwoven with women’s agency; women expressed a desire to develop a two-way trust that included the midwife trusting the woman. This article reports on the overall findings, concentrating on the development of trust and key themes relevant to clinical midwifery practice: need, expectation, the midwife–mother relationship, and impact of continuity of carer and the importance of women’s agency.”  Subheadings: 1) Antecedents, 2) Attribute, 3) Consequences |
| Liang, P., Fleming, J., Gustafsson, L., & Liddle, J. (2017). Occupational experience of caregiving during driving disruption following an acquired brain injury. British Journal of Occupational Therapy, 80(1), 30-38.  Country: Australia | "Therefore, the aim of this study is to explore, using a phenomenological approach, family members’ lived experiences of the occupations they take on during driving disruption following ABI [acute brain injury]." | phenomenology | Population: Family members to a person who have a brain injury (n=15)  Data: Individual interviews  Length of data collection: 6 months | “The occupational experiences related to caregiving during driving disruption emerged as a key finding. The meaning and activities comprising the caregiving occupation during driving disruption are captured in three themes: (1) More than just driving; (2) The invisible and undervalued care and (3) Being a therapist at home. Family members highlighted the challenges of managing broader and multiple responsibilities.”  Subheadings: 1) More than just driving, 2) The invisible and undervalued care, 3) Being a therapist at home |
| Liang, P., Gustafsson, L., Liddle, J., & Fleming, J. (2017). Family members’ needs and experiences of driving disruption over time following an acquired brain injury: An evolving issue. Disability & Rehabilitation, 39(14), 1398-1407.  Country: Australia | "Therefore, the aim of this study is to explore the family members’ needs and experiences of driving disruption of persons with ABI [acute brain injury] over time." | phenomenology & qualitative longitudinal research | Population: Family members to a person who have a brain injury (n=14)  Data: Individual interviews  Length of data collection: 6 months | “Fourteen family members completed 41 interviews. The longitudinal data revealed four phases of driving disruption: (1) Wait and see, (2) Holding onto a quick fix, (3) No way out, and (4) Resolution and adjustment. The phases described a process of building tension and a need for support and resolution over time.”  Subheadings: 1) Wait and see, 2) Holding onto a quick fix, 3) No way out, 4) Resolution and adjustment |
| Liddle, M. J., Baker, S. R., Smith, K. G., & Thompson, A. R. (2018). Young adults' experience of appearance-altering orthognathic surgery: A longitudinal interpretative phenomenologic analysis. The Cleft Palate-Craniofacial Journal, 55(2), 238-247.  Country: UK | "The objective was to gain an experiential account of the processes of change associated specifically with orthognathic surgery in a way that might illuminate the psychological issues involved." | Phenomenology | Population: Patients aged 16-25 years who were scheduled to undergo orthognatic surgery (n=7)  Data: Individual interviews  Length of data collection: 6 to 8 weeks | “Themes were identified in connection with the overall journey of treatment being a rite of passage; the treatment’s role in raising awareness about the anomalies in appearance; the initial shock at the changes that followed surgery; the uncertainty about treatment; the impact of actual negative reactions of others; and the role of significant others in the decision-making process.”  Subheadings: 1) The Journey of treatment—a rite of passage, 2) The course of treatment and appearance sensitivity, 3) The initial shock at the changes that followed surgery, 4) Uncertainty and never being prepared, 5) Impact of actual negative reactions of others, 6) Influences of significant others on decision making |
| Lindberg, K., Mørk, B. E., & Walter, L. (2019). Emergent coordination and situated learning in a Hybrid OR: The mixed blessing of using radiation. Social Science & Medicine, 228, 232-239.  Country: Sweden | "Drawing upon a longitudinal, qualitative study of a Hybrid Operating Room in Sweden, we illustrate how the staff from a variety of medical specialties need to coordinate their tasks and competencies, and learn how to use the technology in a safe way." (from abstract) | Ethnography & qualitative longitudinal research | Population: Nurses and doctors working in the Hybrid OR, but also with hospital technicians, physicists and representatives of the suppliers of the technology  Data: Observations and shadowing, individual interviews, and documents  Length of data collection: 4 years | “This study shows that learning across highly-professionalized communities is a recursive process of emergent coordination and situated learning, which includes the acknowledgement of others’ expertise, task interdependence, and the pragmatic accommodation of latitude and control. Moreover, there was continuous negotiations between the different communities about what should constitute approved practice based on the task being performed. This obstructed the development of a dominant community with the authority to independently exclude other communities.”  Subheadings: 1) Learning how to use the imaging system in the Hybrid OR (Learning about radiation and sharing space, Learning the imaging equipment: technological instructions and professional judgement, Radiation in practice: interdependencies and learning in situ) |
| Lindberg, K., Walter, L., & Raviola, E. (2017). Performing boundary work: The emergence of a new practice in a hybrid operating room. Social Science & Medicine (1982), 182, 81-88.  Country: Sweden | "In this paper, we investigate the boundary work performed in relation to the boundaries in a setting where a new practice is emerging in the midst of other established practices. Rather than analyzing such boundary work as a rhetorical style, like Gieryn, we use Akrich and Latour, (1992) scripting processes as our analytical lens for exploring how boundary work is performed in practice." | Qualitative longitudinal research | Population: Surgical nurses, anastesia nurses, surgigal healthcare assistents, radiology nurses  Data: Observations, individual interviews and documents  Length of data collection: About 2.5 years | “The study shows how the visibility of boundaries is a result of as well as a condition for boundary work, how boundary work is a dynamic and iterative process, and how it unfolds in a recursive relationship between practice and boundaries.”  Subheadings: 1) Initiating boundary work: negotiating perscriptions towards a common subscription, 2) Expanding boundary work: perscribing and conscribing to the emerging practice, 3) Stabilizing boundary work: the inscription of the hybrid practice |
| Lut, I., Evangeli, M., & Ely, A. (2017). “When I went to camp, it made me free”: A longitudinal qualitative study of a residential intervention for adolescents living with HIV in the UK. Children & Youth Services Review, 79, 426-431.  Country: UK | "This UK study presents a qualitative investigation of young peoples' experience of a residential support camp conducted both at the end of the intervention and six months after, using representative sampling methods. It aimed to answer what the experiences and perceived consequences of attending camp were over time." | Qualitative longitudinal research | Population: Adolescents living with HIV (n=11)  Data: Individual interviews  Length of data collection: 6 months | “Six main themes were identified: connecting with new friends and feeling less alone; gaining HIV knowledge and learning about living with HIV; developing a positive self-image; communicating more purposefully; becoming more autonomous; and a desire to engage further with the HIV community. The majority of these themes were reported both at the six week and six month follow-up points. Participants felt that the intervention had increased their confidence, decreased their anxiety about sharing their HIV status and widened their support network.”  Subheadings: 1) Connecting with new friends and feeling less alone, 2) Gaining HIV knowledge and learning about living with HIV, 3) Developing positive self-image and prospects for the future, 4) Communicating more purposefully, 5) Becoming more autonomous, 6) Feeling motivated to continue engaging with the HIV community |
| Malin, H., Liauw, I., & Damon, W. (2017). Purpose and character development in early adolescence. Journal of Youth & Adolescence, 46(6), 1200-1215.  Country: US | "The goal of this study was to qualitatively describe how early adolescents show purpose—in what aspects of life and through what types of actions do they pursue purpose? We further sought to describe the relationship that purpose has to other character strengths (gratitude, compassion, and grit) at this early stage of development, to better understand whether they share a developmental trajectory and how they might differ early in development. Moreover, we wanted to explore the possible developmental interactions among the character strengths. For example, does compassion promote purpose development? Does purpose support grit? Our analysis cannot fully answer these developmental questions, but sets the course for further research by qualitatively exploring these questions: What does purpose look like in early adolescence? How is purpose similar to, and different from, other related character strengths? And, how does purpose interact with other character strengths in early adolescence?" | not described | Population: Early adolescents (98 participants interviewed, and 1.366 students completed the questionnairs)  Data: Individual interviews and questionnairs  Length of data collection: About a year | “Data analyses showed small but significant correlations between purpose and each of the other three character strengths under investigation. Interview data revealed patterns in ways that adolescents acted on their purposeful aspirations; and interview analyses identified qualitative differences in expressions of gratitude and compassion between adolescents who were fully purposeful and those who were not. The findings suggest that character development can be better understood by investigating the multidirectional developmental relationships among different character strengths.”  Subheadings: 1) Survey Results, 2) Interview results (Prevalence and domains of purpose, Engaging in purpose activity, Purpose and other character strengths in early adolescence) |
| Marshall, S., Reidlinger, D. P., Young, A., & Isenring, E. (2017). The nutrition and food-related roles, experiences and support needs of female family carers of malnourished older rehabilitation patients. Journal of Human Nutrition and Dietetics, 30(1), 16-26.  Country: Australia | "What are the nutrition and food-related roles, experiences and support needs of female family carers of community dwelling malnourished older adults admitted to rehabilitation units in rural New South Wales (NSW), Australia, both during admission and following discharge?" | phenomenology & qualitative longitudinal research | Population: Female family carers of community dwelling malnourished older adults (n=4)  Data: Individual interviews  Length of data collection: About 2 weeks | “Three drivers were identified. ‘Responsibility’ was related to the agency who assumed responsibility for providing nutrition support and understanding family carer obligation to provide nutrition support. ‘Family carer nutrition ethos’ was related to how carer nutrition beliefs, knowledge and values impacted the nutrition support they provided, the high self-efficacy of family carers and an incongruence with an evidence-based approach for treating malnutrition. ‘Quality of life’ was related to the carers’ focus upon quality of life as a nutrition strategy and outcome for their care-recipients, as well as how nutrition support impacted upon carer burden.”  Subheadings: 1) Driver: Responsibility (Agency responsible for providing nutrition support, Family carer obligation), 2) Driver: Family carer nutrition ethos (Family carer self-efficacy, Incongruence with evidence-based approach), 3) Driver: Quality of life (Focus on care-recipient quality of life, Family carer burden) |
| McGeechan, G. J., McPherson, K. E., & Roberts, K. (2018). An interpretative phenomenological analysis of the experience of living with colorectal cancer as a chronic illness. Journal of Clinical Nursing, 27(15), 3148-3156.  Country: UK | "The aim of this study was to qualitatively explore the psychosocial and physical consequences of living with colorectal cancer as a chronic illness and how this changes survivor’s views and plans for their future, over time." | Phenomenology & qualitative longitudinal research | Population: Patients with colorectal cancer who attending oncology follow-up clinics (n=6)  Data: Individual interviews  Length of data collection: 6 months | “Two super-ordinate themes emerged from the analysis: physical and psychological consequences of cancer and adapting to life after treatment.”  Subheadings: 1) Physical and psychological consequences of cancer (Fear of cancer recurrence, Functional impairment), 2) Adapting to life after treatment (Making sense of symptoms, Lifestyle adjustments, Functional social support) |
| McKay, V. R., Dolcini, M. M., & Catania, J. A. (2017). Impact of human resources on implementing an evidence-based HIV prevention intervention. AIDS and Behavior, 21(5), 1394-1406.  Country: US | "Using the Interactive Systems Framework, we explored staff fluctuation and the subsequent influence on RESPECT, an HIV prevention EBI [evidence based interventions]." (from abstract) | not described | Population: Staff responsible for providing RESPECT directly to clients (n=53)  Data: Individual interviews and questionnars  Length of data collection: 12 months | “We analyzed interviews qualitatively to describe changes among RESPECT staff and explore the subsequent influences on RESPECT implementation. Organizations reported downsizing, turnover, and expansion of staff positions. Staff changes had multiple influences on RESPECT implementation including clients reached, fidelity to specific RESPECT protocols, and overall sustainability of RESPECT over time. HR fluctuations are common, and our analyses provide an initial characterization of the relationship between HR fluctuation and EBI implementation.”  Subheadings: 1) The impact of staff changes on implementation of respect (Changes in the clients served and program maintenance, Changes in skill and knowledge, Changes in workload for remaining employees, Innovative approaches to downsizing), 2) Case studies (Case I, Case II) |
| McKeganey, N., & Barnard, M. (2018). Change and Continuity in Vaping and Smoking by Young People: A Qualitative Case Study of A Friendship Group. International Journal of Environmental Research and Public Health, 15(5), 1008.  Country: UK Scotland | "In this paper, we report data from a research study that was designed to explore the possible fluidity in young peoples’ perceptions and engagement with e-cigarettes by re-interviewing the members of a small teenage friendship group in Glasgow, Scotland, focusing on how their relationship and perception of e-cigarettes changed over a six-month period." | Case study | Population: Young people (n=8)  Data: Individual interviews  Length of data collection: 6 months | “At time 1, vaping generated much excitement and interest, with six out of eight individuals having their own vape device. At time 2, only two young people still vaped, with the others no longer professing any interest in continued vaping. The two regular smokers, who had been smoking before they first vaped, now only vaped privately and to reduce their tobacco intake. This small case study illustrates plasticity in the use of these devices; just as young people can move into their use, so too can they move away from them. This small study underscores the importance of differentiating between long-term, frequent, consistent use and more episodic, experimental and infrequent use by young people and for undertaking a measurement of actual e-cigarette use at multiple time points in both quantitative and qualitative studies. In addition, the case study illustrates the powerful impact which peers can have on teenagers use of e-cigarettes.”  Subheadings: 1) Smoking and vaping hierarchies, 2) Change and continuity: six months later, 3) Vaping and smoking |
| McKenzie, S. A., Rasmussen, K. M., & Garner, C. D. (2018). Experiences and perspectives about breastfeeding in “public”: A qualitative exploration among normal-weight and obese mothers. Journal of Human Lactation, 34(4), 760-767.  Country: US | "The aims of this study were to (a) describe U.S. women’s experiences with breastfeeding in public and (b) describe how obese women’s experiences compared with normal-weight women’s experiences." | Qualitative longitudinal research | Population: Pregnant women in central New York who intended to breastfeed (n=26)  Data: Individual interviews and fieldnotes  Length of data collection: About 9 months | “The concept of ‘public’ was situational rather than a set of physical places; women experienced challenges while breastfeeding around others in private locations that were indistinguishable from those they encountered in places typically considered public. Women experienced social and physical awkwardness including perceived lack of acceptability, fear of confrontation, exposure, and positioning difficulties. They used strategies to reduce awkwardness, for example, being ‘discreet’ and minimizing breastfeeding around other people. Obese women experienced similar challenges but to a greater degree than normal-weight women.”  Subheadings: Participant characteristics, 1) Interview data, 2) Using health literacy capacities for selfmanagement of health and illness (Health information seeking, Side- effects of medication use), 3) Psychological factors that impact use of health literacy capacities (Perceptions of control, Emotional reactions), 4) Structural factors that impact use of health literacy capacities (Being able to access health services, Environment, Affordability), 5) Quality of relationship with the HCP (Qualities of the HCP, Accessing and appraising information with the HCP) |
| Meijer, E., Vangeli, E., Gebhardt, W. A., & van Laar, C. (first published 2018). Identity processes in smokers who want to quit smoking: A longitudinal interpretative phenomenological analysis. Health, 2020, 24(5), 493-517.  Country: The Netherlands | "We investigated in-depth how smokers’ sense of identity may change during the process of quitting, and what happens to their sense of identity if they cannot quit successfully." | Phenomenology & qualitative longitudinal research | Population: Daily smokers who intended to quit smoking within 2 months (n=10)  Data: Individual interviews and questionnairs  Length of data collection: 22 months | “Two themes of identity change processes are presented: ‘Identity transition makes it easier to quit’ and ‘Identity conflict resolution is needed when quitting is unsuccessful or not attempted.’ Identity transition toward the identity of nonsmoker appeared to be facilitated by permeable identity boundaries, a continuous sense of identity, and a sense of mastery of quitting. Conflicted smoker identities were observed among participants who continued to smoke, along with barriers that appeared to prevent them from identifying with nonsmoking. Among these participants psychological (e.g. using downward comparisons with worse-off smokers) and behavioral strategies (e.g. hiding smoking from others) were seen that may serve to resolve identity conflict and protect a positive sense of identity.”  Subheadings: 1) Identity change toward “nonsmoker” makes it easier to quit (Nonsmoking becomes a part of identity, Permeable identity boundaries enable identity change, Identity change is facilitated by a sense of identity continuity, Identity change is facilitated by a sense of mastery of quitting), 2) Identity conflict resolution is needed when quitting is unsuccessful or not attempted (Barriers to identification with a positive future nonsmoker identity, Perceptions of quitting as not fitting with certain identity aspects, Expectations of feeling incomplete without smoking, Strategies to protect a positive sense of identity when being unable to quit, Psychological strategies, Behavioral strategies), 3) Follow-up survey (T4) |
| Milbourn, B., McNamara, B., & Buchanan, A. (2017). A qualitative study of occupational well-being for people with severe mental illness. Scandinavian Journal Of Occupational Therapy, 24(4), 269-280.  Country: Australia | "The purpose of this paper is to investigate the occupational well-being of Western Australian people diagnosed with an SMI [severe mental illness], who are under ACT [Assertive Community Treatment] care and living in the community, by use of the Occupational Well-being framework. The study seeks to determine if their occupational needs, as presented in the framework, are being met." | not described | Population: People diagnosed with an severe mental illness (n=11)  Data: Individual interviews  Length of data collection: 12 months | “Participants’ everyday activities and occupational well-being appeared severely restricted and largely determined by the type of care they received. There was minimal evidence of the well-being descriptors, though all the participants reported experiencing some form of pleasure, even though some of the pleasurable experiences negatively impacted their health.”  Subheadings: 1) Descriptor one: Accomplishment, 2) Descriptor two: Affirmation, 3) Descriptor three: Agency, 4) Descriptor four: Coherence, 5) Descriptor five: Companionship, 6) Descriptor six: Pleasure, 7) Descriptor seven: Renewal |
| Minton, C., Batten, L., & Huntington, A. (2019). A multicase study of prolonged critical illness in the intensive care unit: Families' experiences. Intensive & Critical Care Nursing, 50, 21-27.  Country: New Zealand | "The purpose of this study, which forms one part of a larger study, is to explore the experiences of family of ICU [intensive care unit] patients with a prolonged critical illness." | Case study | Population: Family of ICU patients (n=16)  Data: Observation described in field notes, in-depth interviews and informal conversations  Length of data collection: 17 to 66 days | “Relentless uncertainty dominated all phases of the trajectory for the family during a family member’s prolonged critical illness in the intensive care unit. When faced with a critical illness, family shifted rapidly into a world of unknowns. Family worked hard to navigate their way through the many uncertainties that dominated each phase of their family member’s illness.”  Subheadings: 1) Being overwhelmed, 2) Living in an uncertain world, 3) An altering uncertainty, 4) Uncertainty in a different location, 5) Moving on |
| Minton, C., Batten, L., & Huntington, A. (2018). The impact of a prolonged stay in the ICU on patients’ fundamental care needs. Journal of Clinical Nursing, 27(11), 2300-2310.  Country: New Zealand | "To explore patients’, families’ and health professionals’ experiences of a long-stay patient in an intensive care unit." (from abstract) | case study | Population: Patients’, families’ and health professionals’ in intensive care unit (n=47 participants)  Data: Observations, interviews, informal conversations, field notes and documents.  Length of data collection: 17 to 66 days | “Challenges and successes of providing fundamental care for long-stay ICU [intensive care unit] patients are attributed to two interlinked factors. First, the biomedical model influences ICU nursing practices, resulting in prioritising tasks and technology for patient survival while simultaneously devaluing relational and comfort work. Fundamental psychosocial needs such as family presence, comfort, relationships and communication may be unmet. Second, the unit environment and culture have a significant impact on long-stay patients’ ICU experiences and form physical and psychological barriers to families being present and involved. Some nurses negotiated these challenges to provide fundamental, patient- and family-centred care by adopting an approach of knowing the patient and these nurses reported satisfaction when seeing patients’ positive responses.”  Subheadings: 1) Immediate and prompt interventions to maintain life, 2) The impact of culture on the delivery of person-centred fundamental care, 3) Promoting relationship-centred care |
| Mitterlechner, M. (2018). Governing integrated care networks through collaborative inquiry. Journal of Health Organization and Management, 32(7), 860-874.  Country: Switzerland | "The purpose of this paper is to develop a theory of governing in integrated care networks. Asking how and why the governance of these networks emerges and evolves over time, it responds to calls for more innovative thinking in this field." | case study | Population: Project meetings at healthcare Centres  Data: Semi-structured interviews, participant observations and archival data.  Length of data collection: Data was collected prospectively over 4 years, documents were collected another 5 years back | “Actors governed the network through repetitive sequences of collaborative inquiry, a practice through which they defined and addressed recurrent problems of network governance and joint network action in creative and experimental ways.”  Subheadings: A case study from the Swiss Lower Engadin, 1) Period 1: non-governance and the emergence of a regional care crisis (2000 – 2006), (Mode of network governance, Joint network action and problematic consequences, Collaborative inquiry and transition to period 2), 2) Period 2: hybrid governance and improving management capacities (2007–2012) (Mode of network governance, Joint network action and problematic consequences, Collaborative inquiry and transition to period 3), 3) Period 3: hybrid governance and care coordination (from 2013) (Mode ofnetwork governance, Joint network action and problematic consequences) |
| Monaro, S., West, S., Pinkova, J., & Gullick, J. & Pinkova, J. (2018). The chaos of hospitalisation for patients with critical limb ischaemia approaching major amputation. Journal of Clinical Nursing, 27(19), 3530-3543.  Country: Australia | "To illuminate the hospital experience for patients and families when major amputation has been advised for critical limb ischaemia (CLI)." ( from abstract) "This study, from the same sample, reports only the experiences of hospitalisation and therefore aims to illuminate the hospital lifeworld of patients with CLI who have been advised to have an amputation, in order to acquire a better understanding of the experience and to inform improvements in person-centred, interdisciplinary hospital care." (from article) | phenomenology | Population: Patients and families when major amputation has been advised for critical limb ischaemia (n=27)  Data: Individual interviews  Length of data collection: 6 months | “Hospitalisation for CLI [critical limb ischaemia], with or without amputation, created a sense of chaos, characterised by being fragile and needing more time for care (fragile body and fragile mind, nurse busyness and carer hypervigilance), being adrift within uncontrollable spaces (noise, unreliable space, precarious accommodation and unpredictable scheduling) and being confused by missed and mixed messages (multiple stakeholders, information overload and cultural/linguistic diversity).”  Subheadings: 1) Being fragile and needing more time for care, 2) Being adrift within uncontrollable spaces, 3) Being confused by missed and mixed messages |
| Monrouxe, L. V., Bullock, A., Gormley, G., Kaufhold, K., Kelly, N., Roberts, C. E., Mattick, K., & Rees, C. (2018). New graduate doctors' preparedness for practice: a multistakeholder, multicentre narrative study. BMJ Open, 8(8), e023146-e023146.  Country: UK (England, Northern Ireland, Scotland and Wales) | "We aim to explore issues around preparedness for practice in terms of how the concept is understood across a range of stakeholder groups and to understand aspects in which new medical graduates are deemed prepared (or unprepared) for clinical practice with the following two broad research questions (RQ): RQ1: How do stakeholders conceptualise ‘preparedness for practice’? RQ2: To what extent do various stakeholders perceive recent medical graduates to be prepared for practice, and what factors do they attribute to this?" | not described | Population: Newly graduated doctors, clinical educators, training programme leads, nurses, pharmacists, managers, policy and government officials (n=185)  Data: Individual interviews, focus group interviews, and audio diaries  Length of data collection: 3 months | “We identified 2186 narratives across all participants (506 classified as ‘prepared’, 663 as ‘unprepared’, 951 as ‘general’). Seven themes were identified; this paper focuses on two themes pertinent to our research questions: (1) explicit conceptualisations of preparedness for practice; and (2) newly graduated junior doctors’ preparedness for the General Medical Council’s (GMC) outcomes for graduates. Stakeholders’ conceptualisations of preparedness for practice included short-term (hitting the ground running) and long-term preparedness, alongside being prepared for practical and emotional aspects. Stakeholders’ perceptions of medical graduates’ preparedness for practice varied across different GMC outcomes for graduates (eg, Doctor as Scholar and Scientist, as Practitioner, as Professional) and across stakeholders (eg, newly graduated doctors sometimes perceived themselves as prepared but others did not).”  Subheadings: 1) theme 1: explicit conceptualisations of preparedness for practice, 2) theme 2: newly graduating doctors’ preparedness across the GMC’s outcomes for graduates (Doctor as scholar and scientist, Doctor as practitioner, Preparedness for patient consultations, Preparedness for diagnosing and managing conditions, Preparedness for communicating effectively with patients and colleagues, Preparedness for prescribing drugs safely, effectively and economically, Preparedness for carrying out practical procedures safely and effectively, Preparedness for using information effectively in the clinical environment, Doctor as professional, Preparedness for ethical and legal aspects, Preparedness for reflecting, learning and teaching others, Preparedness for learning and working effectively in multiprofessional teams, Protecting patients and improving care) |
| Moore, A. M., Dennis, M., Anderson, R., Bankole, A., Abelson, A., Greco, G., & Vwalika, B. (2018). Comparing women's financial costs of induced abortion at a facility vs. seeking treatment for complications from unsafe abortion in Zambia. Reproductive Health Matters, 26(52) 1522195.  Country: Zambia | "This longitudinal study assesses the costs of abortion for the woman and her family, comparing women who obtained an abortion at a facility with those who arrived at a health facility experiencing abortion complications." | not described | Population: Women and their family (n=38 participants)  Data: Interviews and questionnairs  Length of data collection: 3 to 4 months | “About two-thirds of the costs had been incurred by T1, while an additional one-third ofthe total costs was incurred between T1 and T2. Women in all three wealth tertiles sought a TOP [legal termination of pregnancy] in a health facility or an unsafe abortion outside a facility. Women who obtained CUA [unsafe abortions] tended to be further removed from the money that was used to pay for their abortion care. Women’s financial dependence leaves them unequipped to manage a financial shock such as an abortion. Improved TOP and post-abortion care are needed to reduce the health sequelae women experience after both types of abortion-related care.”  Subheadings: 1) Direct and indirect costs of a TOP vs. CUA, 2) Economic factors related to whether women access safe abortion vs. unsafe abortion and subsequent care for complications, 3) Sources of money for abortion-related expenses |
| Morris, R. L., & Sanders, C. (2018). Critical moments in long-term condition management: A longitudinal qualitative social network study. Chronic Illness, 14(2), 119-134.  Country: UK | "The aim of this paper is to explore how long-term condition management changes over time and the influence of social network members on where and how people seek support." | not described | Population: Individuals who had either diabetes, irritable bowel syndrome, or chronic obstructive pulmonary disease (n=30)  Data: Individual interviews and drawings/ maps  Length of data collection: 12 months | “Findings illuminated that changes in health and changes in social networks can influence each other. The social networks implicated in the management of long-term conditions change over time at critical moments and can range between positive and negative reciprocal influences with self-management. Network changes, such as the breaking or reconnecting of ties influenced the context of health management and the degree of engagement with self-management activities.”  Subheadings: 1) Defining critical moments, 2) Positive change to health management over time, 3) The influence of re-evaluating social roles, 4) Reciprocal influence ofenhancing self-management and social network members, 5) Negative changes to health management over time, 6) Familial change and loss influencing health, 7) Health management remained the same over time, 8) Normalised health management over time |
| Morrow, V., Tafere, Y., Chuta, N., & Zharkevich, I. (2017). "I started working because I was hungry": The consequences of food insecurity for children's well-being in rural Ethiopia. Social Science & Medicine 182, 1-9.  Country: Ethiopia | "By exploring children's accounts of their experiences, we highlight the effects of food insecurity on children's well-being in Ethiopia, how food insecurity affects crucial decisions over the life course and how these differ by gender; and the importance of sources of support over time." | Case study & qualitative longitudinal research | Population: Rural children with experience of food insecurity (n=8)  Data: Individual interviews, group discussions, and creative methods  Length of data collection: 7 years | “Children's descriptions of the importance of food and a varied diet (dietary diversity) in everyday life were expressed in a range of qualitative methods, including interviews, group discussions and creative methods. The paper suggests that while the overall picture of food security in Ethiopia has improved in the past decade, for the poorest rural families, food insecurity remains a major factor influencing decisions about a range of matters e children's time allocation, whether to continue in school, whether to migrate for work, and whether they marry. The paper argues that experiences of food insecurity need to be understood holistically, in relation to other aspects of children's lives, at differing stages of the lifecourse during childhood.”  Subheadings: 1) The importance of food for well-being, 2) Interplay of food insecurity, children's work and gender, 3) The importance of formal and informal sources of support, 4) Towards a framework for analysing the intersections of food insecurity and children's trajectories |
| Mozaffar, H., Cresswell, K. M., Williams, R., Bates, D. W., & Sheikh, A. (2017). Exploring the roots of unintended safety threats associated with the introduction of hospital ePrescribing systems and candidate avoidance and/or mitigation strategies: A qualitative study. BMJ Quality & Safety, 26(9), 722-733.  Country: UK | "We therefore revisited the large body of ethnographic evidence generated from the cases to review the evidence for roots of reported unintended safety threats associated with the introduction of ePrescribing in design, implementation and use, in order to develop a taxonomy of these factors, and use these insights to shed light on possible risk mitigation strategies." | Ethnography, case study & qualitative longitudinal research | Population: Health-care providers, managers, policy makers  Data: Individual interviews, observation, and documents (e.g., project plans, risk logs and business cases)  Length of data collection: 2 years | “Our dataset included 214 interviews, 24 observations and 18 documents. We developed a taxonomy of factors underlying unintended safety threats in: (1) suboptimal system design, including lack of support for complex medication administration regimens, lack of effective integration between different systems, and lack of effective automated decision support tools; (2) inappropriate use of systems—in particular, too much reliance on the system and introduction of workarounds; and (3) suboptimal implementation strategies resulting from partial roll-outs/dual systems and lack of appropriate training. We have identified a number of system and organisational strategies that could potentially avoid or reduce these risks.”  Subheadings: 1) Origins of safety threats associated with the introduction of ePrescribing (Inadequacies in system design, Inappropriate use of system, Problems arising from implementation strategies and infrastructure), 2) Strategies to avoid/mitigate unintended safety threats (Technological strategies, Organisational strategies) |
| Mueller, A. S., Jenkins, T. M., Osborne, M., Dayal, A., O'Connor, D. M., & Arora, V. M. (2017). Gender differences in attending physicians' feedback to residents: A qualitative analysis. Journal of Graduate Medical Education, 9(5), 577-585.  Country: US | "...in this study, our aim was to use qualitative data to better understand the lagging performance evaluations of female EM [emergency medicine] residents in PGY-3 [postgraduate year 3]." | not described | Population: Teachers (n=67)  Data: Text comments from student evaluations  Length of data collection: 2 years | “Analysis of the comments revealed that the ideal EM [emergency medicine] resident possesses many stereotypically masculine traits. Additionally, examination of a subset of the residents (those with 15 or more comments, n=35) showed that when male residents struggled, they received consistent feedback from different attending physicians regarding aspects of their performance that needed work. In contrast, when female residents struggled, they received discordant feedback from different attending physicians, particularly regarding issues of autonomy and assertiveness.”  Subheadings: Demographics, 1) Characteristics Valued During EM Residency, 2) Gender Differences in Feedback |
| Munford, R., & Sanders, J. (2019). Harm, opportunity, optimism: Young people's negotiation of precarious circumstances. International Social Work, 62(1), 185-197.  Country: New Zealand | "The study aimed to develop an understanding of the lived experiences, contexts and transitions of young people who faced high levels of adversity throughout their childhood. Of particular interest was investigating identity development and experiences of education and employment. The first-person accounts and those of their trusted others enabled an exploration of how these young people mediated challenging circumstances and negotiated for resources and support from both informal networks and formal service systems." | not described | Population: Young people who faced high levels of adversity, and their trusted others (n=107)  Data: Individual interviews  Length of data collection: About 3 years | “The article explores experiences in two domains: education and employment.”  Subheadings: 1) Disrupted education and precarious employment (Education experiences, Employment experiences), 2) Transformative practice – Expanding opportunities for vulnerable young people |
| Musesengwa, R., Chimbari, M. J., & Mukaratirwa, S. (2017). Initiating community engagement in an ecohealth research project in Southern Africa. Infectious Diseases of Poverty, 6(1), 22-22.  Country: Botswana, South Africa and Zimbabwe | "This paper aims to outline the process of initiating community engagement in an ecohealth study and to describe the issues emerging from its development and implementation." | Case study | Population: Headmen, community liaison officers, principal investigators, country coordinators, project team members, CAB members, community researcher assistants, nurses (n=17)  Data: Individual interviews, participatory workshops, unstructured interviews and direct observations  Length of data collection: 18 months | “The two sites had different cultural values, research literacy levels, and political and administrative structures. The engagement process included 1) introductions to the administrative and political leaders of the area; 2) establishing a community advisory mechanism; 3) community empowerment and 4) initiating sustainable post-study activities. In both sites the study employed community liaison officers to facilitate the community entry and obtaining letters of permission. Both sites opted to form Community Advisory Boards as their main advisory mechanism together with direct advice from community leaders. Empowerment was achieved through the education of ordinary community members at biannual meetings, employment of community research assistants and utilising citizen science. Through the research assistants and the citizen science group, the study has managed to initiate activities that the community will continue to utilise after the study ends. General strategies developed are similar in principle, but implementation and emphasis of various aspects differed in the two communities.”  Subheadings: 1) Obtaining community approvals, 2) Research literacy, 3) Establishing community advisory mechanisms, 4) Community empowerment, 5) Engaging community research assistants, 6) Utilising a citizen science approach, 7) Initiating sustainable post study activities |
| Musto, M. (2019). Brilliant or bad: The gendered social construction of exceptionalism in early adolescence. American Sociological Review, 84(3), 369-393.  Country: US | "...to identify the processes by which educators’ differential responses to boys’ rule-breaking by course level produced gender differences in students’ perceptions of intelligence. AND to illustrate how race intersected with gender when shaping students’ perceptions of intelligence. AND Do students’ gender beliefs about intelligence and exceptionalism vary by course level? If so, what are the processes encouraging students to perceive girls and boys as having different dispositions toward school, and how do their beliefs differ by course level? Does race intersect with gender when shaping higher- and lower-level students’ gender beliefs about intelligence and exceptionalism? If so, how?" | Ethnography | Population: Students and teachers (n=196)  Data: Individual interviews, focus group interviews, and observations  Length of data collection: About 2.5 years | “(…) this article demonstrates how educators’ differential regulation of boys’ rule-breaking by course level contributed to gender-based differences in students’ perceptions of intelligence. In higher-level courses—where affluent, White, and Asian American students were overrepresented—educators tolerated 6th-grade boys’ rule-breaking, such that boys challenged girls’ opinions and monopolized classroom conversations. By 8th grade, students perceived higher-level boys as more exceptionally intelligent than girls. However, in lower-level courses—where non-affluent Latinx students were overrepresented—educators penalized 6th-grade boys’ rule-breaking, such that boys disengaged from classroom conversations. By 8th grade, lower-level students perceived girls as smarter than boys, but not exceptional. This article also demonstrates how race intersected with gender when shaping students’ perceptions of intelligence, with students associating the most superlatives with affluent White boys’ capabilities.”  Subheadings: 1) The gendered construction of exceptionalism at MHMS, 2) Gender relations in higher-level courses (Gender Beliefs in Higher-Level Courses), 3) Gender relations in lower-level courses (Gender Beliefs in Lower-Level Courses), 4) Perceptions of boys’ intelligence by race (Gender and Race in Higher-Level Courses, Gender and Race in Lower-Level Courses) |
| Myrin Westesson, L., Wallengren, C., Baghaei, F., & Sparud-Lundin, C. (2018). Reaching independence through forced learning: Learning processes and illness management in parents of children affected by hemophilia. Qualitative Health Research, 28(14), 2142-2154.  Country: Sweden | "The aim of this study therefore is to explore parents’ learning processes and illness management in daily life during the first year after the start of their child’s treatment." | grounded theory & qualitative longitudinal research | Population: Parents of children with hemophilia (n=8)  Data: Individual interviews and joint interviews  Length of data collection: 12 to 14 months | “The core category, reaching independence through forced learning, reflected the parents’ learning process and their experiences of the challenges during the first year after start of treatment. Incentives for learning were characterized by a longing to reach independence and regain control of one’s life situation. The emerging key incentive for learning was a desire to become independent of health care professionals. Early home treatment reduced the impact of the illness, and by supporting parents in different ways during the learning process, health care professionals can promote the parents’ trajectory toward independency.”  Subheadings: 1) The Core Category “Reaching Independence Through Forced Learning” (Overwhelmed by demands, Preoccupied with practicalities, Emerging independence) |
| Namukwaya, E., Murray, S. A., Downing, J., Leng, M., & Grant, L. (2017). 'I think my body has become addicted to those tablets'. Chronic heart failure patients' understanding of and beliefs about their illness and its treatment: A qualitative longitudinal study from Uganda. Plos One, 12(9), e0182876-e0182876.  Country: Uganda | "This study addresses a gap identified by Selman et al in a review of literature on HF [heart failure] in Africa in 2015 which highlighted the need for culturally sensitive research on patients’ experiences to explore if issues such as communication difficulties observed in high-income countries also exist for HF patients in Uganda." | grounded theory & qualitative longitudinal research | Population: Patients with heart failure (n=21)  Data: Individual inteviews and joint interviews  Length of data collection: 6 months | “A total of 40 face to face qualitative longitudinal interviews (36-patient alone, 4 paired-patient and family carer), were conducted with 21 patients. The findings revealed that heart failure patients were unaware of the symptoms of the illness and their definition of illness differed from that of health professionals. Patients understood their diagnosis, cause of illness, prognosis and the importance of the medicines differently from health professionals, and had insufficient information on self-care. Lay beliefs were used to explain many aspects of the illness and treatments. All these influenced where patients sought care and their adherence to treatment, self-care and follow up leading to uncontrolled disease.”  Subheadings: 1) Limited health literacy (Lack of knowledge and competency to attribute initial symptoms to ill health, Limited competency in appraising and understanding of information given on the diagnosis, cause and treatment of HF) 2) Lay knowledge (Lay definition of illness, The traditional and complementary medicine paradigm, The role of faith, cultural and religious beliefs, Lay perceptions of self-care) |
| Naraine, M. D., Fels, D. I., & Whitfield, M. (2018). Impacts on quality: Enjoyment factors in blind and low vision audience entertainment ratings: A qualitative study. Plos One, 13(12), e0208165-e0208165.  Country: Canada | "The research questions are: 1) what is the longitudinal impact on B/LV [blind and low vision] audiences of the Canadian integrated model of description for an eight-part television comedy; and 2) what are the positive and negative factors identified by users for the AD [audion description] and the show? We hypothesize that the longitudinal enjoyment of the show’s and the quality of the AD (as assessed by viewers) will be positive and that there will be a positive response to the integrated AD related to fit with the show style and describer’s voice characteristics such as pace, language and emotional match. We examine and report on the impact this Canadian-originated approach [2] has on Canadian B/LV [Blind and low vision] viewers over time, considering the emotional impact on B/LV viewers and their responses to this AD [audio description] approach." | not described | Population: People being blind or low vision (n=24)  Data: Open ended questions in questionnaires  Length of data collection: 2 months | “Major findings included that most participants found the integrative style entertaining, a fit with the specific episodes, and enjoyable. Some participants, however, preferred the conventional style and struggled with the language and topic of a dark comedy and its associated descriptions.”  Subheadings: 1) Between-episodes questionnaire (Negative responses; Post study questionnaire; Plot/action, character, setting; Pace; Language level) |
| Nash, B. H., & Mitchell, A. W. (2017). Longitudinal study of changes in occupational therapy students' perspectives on frames of reference. The American Journal Of Occupational Therapy, 71(5), 7105230010p7105230011-7105230010p7105230017.  Country: US | "The purpose of this longitudinal study was to explore students’ views of FoR [frames of reference] as they progressed through the didactic portion of an occupational therapy program and participated in Level I fieldwork. The existing research has tended to focus on how students learn and apply theory, MoP, [models of practice] and FoR throughout coursework and Level II fieldwork. This study was designed to address the following question: How do occupational therapy students’ perspectives of the value of FoR change over the course of the didactic portion of an occupational therapy program?" | phenomenology | Population: Occupational therapy students (n=34)  Data: Individual interviews  Length of data collection: 15 months | “Throughout the program, students’ understanding of how to use FoR [frames of reference] evolved as they learned to implement them in practice. Opinions of the use of FoR seemed to be influenced by the students’ perceptions of the use of FoR by their Level I fieldwork supervisors.”  Subheadings: 1) Theme 1: Value of Frames of Reference, 2) Theme 2: Use of Multiple Frames of Reference, 3) Theme 3: Evolving Understanding of Frames of Reference, 4) Theme 4: Frames of Reference as Just One Factor in Intervention |
| Nešporová, O. (2019). Hazy transition to fatherhood: The experiences of Czech fathers. Journal of Family Issues, 40(2), 143-166.  Country: Czech Republic | "The study focuses on the impacts of fatherhood on the everyday lives of new fathers, while taking into account issues surrounding the involvement of fathers and the various cultural constructs of fatherhood." | Qualitative longitudinal research | Population: Fathers, but also a few mothers (n=32)  Data: Individual interviews  Length of data collection: Up to 21 months | “The expectant fathers evinced vague plans concerning approaching fatherhood; some stressed involvement in childcare while others emphasized the provider role. The concept of a ‘hazy’ transition to fatherhood evolved based on the narratives of first-time fathers and is used to describe the indistinct character of the transition to fatherhood.”  Subheadings: 1) Expectations of Nascent Fathers, 2) First-Time Fathers’ Reports on the Life Changes Brought About by Fatherhood |
| Nicholas, D. B., Barrera, M., Granek, L., D'Agostino, N. M., Shaheed, J., Beaune, L., Bouffet, E., & Antle, B. (2017). Parental spirituality in life-threatening pediatric cancer. Journal of Psychosocial Oncology, 35(3), 323-334.  Country: Canada | "...this paper specifically focuses on how parents experienced and navigated spirituality and faith during the illness trajectory. The following questions are addressed: (1) What is the role of spirituality? (2) What is the relationship between spirituality and hope? and (3) How may spirituality change as a result of having a child with a poor prognosis?" | grounded theory | Population: Parents having a child with a poor prognosis (n=35)  Data: Individual interviews  Length of data collection: 9 months | “Spirituality included religious beliefs and practices, notions of a higher force or cosmos, relationship with a divine being, as well as elements emerging from meaning-making and relationships. Parental expectations of spirituality remained relatively constant across data collection time points (3–9 months postdiagnosis), although limited variation occurred relative to shifting circumstance (e.g., deterioration of the child’s condition). Spirituality appeared to offer: greater acceptance of parents’ inability to protect their child from harm related to her/his life-threatening illness, guidance and emotion decompression, and support from one’s faith community.”  Subheadings: 1) Themes related to beliefs and practices of spirituality (Spirituality and hope, Spirituality facilitates acceptance ofparental inability to protect one’s child from harm, Spirituality as a source ofguidance and emotional decompression, Illness as a moderator ofparental spirituality, Support from a faith community) |
| Nichols, V. P., Williamson, E., Toye, F., & Lamb, S. E. (2017). A longitudinal, qualitative study exploring sustained adherence to a hand exercise programme for rheumatoid arthritis evaluated in the SARAH trial. Disability & Rehabilitation, 39(18), 1856-1863.  Country: UK | "The aim of this parallel interview study was to explore the trial participants’ experiences of the exercise programme and, in particular, how successfully they adhered to the programme over time. We chose a longitudinal study design to investigate the transition from supervised exercise to independent exercise, seeking insight into facilitators and barriers to exercise and changes in symptoms/experience over time. Understanding this process is a crucial part of developing an effective implementation strategy to facilitate the uptake of the SARAH exercise programme into clinical practice." | phenomenology & qualitative longitudinal research | Population: Adults diagnosed with RA reporting pain and dysfunction of hands and who were either not on medication or on a stable drug regime for three months or more (n=14)  Data: Individual interviews  Length of data collection: 8 months | “At 4 months, 11/14 participants reported continuing with the exercises. By 12 months, 7/13 participants still reported exercising. The ability to establish a routine determined whether participants adhered to the exercise programme. This was sometimes influenced by practical issues. We also identified facilitators and barriers to regular exercise in the themes of the following: the therapeutic encounter, perceived benefit of exercises, attitude of mind, confidence, and unpredictability.”  Subheadings: 1) Establishing a routine, 2) Practical issues, 3) Facilitators and barriers to establishing a routine, 4) The therapeutic encounter, 5) Perceived benefits of exercise, 6) Attitude of mind, 7) Confidence, 8) Unpredictability |
| Nightingale, J., Hardy, M., & Snaith, B. (2018). Embedding consultant radiographer roles within radiology departments: A framework for success. Radiography, 24(4), 289-297.  Country: UK | "This article discusses the design, implementation and validation of an outcomes framework for benchmarking competencies for trainee or new-in-post consultant radiographers." | Phenomenology & qualitative longitudinal research | Population: Radiographers (n=5)  Data: Individual interviews  Length of data collection: 5 years | “Early interactions with framework objectives were mechanistic, but as participants better understood the role more creative approaches emerged. Despite diverse clinical expertise, the framework facilitated parity between participants, promoting transparency and credibility which was important in how the consultant role was perceived. All participants achieved all framework outcomes and were subsequently appointed to substantive consultant radiographer positions.”  Subheadings: 1) Initial interviews (Month 1), 2) Mid-point interviews (Month 6), 3) Final Interviews (Month 12), 4) Focus group - validation of interview findings (18 months), 5) Group interview e retrospective review of framework (5 years), 6) Framework revision |
| Nilsson, K., Bååthe, F., Andersson, A. E., Wikström, E., & Sandoff, M. (2017). Experiences from implementing value-based healthcare at a Swedish university hospital - An longitudinal interview study. BMC Health Services Research, 17(1), 169-169.  Country: Sweden | "This study explores how the representatives of four pilot project teams experienced implementing VBHC [value based healthcare] over a period of 2 years in four different groups of patients at a large Swedish University Hospital." | not described | Population: Healthcare developer, physicians and heads of department (n=20)  Data: Individual interviews  Length of data collection: About 20 months | “Value for the patients was experienced as the fundamental drive for implementing VBHC [value based healthcare]. However, multiple understandings of what value for patients’ means existed in parallel. The teams received guidance from consultants during the first 3 months. There were pros and cons to the consultant’s guidance. This period included intensive work identifying outcome measurements based on patients’ and professionals’ perspectives, with less interest devoted to measuring costs. The implementation process, which both gave and took energy, developed over time and included interventions. In due course it provided insights to the teams about the complexity of healthcare. The necessity of coordination, cooperation and working together inter-departmentally was critical.”  Subheadings: 1) Getting started (Pros and cons of being guided by consultants, The process of identifying outcome measurements, Patients’ involvement), 2) Being on the road (Energy giver and thief, Getting stuck and later on regain renewed engagement), 3) Being able to look forward (Measurement as a means to improvement, Coordination between different developmental projects, Cooperation across boundaries) |
| Nixon, S. A., Bond, V., Solomon, P., Cameron, C., Mwamba, C., Hanass-Hancock, J., Margaret C. Maimbolwa, J. Menon, A., Simwaba, P., Sinyinza, R., Siwale, M., Tattle, S., & Yates, T. (2018). Optimism alongside new challenges: Using a rehabilitation framework to explore experiences of a qualitative longitudinal cohort of people living with HIV on antiretroviral treatment in Lusaka, Zambia. AIDS Care, 30(3), 312-317.  Country: Zambia | "This longitudinal qualitative study used a rehabilitation science approach to explore the experiences over time of women and men living with HIV and on antiretroviral therapy (ART) in the high HIV-prevalence setting of Lusaka, Zambia." | Qualitative longitudinal research | Population: Women and men living with HIV and on ART (n=35)  Data: Individual interviews  Length of data collection: About 12 months | “The central pattern that emerged across the participants’ narratives was the paradoxical experience of profound optimism alongside significant new challenges. Participants’ stories of hopefulness in the face of ongoing struggles played out in three interconnected themes: (1) impacts on my body and life; (2) interventions I am grateful to have and new interventions I need; and (3) stigma reduced and created by ART [antiretroviral therapy]. Results reflected the ups and downs of life with HIV as a chronic illness. Participants, whilst committed to and healthier on ART, typically experienced multiple physical, psychological and sensory impairments that varied in type, severity and trajectory. Participants valued improved relationships enabled by ART, but yearned for support for living long-term with HIV. Frequently participants reflected that their needs were overlooked related to managing side-effects, exercise, family planning and healthy sexuality. ART strengthened acceptance by self and others through improved health and productivity and through becoming a source of support for others. However, being on ART also led to stigma, driven by persistent associations with sickness, death, lack of productivity and uncertainty.”  Subheadings: 1) Theme 1: Impacts on my body and my life, 2) Theme 2: Interventions I am grateful to have and interventions that need to evolve, 3) Theme 3: Stigma reduced and also created by ART over time, |
| Nizza, I. E., Smith, J. A., & Kirkham, J. A. (2018). 'Put the illness in a box': A longitudinal interpretative phenomenological analysis of changes in a sufferer's pictorial representations of pain following participation in a pain management programme. British Journal of Pain, 12(3), 163-170.  Country: UK | "This article presents a single case from a wider study where IPA [interpretive phenomenological analysis] interviews with drawings were used longitudinally, to understand how pain and the sense of identity of sufferers changed following participation in a pain management programme (PMP). " | Case study and phenomenology | Population: Woman with fibromyalgia, degenerated discs and depression (n=1)  Data: Individual interviews, and drawings  Length of data collection: 9 months | “(…) revealing how, as control is regained, a sufferer’s relationship with their chronic pain can visibly change and how the drawings, when reviewed retrospectively, enable insight and ownership of progress.”  Subheadings: 1) Time 1: 2 months before the PMP crushed, overwhelmed and isolated, 2) Time 2: 1 month after the PMP - taking ownership of pain, 3) Time 3: 6 months after the PMP - the perils of normality |
| Nordin, A., Andersson Gäre, B., & Andersson, A.-C. (2017). Emergent programme theories of a national quality register - A longitudinal study in Swedish elderly care. Journal of Evaluation in Clinical Practice, 23(6), 1329-1335.  Country: Sweden | "The purpose of this study is to examine and establish the PTs [programme theories] of SA [Senior alert] in CMSs [clinical microsystems] at work units in elderly care. By comparing their PTs with that of the initiator, the paper reports on how PTs in CMSs emerge in relation to the established PT." | case study & qualitative longitudinal research | Population: Senior alert experts (n=15)  Data: Individual interviews  Length of data collection: About 2 years | “The initiator and change recipients described similar programme logics, but differing programme theories. With time, change recipients' programme theories emerged. Their programme theories converged and became more like the programme theory of the initiator.”  Subheadings: 1) The initiator's PT, 2) The CMSs' PTs (Change recipients, Material and artefacts, Actions, Outcomes), 3) ANALYSIS (Similar programme logics but dissimilar programme theories, Confusion between motivation and discomfort, Confusion between teamwork and solitary registration, Connection between improved outcomes and patient‐oriented expectations, Emergent and converging programme theories, Disconnection between learning, improved work, and outcomes) |
| Ober, J. L., & Lape, J. E. (2019). Cultivating acute care rehabilitation team collaboration using the kawa model. Internet Journal of Allied Health Sciences & Practice, 17(3), 1-8.  Country: US | "...to investigate the impact of a teambuilding intervention with use of the Kawa model on acute care rehabilitation team collaboration" | not described | Population: Rehabilitation staff members (n=8)  Data: Questionnairs with open-ended questions  Length of data collection: 5 weeks | “Outcomes showed overall mean improvements in agreement that the Kawa model provides a common method of communication, and 100% of the participants agreed or strongly agreed that use of the Kawa model can improve acute care rehabilitation team collaboration. Qualitative post-survey responses indicated an enhanced understanding of the components of effective team collaboration.”  Subheadings: 1) Quantitative Results, 2) Qualitative Results |
| Pappne Demecs, I., & Miller, E. (2019). Participatory art in residential aged care: A visual and interpretative phenomenological analysis of older residents' engagement with tapestry weaving. Journal of Occupational Science, 26(1), 99-114.  Country: Australia | "In the ‘Tapestry of Home’ project described here, a professional tapestry artist moved her practice and a 1.8 by 2 meter loom into a residential aged care for 6 months to creatively engage residents. This paper explores if and how creative occupation, a participatory art project, might benefit older people living in residential aged care." | phenomenology, case study & participatory research | Population: Older people living in residential aged care (n=3)  Data: Observations, field notes, researcher produced photographs and videos, informal conversations, and individual interviews.  Length of data collection: 6 months | “(…) we describe how three residents experienced the project in different ways: gradually, partially and fully engaged, experiencing ‘flow’ through weaving. Two superordinate themes emerged (creative anticipation and the connecting loom), with researcher-produced photographs visually conveying, comparing and contrasting how these three residents experienced the participatory art project.”  Subheadings: 1) Creative anticipation, 2) The loom: Joy of curiosity, 3) The artist: Motivations, personality and trust, 4) The connecting loom, 5) Connecting to the past, 6) Connecting to others, 7) Connecting to creative self |
| Parappilly, B. P., Mortenson, W. B., Field, T. S., & Eng, J. J. (first published 2019). Exploring perceptions of stroke survivors and caregivers about secondary prevention: A longitudinal qualitative study. Disability and Rehabilitation, 2020, 42(14), 2020-2026.  Country: Canada | "...to explore how the perceived barriers and facilitators associated with participation in secondary prevention activities change over the early stroke recovery period among stroke survivors and their family members." | Qualitative longitudinal research | Population: Stroke survivors and their familiy member (n=28)  Data: Individual interviews  Length of data collection: 6 months | “Thematic analysis identified three themes. (1) ‘A soul searching experience’ indicating that the stroke was anxiety provoking leading to a soul searching experience into exploring its causes to adopt a healthy lifestyle; (2) ‘Old habits die hard’ revealing that stroke survivors encountered barriers to adopt healthy lifestyle changes; (3) ‘Making a fresh start’ recognizing that participants were motivated to make their lifestyle healthier and most sustained some lifestyle changes over the past 6 months.”  Subheadings: 1) Theme 1: a soul-searching experience, 2) Theme 2: old habits die hard, 3) Theme 3: making a fresh start |
| Parker, S., & Mayock, P. (2019). "They're always complicated but that's the meaning of family in my eyes": Homeless youth making sense of "family" and family relationships. Journal of Family Issues, 40(4), 540-570.  Country: Ireland | "(a) What is the nature and shape of homeless youths’ relationships with their families? (b) In what way, if at all, do these relationships change over time? and (c) How do homeless young people construct and make sense of “family” in the context of their own family experiences?" | Qualitative longitudinal research | Population: Homeless youth age 16-24 and their family member (n=50)  Data: Individual interviews  Length of data collection: About 2 years | “Based on selected findings from a qualitative longitudinal study, this article explores the meaning-making processes of homeless young people with explicit attention to the ways in which ‘family’ is produced and (re) negotiated in their lives over time. Four themes are presented—family as reliable and supportive; family as interrupted and ‘broken’; family as fragile and elusive; and family as fluid and ambiguous—revealing the unfolding nature of young people’s constructions of family and family relationships.”  Subheadings: 1) Young People’s Paths to Homelessness: The Role of Family Conflict, 2) Young People’s Level of Contact With Family, 3) Rebuilding Family Relationships: The Process of Reconciliation, 4) Homeless Young People Making Sense of “Family”, 5) Family as Reliable and Supportive, 6) Family as Interrupted and “Broken”, 7) Family as Fragile and Elusive, 8) Family as Fluid and Ambiguous |
| Parkinson, P., & Cashmore, J. (2018). Relocation and the indissolubility of parenthood. Journal of Child Custody, 15(1), 76-92.  Country: Australia | "This article is based upon the findings of a five-year prospective longitudinal study of relocation disputes in Australia, involving interviews with 80 parents and 33 children in 70 families." | Qualitative longitudinal research | Population: Parents and children i families (n=113 participants)  Data: Individual interviews  Length of data collection: 18 months to 2 years | “In the five years following the relocation dispute, much changed for some of these families. A few mothers left without their children; several mothers returned to the original location; some fathers followed. There were also changes in some custody arrangements. The mothers who moved were not surprisingly satisfied with the outcome; however, even mothers who were not allowed to move mostly showed greater improvements in stress levels and mental health than fathers over the five year period, even if the fathers successfully opposed the move. Several mothers adapted to staying if they could see the benefit to their children. All children who moved adapted to the new location and made new friends; however, those who had close relationships with their father found it very hard to be a long distance from him.”  Subheadings: 1) Outcomes of the dispute, 2) Mothers who moved, 3) Mothers’ reactions to a negative outcome, 4) Fathers’ reactions to a negative outcome, 5) Parents’ stress levels and health, 6) Fathers’ contact with their children after a relocation, 7) Children’s experiences of relocation disputes, 8) Children’s views about wanting to move or not, 9) Children’s adaptation to the relocation decision |
| Payne, S., Eastham, R., Hughes, S., Varey, S., Hasselaar, J., & Preston, N. (2017). Enhancing integrated palliative care: what models are appropriate? A cross-case analysis. BMC Palliative Care, 16(1), 64-64.  Country: UK | "We aimed to investigate accounts of hospice integration with local health care providers, using the framework provided by the model in Fig. 1, to determine how service users and healthcare professionals perceived palliative care services and the extent of integration experienced. In addition, we seek to investigate practices associated with care as experienced by patients, family carers and health professionals which promote or limit integration." | case study | Population: Patients, family members and health-care providers (n=70)  Data: Individual interviews, joint interviews, and focus group interviews  Length of data collection: 3 months | “While some care fell short of expectations, all patients reported high levels of satisfaction and valued continuity of care and efficient information sharing. All hospices supported and supplemented local providers, with three hospices also supplanting local provision by providing in-patient facilities.”  Subheadings: 1) Hospice A – Service description, 2) Hospice A - patient/carer perspectives, (Male COPD patient, Male cancer patient, Male CHF patient and female carer), 3) Hospice B – Service description, 4) Hospice B – Patient/carer perspectives (Male COPD patient and female carer, Female cancer patient), 4) Hospice C – Service description, 5) Hospice C – Patient/carer perspective (Female COPD patient, Female cancer patient, Female CHF patient and male carer), 6) Hospice D – service description, 7) Hospice D – patient/carer perspective (Male COPD patient, Female cancer patient, Female CHF patient and female carer), 8) Comparative analysis |
| Peek, S. T. M., Luijkx, K. G., Vrijhoef, H. J. M., Nieboer, M. E., Aarts, S., van der Voort, C. S., Rijnaard, M. D., & Wouters, E. J. M. (2019). Understanding changes and stability in the long-term use of technologies by seniors who are aging in place: A dynamical framework. BMC Geriatrics, 19(1), 236-236.  Country: The Netherlands | "In the current qualitative study, DST [Dynamical Systems Theory] is used as a theoretical lens while addressing the following research questions: (1) When and why does the frequency of use of technology by independentliving older adults remain stable over time; and (2) What drives changes in the frequency of use of technology by independent-living older adults." | not described | Population: Independent living older adults (n=33)  Data: Individual interviews, questionnairs, and observations of technologies in the participants homes  Length of data collection: About 19 months | “A core of six interrelated factors was closely linked to the frequency of technology use: emotional attachment, need compatibility, cues to use, proficiency to use, input of resources, and support. Additionally, disruptive forces (e.g., social influences, competition with alternative means, changes of personal needs) could induce change by affecting these six factors. Furthermore, long-term technology use was in some cases more resilient to disruption than in other cases. Findings were accumulated in a new framework: Dynamics In Technology Use by Seniors (DITUS).”  Subheadings: Sample, 1) Results of the thematic analysis, 2) Stable use of technologies, 3) Shifts to other states of use, |
| Peek, S. T. M., Luijkx, K. G., Vrijhoef, H. J. M., Nieboer, M. E., Aarts, S., van der Voort, C. S., Rijnaard, M.D., & Wouters, E. J. M. (2017). Origins and consequences of technology acquirement by independent-living seniors: Towards an integrative model. BMC Geriatrics, 17(1), 189-189.  Country: the Netherlands | "The current study aimed to understand the origins and consequences of technology acquirement by independent living older adults. We did this by exploring: (1) how and why technologies are acquired by independent-living older adults; and (2) the implications of the ways in which independent-living older adults acquire technologies." | Qualitative longitudinal research | Population: Independent-living older adults (n=33)  Data: Individual interviews, observations  Length of data collection: 18 months | “Findings were accumulated in a new conceptual model: The Cycle of Technology Acquirement by Independent-Living Seniors (C-TAILS), which provides an integrative perspective on why and how technologies are acquired, and why these may or may not prove to be appropriate and effective, considering an independent-living senior’s needs and circumstances at a given point in time. We found that externally driven and purely desire-driven acquirements led to a higher risk of suboptimal use and low levels of need satisfaction.”  Subheadings: Sample, 1) Status quo prior to acquirement (Challenges of independent living, Use of technological and non-technological means, Internal technology related schemas and attitudes, External influence of the social network, External influence of the social organizations, Physical environment), 2) Decisive developments within the status quo, 3) Acquirement enabling mechanisms, 4) Number and types of acquirements by participants, 5) Moderating factors affecting number and types of acquirements by participants, 6) Favorable and unfavorable consequences of acquirements |
| Pemo, K., Phillips, D., & Hutchinson, A. M. (2019). An exploration of breastfeeding practices by Bhutanese women. Journal of Human Lactation, 35(1), 181-191.  Country: Bhutan | "...to explore Bhutanese women’s views, intentions, and experiences related to exclusive breastfeeding" | Qualitative longitudinal research | Population: Women pregnant for the first time (n=24)  Data: Individual interviews  Length of data collection: About 6 weeks | “Two themes (breastfeeding, but uncertainty about achieving exclusive breastfeeding and acceptance that breastfeeding is painful) were identified from interviews at term. Five themes were identified from interviews at 6 weeks after birth (lack of timely breastfeeding information and support from health professionals, misconceptions about exclusive breastfeeding, being unprepared for the reality of breastfeeding, limited control or choice over feeding, and adoption of cultural and traditional practices).”  Subheadings: Demographic Characteristics, 1) Themes (Breastfeeding, but uncertainty about achieving EBF, Acceptance that breastfeeding is painful, Lack of timely breastfeeding information and support from health professionals, Misconceptions about EBF, Being unprepared for the reality of breastfeeding, Limited control or choice over feeding, Adoption of cultural and traditional practices) |
| Perry, J., Wöhlke, S., Heßling, A. C., & Schicktanz, S. (2017). Why take part in personalised cancer research? Patients' genetic misconception, genetic responsibility and incomprehension of stratification-an empirical-ethical examination. European Journal of Cancer Care, 26(5), e12563.  Country: Germany | "We examined the motivation for participation in the clinical trial from the patients' perspective" | longitudinal empirical-ethical study | Population: Colorectal cancer patients (n=40)  Data: Observations, and individual interviews  Length of data collection: 60 weeks | “In addition to therapeutic misconception based on patients’ confusion of research and treatment, and here triggered by misled motivation, information paternalism or incomprehension, we identified genetic misconception and genetic responsibility as new problematic issues. Patients mainly were not aware of the major research aim of future stratification into responders and non- responders nor did they fully acknowledge this as the aim for personalised cancer research.”  Subheadings: 1) Induced solidarity and social expectations as research participation motivation, 2) Genetic responsibility as genetic misconception, 3) Optimisation as personalised benefit, 4) Patients’ incomprehension of stratification as the research aim |
| Peruzzolo, D. L., Barbosa, D. M., & Ramos de Souza, A. P. (2018). Occupational therapy and babies treatment in premature intervention from a hypothesis of psychomotor functioning: Single case study. Brazilian Journal of Occupational Therapy, 26(2), 409-421.  Country: Brazil | "To analyze the effectiveness of a Hypothesis of Psychomotor Functioning (HPF) for treatment of the premature babies" | Case study | Population: Premature babies and occupational therapists (n=1)  Data: Individual interview, video recorded observations, and diaries.  Length of data collection: 3 months | “The case confirms that the psychomotor irregularity may be understood as a psychomotor symptom and its source construed as from a HPF [Hypothesis of Psychomotor Functioning]. The irregularity is considered in a singular way, as observation of how the child constructs its Body Scheme (BS) and Body Image (BI), starting from the bonds with parents. The treatment effectiveness was confirmed from a HPF [Hypothesis of Psychomotor Functioning], as the psychomotor symptom went away (BS), giving place for cognitive and psychomotor acquisitions, and to the maternal resignification of the investments in her baby (BI). The BS is taken as a cognitive and motor psychic functioning related to the BI in construction.”  Subheadings: Clinical Case Presentations, 1) Referral, 2) From the evaluation to the HPF production, 3) The hypothesis of psychomotor functioning for T. Girl, 4) The hypothesis of psychomotor functioning as a clinical path, 5) The evolution of T., 6) The end of treatment |
| Phipps, D. L., Jones, C. E. L., Parker, D., & Ashcroft, D. M. (2018). Organizational conditions for engagement in quality and safety improvement: A longitudinal qualitative study of community pharmacies. BMC Health Services Research, 18(1), 783-783.  Country: UK | "...to understand what is needed for successful improvement efforts in community pharmacies, both to ensure that they make an effective contribution to primary care quality and safety in their own right, and to identify general insights about improvement that might be applicable to other areas." | Qualitative longitudinal research | Population: Community pharmacies (n=10)  Data: Observations, field notes, and focus group interviews  Length of data collection: 12 months | “The progress made by pharmacies in improving their practice can be described in terms of a behavioural change framework, consisting of contemplation (resolving to make changes if they are required), planning (deciding how to carry out change) and execution (carrying out and reflecting on change). Organizational conditions supporting change were identified; these included the prioritisation of improvement, a commitment to change, a trusting and collaborative relationship between staff and managers, and knowledge about quality and safety issues to work on.”  Subheadings: 1) General observations, 2) Doing quality and safety improvement, 3) Facilitating quality and safety improvement |
| Poland, F., Spalding, N., Gregory, S., McCulloch, J., Sargen, K., & Vicary, P. (2017). Developing patient education to enhance recovery after colorectal surgery through action research: A qualitative study. BMJ Open, 7(6), e013498-e013498.  Country: UK | "This study aimed to examine the perceived value of patient education for patients undergoing colorectal surgery for cancer as one component of an ERAS multimodal approach, and how changing the education might better support enhanced recovery by supporting patient self-management." "The study objectives were to understand the role of preoperative education for patients undergoing colorectal surgery by involving patients, carers and staff in: (1) identifying perceived value and value deficits for enhanced recovery; (2) modifying current education practices to address educational deficits; and (3) evaluating these changes for preparing patients to enhance their recovery." | action research | Population: Patients undergoing colorectal surgery for cancer, carers and staff (n=138)  Data: Observations, individual interviews and focus group interviews, and questionnaires  Length of data collection: 12 weeks | “Themes identified were: (1) knowledge and engagement; (2) situated understanding and confidence building; and (3) partnership and proactive involvement in enhancing recovery. All patients articulated needs to prepare mentally and physically to plan for colorectal surgery and rehabilitation. Patients and carers wanted to counter uncertainty about medical procedures: likely bodily changes, recovery timescales and future. They therefore sought as much personalised, relevant information as possible about their disease, planned surgery and recovery. Staff implemented preoperative education to more specifically inform and respond multimodally to individual needs.”  Subheadings: 1) theme 1: knowledge and engagement, 2) theme 2: situated understanding and confidence building, 3) theme 3: partnership and proactive involvement in enhancing recovery |
| Pope, C., McKenna, G., Turnbull, J., Prichard, J., & Rogers, A. (2019). Navigating and making sense of urgent and emergency care processes and provision. Health Expectations, 22(3), 435-443.  Country: UK | "...to explore how people make sense of urgent care provision and processes, and how this impacts on their navigation of services." | not described | Population: Regular users of emergency care, potentially marginalized users, and people from East/Central Europé (n=93)  Data: Individual interviews  Length of data collection: 6 to 12 months | “Participants narratives illuminated considerable uncertainty and confusion regarding urgent and emergency care provision which in part could be traced to the contingent nature of urgent and emergency care need. Accounts of emergency care provision were underpinned by strong moral positioning of appropriate help- seeking, demarcating legitimate service use that echoed policy rhetoric, but did not necessarily translate into individual behaviour. People struggled to make sense of urgent care provision making navigating ‘appropriate’ use problematic.”  Subheadings: 1) The confusing boundaries of urgent care service provision, 2) Contingent nature of need, 3) Moral positioning in making sense of when and how to use urgent care, 4) Re- imagined borders of urgent and emergency provision |
| Porter, T., Ong, B. N., & Sanders, T. (first published 2019). Living with multimorbidity? The lived experience of multiple chronic conditions in later life. Health, 2020, 24(6), 701-718.  Country: UK | "...to understand how older people living with multiple chronic conditions make sense of illness. Our aim is to provide a foundational reading of multiple chronic conditions, beginning with the premise that medical diagnoses do not a priori determine illness." | Phenomenology, Grounded theory & qualitative longitudinal research | Population: Older people living with multiple conditions and participant spouses (n=15)  Data: Individual interviews  Length of data collection: 3 to 6 months | “We argue that the concept of multimorbidity as biomedically imagined has limited relevance to lived experience, while concurrency may also be erroneous. In response, we outline a lived experience of multiple chronic conditions in later life, which highlights differences between clinical and lay assumptions and makes the latter visible.”  Subheadings: 1) Living with multimorbidity? 2) Lay logics of meaning (Normality and control, Biography and normality, Age and normality, Biomedicine and health professionals) |
| Principi, A., Smeaton, D., Cahill, K., Santini, S., Barnes, H., & Socci, M. (first published 2018). What happens to retirement plans, and does this affect retirement satisfaction? International Journal of Aging & Human Development, 2020, 90(2) 152–175  Country: UK, Italy, and US | "This study examines the role of planning and plan fulfillment for retirement satisfaction using a dynamic resource theory approach." | Qualitative longitudinal research | Population: Older workers (n=111)  Data: Individual interviews  Length of data collection: About 2 years | “Realizing plans was found to be linked to retirement satisfaction. However, many retirees adjusted well to retirement without planning in advance, or when plans were thwarted, and sometimes retirement did not live up to expectations despite fulfillment of plans. Psychological resources and resilience were key dimensions of satisfaction regardless of planning. Retirement satisfaction was also associated with social integration, adoption of new social roles, and opportunities to be active within and beyond the private sphere, such as volunteering or participating in leisure oriented clubs or activities. Regardless of planning, the quality of family relationships was a particularly important element for retirement satisfaction or dissatisfaction.”  Subheadings: 1) Fulfilled planners (Satisfaction, Dissatisfaction), 2) Thwarted planners (Satisfaction, Dissatisfaction), 3) Optimistic drifters (Satisfaction, Dissatisfaction), 4) Anxious drifters (Satisfaction, Dissatisfaction) |
| Pyörälä, E., Mäenpää, S., Heinonen, L., Folger, D., Masalin, T., & Hervonen, H. (2019). The art of note taking with mobile devices in medical education. BMC Medical Education, 19(1), 96-96.  Country: Finland | "...to explore students’ perceptions of the study use of mobile devices and digital note taking practices in the first cohort of tablet computer users during their studies. 1) What were the students’ most important self-reported study uses of mobile devices? 2) How did the note taking practices change over the study years? 3) What were the students’ perceptions of the best practices of note taking with mobile devices?" | Action research | Population: Medical and dental students (n=176)  Data: Online questionnaires including open-ended questions, and focus-group interviews  Length of data collection: About 5 years | “The response rates varied between 73 and 95%. Note taking was the most frequently and consistently reported study use of iPads during the study years. While taking notes, students processed the new information in an accomplished way and personalised the digital learning materials by making comments, underlining, marking images and drawing. The visual nature of their learning materials stimulated learning. Students organised the notes for retention in their personalised digital library. In the clinical studies, medical students faced the teachers’ resistance and ambivalence to mobile device usage. This hindered the full-scale benefit of the novel technology in the clinical context.”  Subheadings: 1) The most important self-reported study uses of mobile devices, Students’ perceptions of the best practices of note taking |
| Ralph, A. F., Butow, P., Craig, J. C., Wong, G., Chadban, S. J., Luxton, G., Gutman, T., Hanson, C. S., Ju, A., & Tong, A. (2019). Living kidney donor and recipient perspectives on their relationship: Longitudinal semi-structured interviews. BMJ Open, 9(4), e026629-e026629.  Country: Australia | "The aim of this study is to collect longitudinal data on donor and recipient expectations and perspectives of their relationship in living kidney donor transplantation, which may inform strategies to mitigate risks of relationship tension and conflict and support relationship resilience, thereby contributing to improved outcomes in living kidney donor transplantation" | Grounded theory | Population: Living kidney donors and their recipients (n=32)  Data: Individual interviews  Length of data collection: 13 to 15 months | “We identified seven themes (with respective subthemes): donation as enacting familial responsibility for care; analytical decision making to mitigate regret (avoiding anticipated regret and maintaining control, removing emotional impulsivity); strengthened interpersonal ties (gaining a deeper appreciation among family members, stronger empathy for each other, improving social participation); instability of relational impacts (anger and aggression threatening dynamics, unanticipated stress and emotional lability, triggering familial tension); renegotiating social roles (unexpected continuation of caregiving responsibilities, inability to relinquish the caregiving role, disappointment with unfulfilled renewal of intimacy, dissatisfaction over discrepant energy levels); guilt over unmet expectations and inevitability of the gift relationship (vague and transient indebtedness, expectation of reciprocity, transferring kidney ownership).”  Subheadings: 1) Analytical decision making to mitigate regret (Avoiding anticipated regret and maintaining control, Removing emotional impulsivity), 2) Donation as enacting familial responsibility for care, 3) strengthened interpersonal ties (Gaining a deeper appreciation among family members, Stronger empathy for each other, Improving social participation), 4) Instability of relational impacts (Anger and aggression threatening dynamics, Unanticipated stress and emotional lability, Triggering familial tension), 5) renegotiating social roles (Unexpected continuation of caregiving responsibilities, Inability to relinquish the caregiving role, Disappointment with unfulfilled renewal of intimacy, Dissatisfaction over discrepant energy levels), 6) Guilt over unmet expectations, 7) Inevitability of the gift relationship (Vague and transient indebtedness, Expectation of reciprocity, Transferring kidney ownership) |
| Ramanaik, S., Collumbien, M., Prakash, R., Howard-Merrill, L., Thalinja, R., Javalkar, P., Murthy, S., Cislaghi, B., Beattie, T., Isac, S., Moses, S., Heise, L., & Bhattacharjee, P. (2018). Education, poverty and "purity" in the context of adolescent girls' secondary school retention and dropout: A qualitative study from Karnataka, southern India. Plos One, 13(9), e0202470-e0202470.  Country: India | "...to investigate gender socialisation [...] We analyse how gender-related norms interact with poverty and family background to result in girls’ drop out from secondary school. In addition, we identify the main facilitators of school retention and modifications to gender performance." | Case study | Population: Adolescent girls (n=36)  Data: Individual interviews  Length of data collection: 16 months | “Our study found that poverty and socioeconomic realities at the household level strongly affect conformity with discriminatory gender practices such as restricting girls’ mobility. The value placed on education by parents clearly differentiates the regular school goers from those frequently absent and others who dropped out. With active encouragement of the girls’ educational and career aspirations, parents engendered the girl’s agency to communicate openly both at home and at school, allowing subtle changes to gender performance while resisting the pressure of social sanctions. In contrast, where educational aspirations were weak, parents invested more intensely in enforcing correct performance of gender, prioritising her well-being by aiming to secure her future in a good marriage. Among poorer families, girls’ domestic duties came at the cost of schooling with concerns about protecting her sexual purity predominating.”  Subheadings: 1) Profile of girls participating in the case studies, 2) How and why girls dropped out of school, 3) Regular school goers, 4) Parental aspirations, 5) Communication and cooperation in familial relationships, 6) Support and communication at school, 7) On the brink of dropout? 8) Economic and household responsibilities, 9) Lack of parental support, 10) Coming of age and marriageability, 11) Negative experiences at school |
| Reed, E., Todd, J., Lawton, S., Grant, R., Sadler, C., Berg, J., Lucas, C., & Watson, M. (2018). A multi-professional educational intervention to improve and sustain respondents' confidence to deliver palliative care: A mixed-methods study. Palliative Medicine, 32(2), 571-580.  Country: UK | "Research question: "Can a multi-professional palliative care education intervention improve and sustain candidates’ confidence to deliver palliative care?" Aims: "•To evaluate the impact of the ECEPC on candidates’ confidence in palliative care; •To determine whether this is sustained over time; •To explore the candidate’s perception of the influence of the course on their confidence in practice." | mixed methods | Population: Candidates undertaking the ECEPC educational intervention (112 answered survey, 15 particiopated in interviews)  Data: Individual interviews and questionnairs  Length of data collection: 6 months | “At 3 months, candidates had almost 20 times higher odds of being above any given level of confidence than at baseline which was sustained at 6 months. Qualitative analysis identified examples of increased competence and confidence improving palliative care delivery.”  Subheadings: 1) Phase 1, 2) Phase 2, 3) Confidence, 4)9 Influencing practice |
| Rehackova, L., Araújo-Soares, V., Steven, S., Adamson, A. J., Taylor, R., & Sniehotta, F. F. (first published 2019). Behaviour change during dietary Type 2 diabetes remission: A longitudinal qualitative evaluation of an intervention using a very low energy diet. Diabetic Medicine, 2020, 37, 953–962.  Country: UK | "To understand the process of behaviour change through the experiences of people with Type 2 diabetes engaged in an 8-month diabetes remission intervention including a 2-month weight loss phase with the use of a very low energy diet (VLED), and a 6-month, structured weight maintenance phase." | not described | Population: People with type 2 diabetes (n=11)  Data: Individual interviews  Length of data collection: 8 months | “Eleven of 18 participants completed all three interviews. The following themes of change were identified in their narratives: (1) ‘Building behavioural autonomy’ as a process of growing confidence to engage in health behaviours that are independent of those of other people; (2) ‘Behavioural contagion’ describing how one’s new health behaviours tend to affect those of other people; (3) ‘From rigid to flexible restraint’, reflecting the changes in attitudes and behaviours required for a successful adaptation from weight loss to weight maintenance; and (4) ‘Shift in identity’, representing changes in the participants’ perceptions of themselves.”  Subheadings: 1) Theme 1: building behavioural autonomy (Behavioural interdependence, Formation of behavioural autonomy), 2) Theme 2: behaviour contagion (Minimizing discrepancy, Unintended benefits for others), 3) Theme 3: From rigid to flexible restraint, 4) Theme 4: shift in identity (Increased awareness, Reflection on past behaviour) |
| Richter Sundberg, L., Garvare, R., & Nyström, M. E. (2017). Reaching beyond the review of research evidence: a qualitative study of decision making during the development of clinical practice guidelines for disease prevention in healthcare. BMC Health Services Research, 17(1), 344-344.  Country: Sweden | "Our focus is on the judgement and decision making process of the Prioritization group in the third phase of the NBHW guideline development model, i.e. prioritizing and deciding on guideline recommendations. Accordingly, the aim of this study was to investigate the bases for decisions and the decision making process of the Prioritization group during development of clinical guidelines with a disease preventive scope in Sweden. Three more specific research questions were posed: I. Which decision making criteria were used, and how did research evidence influence the Prioritization group’s judgment and decision making process? II. Did the composition of decision criteria change over time, and if so, how? III. Did the Prioritization group encounter conflicts or dilemmas during judgement and decision making? If so, on what subjects and how were these conflicts or dilemmas managed?" | case study | Population: Experts in preventive guidelines (n=25)  Data: Observations, open ended questions in questionnaris, and policy documents.  Length of data collection: 3 years | “The guideline development model was modified ad-hoc as the group encountered three main types of dilemmas: high quality evidence vs. low adoptability of recommendation; insufficient evidence vs. high urgency to act; and incoherence in assessment and prioritization within and between four different lifestyle areas. The formal guideline development model guided the decision-criteria used, but three new or revised criteria were added by the group: ‘clinical knowledge and experience’, ‘potential guideline consequences’ and ‘needs of vulnerable groups’. The frequency of the use of various criteria in discussions varied over time. Gender, professional status, and interpersonal skills were perceived to affect individuals’ relative influence on group discussions.”  Subheadings: 1) Decision criteria used by the Prioritization group (Research evidence, Severity of the condition, Cost-effectiveness of the intervention, Ethical considerations, Needs of vulnerable groups, Clinical knowledge and experience, Potential guideline consequences, Additional factors – Gender, social status, and interpersonal skills), 2) The decision making process over time, 3) Emerging dilemmas and related strategies (Dilemma #1 – Evidence versus adoptability, Dilemma #2 – Evidence versus urgency, Dilemma #3 – Inconsistent judgments) |
| Ridder, H.-G., & Schrader, J. S. (2017). Processing of intended and unintended strategic issues and integration into the strategic agenda. Health Care Management Review, 44(4), 332-343.  Country: Germany | "Hence, we ask how intended strategic issues are processed into deliberate strategies and how unintended strategic issues are processed into emergent strategies. Finally, we ask how deliberate and emergent strategies are integrated into the strategic agenda of a hospital." | Case study | Population: Members of a hospital board (n=13)    Data: Observations, documents (e.g., official protocols, internal and external documents), and individual interviews  Length of data collection: 2 years | “Our findings suggest that strategic issues are particularly successful within the strategy formation process if interest groups are concerned with the strategic issue, prospective profits are estimated, and relevant decisions makers are involved early on. Structure and interaction processes require clear criteria and transparent procedures for effective strategy formation.”  Subheadings: 1) Intended strategic issues (Structure, Interaction in communication channels), 2) Unintended strategic issues (Structure, Interaction in communication channels), 3) Strategic agenda |
| Riegel, B., Dickson, V. V., Garcia, L. E., Masterson Creber, R., & Streur, M. (2017). Mechanisms of change in self-care in adults with heart failure receiving a tailored, motivational interviewing intervention. Patient Education and Counseling, 100(2), 283-288.  Country: US | "The aim of this study was to identify the mechanism of intervention effectiveness by elucidating the MI [motivational interviewing] techniques used and the relationship between the techniques and changes in self-care. Combined with our prior pilot work, answering these aims will allow us to develop hypotheses about mechanisms of effectiveness, which can be tested in later studies." | mixed methods | Population: Patients with heart failure (n=8)  Data: Questionnairs, observations (e.g., recorded sessions with motivational interviewing)  Length of data collection: 90 days | “Three MI [motivational interviewing] techniques used were related to improved self-care: 1) reflection and reframing, 2) genuine empathy, affirmation, and humor, and 2) individualized problem solving. These techniques stimulated openness to goal setting, positive self-talk, perceived ability to overcome barriers, and change talk. The mechanisms by which the techniques achieved the desired outcomes were the development of discrepancy and self-efficacy, which are consistent with the principles of MI.”  Subheadings: 1) Quantitative results: self-care change over time, 2) Qualitative results: self-care change over time, 3) Integrated results: self-care change over time, 4) Motivational interviewing techniques (Theme 1: reflection and reframing facilitate positive self-talk and change talk, Theme 2: communication style that included genuine empathy, affirmation, and humor promoted perceived ability to overcome barriers, Theme 3: personalized problem solving stimulated openness to goal setting) |
| Robards, F., Kang, M., Steinbeck, K., Hawke, C., Jan, S., Sanci, L., Liew, Y. Y., Kong, M., & Usherwood, T. (2019). Health care equity and access for marginalised young people: A longitudinal qualitative study exploring health system navigation in Australia. International Journal for Equity in Health, 18(1), 41-41.  Country: Australia | "This longitudinal study explored young people’s journeys through the health system in New South Wales (NSW), Australia, over time. The aim was to understand health system navigation, including the use of technology, for young people belonging to one or more marginalized groups." | Grounded theory & qualitative longitudinal research | Population: Marginalised young people aged 12-24 years (n=41)  Data: Individual interviews  Length of data collection: Up to 12 months | “We interviewed 41 young people at baseline who were living in rural or remote areas, sexuality and/or gender diverse, refugee, homeless, and/or Aboriginal. A retention rate of over 85% was achieved. Nineteen belonged to more than one marginalised group allowing an exploration of intersectionality. General practitioners (family physicians) were the most commonly accessed service throughout the study period. Participants were ambivalent about their healthcare journeys. Qualitative analysis identified five themes: 1. Technology brings opportunities to understand, connect and engage with services 2. Healthcare journeys are shaped by decisions weighing up convenience, engagement, effectiveness and affordability. 3. Marginalised young people perceive and experience multiple forms of discrimination leading to forgone care. 4. Multiple marginalisation makes health system navigation more challenging 5. The impact of health system complexity and fragmentation may be mitigated by system knowledge and navigation support”  Subheadings: 1) Health status, 2) Health care access, 3) Themes (Theme 1: Technology brings opportunities to understand, connect and engage with services, Theme 2: Healthcare journeys are shaped by decisions weighing up convenience, engagement, perceived effectiveness and affordability, Theme 3: Marginalised young people perceive and experience multiple forms of discrimination leading to forgone care, Theme 4: Multiple marginalisation makes health system navigation more challenging, Theme 5: The impact of health system complexity and fragmentation may be mitigated by system knowledge and navigation support) |
| Roberts, D., Calman, L., Large, P., Appleton, L., Grande, G., Lloyd‐Williams, M., & Walshe, C. (2018). A revised model for coping with advanced cancer. Mapping concepts from a longitudinal qualitative study of patients and carers coping with advanced cancer onto Folkman and Greer's theoretical model of appraisal and coping. Psycho-Oncology, 27(1), 229-235.  Country: UK | "Data from a study on coping with advanced cancer are used to explore whether the Folkman and Greer model reflects the coping processes participants used. These data are from a serial interview study designed to answer the following questions: a. What do people do to cope well when living with advanced cancer? b. Why and when do they perceive these coping strategies as effective? c. How can health care professionals support effective coping strategies?" | Qualitative longitudinal research | Population: People with advanced cancer (n=26)  Data: Individual interviews  Length of data collection: 4 to 12 weeks | “Mapping coping strategies clearly onto the problem‐ or emotion‐focused elements of the model proved problematic. Fluctuating symptoms, deterioration over time, and uncertain timescales in advanced cancer produce multiple events simultaneously or in quick succession. This demands not only coping with a single event but also frequent repositioning, often to an earlier point in the coping process. In addition, there is substantial ongoing potential for some degree of distress rather than purely ‘positive emotion’ as the final stage in the process is death with several points of permanent loss of capability in the interim.”  Subheadings: 1) Events, 2) Person characteristics, 3) Appraisal, 4) Coping, 5) Event outcome, 6) Emotion outcome, 7) Meaning‐based coping, 8) Positive emotion |
| Robinson, J., Gott, M., Gardiner, C., & Ingleton, C. (2018). The impact of the environment on patient experiences of hospital admissions in palliative care. BMJ Supportive & Palliative Care, 8(4), 485-492.  Country: New Zealand | "...exploring the impact of the environment on experiences of hospitalizations from the patients͛ perspective." | Critical realism | Population: Patients who met one of the Gold Standard Framework Prognostic Indicators for palliative care need (n=14)  Data: Individual interviews  Length of data collection: Unclear, probably a few days up to a few weeks | “Almost all participants described a range of factors associated with the environment which impacted negatively on their experiences of hospitalization. This included challenges with the physical surroundings, the impact on social relationships with other patients, families and health professionals and the influence of the cultural milieu of the hospital setting.”  Subheadings: No subheadings in result section |
| Rodriguez-Morales, L. (2017). In your own skin: The experience of early recovery from alcohol-use disorder in 12-step fellowships. Alcoholism Treatment Quarterly, 35(4), 372-394.  Country: UK | "The study reported here adopts a longitudinal-single case approach to examine a young adult’s experience of early recovery in 12-Step fellowships. It attempts to understand what it is like to recover from an AUD [Alcohol-Use Disorder] in all its social and personal complexity, while providing an in-depth exploration of the individual psychological transformation." | case study & qualitative longitudinal research | Population: Young adults (n=1)  Data: Individual interviews  Length of data collection: 8 months | “During the participant’s 2 months of recovery, a series of intrapersonal changes were reported concerning issues of self-care and emotional development. Following 6 months in recovery, the participant’s recovery focus then centered on the interpersonal issues related to changes in his social network and ways of relating. By 10 months, the participant’s recovery emphasized aspects of self-actualization, including a sense of spirituality. The findings illustrate how issues of authenticity, emotional expression, and identity transformation are intertwined in the participant’s early recovery.”  Subheadings: 1) Two months recovery: Development of self-care, 2) Six months recovery: Finding new ways of relating and being with others, 3) Ten months recovery: Looking toward the future |
| Rosen, J. G., Clermont, A., Kodish, S. R., Matar Seck, A., Salifou, A., Grais, R. F., & Isanaka, S. (2018). Determinants of dietary practices during pregnancy: A longitudinal qualitative study in Niger. Maternal & Child Nutrition, 14(4), 1-1.  Country: Niger | "This paper presents findings from a longitudinal qualitative study in south‐central Niger exploring maternal food consumption practices and their underlying determinants during pregnancy." | Grounded theory & qualitative longitudinal research | Population: Pregnant women, household members, and health workers (n=140)  Data: Individual interviews, and focus group interviews  Length of data collection: 5 months | “Participants categorized foods into 4 primary dietary taxonomies when discussing ideal maternal diets but cited constraints related to accessibility and availability impeding routine consumption of these foods. Perceptions of ‘modern,’ urban foods as healthy, coupled with key structural barriers such as food costs, were identified. Maternal morbidity influenced food consumption, as women reported reducing food intake early in pregnancy in response to illness episodes. Although awareness of optimal foods for supporting healthy pregnancieswas moderately high, somemisconceptions were observed and multilevel barriers to food security restricted opportunities for consuming these foods.”  Subheadings: 1) Typical and ideal diets during pregnancy, 2) Constraints to ideal maternal diet (Structural/community: Food availability, Household: Food access, Physiological: Pregnancy illness and food aversions) , 2) Changes in pregnancy illnesses over time |
| Rosenberg, A., Heimer, R., Keene, D. E., Groves, A. K., & Blankenship, K. M. (2019). Drug treatment accessed through the criminal justice system: Participants' perspectives and uses. Journal of Urban Health, 96(3), 390-399.  Country: US | "Given the existing literature on the treatment of addiction, we focus on non-addictionrelated narratives to understand the broader role drug treatment plays in the lives of justice-involved people." | not described | Population: Participants who were recently released from prison or jail and convicted of a non-violent drug-related crime (n=45)  Data: Individual interviews  Length of data collection: About 2 years | “Many participants who were referred to drug treatment did not consider these programs appropriate for their needs, as many did not perceive themselves to have a drug problem, or did not consider substance use to be their primary problem. Frustrations regarding the ill fitting nature of mandated programs were coupled with theories about non-health-related policy goals of criminal justice-mandated drug treatment, such as prison over flow management and increased profit for the state. None the less, participants used drug treatment to advance their own goals of coping with life’s challenges, reducing their criminal justice system involvement, proving worthiness through rehabilitation, and accessing other resources.”  Subheadings: 1) Participants’ perspectives on criminal justice-related drug treatment , 2) Drug treatment referrals as common, 3) Perceived need for drug treatment, 4) Frustration from lack of self-determination, 5) Reasons behind linkages between the criminal justice and drug treatment systems, 6) Use of drug treatment to pursue non-addiction related goals, 7) Enhanced understanding and coping skills, 8) Reducing criminal justice system involvement, 9) Accessing other resources while participating in drug treatment programs, 10) Proving worthiness through rehabilitation |
| Ross, V., Kõlves, K., Kunde, L., & De Leo, D. (2018). Parents' experiences of suicide-bereavement: A qualitative study at 6 and 12 months after loss. International Journal of Environmental Research and Public Health, 15(4).  Country: Australia | "...to examine the individual experiences of both mothers and fathers bereaved by suicide over time, specifically at the six month and 12 month time points after the death of their child." | not described | Population: Mothers and fathers bereaved by suicide (n=14)  Data: Individual interviews  Length of data collection: 6 months | “Generic qualitative analysis identified three key themes: searching for answers and sense-making, coping strategies and support, and finding meaning and purpose. Some participants showed indications of meaning-making and post-traumatic growth at 12 months after the suicide. According to the dual process model of bereavement, it is likely that participants were still oscillating between sense-making and meaning making, indicating that adapting to bereavement is a dynamic and fluctuating process.”  Subheadings: 1) Three types of pretend fingerspelling (Pre-fingerspelling, Play-fingerspelling, Invented fingerspelling), 2) Part of sign language (Consolidating a name or a word, Private speech, Fingerspelling to demonstrate status, Fingerspelling to exclude a playmate) |
| Rosser, E. A., Scammell, J., Heaslip, V., White, S., Phillips, J., Cooper, K., Donaldson, I., & Hemingway, A. (2019). Caring values in undergraduate nurse students: A qualitative longtitudinal study. Nurse Education Today, 77, 65-70.  Country: UK | "...the purpose of this paper is to report on the final phase of a five-phase case study which uses a prospective qualitative longitudinal approach to understand the beliefs and values of caring, held by student nurses from the day of entry through their education programme to completion" | Case study & Qualitative longitudinal research | Population: Nursing students (n=14)  Data: Individual interviews and focus group interviews  Length of data collection: About 3 years | “Data were analysed using thematic analysis with four themes emerging: i) Articulating the terms caring and dignity ii) Recognising the need for individualisation iii) Learning nursing and iv) Personal journey.”  Subheadings: 1) Articulating the meaning of the terms ‘caring’ and ‘dignity’ (The meaning of the term ‘caring’, The meaning of the term ‘dignity’), 2) Recognising the need for individualisation, 3) Learning nursing, 4) Personal journey |
| Rulifson, G., & Bielefeldt, A. R. (2019). Evolution of students' varied conceptualizations about socially responsible engineering: A four year longitudinal study. Science and Engineering Ethics, 25(3), 939-974.  Country: US | "This study aims to develop a better understanding of how the college experience influences students’ ideas about SRE [socially responsible engineering]. Weidman’s updated Inputs–Environment– Outputs (I–E–O) model of undergraduate socialization (Weidman was used as framework). RQ1: How did students’ pre-college experiences impact their views of socially responsible engineering? RQ2: What are the main influences that shaped evolving ideas about socially responsible engineering during students’ 4 years of college? RQ3: How do undergraduate engineering students change in the ways that they understand socially responsible engineering during college?" | not described | Population: Engineering students (n=21)  Data: Individual interviews and questionnairs  Length of data collection: About 3 years | “Using the Weidman Input–Environment–Output model as a framework, this research found that influences included required classes such as engineering ethics, capstone design, and some technical courses, pre-college volunteering and familial values, co-curricular groups such as Engineers Without Borders and the Society of Women Engineers, as well as professional experiences through internships. Further, some experiences such as technical courses and engineering internships contributed to confine students’ understanding of an engineer’s social responsibility. Overall, students who stayed in engineering tended to converge on basic responsibilities such as safety and bettering society as a whole, but tended to become less concerned with improving the lives of the marginalized and disadvantaged. Company loyalty also became important for some students.”  Subheadings: 1) RQ1: Elements that shaped students’ incoming attitudes, 2) RQ2: Influences during college, 3) EQ3: Changes in how students interpret socially responsible engineering, 4) Synthesized I–E–O model, 5) Student 1: Julie, 6) Julie: inputs, 7) Julie: environment, 7) Julie: outcomes, 8) Student 2: Kim, 9) Kim: inputs, 10) Kim: environment, 11) Kim: outcomes, 12) Student 3: Nathan, 13) Nathan: inputs, 14) Nathan: environment, 15) Nathan: outcomes, 16) Comparison of Julie, Kim, and Nathan |
| Ryba, T. V., Stambulova, N. B., Selänne, H., Aunola, K., & Nurmi, J.-E. (2017). “Sport has always been first for me” but “all my free time is spent doing homework”: Dual career styles in late adolescence. Psychology of Sport & Exercise, 33, 131-140.  Country: Finland | "In this research, we conceptualised dual career as a story that young people tell about their engagement at sport and school (see also Savickas, 2011) to examine three research questions: (a) How and to what extent do adolescent Finnish athletes narrate and integrate their autobiographical events in sport and education into identity narrative?, (b) How and to what extent are sport and education integrated in the adolescent athletes' projected future?, and (c) What does the relationship between one's narratives ofthe past and narratives ofthe future reveal about their dual career style?" | Qualitative longitudinal research | Population: Young athletes (n=18)  Data: Individual interviews  Length of data collection: 6 months | “Thirteen of 18 adolescent athletes drew primarily on the performance narrative plot to construct their life story and five of 18 athletes could not project into the future beyond their athletic selves. We identified three styles of athletes’ career construction. Employing musical terminology as a metaphor, the contrapuntal style entwines sport and education as harmonically related life-themes; monophonic style draws on a prominent athletic life-theme; and dissonant style is underpinned by discord of sport and education. We did not detect direct associations between narrative types (performance, discovery and relational) and career construction styles. We show the dominant style development within an exemplary story.”  Subheadings: 1) Overview of the findings, 2) Unelma's story (Developing an identity storyline: “I have chosen sports and that is what I want to do”, Exploring the future: “That’s not my goal to be best in everything”) |
| Sarkar, D., Murphy, H., Fisseha, T., Koroma, A. S., Hodges, M. H., Adero, N., Ngalombi, S., Nabakooza, J., Wun, J., & Namaste, S. M. L. (2018). Understanding the process of strengthening multi-sectoral efforts for anemia reduction: Qualitative findings from Sierra Leone and Uganda. The International Journal of Health Planning and Management, 33(4), 1024-1044.  Country: Sierra Leone and Uganda | "...explore country experiences developing a multi‐sectoral anemia platform and strategyIn this paper, we present key findings and lessons learned from SPRING's documentation in Sierra Leone and Uganda, along with similarities and variations across the 2 settings, to inform future global and country multi‐sectoral anemia planning efforts." | Case study | Population: NAWG-members (n=25)  Data: Individual interviews  Length of data collection: 11 to 24 months (differed between sites) | “Similar factors were identified in the 2 countries. Setting the agenda was an important first step, accomplished by using country‐specific anemia‐related data and obtaining multi‐sectoral commitment. Establishment of a cohesive coordination structure provided an effective platform to prioritize and align anemia activities. Strong, committed leadership and representation of diverse stakeholders was essential to maintain the legitimacy of anemia efforts. The main barriers to the policy‐making process included misalignment of sectoral mandates, differences in work cultures, as well as competing priorities and increased staff workload.”  Subheadings: 1) Agenda setting (Understanding the problem and raising awareness, Multi‐sectoral commitment), 2) Policy formulation (Cohesive coordination structure, Prioritization and alignment), 3) Legitimation (Leadership, Stakeholder participation), 4) Implementation considerations (Funding, Accountability) |
| Schiltz, J., Derluyn, I., Vanderplasschen, W., & Vindevogel, S. (2019). Resilient and self‐reliant life: South Sudanese refugees imagining futures in the adjumani refugee setting, Uganda. Children & Society, 33(1), 39-52.  Country: Uganda | "This article analyses how South Sudanese refugee youngsters in Uganda imagine and act towards their futures in a humanitarian space that aims for refugees to become resilient and self-reliant. […] In examining what happens when youngsters become acquainted with the refugee policy and the options for the future that are available to them and explores what such policies can mean within the permanent temporariness of the camp." | the methodological approach of bricolage & qualitative longitudinal research | Population: Young South Sudanese refugees (n=30)  Data: Individual interwiews, informal conversations, and observations  Length of data collection: About 2 years | “Youngsters need to become accustomed to a future without substantial progress, or be ready to play the game of chance. As such, a resilience and self-reliance policy not only reveals the powerlessness of refugee youth, but also the limits of a humanitarian project to seek actual solutions to refugee situations.”  Subheadings: 1) Two narratives of camp life and future (Deng – “Everything will be possible”, Evelyn – ‘if you cannot afford it, then you leave it’) |
| Schmid-Mohler, G., Caress, A.-L., Spirig, R., Benden, C., & Yorke, J. (2019). "Thrust out of normality"-How adults living with cystic fibrosis experience pulmonary exacerbations: A qualitative study. Journal of Clinical Nursing, 28(1), 190-200.  Country: Switzerland | "The aim of this study was to explore the experience of adults with CF [cystic fibrosis] during a pulmonary exacerbation over time." | not described | Population: People with Cystic Fibrosis (n=18)  Data: Individual interviews and field notes  Length of data collection: 3-4 weeks | “Patients (11 men and 7 women; median age 29.5 years, range 19–55 years; median FEV_1_ [forced expiratory volume in 1‐s] 45%, range FEV_1_ 23%–105%) experienced pulmonary exacerbations as disruptions of their normality, which led to a substantial increase in their emotional distress. Exacerbations represented a period of threat and domination by CF [cystic fibrosis]; that is, symptoms and treatment consumed energy, restricted physical activity and daily life roles. ‘Noting change,’ ‘waiting until antibiotics help,’ ‘returning to normality’ and ‘establishing a new normality’ characterised their descriptions of the pulmonary exacerbation trajectory. Emotional distress was the major driver for patients’ selfmanagement, and personal goals and illness beliefs influenced also patients’ selfmanagement decisions.”  Subheadings: 1) Characteristics of the Sample, 2) The pulmonary exacerbation trajectory, 3) Noting a change, 4) Waiting until the antibiotics help, 5) Returning to normality, 6) Establishing (new) normality |
| Schröder, S. L., Fink, A., & Richter, M. (2018). Socioeconomic differences in experiences with treatment of coronary heart disease: A qualitative study from the perspective of elderly patients. BMJ Open, 8(11), e024151-e024151.  Country: Germany | "...to identify socioeconomic differences in the patient’s perspective and their experiences with the treatment pathway for CHD [coronary heart disease] in all sectors from therapy to aftercare." | Grounded theory & qualitative longitudinal research | Population: Patients with a confirmed diagnosis of CHD (n=41)  Data: Individual interviews  Length of data collection: 6 months | “From various patient’s experiences along the pathway of care which were found to differ according to SES [socioeconomic status] we derived three major themes: (1) information: patients with higher SES had greater knowledge about treatment and could use medical records as sources of information; (2) illness perception: patients with lower SES focused on improving symptoms and survival, while patients with higher SES focused on physical performance and disease management; and (3) perceived role in healthcare: patients with lower SES tended to delegate responsibility to healthcare professionals.”  Subheadings: 1) Treatment at the hospital, 2) Cardiac rehabilitation, 3) Treatment with drugs, 4) Ambulatory aftercare, 5) Factors and mechanisms of socioeconomic differences in CHD treatment |
| Serholt, S. (2018). Breakdowns in children's interactions with a robotic tutor: A longitudinal study. Computers in Human Behavior, 81, 250-264.  Country: Sweden | "The aim of this paper is to explore the challenges that currently exist when moving robotic tutors into actual classrooms by focusing specifically on breakdowns in children's interactions with a robotic tutor at their school. Video recordings of such instances are analyzed in-depth through qualitative methods, guided by the following research question: What causes break-downs in children's interactions with a robotic tutor, and what consequences do such breakdowns pose for the educational use of robots?" | not described | Population: School children (n=6)  Data: Observations, brief informal interviews, and questionnaires  Length of data collection: 13 weeks | “The results comprise four themes to explain why children's interactions with the robotic tutor break down: (1) the robot's inability to evoke initial engagement and identify misunderstandings, (2) confusing scaffolding, (3) lack of consistency and fairness, and finally, (4) controller problems.”  Subheadings: 1) The robot's inability to evoke initial engagement and identify misunderstandings, 2) Confusing scaffolding, 3) Lack of consistency and fairness, 4) Controller problems (Problems with the touch-interface and false signifiers, Problems with the robot) |
| Sestito, S. F., Rodriguez, K. L., Saba, S. K., Conley, J. W., Mitchell, M. A., & Gordon, A. J. (2017). Homeless veterans’ experiences with substance use, recovery, and treatment through photo elicitation. Substance Abuse, 38(4), 422-431.  Country: US | "We conducted a secondary qualitative analysis aimed at describing H-PACT veterans’ experiences with substance use (SU), substance use recovery (SUR), and substance use treatment (SUT)." | not described | Population: Homeless veterans (n=15)  Data: Individual interviews, and photovoice  Length of data collection: 1 month | “The majority of participants (75%, n=15) discussed SU [substance use], SUR [substance use recovery], and/or SUT [substance use treatment] in regards to their health and health care utilization. SU themes centered on disclosure of addiction or dependency; substances used; repercussions of SU; SU as a coping mechanism; and association of SU with military service. SUR themes included disclosure of length of sobriety; perceived facilitators of SUR in health, beliefs, social, environmental, financial, and creative pursuit domains; and perceived barriers to SUR in beliefs, social, and environmental domains. SUT themes focused on perceived facilitators of SUT in access to Department of Veterans Affairs (VA) and non-VA services and social domains and perceived barriers to SUT in the social domain.”  Subheadings: 1) Participant characteristics, 2) Interview analysis, 2.1) Substance use, (Disclosure of addiction or dependency, Substance used referenced, Repercussions of substance use, Substance use as coping mechanism, Association of substance use with military service), 2.2) Substance use recovery (Disclosure of length of sobriety), 2.3) Facilitators of SUR (Health, Beliefs, Environmental, Financial, Creative pursuit), 2.4) Barriers to SUR (Beliefs, Social, Environmental), 2.5) Substance use treatment, 2.6) Facilitators of SUT (Access to VA and non-VA services, Social), 2.6) Barriers to SUT (Social) |
| Shelton, R. C., Charles, T.-A., Dunston, S. K., Jandorf, L., & Erwin, D. O. (2017). Advancing understanding of the sustainability of lay health advisor (LHA) programs for African-American women in community settings. Translational Behavioral Medicine, 7(3), 415-426.  Country: US | "...to understand factors that influence the long-term implementation, sustainability, and impact of this community-engaged LHA [lay health advisor] program within African- American communities. This paper seeks to (1) advance understanding of barriers and facilitators to the long term implementation and sustainability of community-engaged and community-based LHA programs, including factors that impact the participation and retention of LHAs, and (2) document the impact of LHA programs on women who serve as LHAs and more broadly in African-American communities." "The goal of the qualitative data, presented here, was to inform a more in-depth and comprehensive understanding of factors that influence long-term NWP [National Witness Project] program implementation and sustainability, and to increase the likelihood we had identified the full range of factors, including organizational and contextual factors that were not measured in the quantitative study." | mixed methods | Population: African-American lay health advisors (n=76)  Data: Individual interviews  Length of data collection: 12 to 18 month | “Qualitative data provides insight into inner and outer contextual factors (e.g., community partnerships, site leadership, funding), implementation processes (e.g., training), as well as characteristics of the intervention (e.g., perceived need and fit in African American community) and LHAs [lay health advisors] (e.g., motivations, burnout) that are perceived to impact the continued implementation and sustainability of NWP [National Witness Project]. Factors at the contextual levels and related to motivations of LHAs are critical to the sustainability of LHA programs.”  Subheadings: Qualitative themes, 1) Factors influencing continued NWP implementation and sustainability, 2) The broader impact of NWP on LHAs and their communities |
| SmithBattle, L. (2019). Housing trajectories of teen mothers and their families over 28 years. The American Journal of Orthopsychiatry, 89(2), 258-267.  Country: US | "The specific aims of this substudy were to describe the housing trajectories of teen mothers over 28 years and to explore how their housing trajectories were shaped by family resources, housing programs, and discrimination." | Phenomenology & qualitative longitudinal research | Population: Teen mothers and their family (n=18)  Data: Individual interviews  Length of data collection: 28 years | “Housing instability was pervasive for Black mothers who were disadvantaged as children. Their stories included many moves, doubling up, sending children to live with others, depression, unreliable partners, and racism. Housing instability was a source of toxic stress, interrupted personal and family goals, undermined family routines, and reflected a legacy of discriminatory housing policies. In contrast, White advantaged mothers were stably housed as children and adults, which reflected longstanding family resources, stable marriages, husbands’ steady employment, and tax deductions for homeownership.”  Subheadings: 1) “We Want to Pass This On to Our Kids”, 2) House of Cards: “I’m Getting Ahead On My Own”, 3) House of Cards: “I’ve Been On the Move” |
| Solomon, P., O'Brien, K. K., Nixon, S., Letts, L., Baxter, L., & Gervais, N. (2018a). Qualitative longitudinal study of episodic disability experiences of older women living with HIV in Ontario, Canada. BMJ Open, 8(4), e021507-e021507.  Country: Canada | "...to examine the disability experiences of older women living with HIV over time." | Qualitative longitudinal research | Population: Women aged 50 years or older living with HIV for more than 6 years (n=10)  Data: Individual interviews  Length of data collection: 20 months | “Two major themes related to the episodic nature of the women’s disability. Women were living with multiple and complex sources of uncertainty over time including: unpredictable health challenges, worrying about cognition, unreliable weather, fearing stigma and the effects of disclosure, maintaining housing and adequate finances, and fulfilling gendered and family roles. Women describe strategies to deal with uncertainty over time including withdrawing and limiting activities and participation and engaging in meaningful activities.”  Subheadings: 1) Living with multiple and complex sources of uncertainty over time, 1:1) Unpredictable health challenges, 1:2) Worrying about cognition, 1:3) Unreliable weather, 1:4) Fearing stigma and the effects of disclosure, 1:5) Maintaining housing and adequate finances, 1:6) Fulfilling gendered and family roles, 2) Dealing with uncertainty over time, 2:1) Withdrawing and limiting activities and participation, 2:2) Engaging in meaningful activities |
| Solomon, P., O'Brien, K. K., Nixon, S., Letts, L., Baxter, L., & Gervais, N. (2018b). Trajectories of episodic disability in people aging with HIV: A longitudinal qualitative study. Journal Of The International Association of Providers of AIDS Care, 17, 1-7.  Country: Canada | "The purpose of this study was to understand how the episodic nature of HIV and the associated uncertainty shape the disability experience of adults aging with HIV over time." | Qualitative longitudinal research | Population: Older adults living with HIV (n=24)  Data: Individual interviews  Length of data collection: 20 months | “Longitudinal analyses of the transcribed interviews identified 4 phenotypes of episodic disability over time: decreasing, increasing, stable, or significant fluctuations. Although all participants experienced uncertainty, acceptance and optimism were hallmarks of those whose phenotypes were stable or improved over time.”  Subheadings: 1) Trajectories of episodic disability, 1:1) Decreasing disability over time (Example of decreasing disability over time), 1:2) Increasing disability over time (Example of increasing disability over time), 1:3) Stable over time (Example of stable over time), 1:4) Significant fluctuations in disability over time (Example of significant fluctuations (improvements and deterioration) in disability over time) |
| Stapley, E., Target, M., & Midgley, N. (2017). The journey through and beyond mental health services in the United Kingdom: A typology of parents' ways of managing the crisis of their teenage child's depression. Journal Of Clinical Psychology, 73(10), 1429-1441.  Country: UK | "...exploring the patterns or ideal types of parental experiences that could exist over time among parents of adolescents diagnosed with depression. Specifically, our study sought to create a typology of parents’ ways of managing and dealing with the crisis of their teenage child’s depression over a 2-year period, starting at the point of their child’s referral to CAMHS [child and adolescent mental health services] in the United Kingdom and diagnosis of depression, and then after their child had received treatment at CAMHS, and finally one year later." | not described | Population: Parents of adolescents diagnosed with depression (n=33)  Data: Individual intewrview  Length of data collection: About one year | “Three distinct types or patterns of parental experience were identified: the learning curve parents, the finding my own solutions parents, the stuck parents.”  Subheadings: 1) Ideal type 1: The learning curve parent (Ideal type description, Optimal case), 2) Ideal type 2: The finding my own solutions parent (Ideal type description, Optimalcase), 3) Ideal type 3: The stuck parent (Ideal type description, Optimal case), 4) Demographic data |
| Stephens, G. C., Rees, C. E., & Lazarus, M. D. (2019). How does donor dissection influence medical students' perceptions of ethics? A cross-sectional and longitudinal qualitative study. Anatomical Sciences Education, 12(4), 332-348.  Country: Australia | "This research aimed to identify any areas of interplay between anatomy and medical ethics curricula to ultimately inform development of an evidence-based integrated anatomy and bioethics curriculum. The following exploratory research question was addressed: What are the impacts (if any) of donor dissection on students’ perceptions of medical ethics?" | Qualitative longitudinal research | Population: Medical students (n=207)  Data: Data from online discussion forums, individual interviews and focus group interviews  Length of data collection: 18 months | “Framework analysis identified five themes related to ethics in an anatomical education context: (1) Dignity, (2) Beneficence, (3) Consent, (4) Justification for versus the necessity of dissection, and (5) Dichotomy of objectification and personification. The dominant themes of students’ ethical perceptions changed with time, with a shift from focusing on donors as people, toward the utility of donors in anatomy education. Additionally, themes varied by student demographics including gender, ancestry, and religiosity.”  Subheadings: 1) Student participation, 2) Student demographics, 3) Themes, 4) Dignity, 5) Beneficence, 6) Consent, 7) Justification for versus the necessity of dissection, 8) Dichotomy of objectification and personification, 9) Thematic patterns with demographic factors (Cross-sectional), 10) Temporal thematic changes (Longitudinal) |
| Stevens, M. (2018). Parents' experiences of services addressing parenting of children considered at-risk for future antisocial and criminal behaviour: A qualitative longitudinal study. Children & Youth Services Review, 95, 183-190.  Country: UK | "The study aimed to explore the ways in which families with children identified by services as at risk of future criminal or antisocial behaviour benefit, or do not, from services' intervention, in the longer term. focussing on the perspective of parents, while also including the perspectives of practitioners working [...] The broad objectives were as follows: To explore with primary carers their experiences of interactions with services, and with practitioners their experiences of working with the families • To understand which aspects of families' lives participants felt best responded to service provision • To understand which aspects of service provision were viewed as helpful or unhelpful by primary carers and practitioners." | Qualitative longitudinal research | Population: High need families and practitioners (n=32)  Data: Individual interviews  Length of data collection: About 5 years | “The analysis suggested a number of changes in parenting which appeared to be related to improved outcomes for children and their families. For example, changes in mothers' conceptualisation of their child's behaviour, brought about through therapeutic intervention, could transform parent-child relationships and thereby improve longer-term outcomes. However, other mothers could bring about change without these cognitive shifts, through use of strategies to manage children's behaviour or improve mothers' own wellbeing. Services sometimes played an important role in these changes. The analysis also suggested features of provision which prevented intervention with families being effective.”  Subheadings: 1) Changing parenting to improve family functioning and children's behaviour (Addressing mothers' interpretations of their child's behaviour, Learning strategies to help manage children's behaviour, Addressing mothers' own wellbeing and recognising the impact of parents' personal and mental health histories), 2) Features ofintervention which help bring about change and, conversely, features of intervention which prevent families benefitting (Features of effective parent-practitioner relationships: trust and shared purpose, Barriers to building trust Surveillance and a focus on trivial issues, Reform versus support) |
| Storholm, E. D., Ober, A. J., Hunter, S. B., Becker, K. M., Iyiewuare, P. O., Pham, C., & Watkins, K. E. (2017). Barriers to integrating the continuum of care for opioid and alcohol use disorders in primary care: A qualitative longitudinal study. Journal of Substance Abuse Treatment, 83, 45-54.  Country: US | "The objective of this study was to use an organizational capacity framework to examine perceived barriers to implementing the continuum of care for OAUD [opioid and alcohol use disorders] in a community-based primary care organization over three time points: pre-implementation (preparation), early implementation (practice), and full implementation." | Qualitative longitudinal research | Population: Clinic administrators and medical and mental health providers  Data: Individual interviews, and focus group interviews  Length of data collection: About 3 years | “Barriers were organized by type and size, and are presented over the three time points. Although some barriers persisted, most barriers decreased over time, and respondents reported feeling more efficacious in their ability to successfully deliver OAUD [opioid and alcohol use disorders] treatment. Findings contribute to the needed literature on building capacity to implement OAUD treatment in primary care and suggest that while barriers may be sizable and inevitable, successful implementation is still possible.”  Subheadings: 1) Characteristics of participants, 2) Overview of qualitative results, 4) Constructs, themes, and barriers, 4:1) Construct 1: Fiscal and economic resources (Theme: Billing/cost of services, Theme: Time), 4:2) Construct 2: Workforce and human resources (Theme: Motivation, Theme: Self-Efficacy, Theme: Training, Theme: Staff configuration, Theme: Quantity of staff), 4:3) Construct 3. Physical infrastructure (Theme: Physical space, Theme: Equipment/supplies), 4:4) Construct 4. Intra-organizational and inter-organizational relationships (Theme: Administration and clinic staff collaboration, Theme: Need for referral network), 4:5) Construct 5. Data and informational resources (Theme: Electronic health record:), 4:6) Construct 6. System Boundaries and Size (Theme: Clinic location and attributes, Theme: Population demographics), 4:7) Construct 7. Governance and decision making structure (Theme: Policies and procedures), 4:8) Construct 8. Organizational culture (Theme: Fit with mission values, Theme: Leadership) |
| Sukhera, J., Milne, A., Teunissen, P. W., Lingard, L., & Watling, C. (2018). Adaptive reinventing: implicit bias and the co-construction of social change. Advances in Health Sciences Education, 23(3), 587-599.  Country: Canada | "...to explore how individuals and workplace learning environments influence each other to produce change following a learning activity that disrupts workplace norms. We hoped to draw out how the process of bringing implicit biases into conscious awareness influenced workplace learning and how implicit bias recognition and management could potentially shape workplace transformation." | Case study and grounded theory | Population: Physicians and nurses (n=14)  Data: Individual interviews  Length of data collection: 12 months | “Our results suggest that implicit bias recognition provokes dissonance among participants leading to frustration, and critical questioning of workplace constraints. Once awareness is triggered, participants began reflecting on their biases and engaging in explicit behavioural changes that influenced the perception of structural changes within the learning environment itself.”  Subheadings: 1) Awareness triggers frustration, highlighting workplace constraints, 2) Once bias is recognized, participants adapt through explicit changes in behaviour, 3) Behaviour changes are reinforced through communication, collaboration and role modeling among peers, 4) When confidence increases, some individuals become affordances |
| Superdock, A., K., Barfield, R. C., Brandon, D. H., & Docherty, S. L. (2018). Exploring the vagueness of Religion & Spirituality in complex pediatric decision-making: a qualitative study. BMC Palliative Care, 17(1), 107-107.  Country: US | "...to illuminate the influence of R&S [Religion & Spirituality] on parental decision-making and explore how providers interact with parents for whom R&S are important." | Case study & qualitative longitudinal research | Population: Parents and health care providers of infants with complex life-threatening conditions (n=136)  Data: Individual interviews, field notes, questionnaires, and data from medical charts  Length of data collection: Between 8 days and 531 days (median = 380 days, mean = 324 days, SD = 174 days) | “Parents from 13 cases reported R&S [Religion & Spirituality] directly influenced decision-making. Most HCPs [health care providers] were unaware of this influence. Fifteen R&S themes appeared in parent and HCP transcripts. Themes most often associated with decisionmaking were Hope & Faith, God is in Control, Miracles, and Prayer. Despite instability in the child’scondition, these themes remained consistently relevant across the trajectory of illness. R&S influenced decisions about treatment initiation, procedures, and life-sustaining therapy, but the variance in effect of R&S on parents’ choices ultimately depended upon other medical & non-medical factors.”  Subheadings: 1)Values and beliefs (Faith & hope (Theme 1), God (Themes 2–3), Miracles/divine intervention (Theme 4), Meaning of suffering (Theme 5), Life & death (Themes 6–7)), 2) Practices (Prayer (Theme 8), 3) People (Communities (Theme 11), Faith leaders (Theme 12)), 5) Emotions (Gratitude (Theme 13), Growth (Theme 14), Anger (Theme 15)) |
| Sussman, T., & Orav-Lakaski, B. (first published 2018). "I didn't even make my bed": Hospital relocations and resident adjustment in long-term care over time. The Gerontologist, 2020, 60(1), 32–40.  Country: Canada | "... experiences with the relocation process to Long Term Care from hospital over time. More specifically, the study sought to: (a) describe the relocation trajectories of older adults moving to LTC [long term care] from hospital and (b) identify the conditions that help or hinder older adults’ capacities to adjust to LTC when relocating in the context of multiple moves." | Grounded theory & qualitative longitudinal research | Population: Older adults (n=9)  Data: Individual interviews  Length of data collection: Up to 2 years | “A total of nine residents participated in 24 interviews and most had undergone two or more moves. Analysis of the data revealed that multiple forced moves challenged residents’ capacities to find home in LTC [long term care] by contributing to anxiety and uncertainty, hindering resident–staff relations, and interfering with residents’ desires to connect with one another. Analysis of the data further revealed that relocation frameworks may require adaptations to more accurately reflect older adults’ experiences with relocation from hospital to LTC.”  Subheadings: 1) At home, out of place, or somewhere in between, 2) Forced moves can challenge residents’ capacities to feel at home in LTC, 3) Personalized care supported a sense of place over time, 4) Resident to resident interactions could support or hinder a sense of place |
| Tafvelin, S., Isaksson, K., & Westerberg, K. (2018). The first year of service: A longitudinal study of organisational antecedents of transformational leadership in the social service organisations. British Journal of Social Work, 48(2), 430-448.  Country: Sweden | "The purpose of this longitudinal interview study is to explore organisational antecedents to leadership behaviour among first line managers in social service organisations. Using transformational leadership theory as a framework, we first investigate what kind of leadership the managers are striving to achieve in terms of leadership ideals in their first year of service. By following managers over time, we then aim to capture organisational and working conditions that may hinder managers from performing this leadership ideal in the realm of their everyday life." | not described | Population: First line managers in social service organisations (n=8)  Data: Individual interviews  Length of data collection: 12 months | “(…) the results revealed that the managers strived for a transformational leadership, but several factors in the organisation made it difficult to lead in the way they intended. Hindering factors were identified both at the organisational level, such as ‘top-down management’, ‘financial strain’ and ‘continuous change’, and in the managers’ own working environment in terms of ‘no support’, ‘high work-load’, ‘limited influence’, ‘administrative tasks’ and ‘distance to employees’.”  Subheadings: 1) Leadership ideals during the first year of service, 2) Hindering factors in the organisation, 3) The perceived relationship between hindering factors and leadership ideals over time |
| Taylor, R. D., Carson, H. J., & Collins, D. (2018). The impact of siblings during talent development: A longitudinal examination in sport. Journal of Applied Sport Psychology, 30(3), 272-287.  Country: UK | "Therefore, a 1-year longitudinal study was conducted to examine the impact of siblings on TD [talent development] where both are prevalent within the talent pathway. Interviews were conducted with siblings and parents with the aim of further enhancing our knowledge of the impact of this subsystem on the development process." | case study | Population: Siblings and parents (n=13)  Data: Joint interviews  Length of data collection: One year | “Findings revealed several themes (regularity of interaction, emotional interpersonal skills, rivalry, resilience, and separation) aligning with previous studies, alongside two new themes: communication and skill development.”  Subheadings: 1) Extended relationship dynamics (Skill development, Communication), 2) Temporal nature of the sibling subsystem, 3) Contrasts across families |
| Ten Hoeve, Y., Kunnen, S., Brouwer, J., & Roodbol, P. F. (2018). The voice of nurses: Novice nurses' first experiences in a clinical setting. A longitudinal diary study. Journal of Clinical Nursing, 27(7), e1612-e1626.  Country: The Netherlands | "The current longitudinal study has an open character and focuses on novice nurses’ lived experiences in a clinical setting to gain a greater understanding of which demands from personal and professional life they are confronted with, and what can be done to improve the transition from being a novice to a professional staff nurse. With lived experiences, we refer to the description of and reflections on weekly experiences of novice nurses during their first 2 years on a clinical ward after graduation as a nurse" | phenomenology & qualitative longitudinal research | Population: Novice nurses (n=18)  Data: Diaries  Length of data collection: 2 years | “Eight major themes emerged from the diaries (n = 580): relatedness, competence, development, organisational context, existential events, goals, autonomy and fit. This study revealed that the need for relatedness was by far the most reported theme. Support and positive feedback from colleagues appeared to be crucial for novices starting work in a highly complex environment.”  Subheadings: 1) Background characteristics, 2) Themes and subthemes, 3) Experiences, 4) Relatedness (Sharing experiences with colleagues and supervisors), 5) Competence, 6) Development, 7) Organisational context, 8) Existential events, 9) Goals, 10) Autonomy, 11) Fit |
| Testoni, I., Francescon, E., De Leo, D., Santini, A., & Zamperini, A. (2019). Forgiveness and blame among suicide survivors: A qualitative analysis on reports of 4-year self-help-group meetings. Community Mental Health Journal, 55(2), 360-368.  Country: Italy | "The main objective of this research was to verify whether the forgiveness process spontaneously appear in a self-help group composed by suicide survivors and how it matches with blame processes. In fact, following the perspective of Supiano (2012) and of Lee et al. (2015), we wanted to analyze how the different types of causal attributions, self or other blame and responsibility, intervene, and how these explanations are intertwined with forgiveness processes. Secondarily, we wanted to check the effects of the self-help group intervention, analysing if the participants had an elaboration and/or a decrease of the feelings of self/other blame and an increase of forgiveness and acceptance of the relative’s suicide." | not described | Population: Participants in self-help groups for suicide survivors (n=10)  Data: Reports of narrations of the support group meetings.  Length of data collection: Up to 3 years | “Results show how self-blame was continuously present along all the period and how it increased when new participants entered the group. This finding indicates that self-blame characterizes especially the beginning of the participation, and that any new entrance rekindles the problem. However, no participant had ever definitively demonstrated self-forgiveness, while a general forgiveness appeared when self-blame stopped.”  Subheadings: 1) The thematic analysis: Phase 1—Timeline of the themes, 2) Thematic analysis: Phase 2—The Contents (Looking for causes (self-blame and other-blame), Forgiveness) |
| Theadom, A., Rutherford, S., Kent, B., & McPherson, K. (2019). The process of adjustment over time following stroke: A longitudinal qualitative study. Neuropsychological Rehabilitation, 29(9), 1464-1474.  Country: New Zealand | "This study aimed to explore how people adjust to life after stroke over time and to identify what helps or hinders recovery." | qualitative description methodology & qualitative longitudinal research | Population: People experienced a stroke (n=82)  Data: Individual interviews  Length of data collection: 36 months | “Participants described an ongoing process of shock, disruption, and fear, making sense of what happened, needing to fit in with what’s offered, finding what works for them and evolving a new normal, whilst managing the ups and downs of life. This process needed to be renegotiated over time, as people experienced changes in their recovery, comorbidities and/or wider circumstances. The adjustment process continued over the three years post-stroke, even for those who perceived that they were recovering well.”  Subheadings: 1) Shock, disruption and fear, 2) Making sense of what happened, 3) Needing to fit in with what’s offered, 4) Finding what works for me, 5) Evolving a new normal, 5) Managing the ups and downs |
| Theron, L., & van Rensburg, A. (2018). Resilience over time: Learning from school-attending adolescents living in conditions of structural inequality. Journal of Adolescence, 67, 167-178.  Country: South Africa | "Thus, the questions directing the study we report in this article were: Do the same (or different) resilience-enabling resources inform township-dwelling, school-attending adolescents' resilience accounts when they are invited to self-explain their resilience at two distinct points in time? Which resilience-enabling resources, if any, become significantly more (or less) salient over time and how do township-dwelling, school-attending adolescents explain the resilience-enabling value of these resources?" | Phenomenology & qualitative longitudinal research | Population: School-attending adolescents (n=140)  Data: Drawings combined with written reflections  Length of data collection: 24 months | “A comparison of school-attending adolescents' accounts of their resilience at two points in time revealed the longevity of nine, generic resilience-enabling resources. A comparison of how frequently adolescents reported these resources at Time 1 and 2 showed significant increases for education, faith-based supports, and peer support. A comparison of adolescents' reasons for identifying these three resources showed that education promises an improved future, while all three facilitate respite from hardship and/or mastery over current challenges.”  Subheadings: 1) Promise of a better personal and collective future, 2) Relief from hardship |
| Thomson, R., Martin, J. L., & Sharples, S. (2017). The experience of couples being given an oxygen concentrator to use at home: A longitudinal interpretative phenomenological analysis. Journal of Health Psychology, 22(6), 798-810.  Country: UK | "...the aim of this study was to follow couples and describe their experience as they began LTOT [Long-term oxygen therapy] to understand the issues associated with the device and how these may alter over time." | phenomenology | Population: Couples were one had COPD and oxygene at home (n=8)  Data: Joint interviews  Length of data collection: 5 months | “The findings reported here focus on two super-ordinate themes: ‘the journey of acceptance’ and ‘negotiating changing relationships’. Participants described a gradual process of accepting the device into their lives, the impact on couple’s relationships and the role that expectations have in mediating that process.”  Subheadings: 1) The journey of acceptance (Starting the journey, Along the path to acceptance), 2) Negotiating changing relationships (A source of conflict and harmony, Increasing emotional burden) |
| Thorpe, G., & McArthur, M. (2017). Social adaptation following intestinal stoma formation in people living at home: A longitudinal phenomenological study. Disability & Rehabilitation, 39(22), 2286-2293.  Country: UK | "...this paper explores the outward focus on a disrupted social world, highlighting how stoma-forming surgery impacts on individuals’ abilities to participate and interact socially and how these experiences can change over time." | phenomenology | Population: Individuals having had stoma-forming surgery (n=12)  Data: Individual interviews  Length of data collection: 15 months | “Three categories were identified: participation in the social environment; interpersonal relationships: changes and challenges; and setting and achieving goals.”  Subheadings: 1) Variations in the experience, 2) Participation in the social environment, 3) Interpersonal relationships: changes and challenges, 4) Setting and achieving goals, 5) Essential structure |
| Todis, B., McCart, M., & Glang, A. (2018). Hospital to school transition following traumatic brain injury: A qualitative longitudinal study. Neurorehabilitation, 42(3), 269-276.  Country: US | "Our objective was to better understand the complex variables that influence the hospital–school transition experience by analyzing parent and teacher experiences, impressions, and interactions." | Qualitative longitudinal research | Population: Parents and teachers (n=71)  Data: Individual interviews and observations  Length of data collection: About 4 years (range 0-6) | “This paper presents two themes that emerged from interview and observational data collected over a 7-year period: 1) provision of hospital-school transition services is inconsistent and 2) teachers lack sufficient awareness of and training in TBI to implement hospital transition recommendations. Regardless of the type and quality of transition support, most educators in the students’ schools remained unaware of the students’ needs and how to meet them.”  Subheadings: 1) Inconsistent approaches to hospital-school transition (Hospital shared no information about TBI with the school, Hospital shared information with the school, Parents shared information from the hospital with the school), 2) Lack of educator awareness about the student’s injury, 3) Transitioning to a new grade or school |
| Toly, V. B., Blanchette, J. E., Alhamed, A., & Musil, C. M. (2019). Mothers' voices related to caregiving: The transition of a technology-dependent infant from the NICU to home. Neonatal Network, 38(2), 69-79.  Country: US | "is to explore how mothers of technology-dependent infants perceive their transition experiences just prior to and during the first months after their infants’ initial discharge from the NICU to home" | Qualitative longitudinal research | Population: Mothers caring for an infant expected to be discharged home within two to three weeks for the first time dependent on medical technology (n=19)  Data: Individual interviews  Length of data collection: About 3 months | “Three themes were identified pretransition: negative emotions, positive cognitive-behavioral efforts, and preparation for life at home. Two posttransition themes were negative and positive transition experiences. Throughout the transition, the mothers expressed heightened anxiety, fear, and stress about life-threatening situations that did not abate over time despite the discharge education received.”  Subheadings: 1) Sample description (Mothers, Infants), 2) Major themes, 2:1) Pretransition themes, 2:2) Negative emotions, 2:3) Positive cognitive-behavioral efforts, 2:4) Preparation for life at home, 2:5) Posttransition themes, 2:6) Negative transition experiences, 2:7) Positive transition experiences, |
| Tornero, M. D. L. A., & Capella, C. (2017). Change during psychotherapy through sand play tray in children that have been sexually abused. Frontiers in Psychology, 8, 617-617.  Country: Chile | "The present study therefore seeks to analyze characteristics of play during therapy among child victims of sexual assault between the ages of 7 and 10. Three particular moments of the therapeutic process are observed using sand tray therapy." | not described | Population: Child victims of sexual assault between the ages of 7 and 10 (n=7)  Data: Observations  Length of data collection: 6 months | “Results of the present study reveal common and transversal forms of playful expression among this group of children shown by their engagement with sandplay. During this activity, participants elaborate personal stories that feature violence as a central theme, often involving aggression between two or more individuals. They also express their need for care and protection and work to resolve conflicts using fantasy. The shifting dynamics of sandplay at each stage of therapeutic treatment is an important finding that reveals the progress made during psychotherapy. In the third phase of treatment, sandplay encouraged movement among children, allowing them to act out meaningful scenarios and create structured situations with positive outcomes.”  Subheadings: 1) Play behavior (Use of sand, Categories employed in the activity, Characteristics of the activity, Use of time, Composition), 2) Content (Emerging topics, Character identity, Environmental conditions and personal needs, Feelings and emotional atmosphere, Story development) |
| Tsai, A., C., Hatcher, A. M., Bukusi, E. A., Weke, E., Lemus Hufstedler, L., Dworkin, S. L., Kodish, S., Cohen, C. R., & Weiser, S. D. (2017). A livelihood intervention to reduce the stigma of HIV in rural Kenya: Longitudinal qualitative study. AIDS And Behavior, 21(1), 248-260.  Country: Kenya | "...we conducted a qualitative study to explore longitudinal changes in stigma among persons with HIV participating in a randomized controlled trial in rural Kenya. Treatment-arm participants received a livelihood intervention, called Shamba Maisha (meaning ‘‘farming life’’ in Kiswahili), and our goal was to explore the intervention’s perceived impacts on HIV stigma and to understand the mechanisms through which any stigma-related changes may have occurred." | Qualitative longitudinal research | Population: Persons with HIV participating in a randomized controlled trial (n=54)  Data: Individual interviews  Length of data collection: 6 months | “Initially, participants felt ashamed of their seropositivity and were socially isolated (internalized stigma). They also described how others in the community discriminated against them, labeled them as being ‘‘already dead,’’ and deemed them useless and unworthy of social investment (perceived and enacted stigma). At follow-up, participants in the treatment arm described less stigma and voiced positive changes in confidence and self-esteem. Concurrently, they observed that other community members perceived them as active, economically productive, and contributing citizens. None of these changes were noted by participants in the control arm, who described ongoing and continued stigma.”  Subheadings: 1) Stigma prior to intervention (Felt stigma, Enacted stigma, Internalized stigma), 2) Changes experienced by participants (Changes in self-esteem, Changes in internalized stigma), 3) Changes in community perceptions (Change in status from ‘‘Unproductive’’ to ‘‘Contributing’’, Change in status from ‘‘Dying soon’’ to ‘‘Living’’, Change in status from ‘‘Burden’’ to ‘‘Benefactor’’), 4) Persistent stigma despite intervention, 5) Stigma in the absence of intervention |
| Tse, S., Mak, W. W. S., Lo, I. W. K., Liu, L. L., Yuen, W. W. Y., Yauc, S., Hod, K., Chan, S.-K., & Wong, S. (2017). A one-year longitudinal qualitative study of peer support services in a non-Western context: The perspectives of peer support workers, service users, and co-workers. Psychiatry Research, 255, 27-35.  Country: Hong Kong | "The present paper aims to identify the changes in the perceptions (concerning peer support services and their key ingredients) of PSWs [Peer support workers] among themselves, their co-workers (supervisors, mentors of the PSWs, and non-peer professional staff members), and service users at different community-based mental healthcare centers in Hong Kong over a 12 month period." | Qualitative longitudinal research | Population: Peer support workers, supervisors, mentors of the peer support workers, non-peer professional staff members, and service users (n=14)  Data: Individual interviews  Length of data collection: 12 months | “During the initial implementation of the services, uncertainty about the role of the PSWs [Peer support workers] were reported. However, trusting and beneficial relationships with service users were gradually built, showing growing resilience and confidence over time. The participants realized that PSWs’ experiences of mental illnesses were a unique asset that could help service users to alleviate their own somatic symptoms and improve their connections with others. Our findings highlight that the perceptions of peer support services changed from confusion to viewing PSWs as an asset, to an awareness of the importance of family support, and to the belief that implementing such a program will benefit both service users and PSWs”  Subheadings: 1) Perceptions over time about peer support workers (Essential ingredient of PSWs: turning mental illness-related experiences into assets, Initial uncertainty and confusion, PSWs’ growing resilience and confidence over time), 2) Service users’ changes over time: the challenges and promise of peer support services (Reduced levels of stress and pain, Improved connections with others) 3) The vital role of the family in supporting peer support workers (Supporting PSWs, Families’ concerns) |
| Tudor-Sfetea, C., Rabee, R., Najim, M., Amin, N., Chadha, M., Jain, M., Karia, K., Kothari, V., Patel, T., Suseeharan, M., Ahmed, M., Sherwani, Y., Siddiqui, S., Lin. Y., & Eisingerich, A. B. (2018). Evaluation of Two Mobile Health Apps in the Context of Smoking Cessation: Qualitative Study of Cognitive Behavioral Therapy (CBT) Versus Non-CBT-Based Digital Solutions. JMIR Mhealth and Uhealth, 6(4), e98.  Country: UK | "The purpose of this study was, therefore, first, to explore users’ perceptions of two mHealth apps, one CBT-based [cognitive behavioral therapy] app, Quit Genius (QG), and one non-CBT-based app, National Health Service (NHS) Smokefree (SF), over a variety of critical themes. Second, the study also sought to investigate the perceptions and health behavior with respect to smoking cessation for users of each app." | Qualitative longitudinal research | Population: Smoker who intends to quit (n=29)  Data: Individual interviews  Length of data collection: 1 week | “The thematic analysis resulted in five higher themes and several subthemes. Participants were generally more positive about Quit Genius’s features, as well as about its design and information engagement and quality. Quit Genius users reported increased motivation to quit smoking, as well as greater willingness to continue using their allocated app after 1 week. Moreover, these participants demonstrated preliminary changes in their smoking behavior, although this was in the context of our limited sample, not yet allowing for the finding to be generalizable.”  Subheadings: 1) Thematic analysis, 2) App features (Specific features, General features), 3) App design (Aesthetics and interface, Interactivity), 4) App content (Style of information, Information engagement, Quality of information), 5) App feasibility, 6) App effects, 7) App improvements, 8) Descriptive statistics, |
| Turnbull, J., Pope, C., Prichard, J., McKenna, G., & Rogers, A. (2019). A conceptual model of urgent care sense-making and help-seeking: a qualitative interview study of urgent care users in England. BMC Health Services Research, 19(1), 481.  Country: UK | "Our aim is to explicate how people make sense of urgent care through the work that they do when they experience a health problem, and in turn how this drives action e.g. seeking help from services or choosing not to seek help." | not described | Population: People seeking or not seeking health care (n=100)  Data: Individual interviews  Length of data collection: 12 months | “The amount and nature of the effort (work) undertaken to make sense of urgent care was an overarching theme of the analysis. We distinguished three distinct types of work: illness work, moral work and navigation work. These take place at an individual level but are also shared or delegated across social networks and shaped by social context and time. We have developed a conceptual model that shows how people make sense of urgent care through work which then influences help-seeking decisions and action.”  Subheadings: 1) The work of making sense of urgent care (Illness work, Moral work, Navigation work), 2) The relevance and influence of socio-temporal context |
| Vaghefi, I., & Tulu, B. (2019). The continued use of mobile health apps: Insights from a longitudinal study. JMIR Mhealth and Uhealth, 7(8), e12983.  Country: US | "To understand continued use of mHealth apps and individuals’ decisions related tothis behavior." | Qualitative longitudinal research | Population: University students (n=17)  Data: Individual interviews and diaries  Length of data collection: 2 weeks | “We identified 2 dimensions that help explain continued use decisions of users of mHealth apps: users’ assessment of mHealth app and its capabilities (user experience) and their persistence at their health goals (intent). We present the key factors that influence users’ assessment of an mHealth app (interface design, navigation, notifications, data collection methods and tools, goal management, depth of knowledge, system rules, actionable recommendations, and user system fit) and relate these factors to previous literature on behavior change technology design. Using these 2 dimensions, we developed a framework that illustrated 4 decisions users might make after initial interaction with mHealth apps (to abandon use, limit use, switch app, and continue use). We put forth propositions to be explored in future research on mHealth app use.”  Subheadings: 1) Overview, 2) Users’assessment of mobile health apps (Interface design, Navigation, Notifications, Data collection, Goal management, Depth of knowledge, System rules, Actionable recommendations, Fit between user and system, Users’ persistence at health goals), 3) Typology of mobile health app use decisions (Abandon use, Limit use, Switch app, Continued use) |
| Valdez, A., Nowotny, K. M., Zhao, Q.-W., & Cepeda, A. (2019). Interpersonal partner relationships, bonds to children, and informal social control among persistent male offenders. Social Problems, 66(3), 468-483.  Country: US | "We explore a qualitative examination of the men’s motivations, aspirations, and goals to lead conventional lives and desist from criminal behaviors." | Ethnography | Population: Young men who were gang affiliated as adolescents (n=40)  Data: Individual interviews, and field observations  Length of data collection: One month | “We analyzed semi-structured interviews conducted with a subsample (n=40) during the course of three face-to-face sessions to explore the men’s motivations, aspirations, and goals to lead conventional lives, despite their criminal justice involvement. Specifically, we focus on the complex nature of maintaining ties to children, the navigation of complicated family structures, the processes of seeking partners with economic resources, and on partnerships with criminal and delinquent partners. We document the complex interpersonal nature of these relationships as men contend with serial incarceration and their desires and motivations to desist from criminal behavior.”  Subheadings: 1) Maintaining ties to children, 2) Navigating complicated family structures, 3) Processes of seeking partners with economic resources, 4) “She parties and smokes too much weed”— Partnering with criminal and delinquent partners |
| Van Natta, M., Burke, N. J., Yen, I. H., Rubin, S., Fleming, M. D., Thompson‐Lastad, A., & Shim, J. K. (2018). Complex care and contradictions of choice in the safety net. Sociology of Health & Illness, 40(3), 538-551.  Country: US | "This article explores the complicated and often-contradictory notions of choice at play in complex care management (CCM) programmes in the US healthcare safety net." | ethnography | Population: Patients and providers in the clinic, patients’ homes, and other settings (n=108)  Data: Individual interviews and observations  Length of data collection: About 2 years | “By considering our data alongside philosopher Annemarie Mol’s (2008) conceptualisation of the competing logics of choice and care, we argue that these premises often undermine CCM [complex care management] teams’ efforts to support patients and provide the care they need – not only to prevent medical crises, but to overcome socio-economic barriers as well. We assert that while safety-net CCM programmes are held accountable for the degree to which their patients successfully transform into self-managing, cost-effective actors, much of the care CCM staff provide in fact involves attempts to intervene on structural obstacles that impinge on patient choice. CCM programmes thus struggle between an economic imperative to get patients to make better health choices and a moral imperative to provide care in the face of systemic societal neglect.”  Subheadings: 1) ‘Nobody ever asked me’, 2) ‘I have no choice!’, 3) ‘How do you think we can help you?’ |
| Vandenberg, B. E., Advocat, J., Hassed, C., Hester, J., Enticott, J., & Russell, G. (first published 2018). Mindfulness-based lifestyle programs for the self-management of Parkinson's disease in Australia. Health Promotion International, 2019, 34, 668–676.  Country: Australia | "To characterize the experiences of communityliving adults with Stage 2 PD [Parkinson's disease] participating in a facilitated, group mindfulness-based lifestyle program, and explore how the program influenced beliefs about self-management of their disease." | Qualitative longitudinal research | Population: Adult participants with Parkinson's disease participating in a mindfuldness program (n=16)  Data: Individual interviews  Length of data collection: 8 months | “Prior to the program, participants felt a lack of control over their illness. A desire for control and a need for alternative tools for managing the progression of PD motivated many to engage with the program. Following the program, where participants experienced an increase in mindfulness, many became more accepting of disease progression and reported improved social relationships and self-confidence in managing their disease.”  Subheadings: 1) Perception of control, 2) Acceptance of the disease, 3) Building self-confidence in disease management |
| VanHeuvelen, J. S. (2019). Isolation or interaction: Healthcare provider experience of design change. Sociology of Health & Illness, 41(4), 692-708.  Country: US | "To assess the experience of providers, I examine ethnographic and interview data gathered at ‘Children’s NICU’ as it moved from an open-bay to single-patient rooms." | Ethnography | Population: Health-care providers at children NICU (n=40)  Data: Individual interviews, observations and field notes  Length of data collection: 13 months | “Findings show that changing the structure of the NICU [neotonal intensive care unit] interfered with the original local organisational culture of collaboration. While providers actively worked to maintain the original culture, their success in doing so was mediated by the built environment. Responding to the new space, practitioners developed new practices. Some of the practices (such as doorway discussions and increased individual assessments) directly undermined the original organisational culture, whereas others (hallway hangouts and calling out) worked to transpose the original culture into the new space.”  Subheadings: 1) The local culture of collaboration, 2) Changes in the culture of collaboration, 3) Inconsistent practices, 4) Transposing local culture, 5) Mixed feelings about changes in the local culture |
| Varagona, L. M., & Hold, J. L. (2019). Nursing students' perceptions of faculty trustworthiness: Thematic analysis of a longitudinal study. Nurse Education Today, 72, 27-31.  Country: US | "The purpose of this study was to explore nursing students' perceptions of faculty trustworthiness. The study sought to identify characteristics and behaviors of nursing faculty that lead nursing students to trust them." | Qualitative longitudinal research | Population: Nursing students (n=77)  Data: Focus group interviews  Length of data collection: 2 years | “Three core themes emerged regarding the characteristics and behaviors of faculty members that lead nursing students to trust them: Giving ofOneself, Being Competent, and Having Integrity. The study findings provide guidance to nursing faculty regarding how to be perceived as trustworthy by students and how to avoid being perceived as untrustworthy.”  Subheadings: 1) Giving of oneself, 2) Being competent, 3) Having integrity |
| Vassilev, I., Rogers, A., Kennedy, A., Oatley, C., & James, E. (2019). Identifying the processes of change and engagement from using a social network intervention for people with long‐term conditions. A qualitative study. Health Expectations, 22(2), 173-182.  Country: UK | "An acceptable intervention which facilitates self- management by mobilizing network support and improves network engagement has a positive impact on health and quality of life. This study aims to identify the processes through which such changes and engagement take place" (from abstract) | Case study | Population: People living with long-term conditions (n=15)  Data: Observations, and individual intervews  Length of data collection: 3 months | “Intervention engagement leads to a deepening of relationships within networks, adding new links and achieving personal objectives relevant for improving the health and well- being of users and network members. Such changes are supported through two pathways: the mobilization of network capabilities and by acting as a nudge. The first is a gradual process where potentially relevant changes are further contemplated by forefronting immediate concerns and negotiating acceptable means for achieving change, prioritizing objective over subjective valuations of support provided by network members and rehearsing justifications for keeping the status quo or adopting change. The second pathway changes are enacted through the availability of a potential fit between individual, network and environmental conditions of readiness.”  Subheadings: 1) Building capacity for articulating, reframing and re-orientating relationships and capabilities (Negotiating objectives and engagement with network members, Forefronting evaluations of network support, Rehearsing justifications in engaging others), 2) Nudging a link to enabling environments and activated networks, 3) Environmental fragilities in engaging and sustaining practice change |
| Vestergren, S., Drury, J., & Chiriac, E. H. (2018). How collective action produces psychological change and how that change endures over time: A case study of an environmental campaign. British Journal of Social Psychology, 57(4), 855-877.  Country: Sweden | "The present research aimed to extend previous literature by giving an account of the emergence and endurance of psychological changes through participation in an environmental campaign. In the context of the campaign, we present a qualitative analysis of a longitudinal panel study with quantitative measures of interaction and amount of change. In addition, we explore the processes of endurance of these changes illustrated through a case study of consumer behaviour and consumption attitudes." | Ethnography | Population: Self-defined activists and locals (n=28)  Data: Individual interviews  Length of data collection: 18 months | “Thematic analysis of interviews suggested that participants linked the process of change to categorizing themselves in a new environmental-activist way that influenced their everyday lives beyond the immediate campaign. This recategorization was a result of a conflictual intergroup relationship with the police. The intergroup interaction produced supportive within-group relationships that facilitated the feasibility and sustainability of new world views that were maintained by staying active in the campaign.”  Subheadings: 1) Endurance and prevalence ofchange, 1.1) Intergroup interaction and categorization, 1.2) Intragroup interaction, 1.3) Case study: Consumer behaviour and endurance (Consumer behaviour and endurance) |
| Walker, K. C., Valentiner, L. S., & Langberg, H. (2018). Motivational factors for initiating, implementing, and maintaining physical activity behavior following a rehabilitation program for patients with type 2 diabetes: A longitudinal, qualitative, interview study. Patient Preference and Adherence, 12, 145-152.  Country: Denmark | "The primary objective of the present study is to address the gap in knowledge on motivational changes over time to improve adherence to physical activity from the initiation of behavioral change in a rehabilitation program, to implementation of physical activity outside the program, and, finally, to maintenance of physical activity behavior in everyday life." | Qualitative longitudinal research | Population: Patients with type 2 diabetes (n=5)  Data: Individual interviews  Length of data collection: One year | “Commitment and obligation were emphasized as being motivational in initiating physical activity. Toward the termination of the program, this was challenged by an expressed need for autonomy. Successful behavioral change was characterized by transfer of commitment to a new structure in everyday life, which also honored the request for autonomy. Feeling capable of participating in physical activity was facilitated through knowledge, practical experience, and progress and considered motivational, whereas lack of progress extinguished motivation. Finally, enjoyment of the activity was determining for long-term maintenance of physical activity behavior.”  Subheadings: 1) Balancing the need for commitment and a sense of choice (commitment fosters physical activity – baseline interview, Physical activity challenged by other commitments – 12-week interview, Transfer of commitment to a new structure – 52-week interview), Feeling competent and experiencing progress (Feeling capable of making a difference – baseline interview, Achievement facilitates motivation – 12-week interview, Knowledge on what works and proof that it matters – 52-week interview), 3) setup, surroundings, and the actual activity (Perceived appropriateness for engagement – baseline interview, The actual activity – 52-week interview) |
| Walker-Williams, H. J., & Fouché, A. (2018). Resilience enabling processes and posttraumatic growth outcomes in a group of women survivors of childhood sexual abuse. Health SA Gesondheid, 23, 1-9.  Country: South Africa | "To explore emerging resilience processes and posttraumatic growth outcomes in women survivors of childhood sexual abuse after attending the S2T [programme entitled S2T denoting from Survivor to Thriver]" | not described | Population: Women survivors of childhood sexual abuse (n=8)  Data: Individual interviews, and drawings  Length of data collection: About 12 months | “Qualitative thematic analysis revealed the following enabling resilience processes: group as healing vehicle of change; changing destructive to constructive rumination; coping strengths and meaning-making. Posttraumatic growth outcomes which evolved were: transforming wounded to healer; a changed life philosophy; excavated spirituality and rebriefing strengths through a posttrauma thriver identity.”  Subheadings: 1) Resilience enabling processes, 1:1) Group as healing vehicle of change (Normalising space, Witnessed by other survivors), 1:2) Changing destructive to constructive rumination (Self-awareness, Cognitive reframing and restructuring), 1.3) Coping strengths, 1.4) Experimenting with changed behaviour (Engaging in social support), 1.5) Meaning-making (Acceptance and purpose, Religious activities), 2) Posttraumatic growth outcomes, 2.1) Transforming wounded to healer (Need to help others, Giving unconditionally), 2.2) Changed life philosophy (Re-evaluation of self, Future perspective, Greater appreciation for life), 2.3) Excavated spirituality (Faith as a source of strength, Comfort through relationship with God), 2.4) Re-briefing strengths through post-trauma thriver identity (Painful sharpening’s make a better pencil, Acknowledging the new ‘me’) |
| Wang, C.-L., Kuo, L.-M., Chiu, Y.-C., Huang, H.-L., Huang, H.-L., Hsu, W.-C., Lu, C.-H., Huang, T.-H., Huang, S., & Shyu, Y.-I. L. (2018). Protective preparation: A process central to family caregivers of persons with mild cognitive impairment. International Psychogeriatrics, 30(3), 375-384.  Country: Taiwan | "Therefore, the purpose of this study was to explore and develop a theoretical model to explain the longitudinal changes in the caregiving process for family caregivers of persons with MCI [mild cognitive impairment] in Taiwan." | grounded theory | Population: Family caregivers of persons with MCI (n=13)  Data: Individual interviews  Length of data collection: 2 years | “One core theme emerged: ‘protective preparation.’ This reflected the family caregiving process of preparation for a further decline in cognitive function, and protection from the impact of low self-esteem, accidents, and symptoms of comorbidities for the family member with MCI. Protective preparation contained three components: ambivalent normalization, vigilant preparation, and protective management.”  Subheadings: 1) Study participants, 2) Protective preparation, 3) Component 1: Ambivalent normalization, 4) Component 2: Vigilant preparation, 5) Component 3: Protective management, 6) Illness trajectory of MCI as a contextual variable |
| Weatherred, J. L. (2017). Framing child sexual abuse: A longitudinal content analysis of newspaper and television coverage, 2002-2012. Journal of Child Sexual Abuse, 26(1), 3-22.  Country: US | "This study seeks to quantitatively explore how the media has framed CSA [Child Sexual Abuse], specifically whether it is framed as an individual or a societal level problem, from 2002 to 2012. The first research question seeks to establish the frequency of CSA news stories throughout the time period. RQ1: How often do news stories about CSA appear in U.S. television and newspaper coverage? Because previous research indicated a spike in news coverage that could be attributed to the Catholic Church, a quantitative measurement ofthis coverage was included in the coding sheet. The articles that were about Sandusky, the Catholic Church, the Boy Scouts, or other categories were coded. Therefore, the following research question is posed: RQ2: How much news coverage is about these specific CSA scandals? The next research question seeks to establish the nature of the news coverage, specifically if it is focused on individual or societal causes and solutions. RQ3: Is news coverage about CSA focused on individual or societal level causes and solutions? The fourth question seeks to determine whether differences in coverage of CSA exist between newspapers and television. RQ4: Do newspapers and television differ in whether they focus more on individual-level or societal causes and solutions for CSA?" In order to determine whether there have been changes in how the news media frame CSA, the following research question was posed: RQ5: Has the media’s framing of responsibility shifted from individual- to societal-level causes and solutions over time? | not described | Population: US national news organizations (8 medias, TV-channels and news papers)  Data: Documents (e.g., articles in daily press)  Length of data collection: 10 years | “Study findings indicate a shift in the attribution of responsibility of child sexual abuse among news organizations over the past decade from an individual-level problem with individual-level solutions to a societal-level problem with institutional culpability.”  Subheadings: No subheadings in result section |
| Weiser, S. D., Hatcher, A. M., Hufstedler, L. L., Weke, E., Dworkin, S. L., Bukusi, E. A., Burger, R. L., Kodish, S., Grede, N., Butler, L. M., & Cohen, C. R. (2017). Changes in health and antiretroviral adherence among HIV-infected adults in Kenya: Qualitative longitudinal findings from a livelihood intervention. AIDS & Behavior, 21(2), 415-427.  Country: Kenya | "Here we report on results of a longitudinal qualitative study that was carried out simultaneously alongside the trial and aimed to understand how and why the intervention may have affected participant health." | Qualitative longitudinal research | Population: People living with HIV (n=74)  Data: Individual interviews  Length of data collection: 12 months | “Intervention participants described improvements in HIV health, including increased CD4 counts and energy, improved viral suppression, and fewer HIV-related symptoms. Better health was linked to improved clinic attendance and ART adherence through several mechanisms: (1) reductions in food insecurity and abject hunger; (2) improved financial stability; (3) improved productivity which enhanced social support; (4) better control over work situations; and, (5) renewed desire to prioritize their own health.”  Subheadings: 1) Demographic characteristics, 2) Perceived impacts of the intervention on HIV related health, 3) Improvements in clinic attendance and ART adherence: Key pathways towards health impacts (Pathway 1: Improved food security, Pathway 2: Financial stability and income, Pathway 3: Productivity and social support, Pathway 4: Greater control over work situation, Pathway 5: Prioritization of health) |
| Wenham, A., Atkin, K., Woodman, J., Ballard, K., & MacPherson, H. (2018). Self-efficacy and embodiment associated with Alexander Technique lessons or with acupuncture sessions: A longitudinal qualitative sub-study within the ATLAS trial. Complementary Therapies in Clinical Practice, 31, 308-314.  Country: UK | "The ATLAS trial [1] has provided a useful platform from which to explore the participants' experiences and their perspectives of potential ways in which the changes in clinical outcomes came about. In particular, we aimed to explore in depth the role of learning, self-efficacy and self-care in sustaining benefits over the longer term." | Qualitative longitudinal research | Population: Participants with chronic neck pain who attended Alexander Technique lessons or acupuncture sessions (n=30)  Data: Individual interviews  Length of data collection: 6 months | “Five key themes emerged: pre-trial experiences of biomedical treatment against which subsequent interventions were compared; emergence of tangible benefits from the interventions; factors that contributed to the observed benefits, notably growing self-care and self-efficacy; a developing sense of embodiment as an integral part of the transformative process; and contribution of these factors to sustaining benefits over the longer term.”  Subheadings: 1) Pre-trial experiences of biomedical treatment, 2) Emergence of tangible benefits from the interventions, 3) Factors contributing to the observed benefits 4) Embodiment integral to the transformative process, 5) Sustaining benefit over time |
| Werner-Lin, A., Ersig, A. L., Mueller, R., Young, J. L., Hoskins, L. M., Desai, R., & Greene, M. H. (2018). Catalysts towards cancer risk management action: A longitudinal study of reproductive-aged women with BRCA1/2 mutations. Journal of Psychosocial Oncology, 36(5), 529-544.  Country: US | "The present study sought to identify factors that shape risk management planning, life experiences that shape the capacity or decision about when, how, or whether to enact plans, and the psychosocial impact of those adaptions. Towards this end, we integrated multiple data points over time with BRCA mutation carriers of reproductive age." | Qualitative longitudinal research | Population: Women with increased cancerrisk (n=12)  Data: Individual interviews  Length of data collection: 2 to 3.5 years | “All women shared only two action steps: seeking information about cancer risk and completing genetic testing. The constellation of action steps created a unique action trajectory that was defined, with precise ideas about risk perception and clear behavioral response, or iterative, in which unanticipated life events shifted the speed, accessibility, or order in which risk management and family planning goals were prioritized, planned, or executed. Factors shifting action steps included salient, unanticipated life events, such as infertility, insurance/financial constraints, birth of the last child, or a relative’s cancer diagnosis. Focus on cancer morbidity may obfuscate how women prioritize actions, and ignore varied pragmatic, relational, and social factors affecting how intended actions are completed, particularly during the reproductive years.”  Subheadings: 1) Description of,participants (Original sample (n = 60), Follow-up sample (n = 12)), 2) Summary of findings: Two trajectories and an evolving context. 2.1) Actions: From pre-test planning to post-test decision-making (Action prior to genetic testing, Actions during the genetic testing process, Action subsequent to genetic testing), 2.2) Factors catalyzing action: Dynamic contexts and circumstances (Infertility, Birth ofthe last child, Diagnosis in the same generation, Information seeking in medical and social networks) |
| Whiffin, C. J., Baker, D., Henshaw, L., Nichols, J. J., & Pyer, M. (2018). "Am I a student or a healthcare assistant?" A qualitative evaluation of a programme of prenursing care experience. Journal of Advanced Nursing, 74(11), 2610-2621.  Country: UK | "To examine the experiences of prenursing HCAs during a 6‐month programme of prenursing care experience. Determine the benefit of the experience to the individual participant and their ambition to become a Registered Nurse. • Examine how the experience informs participant's values and behaviours in clinical practice. • Identify key strengths and challenges in the delivery of formal programmes of prenursing care experience." | constructivist approach & qualitative longitudinal research | Population: Health care asisstants in prenursinng positions (n=23)  Data: Focus group interviews  Length of data collection: 6 months | “Five major themes were identified in the analysis of qualitative data: personal development; positioning of role in the healthcare team; support and supervision; perceived benefits; and advice and recommendations. These themes were underpinned by deep aspirations for better care and better nurses in the future.”  Subheadings: 1) Personal development (The journey: motivation, affirmation, confirmation, ready!, An exploration and examination of nurses and nursing), 2) Positioning of role in the healthcare team (Who am I?, Developing competence in the HCA role, Saturation and stagnation—what now, what else?), 3) Support and supervision (Getting it right, Getting it wrong), 4) Perceived benefits, 5) Advice and recommendations |
| Wiklund, M., Ahlgren, C., & Hammarström, A. (2018). Constructing respectability from disfavoured social positions: Exploring young femininities and health as shaped by marginalisation and social context. A qualitative study in Northern Sweden. Global Health Action, 11, 1519960.  Country: Sweden | "The aim of this research was to elucidate constructs of femininities in relation to structuring living conditions and expressions of health in Northern Swedish women. The time period of interest was the transition from unemployed teenagers to young adults in a social context of high unemployment and societal change across the critical ‘school-towork-transition’ life course period." | Qualitative longitudinal research | Population: Youth not in employment, education or training (n=7)  Data: Individual interviews  Length of data collection: 15 years | “The results are presented as the theme of ‘constructing respectability from disfavoured social positions’. Within this theme, and framed by dominant norms of patriarchal femininity, we explored the constructs of normative and altruistic, norm-breaking, and troubled femininity.”  Subheadings: 1) Constructing respectability from disfavoured social positions, 2) Normative and altruistic femininity, 3) Norm-breaking femininity, 4) Troubled femininity |
| Winkel, A. F., Robinson, A., Jones, A. A., & Squires, A. P. (2019). Physician resilience: A grounded theory study of obstetrics and gynaecology residents. Medical Education, 53(2), 184-194.  Country: US | "to create a conceptual model of resiliencein the context of OBGYN [obstetric and gynaecology] residency." | Grounded theory | Population: Obstetrics and gynaecology residents in postgraduate year (n=18)  Data: Individual interviews  Length of data collection: 3 to 6 months | “A conceptual model for resilience as a socio-ecological phenomenon emerged. Resilience was linked to professional identity and purpose served to root the individual and provide a base of support through adversity. Connections to others inside and outside medicine were essential to support developing resilience, as was finding meaning in experiences. The surrounding personal and professional environments had strong influences on the ability of individuals to develop personal resilience.”  Subheadings: 1) Resilience grows along with professional identity, 2) Environment influences developing resilience, 3) Residents must balance personal and professional selves dynamically, 4) Struggles distract from engaging with meaningful aspects of work, 5) Conceptual model of resilience in residents |
| Woodgate, R. L., Zurba, M., Edwards, M., Ripat, J. D., & Rempel, G. (2017). The embodied spaces of children with complex care needs: Effects on the social realities and power negotiations of families. Health & Place, 46, 6-12.  Country: Canada | "This paper presents research findings that advance knowledge around the power and agency families with children with complex care needs (CCN). Our conceptual framework uses concepts from geography towards situating the experiences and social realities of family carers within the ‘embodied space of care’." (from abstract) | ethnography & qualitative longitudinal research | Population: Children with complex care needs, their parents, and siblings (n=92)  Data: Individual interviews and photovoice  Length of data collection: 3 years | “Findings reveal that interactions and decision-making processes relating to health and everyday life were complex and socially interconnected, and emphasize the need for provisions for family-based decision-making and enhanced social inclusion of families and the importance of the renegotiation of power.”  Subheadings: 1) Participants, 2) Embodied and relational spaces of children with CCN, 3) Power negotiations of family carers |
| Wu, F. K. Y., Shek, D. T. L., Chai, C. W. Y., & Zhu, J. X. Q. (2019). Well-being of university students in Hong Kong: A longitudinal case study. International Journal of Child & Adolescent Health, 12(1), 99-107.  Country: Hong Kong | "...to investigate the differences of views and suggest how these influences have influenced the understanding and perception of the assistance of GUR [general university requirements]." | Case study | Population: Freshmen at university (n=42)  Data: Individual interviews, informal meetings, and field notes  Length of data collection: About one year | “Three major observations on students’ views about the effectiveness of GUR [general university requirements] study were generated from the study, including 1) broadened horizons; 2) increased awareness of the connection between GUR study and all-round development and 3) urge of linking GUR components with one’s profession. The longitudinal case study findings suggest that students generally had fruitful learning experiences in GUR study.”  Subheadings: 1) Trusting and reciprocal relationships, 2) Broadened horizons, 3) Increased awareness of connection between GUR study and all-round development, 4) Urge for linking GUR components with one’s profession |
| Yalcin Arslan, F. (2019). Reflection in pre-service teacher education: Exploring the nature of four EFL pre-service teachers' reflections. Reflective Practice, 20(1), 111-124.  Country: Turkey | "this study assessed the nature of student-teachers’individual reflections throughout the phases of school experience, teaching practice, and entry into the workplace. For this purpose, structured reflection was employed in order togain more thorough knowledge regarding this matter. [...] (1) What is the nature of EFL pre-service teachers’reflections during their teaching practicum? (2) What is the nature of EFL teachers’reflection upon their entry into the workplace?" | Case study | Population: Student and teachers at university (n=4)  Data: Individual interviews  Length of data collection: 3 months | “The findings of this interpretation indicated that although most of the reflections concerned environment, behavior, and competence (outer level of the onion model), some of the reflections nevertheless pertained to mission (inner level of the onion model). Moreover, an examination of participants’ individual reflection processes revealed that although reflections initially were outerlevel in nature, by the end of the study they had become more inner-level-oriented.”  Subheadings: No subheadings in result section |
| Yeh, E. A., Chiang, N., Darshan, B., Nejati, N., Grover, S. A., Schwartz, C. E., Slater, R., & Finlayson, M., for the Pediatric MS Adherence Study Group (2019). Adherence in Youth With Multiple Sclerosis: A Qualitative Assessment of Habit Formation, Barriers, and Facilitators. Qualitative Health Research, 29(5), 645-657.  Country: US and Canada | "Therefore, we sought to understand the social and lifestyle factors that influence medication adherence in youth with MS, from the perspective of the youth, over time." | Qualitative longitudinal research | Population: Youth with MS (n=28)  Data: Observations (e.g., transcripts from motivational interview sessions during the intervention)  Length of data collection: 3 months | “Youth described medication adherence as being dependent on the ability to build and maintain healthy habits related to medication use, including embodiment of these habits. Barriers and facilitators included remembering/forgetting, experiences with fatigue, and experiences with medication. These themes were maintained through the second and third interviews.”  Subheadings: Themes, 1) Central theme: Building and maintaining healthy habits (Remembering versus forgetting, Experience with fatigue), 2) Subtheme: Feeling motivated (Extrinsic motivation, Intrinsic motivation), 3) Subtheme: Creating a safe space (Emotional security, Experience with medication), 4) Change through time |
| Young, J. L., Werner-Lin, A., Mueller, R., Hoskins, L., Epstein, N., & Greene, M. H. (2017). Longitudinal cancer risk management trajectories of BRCA1/2 mutation-positive reproductive-age women. Journal of Psychosocial Oncology, 35(4), 393-408.  Country: US | "The current study examined data from follow-up interviews with a subset of Hoskins’ (2010) original cohort. We identified whether and how risk perceptions, management strategies, and family planning decisions articulated at baseline were put into practice. The goals were to learn how closely participants followed their initial plan and what factors, including life events, influenced their final decisions." | grounded theory & qualitative longitudinal research | Population: BRCA1/ 2 mutation-positive women (n=12)  Data: Individual interviews  Length of data collection: 3 years | “To cope with the conflict between cancer risk reduction versus plans for pregnancy, breastfeeding, and child rearing, participants deliberately prioritized either risk reducing surgery or family formation goals.”  Subheadings: 1) BRCA1/2 mutation status: Moving beyond the initial adjustment period, 2) Variable pathways to risk reduction, 3) Pathways to the “new normal”: Shifting between the foreground and the background, 4) Family formation in the foreground/in the background (Family formation in the foreground, Family formation in the background), 5) BRCA1/2 in the foreground/in the background (BRCA1/2 in the foreground, BRCA1/2 in the background) |
| Zadvinskis, I. M., Garvey Smith, J., & Yen, P.-Y. (2018). Nurses' experience with health information technology: Longitudinal qualitative study. JMIR Medical Informatics, 6(2), e38-e38.  Country: US | "This was a longitudinal qualitative study that explored medical-surgical nurse perceptions of HIT [health information technology] implementation over time. We interviewed nurses three times after EHR and bar code medication administration (BCMA) implementation to capture evolving adaptation of their perceptions and behaviors in this specific job role. The objective of the study was to explore nurses’ experience of HIT implementation, and how they adapted their perceptions and behavior to HIT upgrades and optimization over time" | Phenomenology & qualitative longitudinal research | Population: Nurses (n=19)  Data: Individual interviews  Length of data collection: 15 months | “Nurses reported personal-level and organizational-level factors that facilitated HIT [health information technology] adaptation. We also generated network graphs to illustrate the occurrence of themes. Thematic interconnectivity differed due to nurses’ concerns and satisfaction at different time points. Equipment and workflow were the most frequent themes across all three rounds. Nurses were the most dissatisfied approximately 9 months after HIT implementation. Eighteen months after HIT implementation, nurses’ perceptions appeared more balanced.”  Subheadings: 1) Principal findings, 2) Thematic findings, 2.1) E1: Nurses’ interaction with HIT, 2.2) E2: Nursing performance regarding task accomplishment, 2.3) E3: Unit-specific teamwork, 2.4) E4: Interdisciplinary teamwork, 2.5) E5: Quality of care (Adaptation, Organizational factors), 3) Visualization of theme and subtheme interrelationships |
| Zuurmond, M., Nyante, G., Baltussen, M., Seeley, J., Abanga, J., Shakespeare, T., Collumbien, M., & Bernays, S. (2019). A support programme for caregivers of children with disabilities in Ghana: Understanding the impact on the wellbeing of caregivers. Child: Care, Health & Development, 45(1), 45-53.  Country: Ghana | "...to study the interplay of various factors at different levels of a social system (family, support group, and community) affecting changes in the caregiver. [...] The research questions addressed in this paper are to explore (a) in what ways the intervention impacted upon the caregiver wellbeing and (b) the change process that caregivers engaged in, in order to inform future strengthening of the intervention." | Qualitative longitudinal research | Population: Care-givers to children with celeral palsy (n=15)  Data: Individual interviews  Length of data collection: 12 months | “Low levels of knowledge, high levels of stigma, physical and emotional exhaustion, and often difficult family relationships with social exclusion of the child and caregiver were common themes at the outset. Caregivers struggled to combine their caring and economic activities. This was exacerbated by the common absence of the father. Two months after completion of the training, their reported wellbeing had improved. The reasons for this were an improved understanding about their child's condition, positive attitudinal change towards their child, feelings of hope, and through the group support, a profound realisation that they are ‘not on their own.’ While relationships within the family remained complex in many cases, the support group offered an important and alternative social support network.”  Subheadings: 1) Our case study families, 2) Understanding of the condition, 3) Caring practice, 4) Stigma and social inclusion/exclusion, 5) Caregiver mental health, 6) Poverty |
